# Visible Light Activation of Boronic Esters Enables Efficient Photoredox C(sp^2^)–C(sp^3^) Cross‐Couplings in Flow

**DOI:** 10.1002/anie.201605548

**Published:** 2016-10-06

**Authors:** Fabio Lima, Mikhail A. Kabeshov, Duc N. Tran, Claudio Battilocchio, Joerg Sedelmeier, Gottfried Sedelmeier, Berthold Schenkel, Steven V. Ley

**Affiliations:** ^1^ Department of ChemistryUniversity of CambridgeLensfield RoadCambridgeCB2 1EWUK; ^2^ Novartis Pharma AGNovartis Campus4002BaselSwitzerland

**Keywords:** boronic esters, cross-coupling, flow chemistry, photoredox catalysis, synthetic methods

## Abstract

We report herein a new method for the photoredox activation of boronic esters. Using these reagents, an efficient and high‐throughput continuous flow process was developed to perform a dual iridium‐ and nickel‐catalyzed C(sp^2^)–C(sp^3^) coupling by circumventing solubility issues associated with potassium trifluoroborate salts. Formation of an adduct with a pyridine‐derived Lewis base was found to be essential for the photoredox activation of the boronic esters. Based on these results we were able to develop a further simplified visible light mediated C(sp^2^)–C(sp^3^) coupling method using boronic esters and cyano heteroarenes under flow conditions.

Visible light photoredox catalysis has emerged as a powerful tool to trigger and control carbon radical reactions under mild and environmentally benign conditions.^1^ In particular, new C−C bond forming reactions have been developed using this novel activation mode.^2^ Classical palladium‐catalyzed C(sp^2^)–C(sp^3^) cross‐coupling reactions are especially challenging owing to a slow transmetallation step of C(sp^3^) nucleophiles and competitive β‐hydride elimination.^3^ Concomitantly reported by Molander^4^ and MacMillan^5^ in 2014, dual photoredox/nickel catalysis has become a powerful protocol to construct C(sp^2^)–C(sp^3^) bonds under mild conditions.^6^ In particular, Molander's approach makes use of the ability of highly oxidizing iridium photocatalysts to afford single‐electron oxidation of organotrifluoroborate salts in order to produce carbon‐centred radicals (Figure 1).^7^ These photo‐generated C(sp^3^) radical species can then be used in the nickel catalytic cycle to afford a variety of cross‐coupled products.^4^, ^8^ Despite the elegance of these new methods, severe solubility issues associated with the use of charged trifluoroborate, carboxylate^9^ or silicate^10^ salts often require the use of polar aprotic solvents or solvent mixtures (such as DMF or DMSO) at low concentrations (typically <0.1 m). These diluted conditions result in long irradiation times (24 to 48 h), low throughput and are difficult to scale‐up. Moreover, these water soluble, high boiling point solvents are not compatible with the REACH regulation and pose issues for downstream processing.^11^ A more soluble source of organic radical precursor for use in a more acceptable solvent would therefore be beneficial.^12^

**Figure 1:**
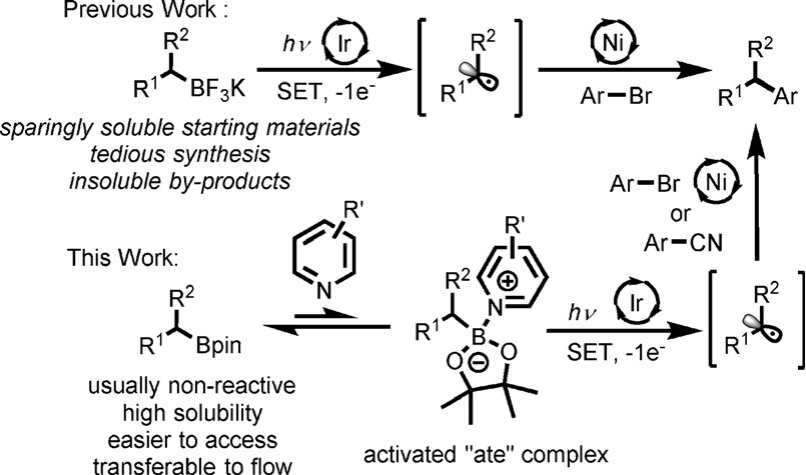
Use of boronic pinacol esters for photoredox C(sp^3^) radical generation.

Boronic esters are suitable precursors as they are widely available, both from commercial and synthetic sources.^13^ Although organoboron species have been extensively described as tin‐free source of alkyl radicals,^14^ only a limited number of studies have focused on the use of boronic esters as alkyl radical precursors.^15^ Despite the growing interest of generating carbon radicals using trifluoroborate salts, no reports to date for the activation of boronic esters using photoredox catalysis have been made.^16^ Therefore, we anticipated that by the use of a suitable activating method, these species could be used as starting materials for photoredox activation and thereby expand the scope of carbon radical chemistry.

Finally, solving solubility issues associated with these reactions would enable the use of continuous processing methods which could provide additional advantages. For instance, more efficient irradiation with microchannel devices and shorter residence times often result in faster and cleaner photo‐reactions in flow.^17^ Moreover, due to the inherent limitation of light attenuation through absorbing media, resulting in a small light penetration depth,17a batch photo‐reactors become inefficient when scaling up a reaction.^18^ Given our expertise in continuous processing13e, 19 and with the recent availability of suitable equipment,^20^ we aimed to develop a flow system using a photoredox catalyst to activate boronic esters in order to achieve a fast and scalable C(sp^2^)–C(sp^3^) coupling process.

To begin this study, reaction conditions similar to those reported by Molander^4^ were examined with the exception that **cat(1)**=[Ir(dF(CF_3_)ppy)_2_dtbpy]PF_6_ (dF(CF_3_)ppy= 2‐(2,4‐difluorophenyl)‐5‐(trifluoromethyl)pyridine; dtbpy=4,4′‐di‐*tert*‐butyl‐2,2′‐bipyridine) was used instead of **cat(2)**=[Ir(dF(CF_3_)ppy)_2_bpy]PF_6_ (bpy=2,2′‐bipyridine) as the initial photoredox catalyst in acetone (Table 1).

**Table 1:** Effect of the pyridine‐derived base additive on the dual Ir/Ni‐catalyzed cross‐coupling using benzyl boronic esters in flow.^[a]^ 
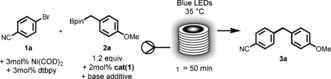

Entry	Base additive	*K*_eq_^[b]^	Yield [%]^[d]^
1	none	–	0
2	2,6‐lutidine	5.1×10^−12^	0
3	Pyridine	8.1×10^−4^	47
4	DMAP	0.30^[c]^	82
**5**	**DMAP**	**0.30^[c]^**	**87 (82^[e]^)**

[a] On 0.25 mmol of **1 a** (0.1 m in acetone). [b] Calculated equilibrium constant between **2 a** and the base additive at 298 k in acetone using ωB97xD/cc‐PVTZ+SMD‐solvation level of theory (see section 3 of the SI). [c] Experimental value observed is *K*_eq_=0.8 (for more details see the SI, Figure S1). [d] NMR yields calculated versus CH_2_Br_2_ as an internal standard in the crude ^1^H‐NMR. [e] Isolated yield.

Using trifluoroborate salts, clogging issues were immediately observed in the flow equipment due to the rapid precipitation of insoluble potassium salts. When switching to the commercial boronic pinacol ester (**2 a**), a fully homogeneous solution in acetone was obtained but we failed to observe any cross‐coupling product with 4‐cyanobromobenzene (**1 a**). (Table 1, entry 2).

Initial investigations highlighted the dramatic effect of the nature of the pyridine‐derived base additive on the yield of the reaction. In particular, without any base additive (entry 1) or using 2,6‐lutidine (entry 2, used by Molander), no product formation was observed, while using the less sterically hindered pyridine or the more electron‐rich 4‐(dimethylamino)pyridine (DMAP) resulted in a greatly enhanced formation of product (entries 3 and 4). This behavior correlates with calculated equilibrium constants of the corresponding base complexing with **2 a**. It was postulated therefore that a Lewis acid–base adduct ^[21]^ formed between the base additive and **2 a** served as a reactive intermediate. NMR experiments confirmed a fast and reversible dynamic complex formation between DMAP and **2 a** (for more details see the Supporting Information (SI), Figure S1). Additionally, in silico studies of the resulting complexes confirmed that complexation favors the postulated single‐electron oxidation process and the subsequent formation of the reactive benzylic radical from the pinacol ester **2 a** (Figure 4; for more details see the SI, Schemes S1–S3). Based on these promising preliminary results, we decided to explore the scope of this method and compare it with the existing batch method^4^ using trifluoroborate salts (Figure 2).

**Figure 2:**
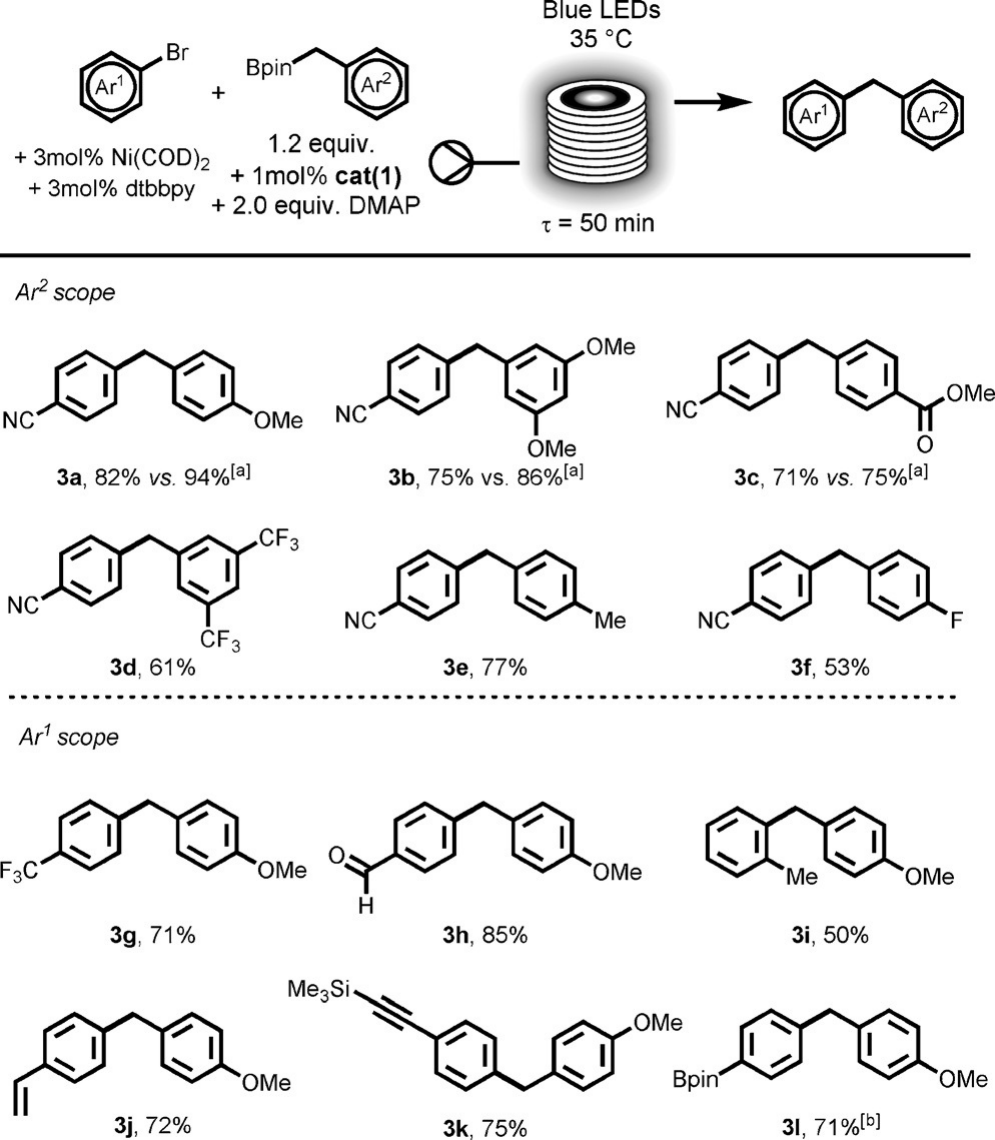
Scope of the dual Ir/Ni‐catalyzed benzyl boronic ester arylation in flow (0.5 mmol scale, 0.1 m in acetone). Isolated yields, a slug containing all the premixed reaction mixture was eluted through the photo‐reactor at 100 μL min^−1^, collected, filtered through a plug of Celite and concentrated before being purified by flash column chromatography. [a] Isolated yield reported by Molander for the product.^4^ [b] Isolated as the phenol after oxidation of the aryl boronic pinacol ester with H_2_O_2_/NaOH.

In general, the flow process using boronic esters resulted in slightly lower yields than the previously reported examples (**3 a** to **3 c**). However, the reaction time is dramatically decreased, from 24 h in batch (with trifluoroborate salts)^4^ to 50 min in flow, thus significantly increasing the productivity.^22^ As an example, space‐time‐yield (STY) of **3 a** with regard to the batch conditions is 2 mmol h^−1^ L(reactor)^−1^ whereas the use of a flow photo reactor, allows us to reach an impressive 100 mmol h^−1^ L(reactor)^−1^ of **3 a**. This clearly shows the massive intensification of the process due to the solubility improvement, more efficient light absorption and elevated pressure of the flow system enabling reactions which involve association.^23^

The reaction scope revealed that electron‐rich organoboron compounds were converted in good to excellent yields (**3 a**,**b**,**e**) whereas compounds bearing electron‐withdrawing substituents were associated to lower isolated yields of coupled products (**3 c**,**d**,**f**). This is consistent with the putative single‐electron oxidation mechanism, since higher electron density will make the boronates more reactive towards oxidation. The aryl bromide coupling partners could be varied tolerating the presence of sensitive aldehyde (**3 h**), alkene (**3 j**) and alkyne (**3 k**) groups. Remarkably an *ortho*‐substituent (**3 i**) is also well tolerated. The use of a boronic ester containing aryl bromide (**3 l**) served to confirm the orthogonality between the C(sp^2^) and the C(sp^3^) coupling events as previously described with trifluoroborate salts.^24^

Despite the benefits proved by the replacement of benzylic trifluoroborate salts by their boronic ester counterparts, large amount of additives is still necessary and the use of Ni(COD)_2_ (COD=cyclooctadienyl) required the preparation in a glovebox. Since these issues arise from the specific activation of aryl bromides, we envisaged that using other electrophiles could greatly simplify the reaction conditions. In particular, the MacMillan group reported elegant photoredox arylation methods using electron‐deficient cyanoarenes as single‐electron acceptors.^25^ These cyanoarenes could receive an electron from a photoredox catalyst to generate persistent radical anions ^[26]^ that can couple with other transiently generated radicals via a postulated radical–radical coupling pathway.^24^,^24^ Therefore, as the boronic esters should require a single‐electron oxidation and the cyanoarenes a single‐electron reduction, a net neutral photoredox coupling process could ensue with only a photoredox catalyst.1a

Among the cyanoarenes employed by MacMillan, the 4‐cyanopyridine scaffold was of particular interest since it would also serve as a Lewis base that could bind to boronic esters and potentially circumvent the use of DMAP additive. The investigation commenced using 4‐cyanopyridine (**4 a**) as a model substrate. Slight modifications of the previous reaction conditions were required to couple **4 a** and **2 a** (see SI, Figure S3). As expected, a good yield of **5 a** was achieved within 100 min of irradiation at 60 °C using 1 mol % of **cat(1)** without the use of DMAP or any other additives. These conditions highlighted again the advantages of the flow setup for photomediated reactions where temperature could be precisely controlled. Although a longer residence time was necessary to achieve good yields, the concentration in starting material could be raised from 0.1 m to 0.25 m thus allowing similar STYs to the previous process using aryl bromides.

The scope of this new reaction was then explored (Figure 3). Electronic effects of the pinacol boronic esters followed the same reactivity trend as in the previous reaction, with more electron‐rich substrates providing higher yields. Secondary benzylic boronic esters proceeded in higher yields than their primary counterparts due to the higher stability of secondary alkyl radicals. *Para*‐, *meta*‐ (**5 j** and **5 l**) and even bis *ortho*‐substituted (**5 k**) benzyl boronic esters could also be successfully arylated using this method. Screening the cyanoarene partner revealed that only nitrogen‐containing heteroaromatic nitriles could be successfully coupled. Interestingly, N‐heterocycles are generally seen as catalyst inhibitors but proved to be crucial in our reaction. For example, the commonly employed 1,4‐dicyanobenzene was ineffective under these conditions, probably because this substrate could not activate the boronic ester due to a lack of coordination.^27^ As already observed by MacMillan, 4‐cyanopyridine was one of the most successful cyanoarene of those examined (**5 a** to **5 d**). Pleasingly, in addition to cyanopyridine, our transformation was successfully applied to several N‐heterocycles (**5 e** to **5 r**, Figure 3). Variations around the 4‐cyanopyridine scaffold were also possible with 4‐cyanoquinoline (**5 n**) and other nitrile substituted 4‐cyanopyridines derivatives (**5 o** and **5 p**) giving very interesting coupled products with selective coupling at the most electron‐poor 4‐position (the rest of the mass balance being unreacted cyanoarene). Interestingly, the reaction could tolerate the unprotected 4‐cyano‐7‐azaindole (**5 m**).

**Figure 3:**
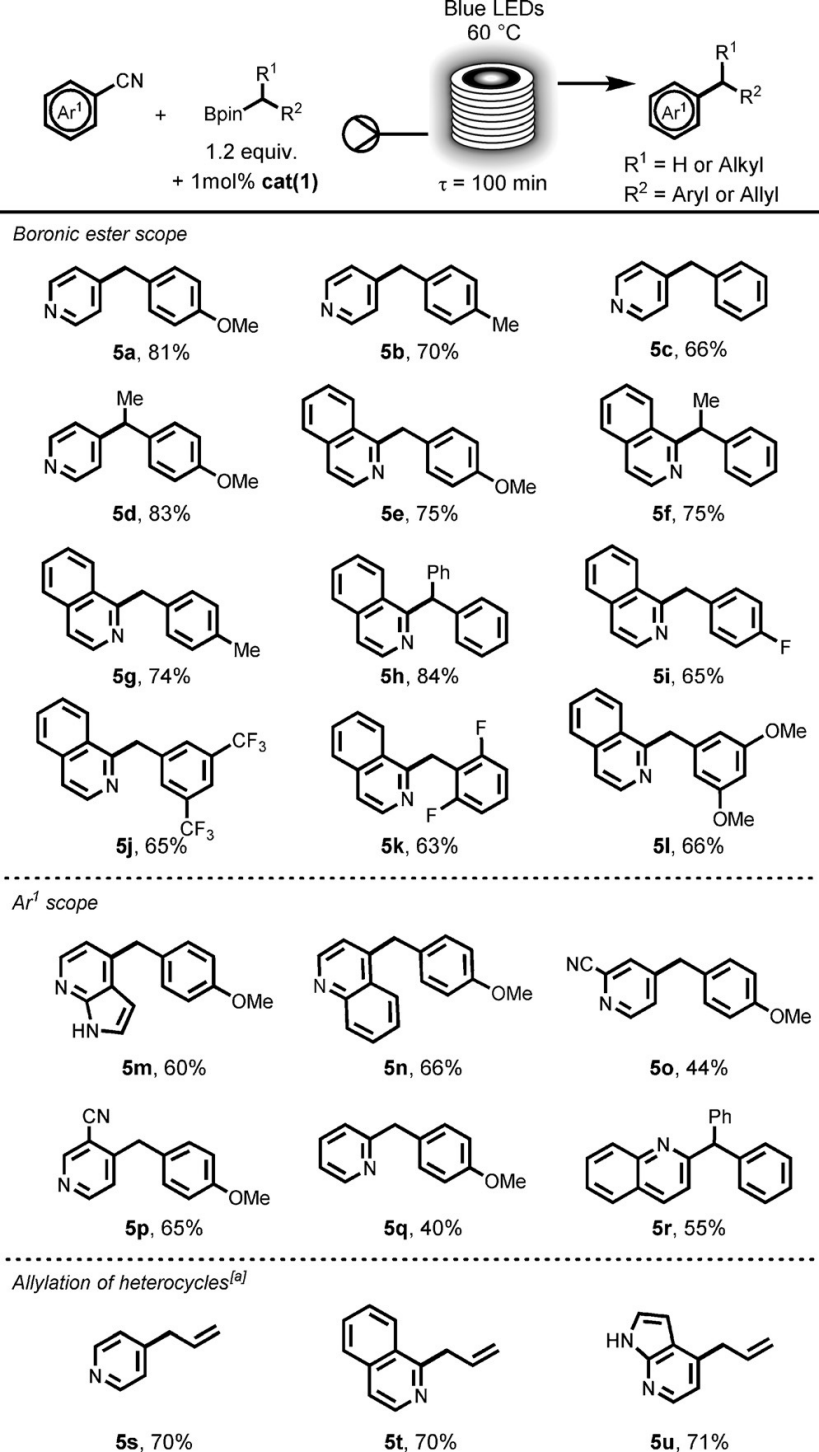
Scope of photoredox benzyl and allyl boronic esters arylation with cyanoarenes in flow (for benzyl: 0.25 mmol scale, 0.25 m in acetone). Isolated yields, a slug containing all the premixed reaction mixture was eluted through the reactor at 100 μL min^−1^, collected and concentrated before being purified on flash column chromatography. [a] Allylation experiments were performed on a 1.0 mmol scale (0.5 m in acetone), using 2.5 equivalents of the allylboronic acid pinacol ester and 0.5 mol % of **cat(1)** at 200 μL min^−1^ (*τ*=50 min).

In the effort to increase the method's diversity, we were able to extend the standard reaction conditions for the allylation of N‐heterocycles (e.g. **5 s** to **5 u**) using the commercially available allylboronic acid pinacol ester.

To rationalize the mechanism of this last protocol, calculations of equilibrium constants for Lewis acid–base adducts and their corresponding single‐electron reduction and oxidation potentials were performed at DFT level (for more details see the SI, Schemes S2 and S3). These results suggest that, as in the case of DMAP, 4‐cyanopyridine (**4 a**) and the boronic ester **2 a** are likely to form a complex **6** (Figure 4). This complex formation facilitates the single‐electron oxidation of **2 a** (*E*_1/2_ (**6**)=0.73 V vs. *E*_1/2_ (**2 a**)=1.57 V).^28^ This value makes this SET event possible within a reductive quenching cycle of **cat(1)** (=***Ir*** ^***III***^), ^[29]^ in agreement with that postulated by Molander.^4^ Based on our assumption, the excited **[*Ir*** ^***III***^ ]* species (*E*_1/2_^III*/II^=1.21 V)^30^ is first quenched by **6** (*E*_1/2_=0.73 V) leading, after rapid C−B bond cleavage (1.7 kcal mol^−1^ barrier), to a carbon‐centered radical **7** and the pyridinium **40**. The ***Ir*** ^***II***^ (*E*_1/2_^III/II^=−1.37 V)^30^ species thus generated can immediately reduce the activated pyridinium **40** (*E*_1/2_=−0.32 V),^28^ generating the radical **8** that quickly couples with **7** to form an intermediate that eliminates cyanide to give the coupled product **5 a**.

**Figure 4:**
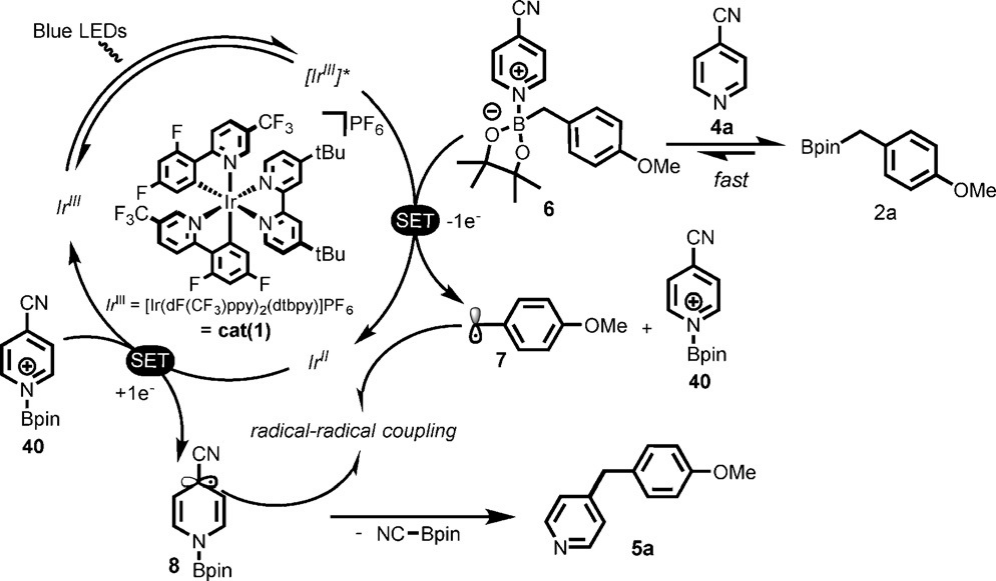
Proposed mechanistic description for the photoredox net neutral coupling of cyanoarenes with organo boronic esters.

In summary, we have demonstrated a new activation mode of boronic esters that allow them to react under photoredox conditions by formation of a complex with a pyridine‐like Lewis base. This modification not only enhanced the existing chemistry with trifluoroborate salts but also facilitated its application using flow chemistry. These results enabled the development of an efficient new C(sp^2^)–C(sp^3^) photoredox coupling process using heteroaromatic nitriles and pinacol boronic esters, whereby no additive other than the photoredox catalyst was required as the nitrogen‐containing heteroarene served as an activator of the boronic ester partner.

## Supporting information

As a service to our authors and readers, this journal provides supporting information supplied by the authors. Such materials are peer reviewed and may be re‐organized for online delivery, but are not copy‐edited or typeset. Technical support issues arising from supporting information (other than missing files) should be addressed to the authors.

Supplementary

## References

[1] For general reviews on visible light photoredox catalysis see:

[1a] C. K. Prier , D. A. Rankic , D. W. C. MacMillan , Chem. Rev. 2013, 113, 5322–5363; 23509883 10.1021/cr300503r PMC4028850

[1b] J. M. R. Narayanam , C. R. J. Stephenson , Chem. Soc. Rev. 2011, 40, 102–113; 20532341 10.1039/b913880n

[1c] R. A. Angnes , Z. Li , C. R. D. Correia , G. B. Hammond , Org. Biomol. Chem. 2015, 13, 9152–9167; 26242759 10.1039/c5ob01349f

[1d] K. Zeitler , Angew. Chem. Int. Ed. 2009, 48, 9785–9789; 10.1002/anie.200904056 19946918

[2] T. Koike , M. Akita , Inorg. Chem. Front. 2014, 1, 562–576.

[3] A. De Meijere , F. Diederich , Metal-Catalyzed Cross-Coupling Reactions, Wiley-VCH, Weinheim, 2004.

[4] J. C. Tellis , D. N. Primer , G. A. Molander , Science 2014, 345, 433–436. 24903560 10.1126/science.1253647 PMC4406487

[5] Z. Zuo , D. T. Ahneman , L. Chu , J. A. Terrett , A. G. Doyle , D. W. C. MacMillan , Science 2014, 345, 437–440. 24903563 10.1126/science.1255525 PMC4296524

[6] For a review on dual nickel and photoredox catalysis see: Y.-Y. Gui , L. Sun , Z.-P. Lu , D.-G. Yu , Org. Chem. Front. 2016, 3, 522–526.

[7] Original description of trifluoroborate salts as alkyl radical precursor: Y. Yasu , T. Koike , M. Akita , Adv. Synth. Catal. 2012, 354, 3414–3420.

[8a] D. N. Primer , I. Karakaya , J. C. Tellis , G. A. Molander , J. Am. Chem. Soc. 2015, 137, 2195–2198; 25650892 10.1021/ja512946e PMC4466116

[8b] I. Karakaya , D. N. Primer , G. A. Molander , Org. Lett. 2015, 17, 3294–3297. 26079182 10.1021/acs.orglett.5b01463 PMC4854197

[9] J. Xuan , Z. G. Zhang , W. J. Xiao , Angew. Chem. Int. Ed. 2015, 54, 15632–15641; 10.1002/anie.201505731 26509837

[10] C. Lévêsque , L. Chenneberg , V. Corcé , J.-P. Goddard , C. Ollivier , L. Fensterbank , Org. Chem. Front. 2016, 3, 462–465.

[11] L. Delhaye , A. Ceccato , P. Jacobs , C. K̈ttgen , A. Merschaert , Org. Process Res. Dev. 2007, 11, 160–164.

[12] P. Isnard , E. Guntrum , T. Senac , P. Cruciani , Org. Process Res. Dev. 2013, 17, 1517–1525.

[13] For recent methods to synthesize benzylic pinacol esters:

[13a] T. C. Atack , R. M. Lecker , S. P. Cook , J. Am. Chem. Soc. 2014, 136, 9521–9523; 24955892 10.1021/ja505199u

[13b] J. W. Clary , T. J. Rettenmaier , R. Snelling , W. Bryks , J. Banwell , W. T. Wipke , B. Singaram , J. Org. Chem. 2011, 76, 9602–9610; 22040316 10.1021/jo201093u

[13c] H. Li , L. Wang , Y. Zhang , J. Wang , Angew. Chem. Int. Ed. 2012, 51, 2943–2946; 10.1002/anie.201108139 22328139

[13d] W. N. Palmer , J. V. Obligacion , I. Pappas , P. J. Chirik , J. Am. Chem. Soc. 2016, 138, 766–769; 26714178 10.1021/jacs.5b12249

[13e] C. Battilocchio , F. Feist , A. Hafner , M. Simon , D. N. Tran , D. M. Allwood , D. C. Blakemore , S. V. Ley , Nat. Chem. 2016, 8, 360–367. 27001732 10.1038/nchem.2439

[14] C. Ollivier , P. Renaud , Chem. Rev. 2001, 101, 3415–3434. 11840989 10.1021/cr010001p

[15] We could only find one reference of carbon radical generation using boronic esters: C. Ollivier , P. Renaud , Angew. Chem. Int. Ed. 2000, 39, 925–928; 10.1002/(sici)1521-3773(20000303)39:5<925::aid-anie925>3.0.co;2-m 10760895

[16] G. Duret , R. Quinlan , P. Bisseret , N. Blanchard , Chem. Sci. 2015, 6, 5366–5382. 10.1039/c5sc02207j PMC5502392 28717443

[17a] Y. Su , N. J. W. Straathof , V. Hessel , T. Noël , Chem. Eur. J. 2014, 20, 10562–10589; 25056280 10.1002/chem.201400283

[17b] M. Neumann , K. Zeitler , Org. Lett. 2012, 14, 2658–2661; 22587670 10.1021/ol3005529

[17c] J. W. Beatty , C. R. J. Stephenson , J. Am. Chem. Soc. 2014, 136, 10270–10273; 25003992 10.1021/ja506170g PMC4233208

[17d] D. Cambié, C. Bottecchia, N. J. W. Straathof, V. Hessel, T. Noël, *Chem. Rev* **2016**, DOI: 10.1021/acs.chemrev.5b00707; 10.1021/acs.chemrev.5b00707 26935706

[17e] F. R. Bou-Hamdan , P. H. Seeberger , Chem. Sci. 2012, 3, 1612–1616.

[18] K. Loubière , M. Oelgemöller , T. Aillet , O. Dechy-Cabaret , L. Prat , Chem. Eng. Process. Process Intensif. 2016, 104, 120–132.

[19a] D. N. Tran , C. Battilocchio , S.-B. Lou , J. M. Hawkins , S. V. Ley , Chem. Sci. 2015, 6, 1120–1125; 10.1039/c4sc03072a PMC5811102 29560199

[19b] J.-S. Poh , D. N. Tran , C. Battilocchio , J. M. Hawkins , S. V. Ley , Angew. Chem. Int. Ed. 2015, 54, 7920–7923; 10.1002/anie.201501538 PMC4515080 26013774

[19c] R. J. Ingham , C. Battilocchio , D. E. Fitzpatrick , E. Sliwinski , J. M. Hawkins , S. V. Ley , Angew. Chem. Int. Ed. 2015, 54, 144–148; 10.1002/anie.201409356 PMC4502965 25377747

[19d] S. V. Ley , D. E. Fitzpatrick , R. M. Myers , C. Battilocchio , R. J. Ingham , Angew. Chem. Int. Ed. 2015, 54, 10122–10136; 10.1002/anie.201501618 PMC4834626 26193360

[20] M. Baumann , I. R. Baxendale , React. Chem. Eng. 2016, 1, 147–150.

[21] For complexation of boron atoms with nitrogen bases see:

[21a] W. B. Reid , J. J. Spillane , S. B. Krause , D. A. Watson , J. Am. Chem. Soc. 2016, 138, 5539–5542; 27104749 10.1021/jacs.6b02914 PMC4957246

[21b] R. B. Coapes , et al., J. Chem. Soc. Dalton Trans. 2001, 1201–1209.

[22] Batch reactions using boronic esters were not going to completion using Molander's conditions,^[4]^ we observed nickel deactivation (see SI, S10).

[23] Reactions with negative volume of activation are faster at higher pressure. See: http://goldbook.iupac.org/V06644.html.

[24] Y. Yamashita , J. C. Tellis , G. A. Molander , Proc. Natl. Acad. Sci. USA 2015, 112, 12026–12029. 26371299 10.1073/pnas.1509715112 PMC4593084

[25a] A. McNally , C. K. Prier , D. W. C. MacMillan , Science 2011, 334, 1114–1117; 22116882 10.1126/science.1213920 PMC3266580

[25b] K. Qvortrup , D. A. Rankic , D. W. C. MacMillan , J. Am. Chem. Soc. 2014, 136, 626–629; 24341523 10.1021/ja411596q PMC3988471

[25c] J. A. Terrett , M. D. Clift , D. W. C. MacMillan , J. Am. Chem. Soc. 2014, 136, 6858–6861; 24754456 10.1021/ja502639e PMC4333594

[25d] J. D. Cuthbertson , D. W. C. MacMillan , Nature 2015, 519, 74–77. 25739630 10.1038/nature14255 PMC4378681

[26] D. Mangion , D. R. Arnold , Acc. Chem. Res. 2002, 35, 297–304. 12020167 10.1021/ar010108z

[27] No stable complex between 1,4-dicyanobenzene and pinacol ester **2 a** could be located in silico.

[28] All half-wave reduction potentials are given in volts against the saturated calomel electrode (SCE). See SI (§3.1) for calculation method.

[29] Stern–Volmer quenching experiments will be part of a future work and will be reported in due course to confirm this hypothesis.

[30] For photocatalyst potentials see: K. Teegardin , J. I. Day , J. Chan , J. Weaver , Org. Process Res. Dev. 2016, 20, 1156–1163. 27499607 10.1021/acs.oprd.6b00101 PMC4972501

