# Supplementary material for: Visible Light Activation of Boronic Esters Enables Efficient Photoredox C(sp2)–C(sp3) Cross‐Couplings in Flow
Source: Angew Chem Int Ed Engl. 2016 Oct 6;55(45):14085–9. doi: 10.1002/anie.201605548 (PMC5113707; doi:10.1002/anie.201605548)
Supplement: Supplementary file 1 — Supplementary [file ANIE-55-14085-s001.pdf]

Angewandte Chemie
Eine Zeitschrift der Gesellschaft Deutscher Chemiker

Supporting Information

Visible Light Activation of Boronic Esters Enables Efficient Photoredox C(sp^2)–C(sp^3) Cross-Couplings in Flow

*Fabio Lima, Mikhail A. Kabeshov, Duc N. Tran, Claudio Battilocchio, Joerg Sedelmeier, Gottfried Sedelmeier, Berthold Schenkel, and Steven V. Ley**

anie_201605548_sm_miscellaneous_information.pdf

Contents

1. General information 3
2. Complexation between 2a and DMAP 5
3. Calculation of equilibrium constants and single electron oxidation/reduction potentials 6
3.1 Computational methods 6
3.2 Calculations results 7
4. Optimisation and control experiments 10
4.1 Dual catalysed coupling in Flow 10
4.2 Photoredox Coupling in Flow 11
5. Synthesis and characterisation of starting materials 12
5.1 Synthesis of non-commercial benzylic boronic acid pinacol esters starting materials 12
6. Synthesis and characterisation of coupling products 16
6.1 Dual Ir/Ni catalysed coupling of benzyl pinacol esters with aryl bromides in continuous flow. 16
6.1.1 General procedure I: GP(I) 16
6.1.2 Characterisation of coupling products 16
6.2 Flow photoredox coupling of pinacol esters with cyanoarenes 21
6.2.1 General Proedure II: GP(II) 21
6.2.2 Characterisation of coupling products 22
6.2.3 General procedure III: GP(III): 29
7. NMR spectra 31
7.1 Organoboron starting materials 31
7.2 Coupling Products 44
7.2.1 Dual Ir/Ni catalysed arylation of benzylboronic esters 44
7.2.2 Beznzyl Pinacolborolanes Flow Arylation with cyanoarenes 56
8. Cartesian coordinates of all computed structures optimised at ωB97xd/cc-PVDZ + SMD level of theory and their uncorrected electronic energies (in Hartrees). 78
9. References 127

1 General information

Photoredox catalysts, cyanoarenes, commercially available organoborons and benzyl bromides starting materials were used as received without further purification.

Analytical thin layer chromatography (TLC) was performed on pre-coated silica gel glass plates (Merck 60 F_{254}), visualised using ultraviolet light (254 nm or 365 nm for fluorescent compounds) or an alkaline solution of potassium permanganate. Purification by flash chromatography was carried out using an automated purification system (Biotage SP4 unit) with prepacked flash columns from Silicycle (PN: FLH-R10030B-ISO04) or Biotage SNAP KP-NH (PN: FSN0-0909-001). Crudes were loaded onto columns using dry loading technique with Isolute HM-N (Biotage PN: 9800-5000).

^{1}H-NMR spectra were recorded on a Bruker Avance DPX-400 or DRX-600 spectrometer at 400 and 600 MHz respectively and are reported as follows: chemical shift δ in ppm (multiplicity, coupling constants *J* in Hz, number of protons, assignment). The multiplicity and shape of the ^{1}H signals are designated by the following abbreviations: s = singlet, d = doublet, t = triplet, q = quartet, m = multiplet, br. = broad, or combinations of thereof. All chemical shifts δ are reported to the nearest 0.01 ppm with the residual solvent peak as the internal reference (chloroform-d = 7.26 ppm, methanol-d^4 = 3.31 ppm, acetone-d^6 = 2.05 ppm). ^{13}C-NMR spectra were recorded on the same spectrometers at 100 and 150 MHz with ^{1}H decoupling. All ^{13}C resonances are reported to the nearest 0.1 ppm with the central resonance of the solvent peak as the internal reference (chloroform-d = 77.16 ppm, methanol-d^4 = 49.00 ppm, acetone-d^6 = 29.84 ppm). The ^{13}C signal of the carbon bonded to boron was not observed in some cases due to quadrupolar relaxation. ^{19}F-NMR spectra were recorded on a Bruker DPX-400 spectrometer at 376 MHz with ^{1}H decoupling. All chemical shifts δ are reported to the nearest 0.1 ppm with $CFCl_3$ as the external standard ($CFCl_3$ = 0.0 ppm). ^{11}B-NMR NMR spectra were recorded on a Bruker DPX-400 or DRX-600 spectrometer at 128 MHz and 193 MHz respectively with ^{1}H decoupling. All chemical shifts δ are reported to the nearest 0.1 ppm with $BF_3{\cdot}OEt_2$ as the external standard ($BF_3{\cdot}OEt_2$ = 0.0 ppm). Spectra are assigned using ^{1}H-COSY, ^{13}C-DEPT-135 and HMQC where appropriate to facilitate structural determination.

Infrared spectra were recorded neat on a PerkinElmer Spectrum One FT-IR spectrometer using Universal ATR sampling accessories. High resolution mass spectrometry (HRMS) was performed using a Waters Micromass LCT Premier™ spectrometer using time of flight mass detection and positive ESI ionization method. All reported values are within 5 ppm of the calculated value.

Melting points were recorded on a Stanford Research Systems OptiMelt Automated Melting Point System calibrated against vanillin (m.p. 83 °C), phenacetin (m.p. 136°C) and caffeine (m.p. 237°C).

The removal of solvent under reduced pressure was carried out on a standard rotary evaporator. Photochemical flow reactions were performed using a Vapourtec E-series platform equipped with the UV-150 module.[1] This module consists of a temperature controlled irradiation chamber where a transparent fluorinated ethylene polymer (FEP) reactor (1 mm i.d., 10 mL,

PN: 50-1287) is coiled around a blue LED assembly (emitting at 420 nm with a total output power of 17 W, PN: 50-4036).

2 Complexation between 2a and DMAP

When studying the ^{1}H-NMR spectrum of the mixture **2a** and DMAP (**23**) in acetone-d^6 we could observe that gradually increasing the total concentration of DMAP ($[DMAP]_T$) in the NMR tube had a shielding effect on the benzylic CH_2 signal (see protocol[2] and **Figure S1**). This is a proof of a fast dynamic complex formation where weighted average signals of the complex **26** and **2a** + **23** are observed. [2] We also tried to separate signals at lower temperature (at 228 K and 213 K) but signals were still fully averaged at this temperature meaning that the equilibrium is faster than NMR measurement timescale even at these low temperatures.

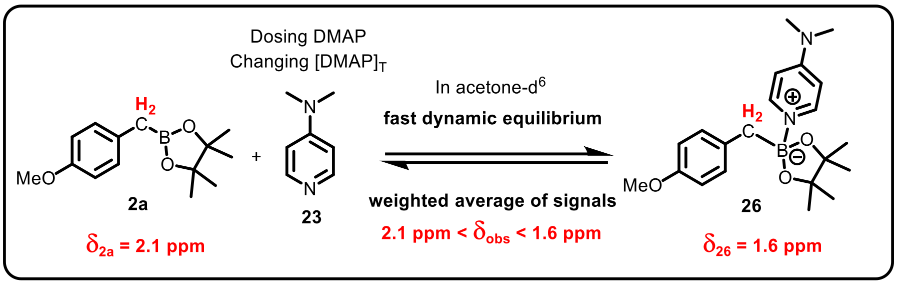

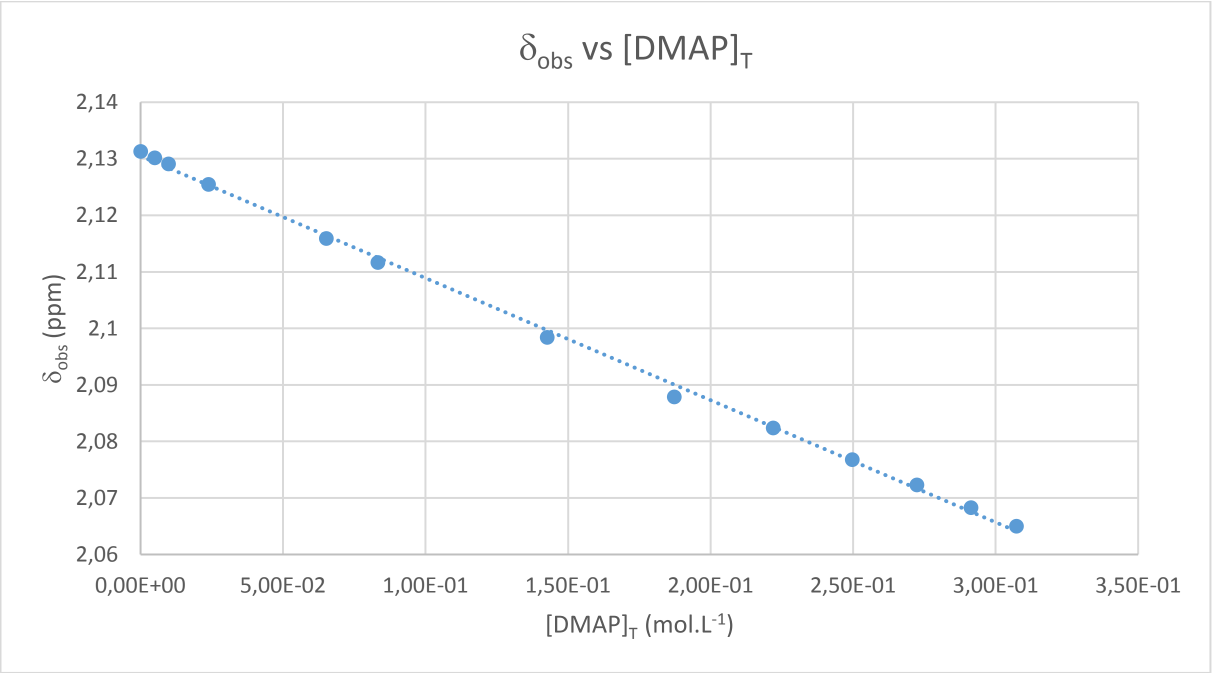

Figure S1 – Chemical shift of benzylic CH_2 of **2a** at different DMAP concentrations

Following the protocol described by Espenson[2] we were able to extrapolate the equilibrium constant (K_{eq} = 0.8) and the chemical shift of the complex (δ_{26} = 1.6 ppm) which was close to the calculated equilibrium constant (K_{eq} = 0.3).

3 Calculation of equilibrium constants and single electron oxidation/reduction potentials

3.1 Computational methods

Geometries of all structures (minima and saddle points) were optimized at the ωB97xd/cc-pVDZ level[3–5] in acetone as a solvent (using the SMD solvation model[6] $\varepsilon = 20.493$) using Gaussian 09 software;[7] subsequent vibrational frequency calculations were performed at the same level for all calculated structures. Transition states found possess exactly one negative Hessian eigenvalue, while all other stationary points were confirmed to be genuine minima on the potential energy surface (PES). Intrinsic reaction coordinate (IRC) analysis was performed to unambiguously assign located transition states when needed. Electronic energies were obtained by performing single point calculations at the ωB97xd/cc-pVTZ level in solvent. Gibbs energies were calculated as $\Delta G = \Delta H - \mathrm{T}\Delta S$ at 298 K where enthalpies and entropies were obtained by using standard statistical mechanical formulae for the ideal gas, rigid rotor, and harmonic oscillator approximations following the normal-mode analysis in vacuum. A correction of $(1.9 \cdot \Delta n)$ kcal.mol^{-1} (corresponding to the difference between the concentration of the ideal gas at 298 K and 1 atm and its 1 mol.L^{-1} concentration; Δn is the change in number of moles in the reaction) has been applied in order that the computed values refer to 1 mol.L^{-1} standard state.

All redox potentials reported in the manuscript are relative and calculated using single electron oxidation of potassium benzyltrifluoroborate **17** ($E_{1/2}^{red}$ = 1.10 V)[8] and single electron reduction of 1,4-biscyanobenzene **12** ($E_{1/2}^{red}$ = -1.61 V)[9] as two experimental reference points describing redox properties of the structurally similar compounds. Also, the calculated single electron reduction potential of **40** ([4-CN-pyridine-Bpin]$^+$; $E_{1/2}^{red}$ = -0.37 V) is comparable to the previously measured reduction potentials of slightly more electron-rich alkyl pyridinium salts ($E_{1/2}^{red}$ = -0.62 V to -0.85 V).[10]

3.2 Calculations results

Results for the calculation of the standard reaction Gibbs energy for SET events of the non-activated substrates using iridium species from the reductive quenching cycle of **cat(1)** are depicted in **Scheme S1**.

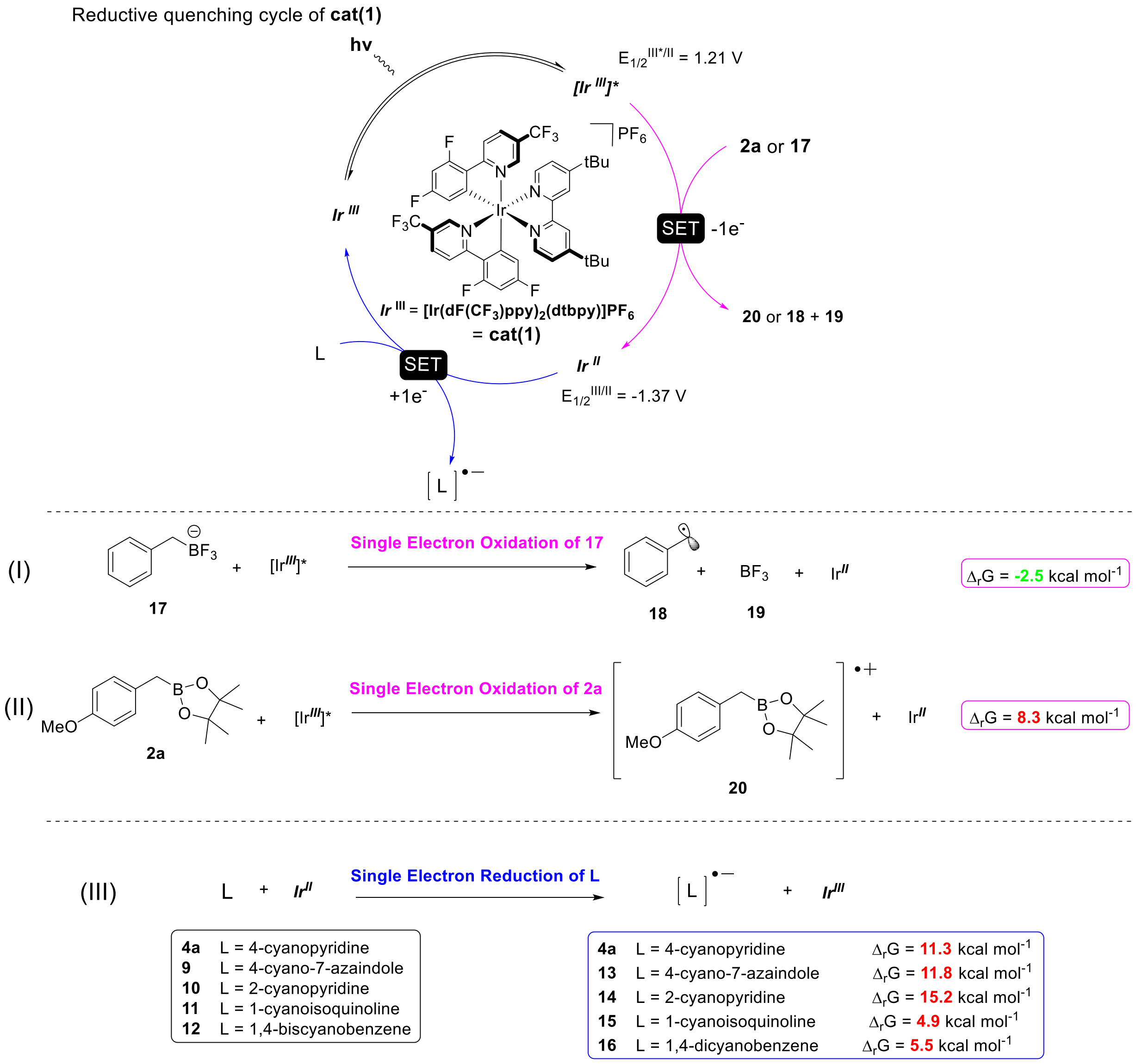

Scheme S1. The single electron oxidation-reduction reactions of **non-activated** substrates (Gibbs energies for single electron transfer reactions calculated using $E_{1/2}^{III*/II}$ = 1.21 V and $E_{1/2}^{III/II}$ = -1.37 V).

It can be observed from these calculations that trifluoroborate salt **17** can donate an electron to the light excited [***Ir^III^***]* to spontaneously generate the dissociated benzyl radical **18** and BF_3 **19** (eq. (I)). However, under the same conditions, the single electron oxidation of benzyl boronic acid pinacol ester **2a** was not observed to lead to a dissociation and was attributed a positive reaction Gibbs energy meaning that the process is not thermodynamically favourable (eq. (II)). On the single electron reduction side, all cyanoarene computed (**4a** and **9**–**12**) are not able to accept an electron from the ***Ir^II^*** (eq. (III)). It can be noted that in his arylation methods employing cyanoarenes, MacMillan employed the more potent single electron donors $Ir(ppy)_3$[9] and $Ir(pF(^tBu)ppy)_3$[11] photoredox catalysts to make these happen. These observations made us

believe that activation of these species will be required to make them react under photoredox conditions. As a fast and reversible dynamic complex formation was observed to be happening between **2a** and DMAP (**23**), we postulated that the same behaviour could be observed with other *N*-heterocycles. Therefore, complexation of our model boronic ester **2a** with various Lewis bases (**4a**, **9**, **11**, **12** and **21**–**23**) was calculated (**Scheme S2**). Relationship between standard reaction Gibbs energy and equilibrium constants ($\Delta G = -RT\ln(K_{eq})$) was used to calculate the complexation constants between **2a** and the Lewis bases.

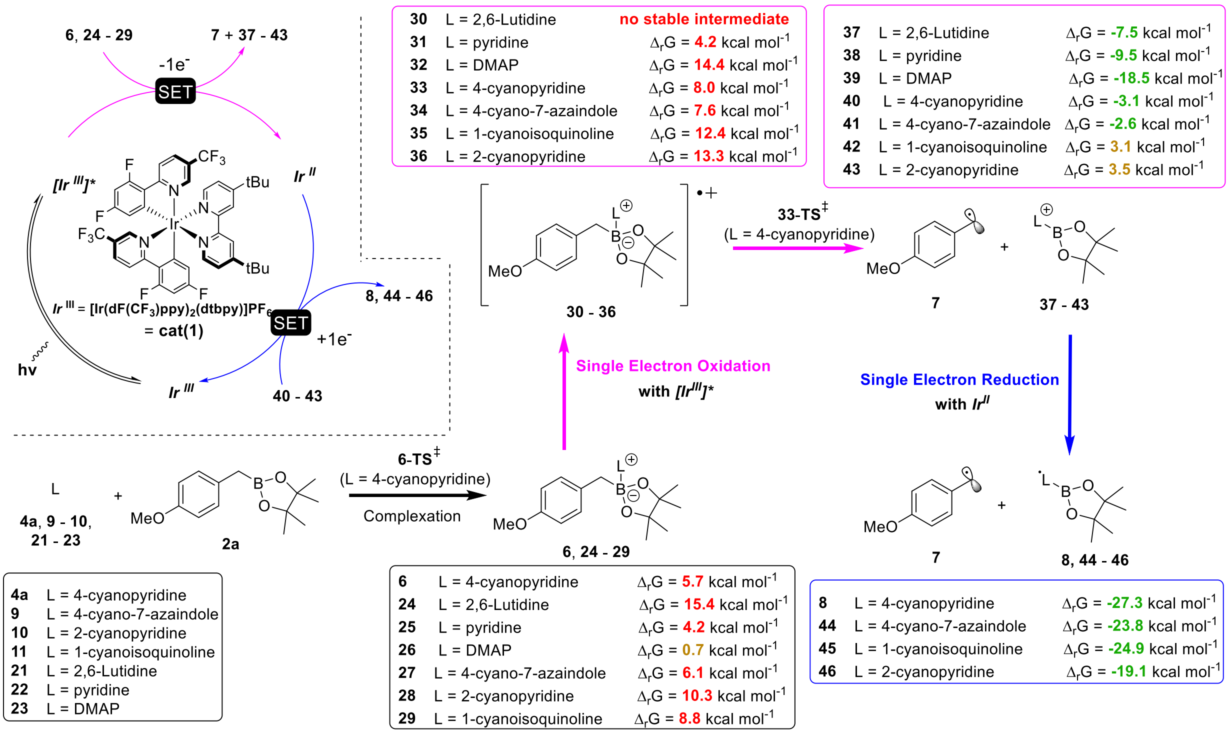

Scheme S2 The single electron oxidation and reduction reactions of activated substrates (reaction Gibbs energies for single electron transfer reactions calculated using $E_{1/2}^{III*/II} = 1.21$ V and $E_{1/2}^{III/II} = -1.37$ V; mixture **2a** + L is set as a ground state for all the transformation)

Formation of complexes **6**, **24**–**29**, although being endergonic, in the following steps leads to thermodynamically favorable C–B cleavage and the radical **7** formation (compare reaction Gibbs energies of **7** + **37** – **41** in **Scheme S2** vs. **20** in **Scheme S1** where no dissociation could occur). C–B bond cleavage is characterised by a low barrier (1.7 kcal mol^{-1} for L = 4-cyanopyridine, **33-TS**) thus happening spontaneously after the single electron oxidation step. As the formation of **7** + **39** is the most favourable ($\Delta_r G$ = -18.5 kcal.mol^{-1}), DMAP is expected to be the most efficient catalyst among other bases. At the same time, the formation of the sterically hindered complex **24** with 2,6-lutidine is thermodynamically unfavourable and for its single electron oxidation, there could not be located any stable intermediate **31** on the potential energy surface (**Scheme S2**). Resulting heterocyclic cationic borates **40** – **43** (produced after C–B cleavage) have been identified as the most plausible substrates for single electron reduction, an essential step in the proposed mechanism. Indeed, the formation of the radicals **8**, **44**–**46** from the cationic intermediates **40**–**43** is extremely favourable (**Scheme S2**). The

resulting two radicals could then easily engage in radical-radical coupling to result after cyanide elimination to the aromatised products.

To better illustrate the transformation discussed in the mechanism (see **Figure 4**), energy diagram for the reaction of **2a** with **4a** was drawn representing the energies of all the intermediates and transition states involved in the mechanism (**Scheme S3**). No transition states for pure single electron transfer events were located as we postulated that these steps only consist of a pure vertical excitation with minor nuclear rearrangements.

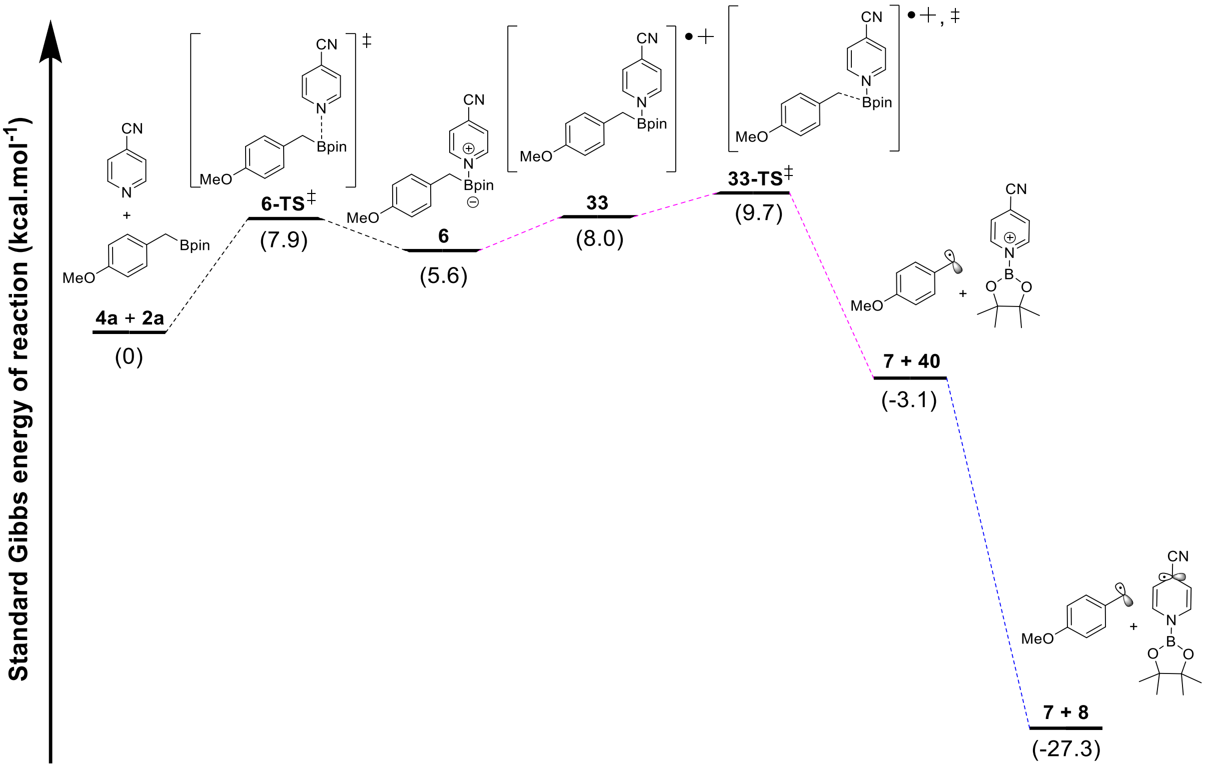

Scheme S3 Energy profile of reaction between **2a** and **4a** described in the proposed mechanism (**Figure 4**).

From this diagram it can be seen that the single electron oxidation (**6** to **7** + **40**) is the rate determining step of the reaction (**33-TS** being the highest energy point).

4 Optimisation and control experiments

4.1 Dual catalysed coupling in flow

Optimisation protocol: A 5 mL microwave vial was charged with 4-bromobenzonitrile (46 mg, 0.25 mmol), the photoredox catalyst Ir(dF(CF$_3$)ppy$_2$)(dtbpy)]PF$_6$ (3 mg, 1.0mol%), 4,4'-di-tert-butyl-2,2'-bipyridine (3 mg, 3mol%), 4-(dimethylamino)pyridine (60 mg, 2.0 equiv.) and **2a** (75 mg, 0.30 mmol, 1.2 equiv). The vial was then transferred in a glovebox where the Ni(COD)$_2$ (3 mg, 3mol%) was added and sealed with a rubber septum. Then 2.5 mL of acetone was added to obtain a clear yellow to brownish transparent solution after sonication for 5 min. The clear solution was then pumped at 200 µL/min as a slug of reaction mixture pushed with acetone through the reactor coil (10 mL reactor coil) irradiated by 420 nm LEDs (17 W total output power) thus giving an irradiation time of 50 min. The totality of the crude mixture was collected in vial wrapped in aluminium foil, filtrated on a plug of Celite and concentrated *in vacuo*. Dimbromomethane (10 µL, exact mass measured on balance) was added to the crude mixture as internal standard to determine the NMR yield in **3a**.

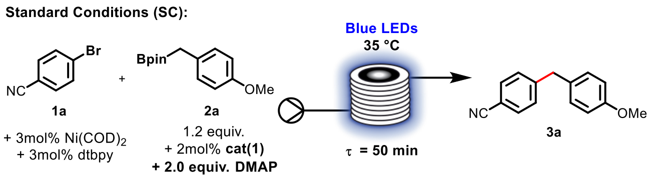

Entry	variation form SC	NMR Yield
1	none	87%
2	additive: 2,6-lutidine (3.5 equiv.)	0%
3	additive: pyridine (3.5 equiv.)	47%
4	additive: DMAP (3.5 equiv.)	82%
5	no DMAP	0%
6	Ni(acac)$_2$ instead of Ni(COD)$_2$	56%
7	no Ni(COD)$_2$	0%
8	no **cat(1)**	0%
9	no dtbpy	52%
10	no light	0%

Figure S2 – Selected optimisation and control experiments for the dual Ir/ Ni catalysed benzyl pinacol ester arylation with arylbromides in flow

Since we have had a satisfactory yield in **3a** using the boronic ester **2a** and changing the base additive from 2,6-lutidine to DMAP, we have not re-optimised all of Molander's parameters. Ni(II) could be used as a nickel source (entry 6) but resulted in a lower conversion after 50 min (rest of the mass balance being unreacted **1a**). For full optimisation of the method with trifluoroborate salts, the supplementary material from Molander can be consulted.[12]

We also investigated the possible transfer of this method in batch but using Molander's reaction setup (batch irradiation with CFL (20W instead of 26W), see supplementary material[12]) using our optimised protocol. Unfortunately, the reaction mixture turned either black or green after about 2h of irradiation and resulted in poor conversion of **1a** (< 20%). We believed that the nickel catalyst was not stable enough under these conditions and that improved kinetics in the flow system leads to full conversion of **1a** before the catalyst can deactivate.

4.2 Photoredox coupling in flow

Optimisation protocol: A 5 mL conical shape microwave vial was charged with 4-cyanopyridine **4a** (26 mg, 25mmol), the photoredox catalyst (1mol% or 4mol%) and the 4-methoxybenzyl boronic acid pinacol ester **2a** (75 mg, 0.30 mmol, 1.2 equiv). Then 1.0 mL of acetone was added to obtain a clear yellow transparent 0.25 M solution after sonication for 1 min. The clear solution was then pumped at 100 µL/min as a slug of reaction mixture pushed with acetone through the reactor coil (10mL FEP reactor coil) irradiated by 420 nm LEDs (17 W total output power) thus giving an irradiation time of 100 min. The crude mixture was collected in vial wrapped in aluminium foil and concentrated and dried *in vacuo*. Dimbromomethane (10 µL, exact mass measured on balance) was added to the crude mixture as internal standard to determine the NMR yield in **5a**.

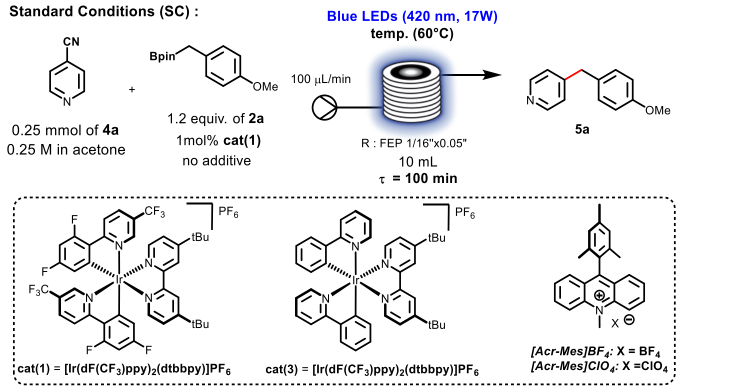

Entry	Variation from SC	NMR Yield
1	none	88%
2	catalyst: 1mol% **cat(3)**	10%
3	catalyst: 4mol% [Acr-Mes]BF_4	45%
4	catalyst: 4mol% [Acr-Mes]ClO_4	84%
5	concentration = 0.08 M; temperature = 45°C	32%
6	temperature = 35°C	68%
7	temperature = 45°C	77%
8	temperature = 80°C	88%
10	additive: TEMPO 2.0 equiv.	0%
11	no light	0%
12	no **cat(1)**	0%
13	no catalyst, 2.0 equiv. AIBN	0%

Figure S3 – Selected optimisation and control experiments for the photoredox benzyl pinacol ester arylation with cyanoarene in flow

Selection of the photocatalyst was a crucial parameter with the most oxidising photocatalysts being more potent. Interestingly the organic dye [Acr-Mes]ClO_4 proved to give **5a** in high yield but a short reaction scope revealed that it was a less general catalyst than **cat(1)**. Another key parameter was to raise the concentration from 0.08M to 0.25 M (or even 0.5 M). This is indeed helps bimolecular association of **2a** and **4a** for complex formation (See **Figure S1**). Temperature increase as well was found to lead to a more selective process. Certainly because of the improved kinetics that could either help initial complexation or the single electron oxidation process both requiring to overcome an activation barrier (see **Scheme S3**).

5 Synthesis and characterisation of starting materials

5.1 Synthesis of non-commercial benzylic boronic acid pinacol esters starting materials

The following protocol was used to synthesise the non-commercial benzylboronic esters[13]

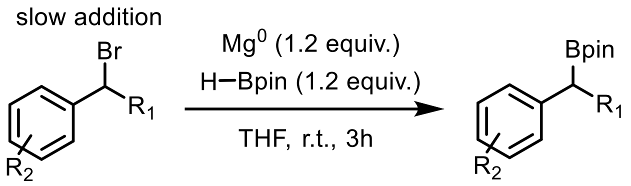

A 100 mL round-bottom flask equipped with a magnetic stir bar was charged with magnesium turnings (290 mg, 12 mmol) and was activated by addition of iodine crystals and warming until iodine sublimed. The flask was cooled to 25 °C and was purged with Ar. Dry THF (25 mL) was added to the flask, followed by the addition of neat pinacolborane (1.2 mL, 12 mmol). Benzylic bromide coumpound (10 mmol) diluted in 5 mL of dry THF was then added dropwise over 10 min with constant stirring at 25 °C. After stirring the reaction mixture at room temperature for 3h, it was cooled to 0 °C (ice bath) and acidified with 3 M aqueous HCl (15 mL) (Caution! hydrogen evolution). After 10 min of stirring the reaction mixture was warmed to 25 °C and stirred for an additional 30 min. The reaction mixture was then transferred to a separatory funnel and extracted with diethyl ether (3 × 15 mL). The combined organic layers were dried over anhydrous $MgSO_4$, filtered, and dried in vacuo (25 °C, 1 Torr). Residue was then purified over silica gel flash column chromatography (eluent: hexane/EtOAc, 20:1).

2-(3,5-dimethoxybenzyl)-4,4,5,5-tetramethyl-1,3,2-dioxaborolane (2b)

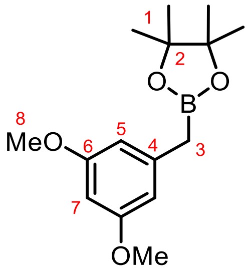

^{1}H NMR (600 MHz, CDCl$_3$) δ 6.37 (d, J = 2.1 Hz, 2H, H$_7$), 6.27 (t, J = 2.1 Hz, 1H, H$_5$), 3.78 (s, 6H, H$_8$), 2.26 (s, 2H, H$_3$), 1.26 (s, 12H, H$_1$) **^{13}C NMR (151 MHz, CDCl$_3$)** δ 160.6 (C$_6$), 140.9 (C$_4$), 107.1 (C$_5$), 97.3 (C$_7$), 83.4 (C$_2$), 55.2 (C$_8$), 24.7 (C$_1$) **^{11}B NMR (193 MHz, CDCl$_3$)** δ 33.1 **IR (ATR – neat)** $\tilde{\nu}\ (cm^{-1})$ = 2929, 2222, 1623, 1608, 1508, 1467, 1433, 1376, 1274, 1161, 1116, 898. **HRMS for [C$_{15}$H$_{24}$0$_4$^{11}B]$^+$** calcld. 279.1762 found 279.1764. ***R$_f$*** (1:9, EtOAc/Hex) = 0.19.

methyl 4-((4,4,5,5-tetramethyl-1,3,2-dioxaborolan-2-yl)methyl)benzoate (2c)

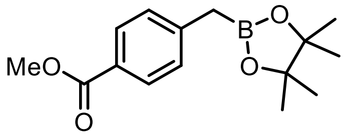

^{1}H NMR (600 MHz, CDCl$_3$) δ 7.92 (d, *J* = 8.3 Hz, 2H), 7.25 (d, *J* = 8.3 Hz, 2H), 3.89 (s, 3H), 2.36 (s, 2H), 1.23 (s, 12H). **^{13}C NMR (151 MHz, CDCl$_3$)** δ 167.3, 144.7, 129.6, 128.9, 126.9, 83.6, 51.9, 24.7. **HRMS for [$C_{15}H_{22}O_4{}^{11}B$]$^+$** calcld. 277.1611 found 277.1621. ***R_f*** (1:4, EtOAc/Hex) = 0.33. Spectroscopic data were consistent with literature values. [14]

2-(3,5-bis(trifluoromethyl)benzyl)-4,4,5,5-tetramethyl-1,3,2-dioxaborolane (2d)

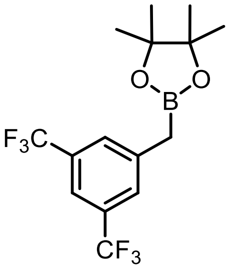

^{1}H NMR (600 MHz, CDCl$_3$) δ 7.65 (s, 1H), 7.64 (s, 2H), 2.42 (s, 2H), 1.25 (s, 12H) **^{13}C NMR (151 MHz, CDCl$_3$)** δ 141.3, 131.2 (q, *J* = 32.8 Hz), 129.1 (d, *J* = 2.5 Hz), 126.2, 124.4, 122.6, 120.8, 119.0 (dt, *J* = 7.9, 4.0 Hz), 84.0, 24.7 **^{11}B NMR (193 MHz, CDCl$_3$)** δ 32.8 **^{19}F NMR (376 MHz, CDCl$_3$)** δ -62.9 **HRMS for [$C_{15}H_{18}O_2{}^{11}B^{23}Na$]$^+$** calcld. 355.1304 found 355.1287. ***R_f***(1:9, EtOAc/Hex) = 0.40. Spectroscopic data were consistent with literature values.[15]

2-(4-fluorobenzyl)-4,4,5,5-tetramethyl-1,3,2-dioxaborolane (2f)

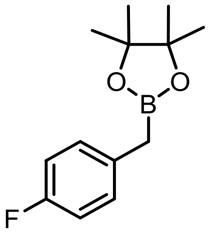

^{1}H NMR (600 MHz, CDCl$_3$) δ 7.13 (dd, *J* = 8.3, 5.6 Hz, 2H), 6.93 (t, *J* = 8.7 Hz, 2H), 2.26 (s, 2H), 1.24 (s, 12H).**^{13}C NMR (151 MHz, CDCl$_3$)** δ 161.6, 160.0, 134.1 (d, *J* = 3.1 Hz), 130.2 (d, *J* = 7.6 Hz), 115.0, 114.8, 83.5, 24.7 **^{11}B NMR (193 MHz, CDCl$_3$)** δ 33.1 **^{19}F NMR (376 MHz, CDCl$_3$)** δ -62.90 **HRMS for [$C_{13}H_{19}O_2F^{11}B$]$^+$** calcld. 237.1457 found 237.1456. ***R_f***(1:9, EtOAc/Hex) = 0.33. Spectroscopic data were consistent with literature values [16]

2-(1-(4-methoxyphenyl)ethyl)-4,4,5,5-tetramethyl-1,3,2-dioxaborolane (2h):

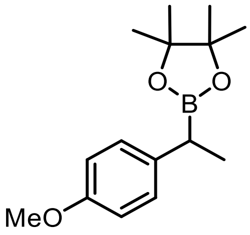

^{1}H NMR (600 MHz, $CDCl_3$) δ 7.15 (d, *J* = 8.6 Hz, 2H), 6.83 (d, *J* = 8.7 Hz, 2H), 3.79 (s, 3H), 2.39 (q, *J* = 7.5 Hz, 1H), 1.31 (d, *J* = 7.5 Hz, 3H), 1.23 (s, 6H), 1.22 (s, 6H) **^{13}C NMR (151 MHz, $CDCl_3$)** δ 157.2, 137.0, 128.6, 113.8, 83.2, 24.6, 17.37 **HRMS for $[C_{15}H_{23}O_3{}^{11}B^{23}Na]^+$** calcld. 285.1632 found 285.1621. Spectroscopic data were consistent with literature values. [16]

Synthesis of 4,4,5,5-tetramethyl-2-(1-phenylethyl)-1,3,2-dioxaborolane (2i)

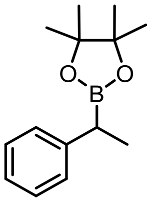

^{1}H NMR (600 MHz, $CDCl_3$) δ 7.27 (t, *J* = 7.5 Hz, 7H), 7.23 (d, *J* = 7.2 Hz, 6H), 7.14 (t, *J* = 7.2 Hz, 3H), 2.44 (q, *J* = 7.4 Hz, 3H), 1.34 (d, *J* = 7.5 Hz, 9H), 1.22 (s, 18H), 1.21 (s, 18H) **^{13}C NMR (151 MHz, $CDCl_3$)** δ 145.0, 128.3, 127.8, 125.0, 83.3, 24.6, 24.5, 17.0 **HRMS for $[C_{14}H_{21}O_2{}^{11}B^{23}Na]^+$** calcld. 255.1527 found 255.1515. $\boldsymbol{R_f}$ (1:9, EtOAc/Hex) = 0.62. Spectroscopic data were consistent with literature values.[13]

2-benzhydryl-4,4,5,5-tetramethyl-1,3,2-dioxaborolane (2j)

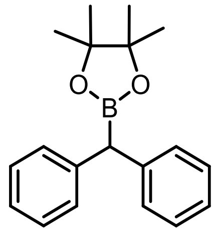

^{1}H NMR (600 MHz, $CDCl_3$) δ 7.30 (br. s, 8H), 7.20 (br. s, 2H), 3.91 (s, 1H), 1.27 (s, 12H) **^{13}C NMR (151 MHz, $CDCl_3$)** δ 142.1, 129.1, 128.4, 125.6, 83.7, 24.6 **^{11}B NMR (193 MHz, $CDCl_3$)** δ 33.1 **HRMS for $[C_{19}H_{23}0_2{}^{11}B^{23}Na]^+$** calcld. 317.1683 found 317.1671. $\boldsymbol{R_f}$ (1:9, EtOAc/Hex) = 0.39. Spectroscopic data were consistent with literature values.[17]

2-(2,6-difluorobenzyl)-4,4,5,5-tetramethyl-1,3,2-dioxaborolane (2k)

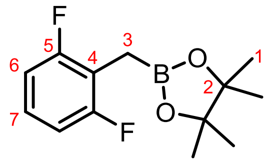

^{1}H NMR (600 MHz, CDCl$_3$) δ 7.10 – 7.04 (m, 1H, H_7), 6.83 (t, *J* = 7.5 Hz, 2H, H_6), 2.24 (s, 2H, H_3), 1.25 (s, 12H, H_1). **^{13}C NMR (151 MHz, CDCl$_3$)** δ 126.1 (C_7), 110.7 (C_6), 110.6 (C_4), 83.7 (C_2), 24.6 (C_1) **^{11}B NMR (193 MHz, CDCl$_3$)** δ 33.1 **HRMS for [$C_{13}H_{18}O_2F_2{}^{11}B$]$^+$** calcld. 255.1362 found 255.1377. ***R_f*** (1:9, EtOAc/Hex) = 0.47.

6 Synthesis and characterisation of coupling products

6.1 Dual Ir/Ni catalysed coupling of benzyl pinacol esters with aryl bromides in continuous flow.

6.1.1 General procedure I: GP(I)

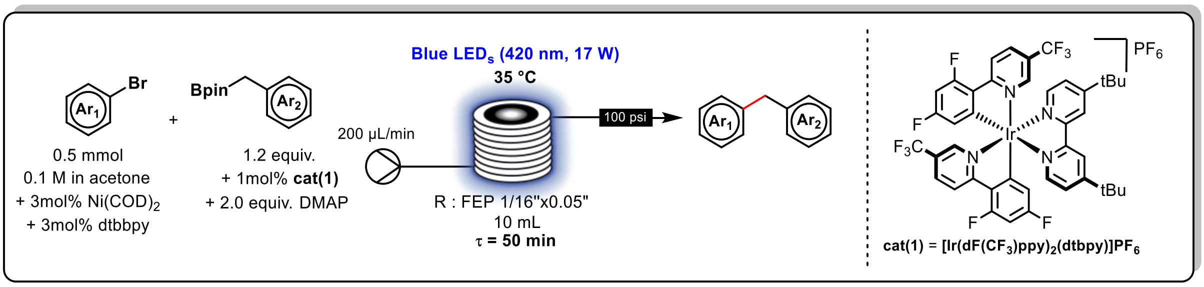

A 10 mL microwave vial was charged with the aryl bromide (0.50 mmol), the photoredox catalyst Ir(dF(CF_3)ppy$_2$)(dtbpy)]PF_6 (6.0 mg, 1.0mol%), 4,4'-di-tert-butyl-2,2'-bipyridine (6.1 mg, 3mol%), 4-(dimethylamino)pyridine (120 mg, 2.0 equiv.) and the benzyl pinacol boronic ester (0.60 mmol, 1.2 equiv). The vial was then transferred in a glovebox where the Ni(COD)$_2$ (6.0 mg, 3mol%) was added and sealed with a rubber septum. Then 5.0 mL of acetone was added to obtain a clear yellow to brownish transparent solution after sonication for 1 to 5 min. The clear solution was then pumped at 200 μL/min as a slug of reaction mixture pushed with acetone through the reactor coil (10 mL reactor coil) irradiated by 420 nm LEDs (17 W total output power) thus giving an irradiation time of 50 min. The totality of the crude mixture was collected in vial wraped in aluminium foil, filtrated on a plug of Celite and concentrated *in vacuo*. The residue was then immobilised on Isolute HM-N for easy dry loading and purified by Biotage flash column chromatography on a regular silica gel cartridge (from Silicycle) (EtOAc/Hexane, 0:100 to 5:95 gradient) to yield the pure product.

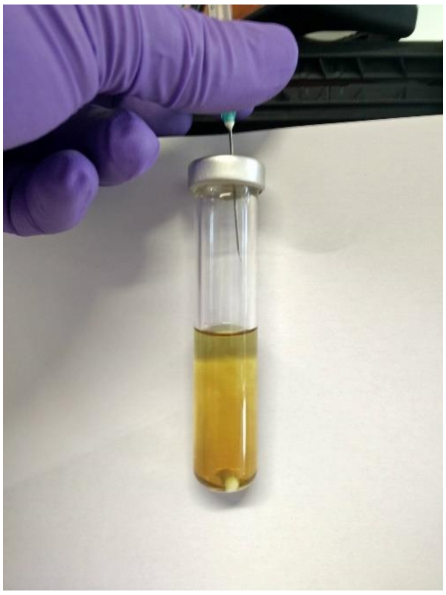

Figure S4 – Representative reaction mixture aspect for **GP(I)**

6.1.2 *Characterisation of coupling products*

Synthesis of 4-(4-methoxybenzyl)benzonitrile (3a):

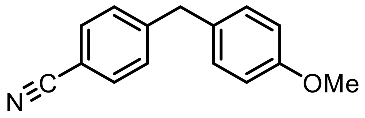

3a was obtained following ***GP(I)*** as a colourless oil (91 mg, 82% yield, $\boldsymbol{R_f}$ (1:4, EtOAc/Hex) = 0.29). **^{1}H NMR (600 MHz, CDCl$_3$)** δ 7.57 (2H, d, *J* = 8.2 Hz), 7.28 (2H, d, *J* = 8.3 Hz), 7.08 (2H, d, *J* = 8.5 Hz), 6.86 (2H, d, *J* = 8.6 Hz), 3.98 (2H, s), 3.80 (3H, s). **^{13}C NMR (151 MHz, CDCl$_3$)** δ 158.4, 147.2 , 132.3, 131.4, 129.9, 129.5, 119.0, 114.2, 109.9, 55.3, 41.1. **HRMS for [$C_{15}H_{14}NO$]$^+$** calcd. 224.1075; found 224.1077. Spectroscopic data were consistent with literature values. [18]

Synthesis of 4-(3,5-dimethoxybenzyl)benzonitrile (3b)

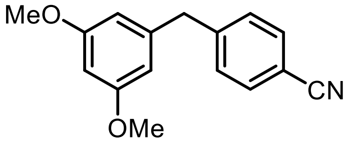

3b was obtained following ***GP(I)*** as a colourless oil (95 mg, 75% yield, $\boldsymbol{R_f}$ (1:4, EtOAc/Hex) = 0.23). **^{1}H NMR (600 MHz, CDCl$_3$)** δ 7.58 (d, *J* = 8.3 Hz, 2H), 7.30 (d, *J* = 8.3 Hz, 2H), 6.35 (t, *J* = 2.2 Hz, 1H), 6.31 (d, *J* = 2.2 Hz, 2H), 3.96 (s, 2H), 3.77 (s, 6H). **^{13}C NMR (151 MHz, CDCl$_3$)** δ 161.1, 146.4, 141.5, 132.3, 129.6, 119.0, 110.1, 107.2, 98.3, 55.3, 42.1. **HRMS for [$C_{16}H_{16}NO_2$]$^+$** calcld. 254.1181 found 254.1192. Spectroscopic data were consistent with literature values. [18]

Synthesis of methyl 4-(4-cyanobenzyl)benzoate (3c)

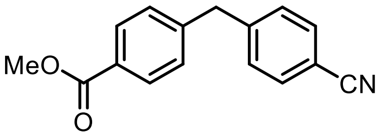

3c was obtained following ***GP(I)*** as a colourless oil (95 mg, 71% yield, $\boldsymbol{R_f}$ (1:4, EtOAc/Hex) = 0.19). **^{1}H NMR (600 MHz, CDCl$_3$)** δ 7.99 (d, *J* = 8.3 Hz, 2H), 7.59 (d, *J* = 8.2 Hz, 2H), 7.28 (d, *J* = 8.3 Hz, 2H), 7.24 (d, *J* = 8.2 Hz, 2H), 4.09 (s, 2H), 3.91 (s, 3H).**^{13}C NMR (151 MHz, CDCl$_3$)** δ 166.8, 145.6, 144.5, 132.4, 130.1, 129.7, 129.0, 128.7, 118.8, 110.4, 52.1, 41.9. **HRMS for [$C_{16}H_{14}NO_2$]$^+$**.calcld. 252.1025 found 252.1029. Spectroscopic data were consistent with literature values. [18]

Synthesis of 4-(3,5-bis(trifluoromethyl)benzyl)benzonitrile (3d)

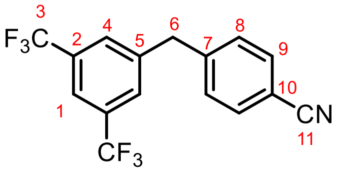

3d was obtained following ***GP(I)*** as a colourless oil (100 mg, 61% yield, $\boldsymbol{R_f}$ (1:4, EtOAc/Hex) = 0.17). **^{1}H NMR (600 MHz, CDCl$_3$)** δ 7.78 (s, 1H, H_1), 7.65 (d, $^{3}J_{HH}$ = 8.2 Hz, 2H, H_9), 7.62 (s, 2H, H_4), 7.30 (d, $^{3}J_{HH}$ = 8.2 Hz, 2H, H_8), 4.18 (s, 2H, H_6).**^{13}C NMR (151 MHz, CDCl$_3$)** δ 144.1 (C_7), 141.7 (C_5), 132.6 (C_9), 132.1 (q, $^{1}J_{CF}$ = 33.4 Hz, C_3), 129.6 (C_4), 129.0 (C_2), 124.0 (C_8), 120.9 (C_1), 118.5 (C_{11}), 111.1 (C_{10}), 41.4 (C_6). **IR (ATR – neat)** $\tilde{\nu}$ (cm^{-1}) = 2929, 2222, 1624, 1607, 1508, 1467, 1433, 1376, 1274, 1161, 1116, 898. **HRMS for [$C_{16}H_{10}NF_6$]$^+$** calcld. 330.0717 found 330.0730.

Synthesis of 4-(4-methylbenzyl)benzonitrile (3e):

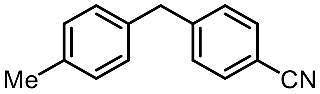

3e was obtained following ***GP(I)*** as colourless needles (80 mg, 77% yield, $\boldsymbol{R_f}$ (1:4, EtOAc/Hex) = 0.45). **^{1}H NMR (600 MHz, CDCl$_3$)** δ 7.57 (d, *J* = 8.3 Hz, 2H), 7.29 (d, *J* = 8.4 Hz, 2H), 7.13 (d, *J* = 7.8 Hz, 2H), 7.06 (d, *J* = 7.9 Hz, 2H), 4.00 (s, 2H), 2.34 (s, 3H). **^{13}C NMR (151 MHz, CDCl$_3$)** δ 147.1, 136.3, 132.2, 129.5, 128.8, 119.0, 109.9, 41.6, 21.0. **M.p.** 60 °C **HRMS for [$C_{15}H_{14}N$]$^+$** calcld. 208.1126 found 208.1133. Spectroscopic data were consistent with literature values. [19]

Synthesis of 4-(4-fluorobenzyl)benzonitrile (3f)

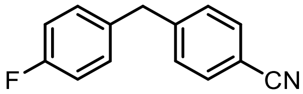

3f was obtained following ***GP(I)*** as a colourless oil (56 mg, 53% yield, $\boldsymbol{R_f}$ (1:4, EtOAc/Hex) = 0.42). **^{1}H NMR (600 MHz, CDCl$_3$)** δ 7.59 (d, *J* = 8.3 Hz, 2H), 7.27 (d, *J* = 8.3 Hz, 2H), 7.12 (dd, *J* = 8.6, 5.4 Hz, 2H), 7.01 (t, *J* = 8.7 Hz, 2H), 4.01 (s, 3H). **^{13}C NMR (151 MHz, CDCl$_3$)** δ 162.5, 160.9, 146.5, 135.0 (d, J = 3.3 Hz), 132.4, 130.4 (d, J = 7.9 Hz), 129.5, 118.9, 115.7, 115.5, 110.2, 41.1. **HRMS for [$C_{14}H_{11}NF$]$^+$** calcld. 212.0876 found 212.0883. Spectroscopic data were consistent with literature values. [20]

Sythesis of 1-methoxy-4-(4-(trifluoromethyl)benzyl)benzene (3g)

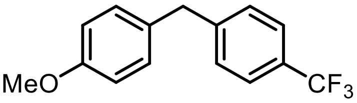

3g was obtained following ***GP(I)*** as a colourless oil (95 mg, 71% yield, $\boldsymbol{R_f}$ (1:4, EtOAc/Hex) = 0.46). **^{1}H NMR (600 MHz, CDCl$_3$)** δ 7.55 (d, *J* = 8.1 Hz, 2H), 7.30 (d, *J* = 8.0 Hz, 2H), 7.11 (d, *J* = 8.7 Hz, 2H), 6.87 (d, *J* = 8.7 Hz, 2H), 3.99 (s, 2H), 3.81 (s, 3H). **^{13}C NMR (151 MHz, CDCl$_3$)** δ 158.2, 145.7, 132.1, 129.9, 129.1, 125.4, 114.1, 55.6, 40.8. **HRMS for [$C_{15}H_{14}OF_3$]$^+$** calcld. 267.0997 found 267.0989. Spectroscopic data were consistent with literature values. [21]

Synthesis of 4-(4-methoxybenzyl)benzaldehyde (3h)

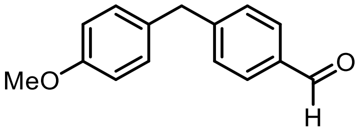

3h was obtained following ***GP(I)*** as a colourless oil (96 mg, 85% yield, $\boldsymbol{R_f}$ (1:4, EtOAc/Hex) = 0.26). **^{1}H NMR (600 MHz, $CDCl_3$)** δ 7.55 (d, J = 8.1 Hz, 2H), 7.30 (d, J = 8.0 Hz, 2H), 7.11 (d, J = 8.7 Hz, 2H), 6.87 (d, J = 8.7 Hz, 2H), 3.99 (s, 2H), 3.81 (s, 3H). **^{13}C NMR (151 MHz, $CDCl_3$)** δ 192.0, 158.3, 149.0, 134.6, 131.8, 130.4, 130.0, 129.9, 129.4, 128.9, 114.1, 55.3, 41.2. **HRMS for $[C_{15}H_{15}O_2]^+$** calcld. 227.1072 found 227.1071. Spectroscopic data were consistent with literature values. [22]

Synthesis of 1-(4-methoxybenzyl)-2-methylbenzene (3i)

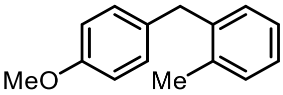

3i was obtained following ***GP(I)*** as a colourless oil (53 mg, 50% yield, $\boldsymbol{R_f}$ (1:4, EtOAc/Hex) = 0.54). **^{1}H NMR (600 MHz, $CDCl_3$)** δ 7.20 – 7.13 (m, 3H), 7.11 – 7.08 (m, 1H), 7.05 (d, J = 8.7 Hz, 2H), 6.83 (d, J = 8.7 Hz, 2H), 3.94 (s, 2H), 3.79 (s, 3H), 2.26 (s, 3H). **^{13}C NMR (151 MHz, $CDCl_3$)** δ 157.8, 139.3, 136.5, 132.4, 130.2, 129.8, 129.7, 126.3, 125.9, 113.8, 55.2, 38.5, 19.6. **HRMS for $[C_{15}H_{16}O]^+$** calcld. 212.1201 found 212.1202. Spectroscopic data were consistent with literature values.[23]

Synthesis of 1-methoxy-4-(4-vinylbenzyl)benzene (3j)

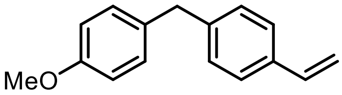

3j was obtained following ***GP(I)*** as a colourless oil (81 mg, 72% yield, $\boldsymbol{R_f}$ (1:4, EtOAc/Hex) = 0.51). **^{1}H NMR (600 MHz, $CDCl_3$)** δ 7.36 (d, J = 8.1 Hz, 2H), 7.14 (dd, J = 21.3, 8.4 Hz, 4H), 6.86 (d, J = 8.7 Hz, 2H), 6.71 (dd, J = 17.6, 10.9 Hz, 1H), 5.73 (dd, J = 17.6, 0.7 Hz, 1H), 5.22 (dd, J = 10.9, 0.6 Hz, 1H), 3.94 (s, 2H), 3.80 (s, 3H).**^{13}C NMR (151 MHz, $CDCl_3$)** δ 157.9, 141.3, 136.6, 135.4, 133.1, 129.9, 129.0, 126.3, 113.9, 113.2, 55.3, 40.8. **HRMS for $[C_{16}H_{17}O]^+$** calcld. 225.1279 found 225.1274. Spectroscopic data were consistent with literature values.[24]

Synthesis of ((4-(4-methoxybenzyl)phenyl)ethynyl)trimethylsilane (3k)

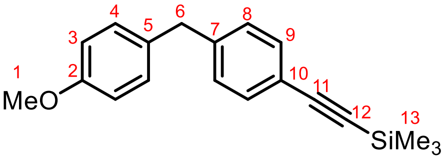

3k was obtained following ***GP(I)*** as a colourless oil (91 mg, 75% yield, $\boldsymbol{R_f}$ (1:4, EtOAc/Hex) = 0.54). **^{1}H NMR (600 MHz, $CDCl_3$)** δ 7.39 (d, $^3J_{HH}$ = 8.2 Hz, 2H, H_9), 7.11 (d, $^3J_{HH}$ = 8.2 Hz, 2H, H_8), 7.07 (d, $^3J_{HH}$ = 8.7 Hz, 2H, H_4), 6.84 (d, $^3J_{HH}$ = 8.7 Hz, 2H, H_3), 3.92 (s, 2H, H_6), 3.79 (s, 3H, H_1), 0.25 (s, 9H, H_{13}). **^{13}C NMR (151 MHz, $CDCl_3$)** δ 158.1 (C_2), 142.2 (C_7), 132.6 (C_5), 132.1 (C_9), 129.8 (C_4), 128.7 (C_8), 120.7 (C_{10}), 113.9 (C_3), 105.2 (C_{11}), 93.6 (C_{12}), 55.2

(C_1), 40.9 (C_6), 0.0 (C_{13}). **IR (ATR – neat)** $\tilde{\nu}$ (cm^{-1}) = 2956, 2901, 2155, 1609, 1584, 1510, 1504, 1455, 1441, 1300, 1243, 1175, 1031, 840, 813, 755. **HRMS for [$C_{19}H_{22}OSi$]$^+$** calcld. 294.1440 found 294.1442.

Synthesis of 4-(4-methoxybenzyl)phenol (3l)

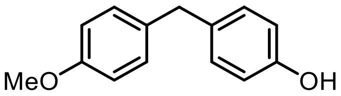

3l was obtained following ***GP(I)*** using 2-(4-bromophenyl)-4,4,5,5-tetramethyl-1,3,2-dioxaborolane as arylbromide followed by an NaOH/H_2O_2 oxidation of the arylpinacolborolane product as described by Molander.[25] The corresponding phenol was then isolated as a white solid (76 mg, 71% yield, R_f (1:4, EtOAc/Hex) = 0.13). **^{1}H NMR (600 MHz, $CDCl_3$)** δ 7.12 (d, *J* = 8.7 Hz, 2H), 7.06 (d, *J* = 8.5 Hz, 2H), 6.86 (d, *J* = 8.7 Hz, 2H), 6.77 (d, *J* = 8.5 Hz, 2H), 5.31 (s, 1H), 3.88 (s, 2H), 3.81 (s, 3H).**^{13}C NMR (151 MHz, $CDCl_3$)** δ 157.8, 153.7, 133.8, 133.8, 130.0, 129.8, 115.3, 113.9, 55.3, 40.1 **HRMS for [$C_{14}H_{14}O_2$]$^+$** calcld. 214.0994 found 214.0986. Spectroscopic data were consistent with literature values.[26]

6.2 *Flow photoredox coupling of pinacol esters with cyanoarenes*

6.2.1 *General procedure II: GP(II)*

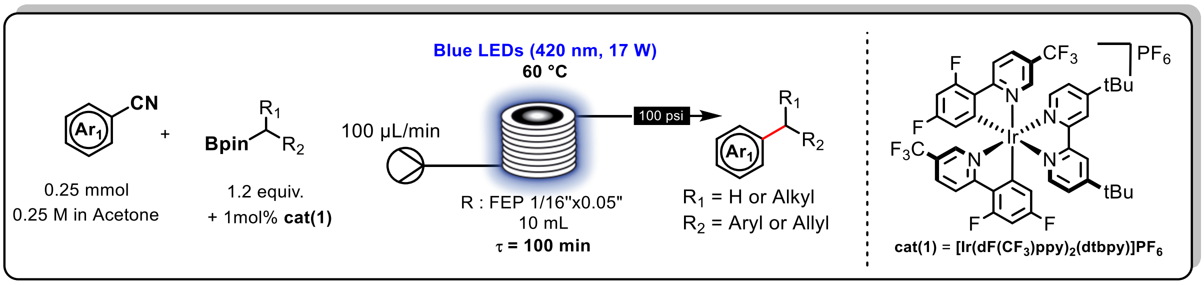

A 5 mL conical shape microwave vial was charged with the cyanoarene (0.25mmol), the photoredox catalyst Ir(dF(CF_3)ppy_2)(dtbpy)]PF_6 (3.0 mg, 1.0mol%) and the benzyl pinacol boronic ester (0.30 mmol, 1.2 equiv). Then 1.0 mL of acetone was added to obtain a clear yellow transparent 0.25 M solution after sonication for 1 min. The clear solution was then pumped at 100 µL/min as a slug of reaction mixture pushed with acetone through the reactor coil (10 mL FEP reactor coil) irradiated by 420 nm LEDs (17 W total output power) thus giving an irradiation time of 100 min. The crude mixture was collected in vial wrapped in aluminium foil and concentrated *in vacuo*. The residue was then purified by Bioatage flash column chromatography on KP-NH modified silica gel cartridge (EtOAc/Hexane, 0% to 20% gradient) to yield the pure product.

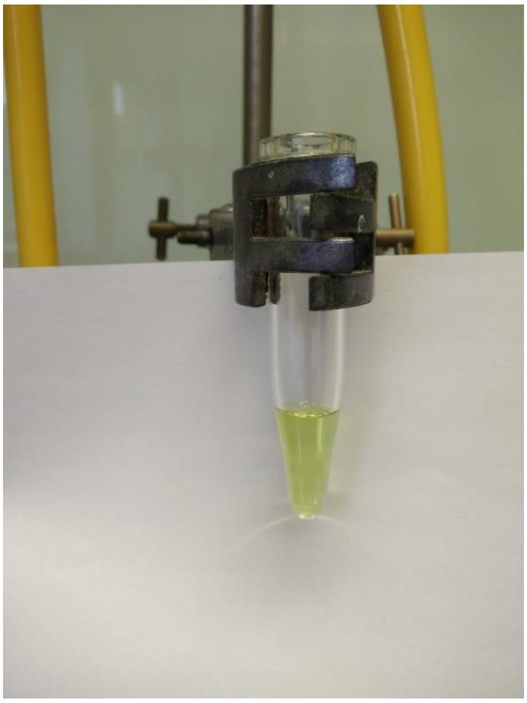

Figure S5 – Representative reaction mixture aspect for **GP(II)**

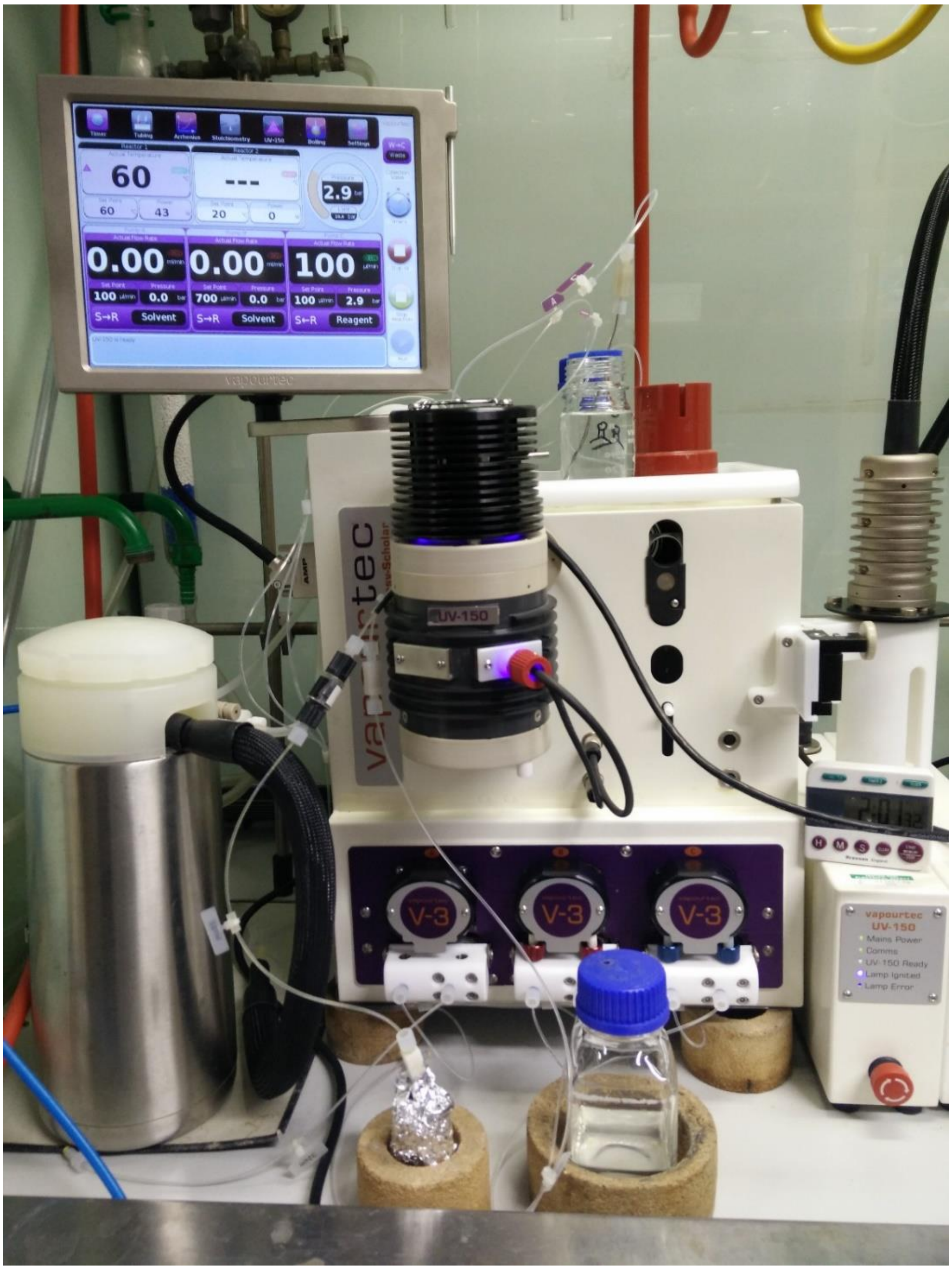

Figure S6 – Flow reaction setup for **GP(II)**

6.2.2 Characterisation of coupling products

Synthesis of 4-(4-methoxybenzyl)pyridine (5a)

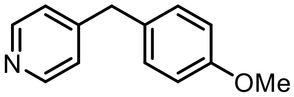

5a was obtained following ***GP(II)*** as a pale yellow oil (40 mg,81% yield, $\boldsymbol{R_f}$ (EtOAc/Hex, 1:1) = 0.13). **^{1}H NMR (600 MHz, CDCl$_3$)** δ 8.49 (d, *J* = 5.8 Hz, 2H), 7.13 – 7.07 (m, 4H), 6.86 (d, *J* = 8.7 Hz, 2H), 3.91 (s, 2H), 3.80 (s, 3H). **^{13}C NMR (151 MHz, CDCl$_3$)** δ 158.4, 150.6, 149.7, 130.9, 130.0, 124.1, 114.1, 55.3, 40.3. **HRMS for [C$_{13}$H$_{14}$NO]$^+$** calcd. 200.1075; found 200.1078. Spectroscopic data were consistent with literature values.[18]

Synthesis of 4-(4-methylbenzyl)pyridine (5b)

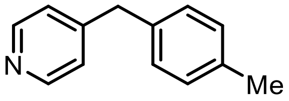

5b was obtained following ***GP(II)*** as a colourless oil (32 mg, 70% yield, $\boldsymbol{R_f}$ (EtOAc/Hex, 1:1) = 0.13). **^{1}H NMR (600 MHz, $CDCl_3$)** δ 8.49 (d, J = 5.3 Hz, 2H), 7.13 (d, J = 7.8 Hz, 2H), 7.10 (d, J = 5.5 Hz, 2H), 7.07 (d, J = 7.9 Hz, 2H), 3.93 (s, 2H), 2.34 (s, 3H).**^{13}C NMR (151 MHz, $CDCl_3$)** δ 150.3, 149.8, 136.3, 135.8, 129.4, 128.9, 124.1, 40.8, 21.0. **HRMS for $[C_{13}H_{14}N]^+$** calcld. 184.1121 found 184.117. Spectroscopic data were consistent with literature values.[27]

Synthesis of 4-(benzyl)pyridine (5c)

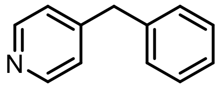

5c was obtained following ***GP(II)*** as a colourless oil (28 mg, 66% yield, $\boldsymbol{R_f}$ (EtOAc/Hex, 1:1) = 0.13). **^{1}H NMR (600 MHz, $CDCl_3$)** δ 8.53 (br.s, 2H), 7.33 (t, *J* = 7.5 Hz, 2H), 7.26 (t, *J* = 7.4 Hz, 2H), 7.18 (d, *J* = 7.4 Hz, 2H), 7.15 (d, *J* = 4.4 Hz, 2H), 3.99 (s, 2H). **^{13}C NMR (151 MHz, $CDCl_3$):** δ 150.6, 149.3, 138.7, 129.0, 128.8, 126.7, 124.4, 41.3. **HRMS for $[C_{12}H_{12}N]^+$** calcd. 170.0970; found 170.0977. Spectroscopic data were consistent with literature values.[28]

Synthesis of 4-(1-(4-methoxyphenyl)ethyl)pyridine (5d)

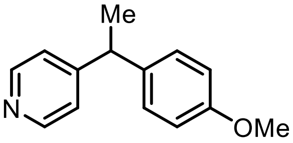

5d was obtained following ***GP(II)*** as a colourless oil (44 mg, 83% yield, $\boldsymbol{R_f}$ (EtOAc/Hex, 1:1) = 0.12). **^{1}H NMR (600 MHz, $CDCl_3$)** δ 8.49 (d, *J* = 5.9 Hz, 2H), 7.16 – 7.05 (m, 4H), 6.85 (d, *J* = 8.6 Hz, 2H), 4.07 (q, *J* = 7.2 Hz, 1H), 3.79 (s, 3H), 1.61 (d, *J* = 7.2 Hz, 3H). **^{13}C NMR (151 MHz, $CDCl_3$)** δ 158.3, 155.5, 149.8, 136.5, 128.5, 122.9, 114.0, 55.2, 43.4, 21.2. **HRMS for $[C_{12}H_{12}N]^+$** calcd. 214.1226; found 214.1220. Spectroscopic data were consistent with literature values. [26]

Synthesis of 1-(4-methoxybenzyl)isoquinoline (5e)

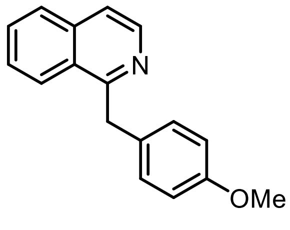

5e was obtained following ***GP(II)*** as a pale green oil (47 mg,75% yield, $\boldsymbol{R_f}$ (EtOAc/Hex, 1:1) = 0.48) **^{1}H NMR (600 MHz, $CDCl_3$)** δ 8.50 (d, *J* = 5.7 Hz, 1H), 8.17 (d, *J* = 8.5 Hz, 1H), 7.81 (d, *J* = 8.2 Hz, 1H), 7.64 (ddd, *J* = 8.1, 7.0, 1.0 Hz, 1H), 7.56 (d, *J* = 5.8 Hz, 1H), 7.53 (ddd, *J* = 8.2, 6.9, 1.1 Hz, 2H), 7.21 (d, *J* = 8.7 Hz, 2H), 6.80 (d, *J* = 8.7 Hz, 2H), 4.62 (s, 2H), 3.75 (s, 3H). **^{13}C NMR (151 MHz, $CDCl_3$)** δ 160.5, 158.0, 142.0, 136.6, 131.6, 129.8, 129.5, 127.3,

127.1,127.0, 125.8, 119.7, 113.9, 55.2, 41.2. **HRMS for [$C_{17}H_{16}ON]^+$** calcld. 250.1226 found 250.1216. Spectroscopic data were consistent with literature values.[29]

Synthesis of 1-(1-phenylethyl)isoquinoline (5f)

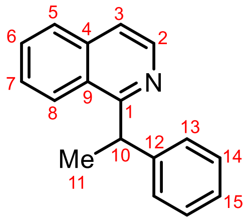

5f was obtained following ***GP(II)*** as a white solid (44 mg, 75% yield, **R_f** (EtOAc/Hex, 1:1) = 0.32).**^{1}H NMR (600 MHz, $CDCl_3$)** δ 8.59 (d, J = 5.7 Hz, 1H, H_2), 8.19 (d, J = 8.6 Hz, 1H, H_8), 7.79 (d, J = 8.2 Hz, 1H, H_5), 7.60 (t, J = 7.5 Hz, 1H, H_6), 7.55 (d, J = 5.7 Hz, 1H, H_7), 7.49 (t, J = 7.8 Hz, 1H, H_3), 7.33 (d, J = 7.5 Hz, 2H, H_{13}), 7.26 (t, J = 7.6 Hz, 2H, H_{14}), 7.16 (t, J = 7.3 Hz, 1H, H_{15}), 5.10 (q, J = 7.0 Hz, 1H, H_{10}), 1.87 (d, J = 7.0 Hz, 3H, H_{11}). **^{13}C NMR (151 MHz, $CDCl_3$)** δ 162.9 (C_1), 145.9 (C_2), 141.7 (C_{12}), 136.5 (C_4), 129.5 (C_6), 128.5 (C_{14}), 127.6 (C_{13}), 127.4 (C_8), 127.0 (C_7), 126.2 (C_{15}), 125.3 (C_5), 120.0 (C_3), 43.2 (C_{10}), 21.9 (C_{11}). **IR (ATR – neat)** $\tilde{\nu}$ (cm^{-1}) = 3060, 3020, 2968, 2925, 1619, 1584, 1560, 1491, 1354, 1140, 876. **M.p** 62 °C **HRMS for [$C_{17}H_{16}N]^+$** calcld. 234.1277 found 234.1266.

Synthesis of 1-(4-methylbenzyl)isoquinoline (5g)

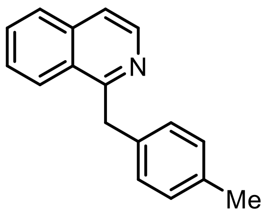

5g was obtained following ***GP(II)*** as a colourless oil (43 mg, 70% yield, **R_f** (EtOAc/Hex, 1:1) = 0.34). **^{1}H NMR (600 MHz, $CDCl_3$)** δ 8.51 (d, J = 5.7 Hz, 1H), 8.17 (d, J = 8.5 Hz, 1H), 7.81 (d, J = 8.2 Hz, 1H), 7.63 (t, J = 7.5 Hz, 1H), 7.56 (d, J = 5.7 Hz, 1H), 7.53 (t, J = 7.6 Hz, 1H), 7.19 (d, J = 7.9 Hz, 2H), 7.07 (d, J = 7.9 Hz, 2H), 4.65 (s, 2H), 2.29 (s, 3H). **^{13}C NMR (151 MHz, $CDCl_3$)** δ 160.4, 142.0, 136.6, 136.4, 135.7, 129.8, 129.2, 128.5, 127.3, 127.2, 125.9, 119.7, 41.7, 21.0. **HRMS for [C17H16N]$^+$** calcld. 234.1277 found 234.1265 Spectroscopic data were consistent with literature values.[30]

Synthesis of 1-benzhydrylisoquinoline (5h)

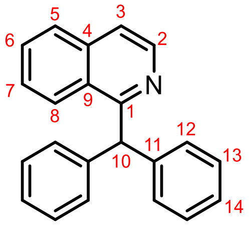

5h was obtained following ***GP(II)*** as a white solid (62 mg, 84% yield, **R_f** (EtOAc/Hex, 1:9) = 0.16). **^{1}H NMR (600 MHz, $CDCl_3$)** δ 8.56 (d, J = 5.7 Hz, 1H, H_2), 8.27 (d, J = 8.6 Hz, 1H, H_8),

7.85 (d, J = 8.2 Hz, 1H, H_5), 7.66 (t, J = 7.5 Hz, 1H, H_6), 7.57 – 7.54 (m, 2H, H_7 and H_3), 7.34 – 7.31 (m, 4H, H_{13}), 7.29 – 7.23 (m, 6H, H_{12} and H_{14}), 6.52 (s, 1H, H_{10}). **^{13}C NMR (151 MHz, $CDCl_3$)** δ 161.9 (C_1), 142.8 (C_{11}), 142.0 (C_2), 136.6 (C_4), 129.6 (C_6), 128.3 (C_{13}), 127.5 (C_{12}), 127.3 (C_8 and C_9), 126.5 (C_7), 125.2 (C_{14}), 119.6 (C_5), 54.8 (C_{10}). **IR (ATR – neat)** $\tilde{\nu}\ (cm^{-1})$ = 3056, 3020, 1621, 1586, 1560, 1495, 1447, 1380, 1344, 1156, 1029, 826. **M.p** 91 °C **HRMS for $[C_{22}H_{18}N]^+$** calcld. 296.1434 found 296.1421.

Synthesis of 1-(4-fluorobenzyl)isoquinoline (5i)

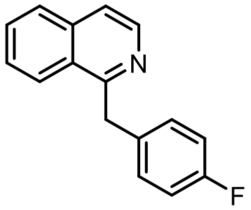

5i was obtained following ***GP(II)*** as a white solid (39 mg, 65% yield, **R_f** (EtOAc/Hex, 1:1) = 0.48). **^{1}H NMR (600 MHz, $CDCl_3$)** δ 8.50 (d, J = 5.6 Hz, 1H), 8.13 (d, J = 8.4 Hz, 1H), 7.83 (d, J = 8.1 Hz, 1H), 7.65 (t, J = 7.5 Hz, 1H), 7.58 (d, J = 5.7 Hz, 1H), 7.55 (t, J = 7.6 Hz, 1H), 7.26 – 7.22 (m, 2H), 6.95 (t, J = 8.5 Hz, 2H), 4.65 (s, 2H). **^{13}C NMR (151 MHz, $CDCl_3$)** δ 162.3, 160.7, 159.9, 142.0, 136.6, 135.0 (d, J = 3.2 Hz), 130.0, 129.9 (d, J = 4.6 Hz), 127.4, 127.3, 127.1, 125.6, 119.9, 115.4, 115.2, 41.1. **HRMS for $[C_{16}H_{13}NF]^+$** calcld. 238.1027 found 238.1025. Spectroscopic data were consistent with literature values.[31]

Synthesis of 1-(3,5-bis(trifluoromethyl)benzyl)isoquinoline (5j)

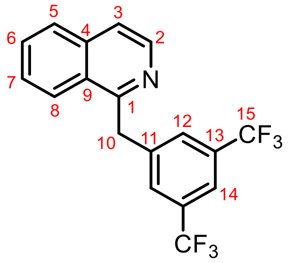

5j was obtained following ***GP(II)*** as a white solid (48 mg, 55% yield, **R_f** (EtOAc/Hex, 1:1) = 0.67). **^{1}H NMR (600 MHz, $CDCl_3$)** δ 8.52 (d, J = 5.7 Hz, 1H, H_2), 8.10 (d, J = 8.5 Hz, 1H, H_8), 7.87 (d, J = 8.2 Hz, 1H, H_5), 7.78(s, 2H, H_{12}), 7.73 (s, 1H, H_{14}), 7.70 (t, J = 7.5 Hz, 1H, H_6), 7.63-7.60 (m, 2H, H_7 and H_3), 4.78 (s, 2H, H_{10}).**^{13}C NMR (151 MHz, $CDCl_3$)** δ 157.9 (C_1), 142.2 (C_2), 141.6 (C_{11}), 136.6 (C_4), 131.6 (q, J = 33.2 Hz, C_{15}), 130.2 (C_{12}), 129.0 (C_6), 127.7 (d, J = 4.7 Hz, C_{13}), 126.9 (C_8), 124.8 (C_9), 124.2 (C_7), 122.4 (C_5), 120.5 (C_{14}), 120.4 (C_3), 41.0 (C_{10}). **IR (ATR – neat)** $\tilde{\nu}\ (cm^{-1})$ = 3051, 2929, 1621, 1586, 1558, 1506, 1463, 1372, 1280, 1165, 1116, 880, 828. **M.p.** 82 °C. **HRMS for $[C_{18}H_{12}NF_6]^+$** calcld. 356.0868 found 356.0858

Synthesis of 1-(2,6-difluorobenzyl)isoquinoline (5k)

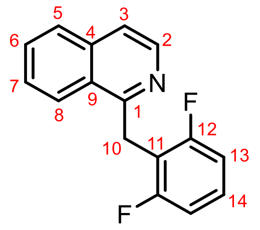

5k was obtained following ***GP(II)*** as a white solid (40 mg, 63% yield, **R*f*** (EtOAc/Hex, 1:1) = 0.67). **^{1}H NMR (600 MHz, CDCl$_3$)** δ 8.42 (d, *J* = 5.7 Hz, 1H, H_2), 8.30 (d, *J* = 8.4 Hz, 1H, H_8), 7.85 (d, *J* = 8.1 Hz, 1H, H_5), 7.73 – 7.68 (m, 1H, H_6), 7.64 (t, *J* = 7.6 Hz, 1H, H_7), 7.54 (d, *J* = 5.7 Hz, 1H, H_3), 7.27 – 7.20 (m, 1H, H_{14}), 6.94 (t, *J* = 7.8 Hz, 2H, H_{13}), 4.73 (s, 2H, H_{10}). **^{13}C NMR (151 MHz, CDCl3)** δ 161.7 (dd, *J* = 248 Hz, *J* = 8.7 Hz, C_{12}), 157.6 (C_1), 142.0 (C_2), 136.2 (C_4), 129.8 (C_6), 128.2 (t, *J* = 10.3 Hz, C_{14}), 127.4 (C_8), 127.2 (C_9), 126.9 (C_7), 124.8 (C_5), 119.6 (C_3), 114.8 (t, *J* = 19.9 Hz, C_{11}), 111.1 (dd, *J* = 20.9, 5.1 Hz, C_{13}), 28.4 (C_{10}). **^{19}F NMR (376 MHz, CDCl$_3$)** δ -113.81. **IR (ATR – neat)** $\tilde{\nu}$ (cm^{-1}) = 2924, 2853, 1623, 1590, 1566, 1465, 1380, 1205, 1019, 1003, 860, 743. **M.p.** 83 °C. **HRMS for [$C_{16}H_{12}NF_2$]$^+$** calcld. 256.0932 found 256.0927

Synthesis of 1-(3,5-dimethoxybenzyl)isoquinoline (5l)

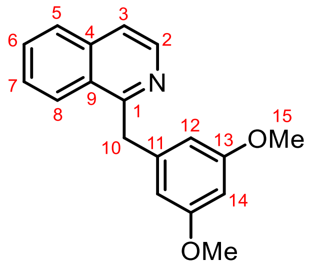

5l was obtained following ***GP(II)*** as a pale green oil (46 mg, 66% yield, **R*f*** (EtOAc/Hex, 1:1) = 0.39) **^{1}H NMR (600 MHz, CDCl$_3$)** δ 8.51 (d, *J* = 5.7 Hz, 1H, H_2), 8.17 (d, *J* = 8.5 Hz, 1H, H_8), 7.82 (d, *J* = 8.2 Hz, 1H, H_5), 7.65 (t, *J* = 7.5 Hz, 1H, H_6), 7.58 (d, *J* = 5.7 Hz, 1H, H_3), 7.54 (t, *J* = 7.6 Hz, 1H, H_7), 6.46 (s, 2H, H_{12}), 6.30 (s, 1H, H_{14}), 4.62 (s, 2H, H_{10}), 3.73 (s, 6H, H_{15}). **^{13}C NMR (151 MHz, CDCl$_3$)** δ 160.8 (C_{13}), 159.8 (C_1), 141.9 (C_2), 141.8 (C_{11}), 136.6 (C_4), 129.9 (C_6), 127.3 (C_9 and C_8), 127.2 (C_7), 125.8 (C_5), 119.9 (C_3), 106.9 (C_{12}), 98.1 (C_{14}), 55.2 (C_{15}), 42.3 (C_{10}). **IR (ATR – neat)** $\tilde{\nu}$ (cm^{-1}) = 3056, 3000, 2937, 2837, 1596, 1560, 1500, 1461, 1427, 1383, 1342, 1322, 1288, 1205, 1154, 1058, 822, 789, 747, 730, 684. **HRMS for [$C_{18}H_{18}NO_2$]$^+$** calcld. 280.1332 found 280.1328.

Synthesis of 4-(4-methoxybenzyl)-1H-pyrrolo[2,3-b]pyridine (5m)

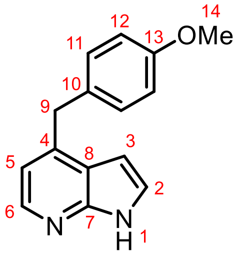

5m was obtained following ***GP(II)*** (using 3.5 mL of acetone instead of 1.0 mL because of the low starting material solubility) as a brownish solid (36 mg, 60% yield, **R*f*** (EtOAc/Hex, 1:1) = 0.19). **^{1}H NMR (600 MHz, CDCl$_3$)** δ 11.12 (s, 1H, H_1), 8.25 (d, *J* = 4.8 Hz, 1H, H_6), 7.32 (d, *J* = 3.0 Hz, 1H, H_2), 7.18 (d, *J* = 8.4 Hz, 2H, H_{11}), 6.87 – 6.84 (m, 3H, H_{12} and H_5), 6.51 (d, *J* = 3.3 Hz, 1H, H_3), 4.23 (s, 2H, H_9), 3.79 (s, 3H, H_{14}). **^{13}C NMR (151 MHz, CDCl$_3$)** δ 158.2 (C_{13}), 148.7 (C_7), 143.4 (C_6), 142.6 (C_4), 131.4 (C_{10}), 130.0 (C_{11}), 124.6 (C_2), 120.2 (C_8), 116.0 (C_5), 114.0 (C_{12}), 99.3 (C_3), 55.2 (C_{14}), 38.2 (C_9). **IR (ATR – neat)** $\tilde{\nu}$ (cm^{-1}) = 3127, 3000, 2929, 2837, 2762, 1592, 1508, 1439, 1348, 1243, 1181, 1031, 823. **M.p.** 118°C. **HRMS for [$C_{15}H_{15}ON_2$]$^+$** calcld. 239.1179 found 239.1179.

Synthesis of 4-(4-methoxybenzyl)quinoline (5n)

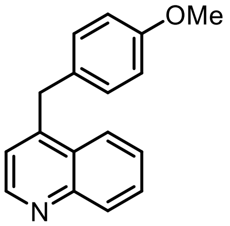

5n was obtained following ***GP(II)*** as a white solid (41 mg, 66% yield, **R*f*** (EtOAc/Hex, 1:1) = 0.85).**^{1}H NMR (600 MHz, CDCl$_3$)** δ 8.19 (d, *J* = 8.5 Hz, 1H), 8.14 (d, *J* = 8.2 Hz, 1H), 7.85 (d, *J* = 7.5 Hz, 1H), 7.72 (t, *J* = 7.5 Hz, 1H), 7.55 (s, 1H), 7.23 (d, *J* = 8.6 Hz, 2H), 6.90 (d, *J* = 8.6 Hz, 2H), 4.33 (s, 2H), 3.82 (s, 3H). **^{13}C NMR (151 MHz, CDCl$_3$)** δ 161.2, 158.7, 147.6, 131.2, 130.2, 129.9, 129.8, 128.4, 125.5, 124.72, 124.2, 119.2, 115.7, 114.4, 55.3, 44.3. **HRMS for [$C_{17}H_{16}NO$]$^+$** calcld. 250.1226 found 250.1227. **M.p.** 81 °C. Spectroscopic data were consistent with literature values.[18]

Synthesis of 4-(4-methoxybenzyl)picolinonitrile (5o)

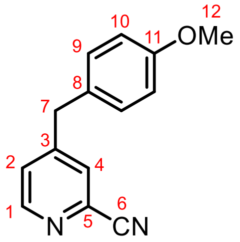

5o was obtained following ***GP(II)*** as a pale yellow oil (25 mg, 44% yield, **R*f*** (EtOAc/Hex, 1:1) = 0.54).**^{1}H NMR (600 MHz, CDCl$_3$)** δ 8.72 (d, *J* = 5.0 Hz, 1H, H_1), 7.34 (d, *J* = 5.0 Hz, 1H, H_2), 7.30 (s, 1H, H_4), 7.17 (d, *J* = 8.5 Hz, 2H, H_9), 6.88 (d, *J* = 8.6 Hz, 2H, H_{10}), 4.16 (s, 2H, H_7), 3.81 (s, 3H, H_{12}). **^{13}C NMR (151 MHz, CDCl$_3$)** δ 163.3 (C_{11}), 158.6 (C_3), 150.2 (C_1), 130.2 (C_9), 129.9 (C_5), 124.5 (C_2), 122.6 (C_8), 120.8 (C_4), 116.6 (C_6), 114.3 (C_{10}), 55.3 (C_{12}), 43.6 (C_7). **IR (ATR – neat)** $\tilde{\nu}$ (cm^{-1}) = 2956, 2933, 2238, 1609, 1594, 1508, 1461, 1395, 1302, 1243, 1177, 1033, 830, 817, 775. **HRMS for [$C_{14}H_{13}N_2O$]$^+$**calcld. 225.1022 found 225.1022.

Synthesis of 4-(4-methoxybenzyl)nicotinonitrile (5p)

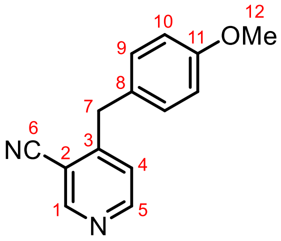

5p was obtained following ***GP(II)*** as a colourless oil (36 mg, 65% yield, **R*f*** (EtOAc/Hex, 1:1) = 0.23). **^{1}H NMR (600 MHz, CDCl$_3$)** δ 8.82 (s, 1H, H_1), 8.65 (d, *J* = 5.2 Hz, 1H, H_5), 7.17 (d, *J* = 5.2 Hz, 1H, H_4), 7.15 (d, *J* = 8.7 Hz, 2H, H_9), 6.88 (d, *J* = 8.7 Hz, 2H, H_{10}), 4.14 (s, 2H, H_7), 3.81 (s, 3H, H_{12}).**^{13}C NMR (151 MHz, CDCl$_3$)** δ 158.8 (C_{11}), 154.1 (C_3), 153.0 (C_5), 152.8 (C_1), 130.2 (C_9), 128.6 (C_8), 124.1 (C_4), 116.0 (C_6), 114.4 (C_2), 110.3 (C_{10}), 55.3 (C_{12}), 38.9 (C_7). **HRMS for [$C_{14}H_{13}ON_2$]$^+$** calcld. 225.1022 found 225.1019.

Synthesis of 2-(4-methoxybenzyl)pyridine (5q)

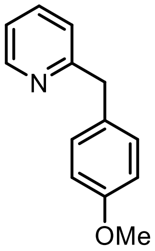

5q was obtained following ***GP(II)*** as a pale yellow oil (20 mg, 40% yield, **R*f*** (EtOAc/Hex, 1:1) = 0.36).**^{1}H NMR (600 MHz, CDCl$_3$)** δ 8.55 (d, *J* = 4.5 Hz, 1H), 7.58 (td, *J* = 7.7, 1.2 Hz, 1H), 7.19 (d, *J* = 8.4 Hz, 2H), 7.11 (t, *J* = 8.2 Hz, 2H), 6.85 (d, *J* = 8.5 Hz, 2H), 4.11 (s, 2H), 3.79 (s, 3H). **^{13}C NMR (151 MHz, CDCl$_3$)** δ 161.3, 158.2, 149.2, 136.6, 131.5, 130.1, 123.0, 121.2, 114.0, 55.2, 43.7 **HRMS for [$C_{13}H_{14}ON$]$^+$** 200.1070 found 200.1071. Spectroscopic data were consistent with literature values.[23]

Synthesis of 2-benzhydrylquinoline (5r)

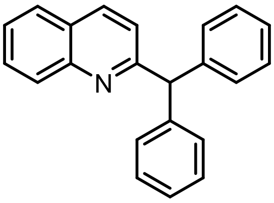

5r was obtained following ***GP(II)*** as a colourless oil (41 mg, 55% yield, **R*f*** (EtOAc/Hex, 1:1) = 0.72). **^{1}H NMR (400 MHz, CDCl$_3$)** δ 8.08 (t, *J* = 7.8 Hz, 2H), 7.79 (dd, *J* = 8.1, 1.1 Hz, 1H), 7.70 (ddd, *J* = 8.5, 6.9, 1.4 Hz, 1H), 7.51 (ddd, *J* = 8.1, 7.0, 1.1 Hz, 1H), 7.33 – 7.27 (m, 5H), 7.26 – 7.21 (m, 6H), 5.94 (s, 1H). **^{13}C NMR (151 MHz, CDCl$_3$)** δ 163.1, 147.9, 142.6, 136.4, 129.5, 129.5, 129.4, 128.4, 127.5, 126.8, 126.6, 126.3, 122.0, 60.1. **HRMS for [$C_{22}H_{18}N$]$^+$** calcld. 296.1434 found 296.1421 Spectroscopic data were consistent with literature values.[32]

6.2.3 General procedure III: GP(III):

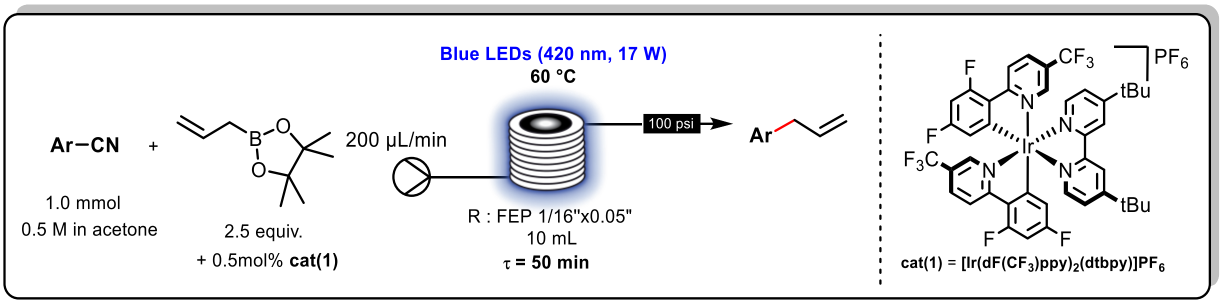

A 5 mL conical shape microwave vial was charged with the cyanoarene (1.0 mmol), the photoredox catalyst Ir(dF(CF_3)ppy_2)(dtbpy)]PF_6 (6.0 mg, 0.5mol%) and the allyl pinacol boronate (2.5 mmol, 2.5 equiv). Then 1.5 mL of acetone was added to obtain a clear yellow transparent 0.5 M solution after sonication for 1 min. The clear solution was then pumped at 200 µL/min as a slug of reaction mixture pushed with acetone through the reactor coil (10 mL reactor coil) irradiated by 420 nm LEDs (17W total output power) thus giving an irradiation time of 100 min. The crude mixture was collected in vial wrapped in aluminium foil and concentrated *in vacuo*. The residue was then purified by Bioatage flash column chromatography on KP-NH modified silica gel cartridge (EtOAc/Hexane, 0:100 to 1:4 gradient) to yield the pure product.

Synthesis of 4-allylpyridine (5s)

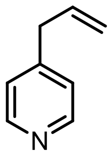

5s was obtained following ***GP(III)*** as a colourless oil (83mg, 70% yield, ***R$_f$*** (EtOAc/Hex, 2:1) = 0.30). **^{1}H NMR (600 MHz, $CDCl_3$)** δ 8.50 (d, *J* = 6.0 Hz, 2H), 7.11 (d, *J* = 5.8 Hz, 2H), 5.92 (ddt, *J* = 16.9, 10.1, 6.7 Hz, 1H), 5.18 – 5.08 (m, 2H), 3.37 (d, *J* = 6.7 Hz, 2H). **^{13}C NMR (151 MHz, $CDCl_3$)** δ 149.8, 148.9, 135.1, 123.9, 117.4, 39.3. **HRMS for [$C_8H_{10}N$]$^+$** calcld. 120.0808 found 120.0807. Spectroscopic data were consistent with literature values.[33]

Synthesis of 1-allylisoquinoline (5t)

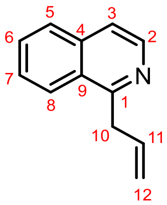

5t was obtained following ***GP(III)*** as a colourless oil (117mg, 70% yield, **R$_f$** (EtOAc/Hex, 2:1) = 0.49). **^{1}H NMR (600 MHz, $CDCl_3$)** δ 8.46 (d, *J* = 5.7 Hz, 1H, H_2), 8.16 (d, *J* = 8.5 Hz, 1H, H_8), 7.81 (d, *J* = 8.2 Hz, 1H, H_5), 7.67 (t, *J* = 7.5 Hz, 1H), 7.59 (t, *J* = 7.7 Hz, 1H), 7.53 (d, *J* = 5.7 Hz, 1H, H_3), 6.22 (ddt, *J* = 16.8, 10.1, 6.5 Hz, 1H, H_{11}), 5.23 – 5.12 (m, 2H, H_{12}), 4.10 (d, *J* = 6.5 Hz, 2H, H_{10}). **^{13}C NMR (151 MHz, $CDCl_3$)** δ 159.7 (C_1), 142.1 (C_2), 136.3 (C_4), 135.4 (C_{11}), 129.9 (C_6), 127.3 (C_8), 127.1 (C_9), 127.0 (C_7), 125.4 (C_5), 119.6 (C_3), 116.8 (C_{12}), 40.2

(C_{10}). **IR (ATR – neat)** $\tilde{\nu}$ (cm^{-1}) = 3055, 1639, 1623, 1588, 1562, 1500, 1385, 1352, 1340, 1144, 991, 906, 820, 745. **HRMS for $C_{12}H_{12}N$** calcld. 170.0964 found 170.0959

Synthesis of 4-allyl-1H-pyrrolo[2,3-b]pyridine (5u)

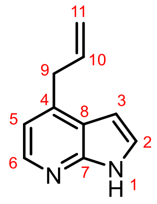

5u was obtained following ***GP(III)*** (using 5 mL of acetone instead of 1.5 mL because of the low starting material solubility) as a white solid (113mg, 71% yield, **R_f** (EtOAc/Hex, 1:1) = 0.19).**^{1}H NMR (600 MHz, $CDCl_3$)** δ 11.88 (s, 1H, H_1), 8.30 (d, *J* = 4.9 Hz, 1H, H_6), 7.40 (d, *J* = 3.2 Hz, 1H, H_2), 6.96 (d, *J* = 4.9 Hz, 1H, H_5), 6.57 (d, *J* = 3.3 Hz, 1H, H_3), 6.09 (ddt, *J* = 16.8, 10.0, 6.7 Hz, 1H, H_{10}), 5.19 (dd, *J* = 25.9, 13.5 Hz, 2H, H_{11}), 3.71 (d, *J* = 6.7 Hz, 2H, H_9). **^{13}C NMR (151 MHz, $CDCl_3$)** δ 148.8 (C_7), 142.5 (C_6), 141.9 (C_4), 135.5 (C_{10}), 124.7 (C_2), 120.2 (C_8), 116.8 (C_5), 115.5 (C_{11}), 98.9 (C_3), 37.4 (C_9). **IR (ATR – neat)** $\tilde{\nu}$ (cm^{-1}) = 3079, 2917, 2829, 1637, 1588, 1493, 1433, 1403, 1340, 1302, 1276, 989, 902, 809, 719. **M.p.** 80°C. **HRMS for $[C_{10}H_{11}N_2]^+$** calcld. 159.0917 found 159.0915

7 NMR spectra

7.1 Organoboron starting materials

2-(3,5-dimethoxybenzyl)-4,4,5,5-tetramethyl-1,3,2-dioxaborolane (2b)

^{1}H-NMR, $CDCl_3$, 600 MHz

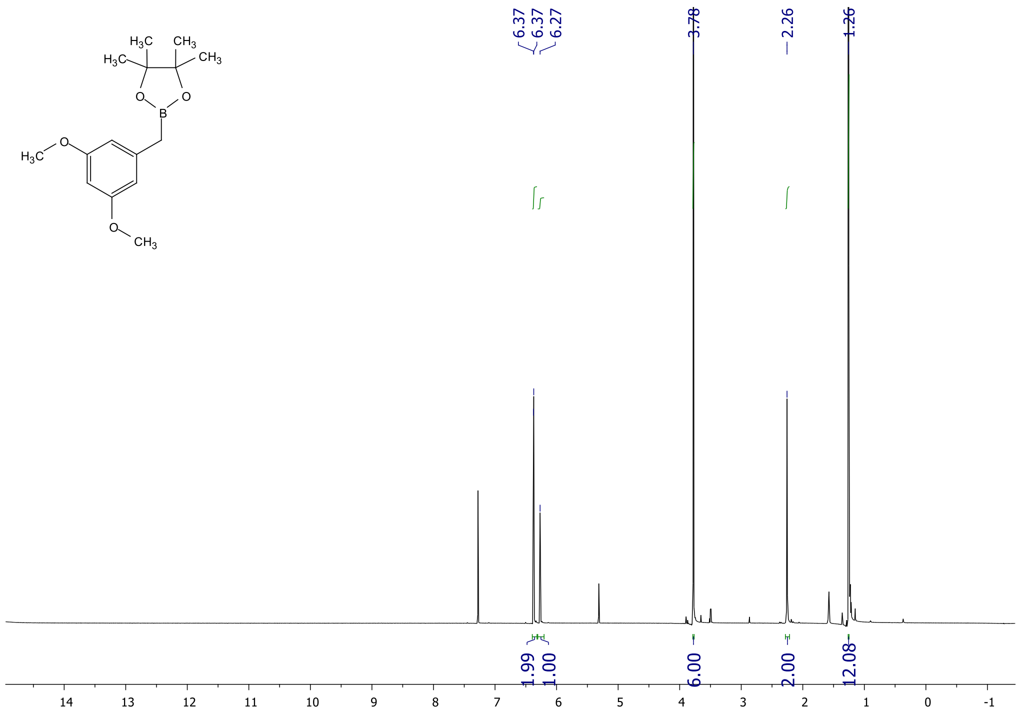

^{13}C-NMR, $CDCl_3$, 151 MHz

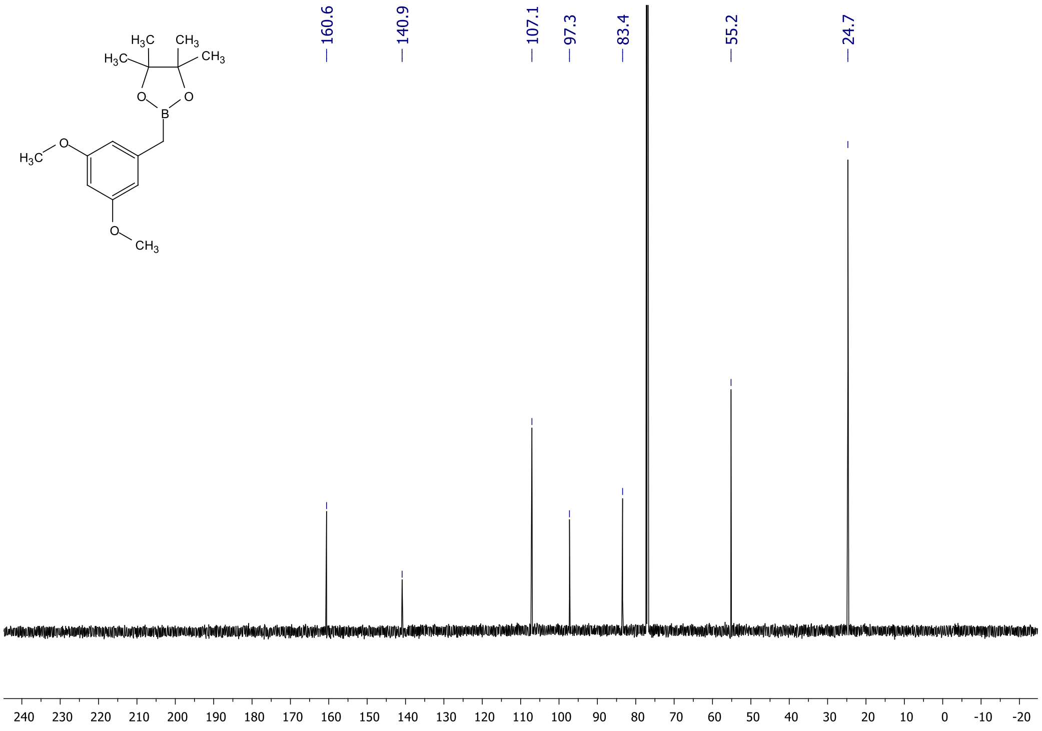

^{11}B-NMR, $CDCl_3$, 193 MHz

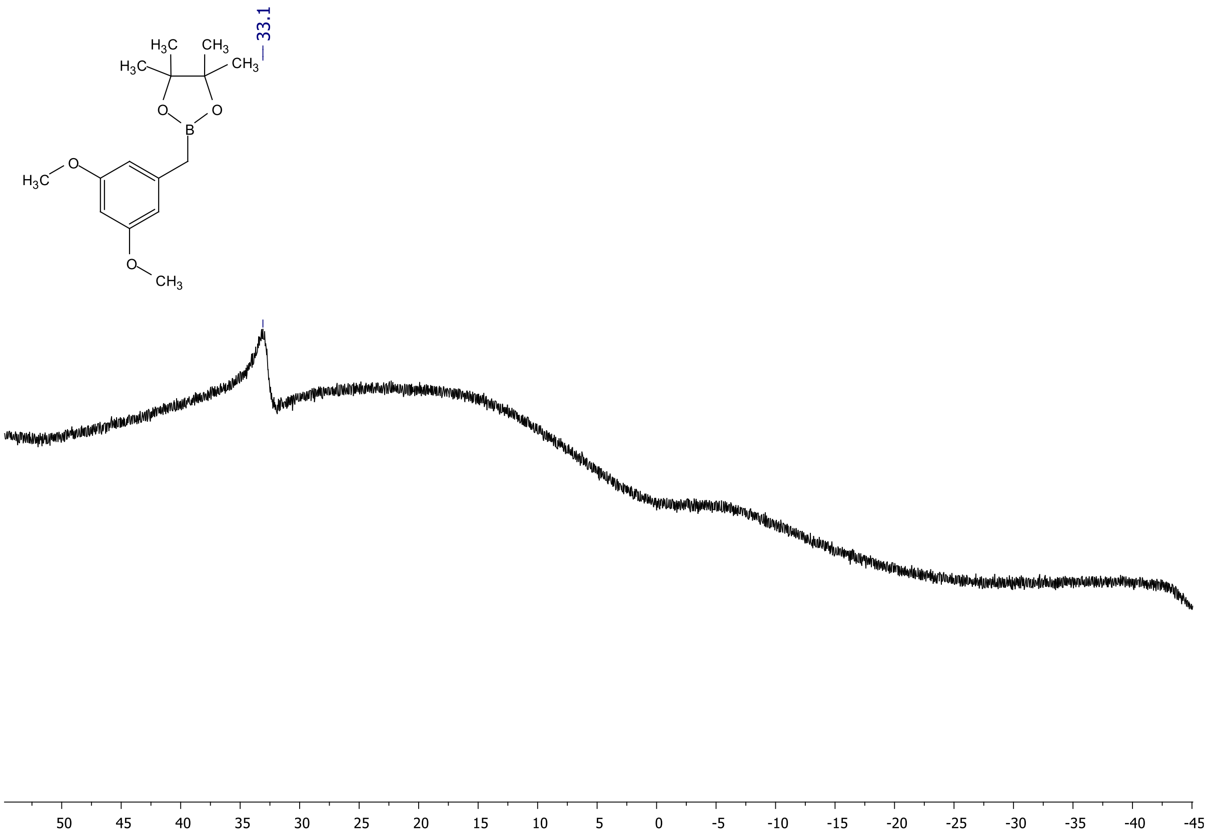

methyl 4-((4,4,5,5-tetramethyl-1,3,2-dioxaborolan-2-yl)methyl)benzoate (2c)

^{1}H-NMR, $CDCl_3$, 600 MHz

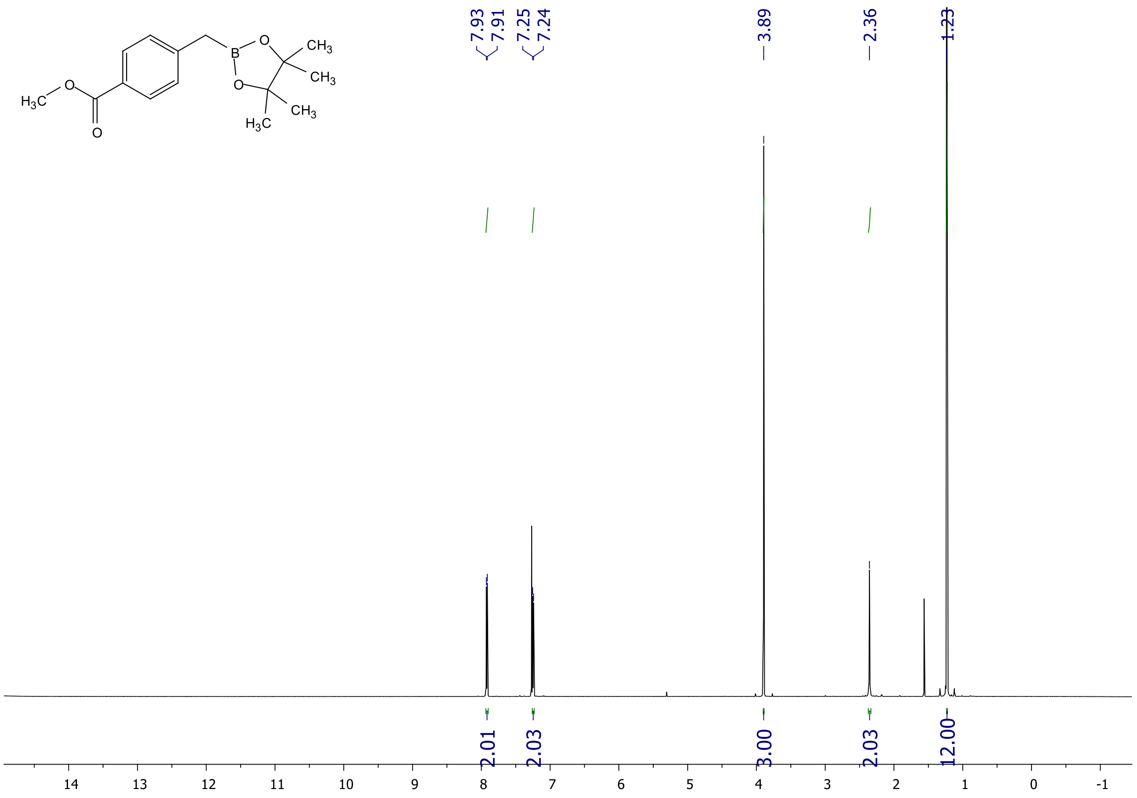

^{13}C-NMR, $CDCl_3$, 151 MHz

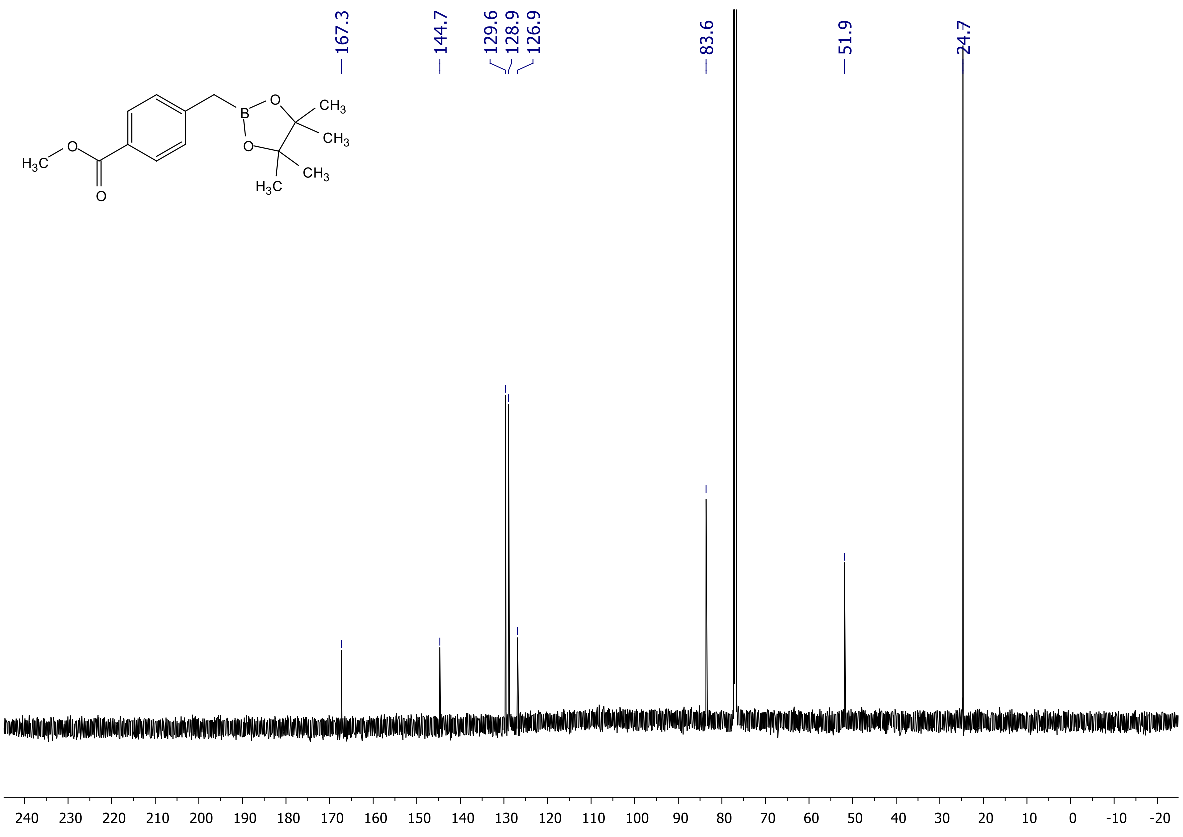

2-(3,5-bis(trifluoromethyl)benzyl)-4,4,5,5-tetramethyl-1,3,2-dioxaborolane (2d)

^{1}H-NMR, $CDCl_3$, 600 MHz

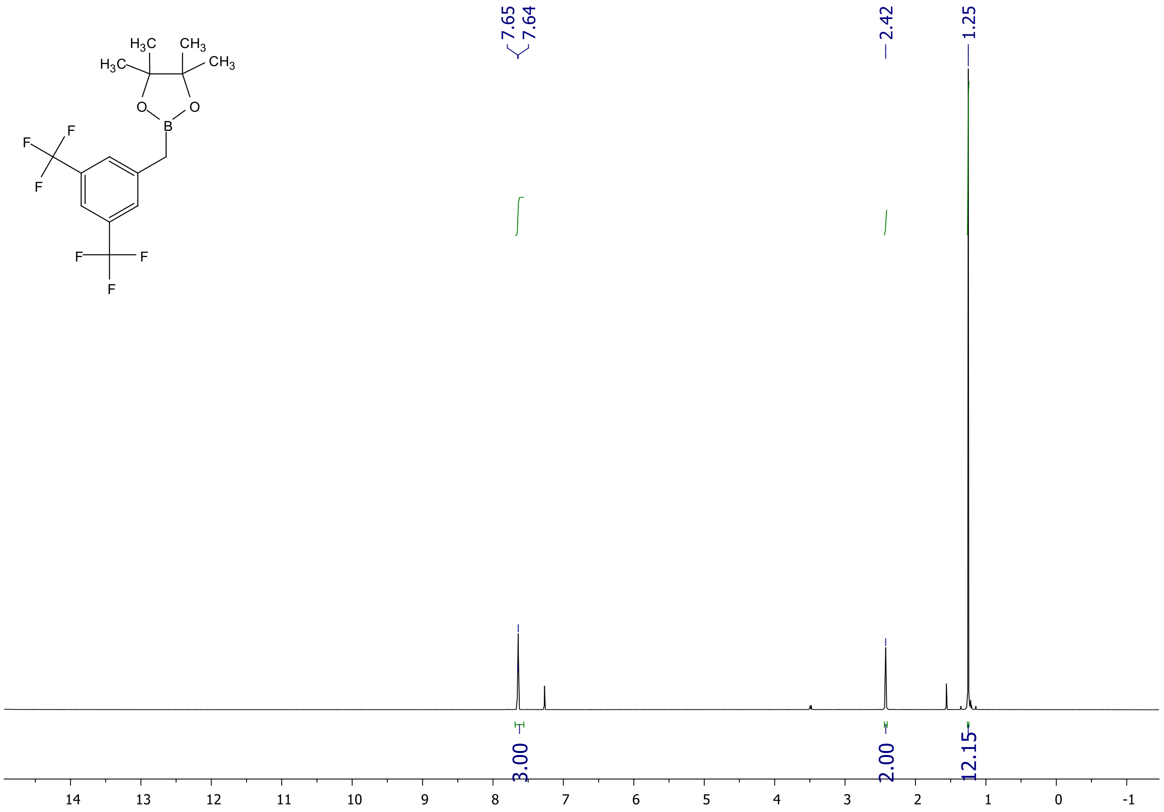

^{13}C-NMR, $CDCl_3$, 151 MHz

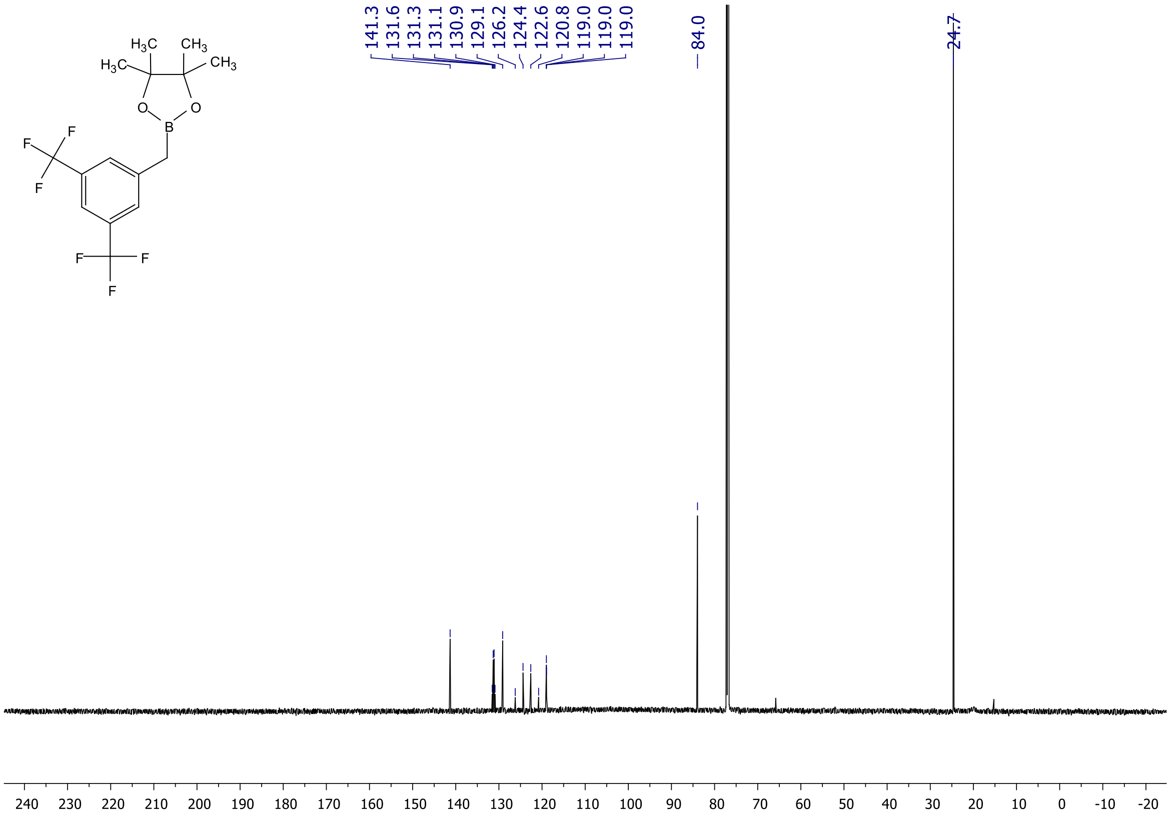

^{11}B-NMR, $CDCl_3$, 193 MHz

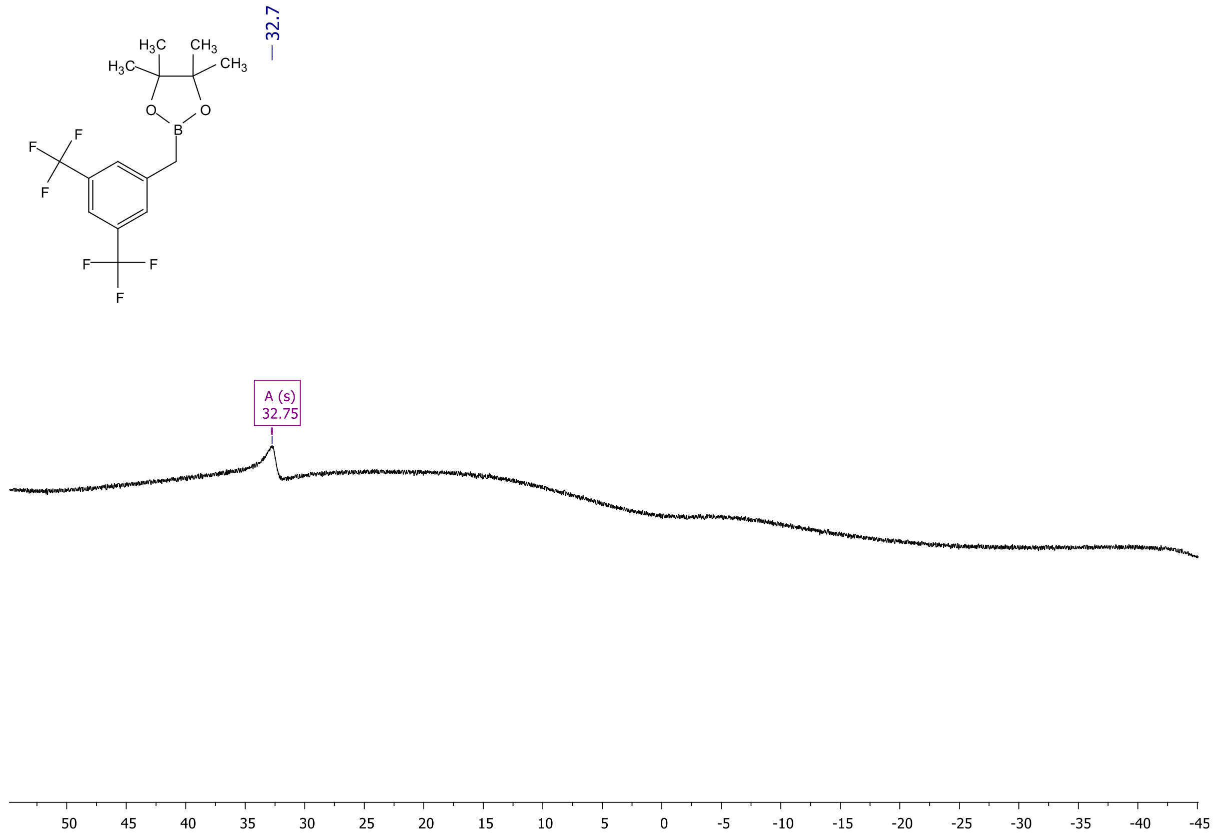

^{19}F-NMR, $CDCl_3$, 376 MHz

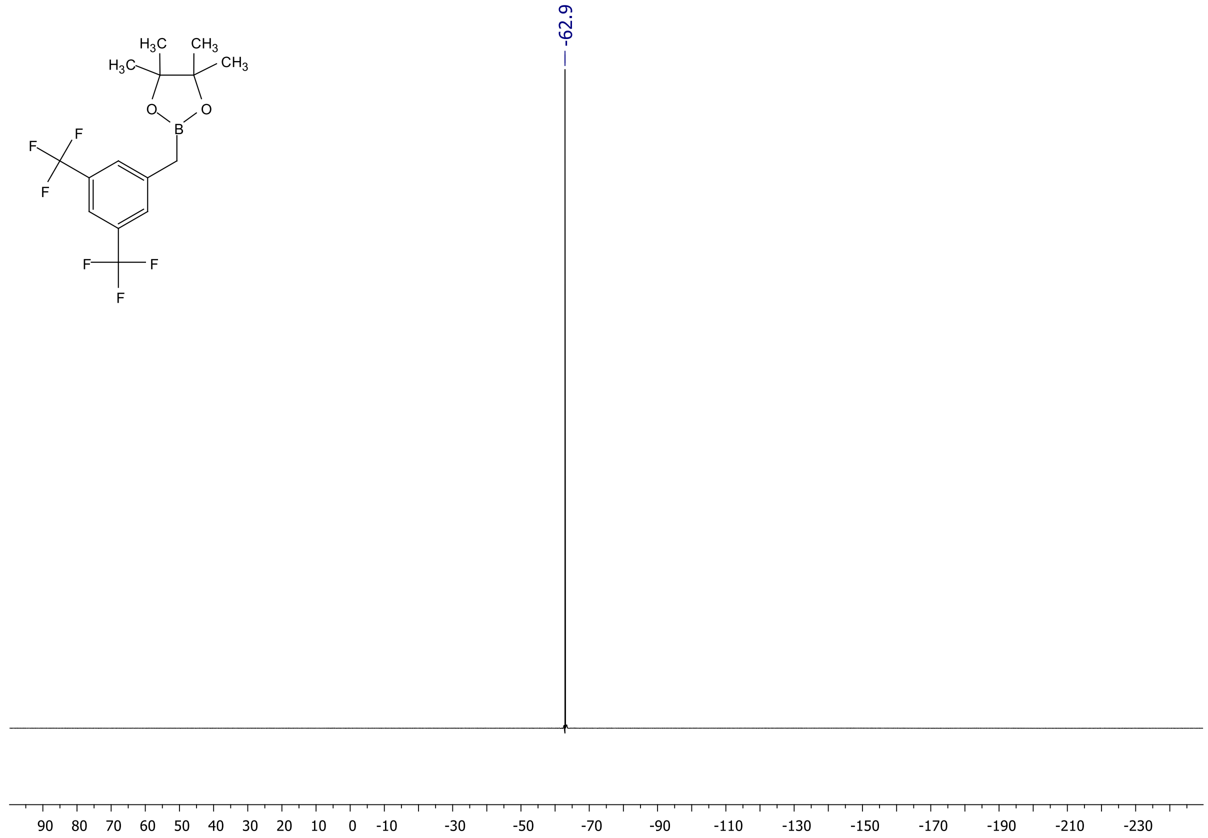

2-(4-fluorobenzyl)-4,4,5,5-tetramethyl-1,3,2-dioxaborolane (2f)

^{1}H-NMR, $CDCl_3$, 600 MHz

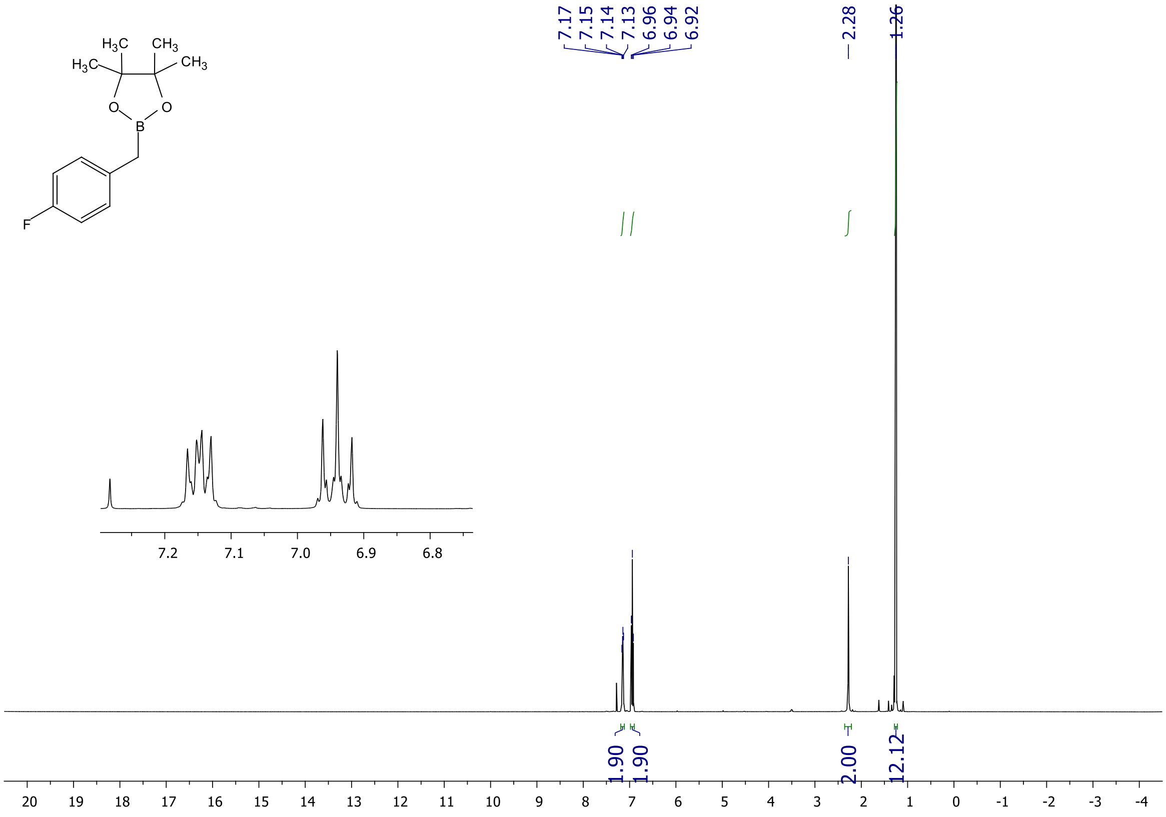

^{13}C-NMR, $CDCl_3$, 151 MHz

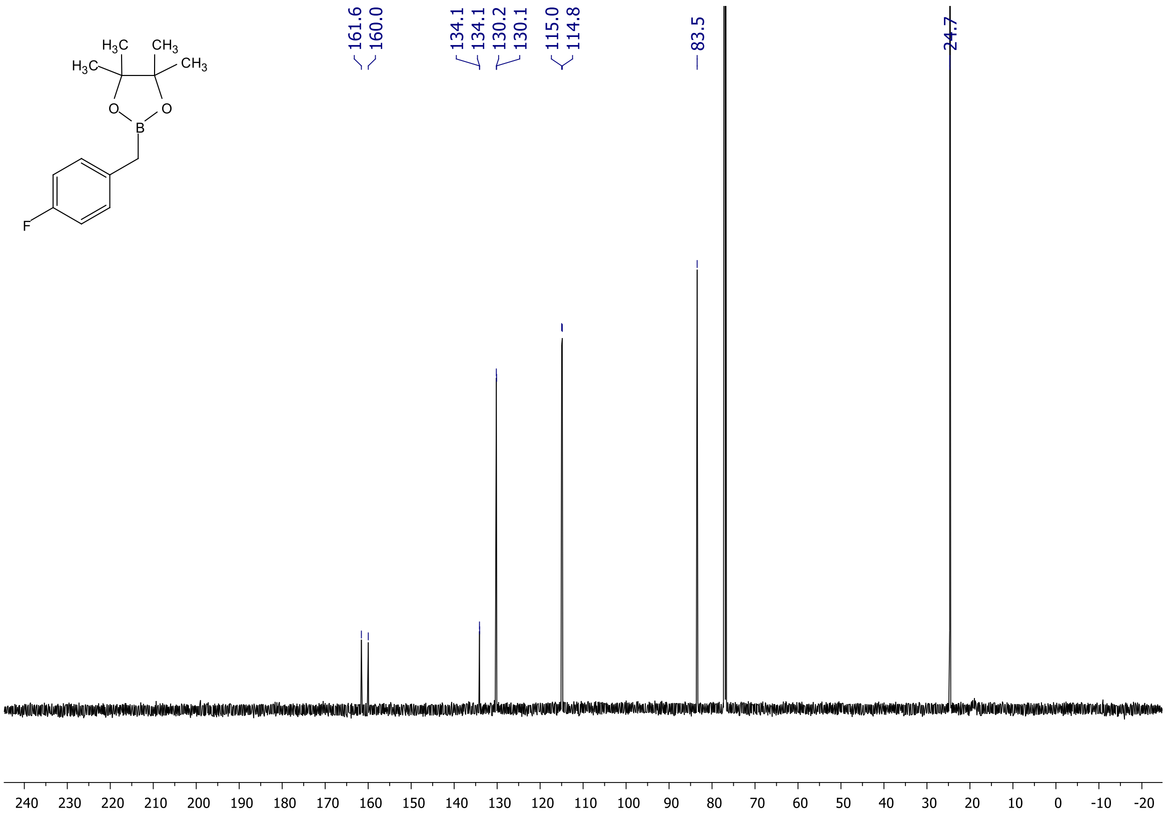

^{11}B-NMR, $CDCl_3$, 193 MHz

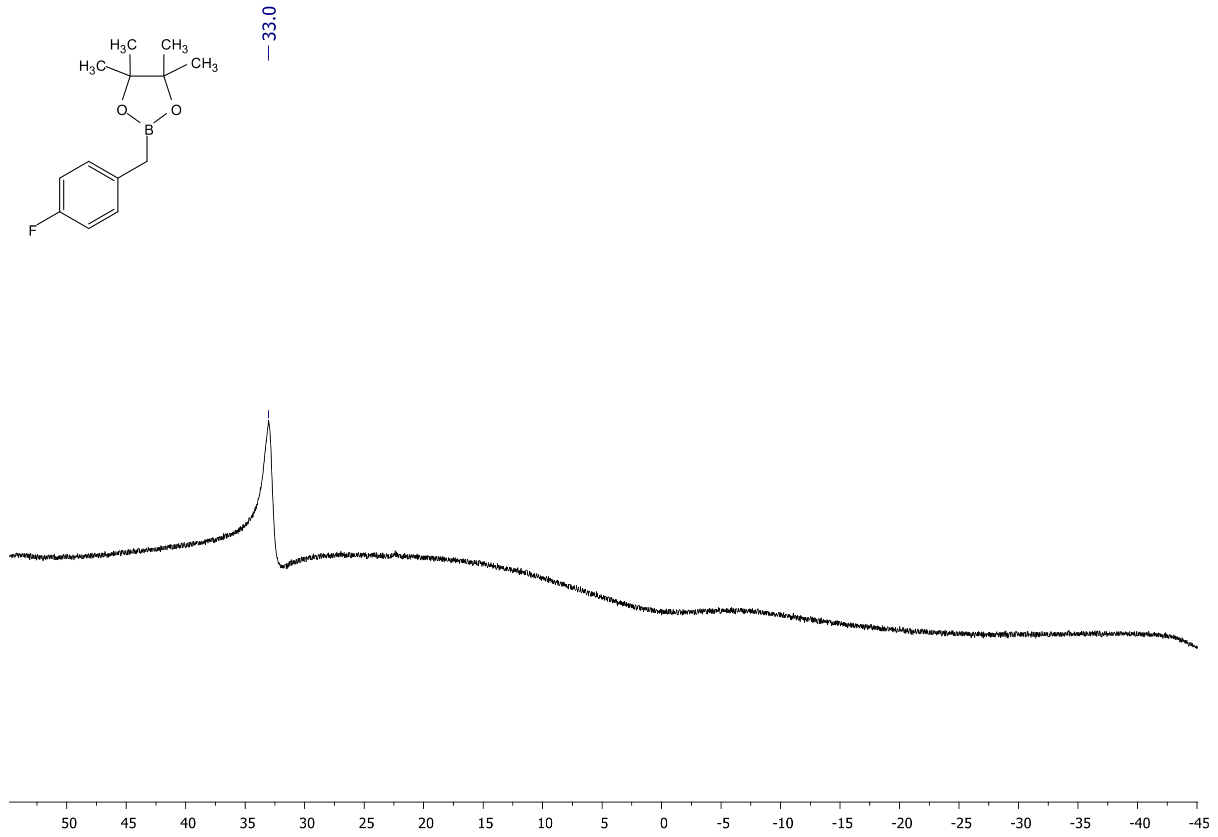

^{19}F-NMR, $CDCl_3$, 377 MHz

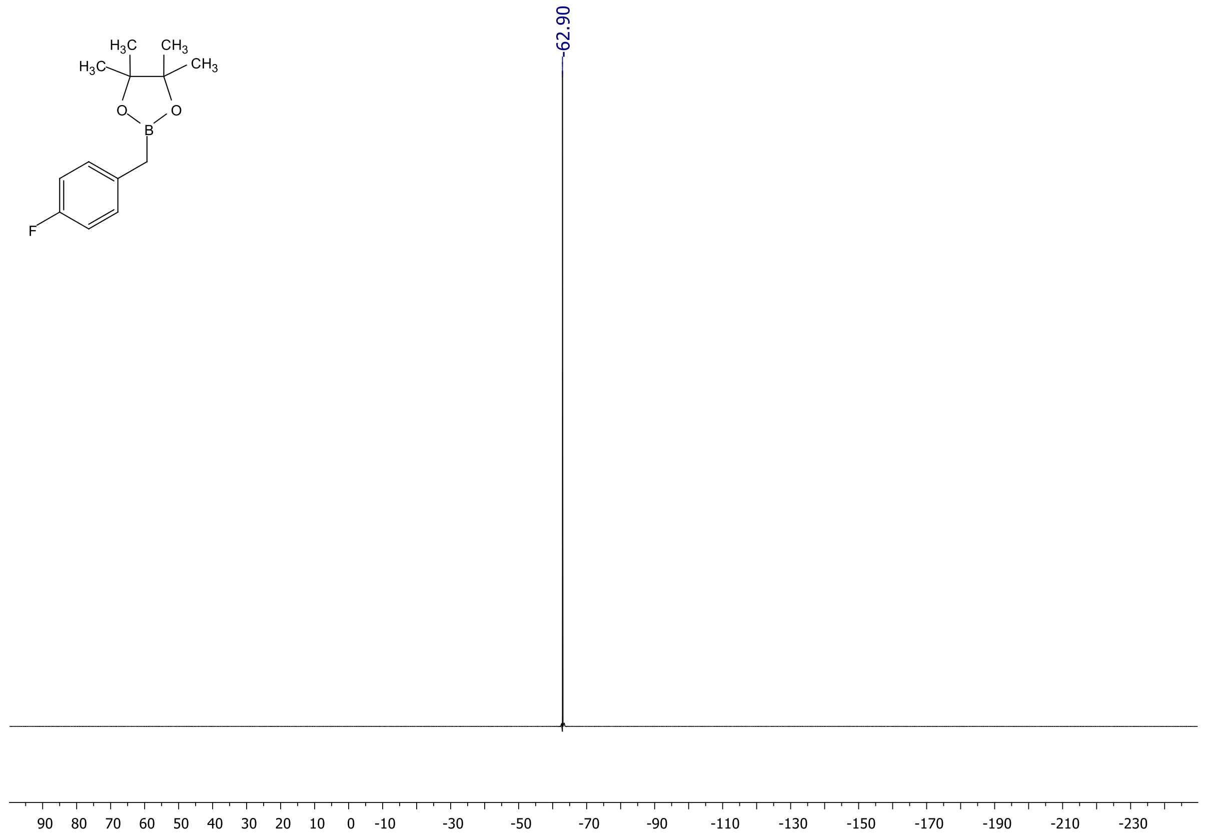

2-(1-(4-methoxyphenyl)ethyl)-4,4,5,5-tetramethyl-1,3,2-dioxaborolane (2h)

1H-NMR, $CDCl_3$, 600 MHz

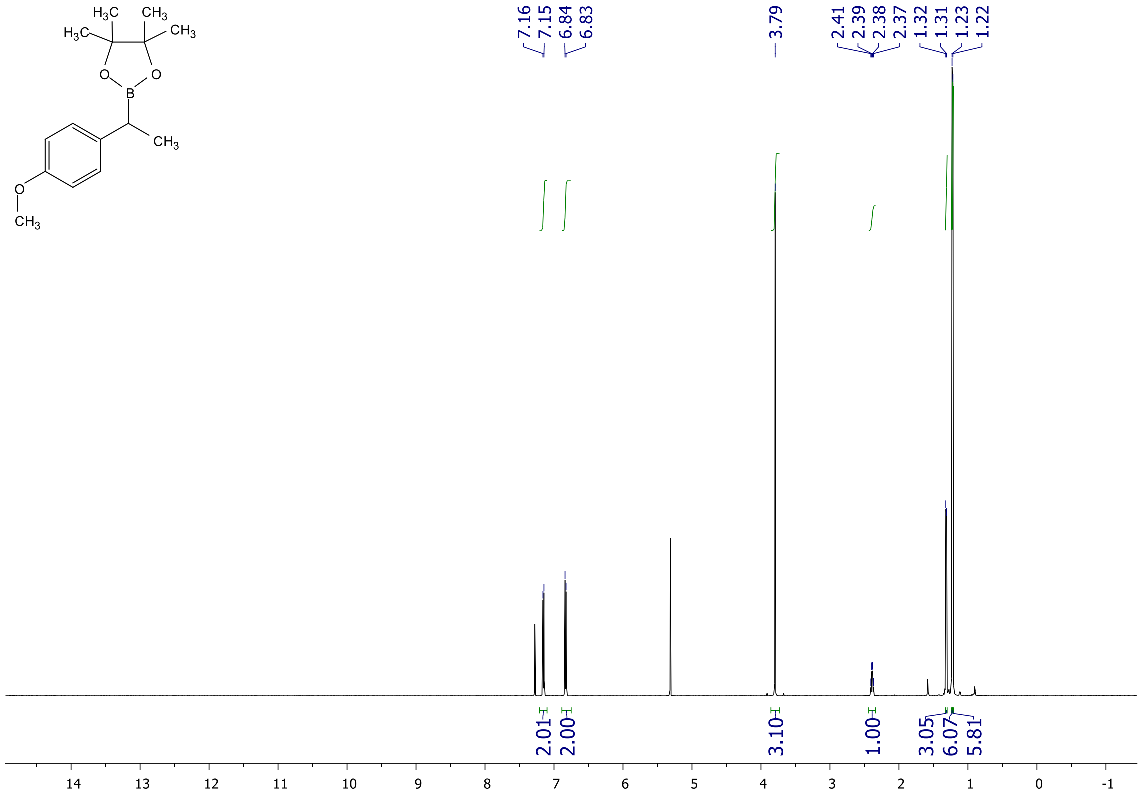

^{13}C-NMR, $CDCl_3$, 151 MHz

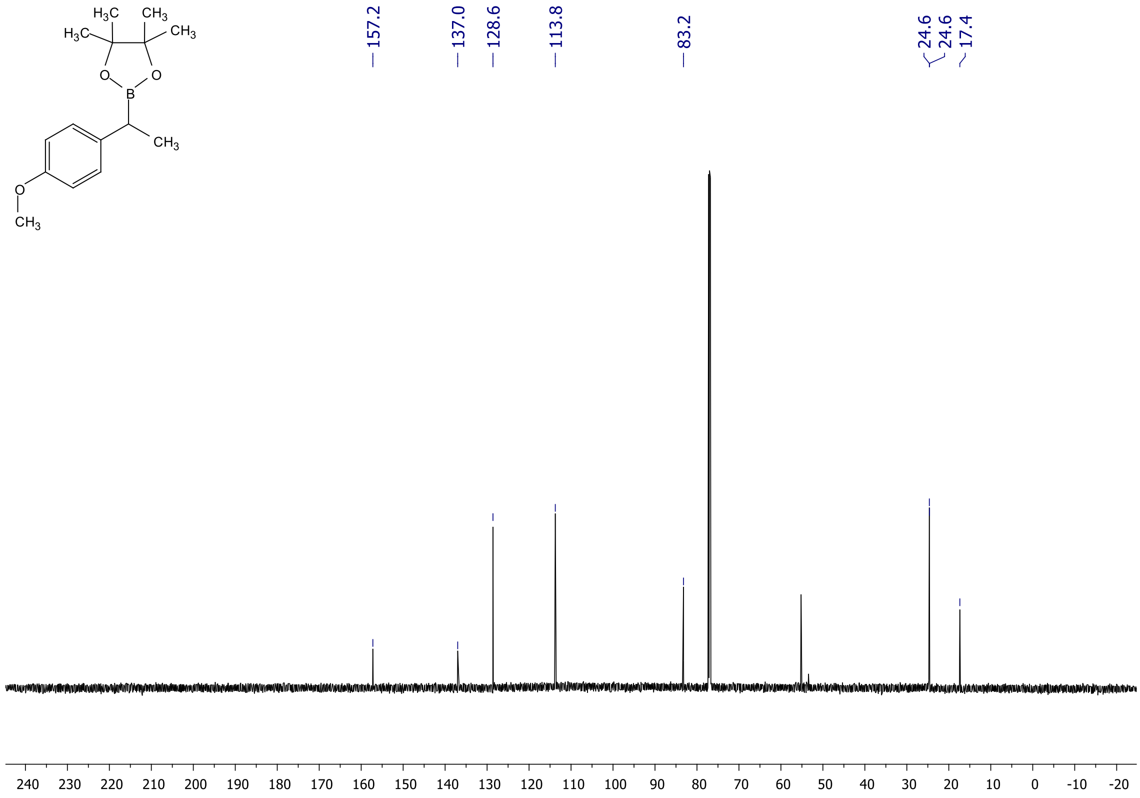

4,4,5,5-tetramethyl-2-(1-phenylethyl)-1,3,2-dioxaborolane (2i)

^{1}H-NMR, $CDCl_3$, 600 MHz

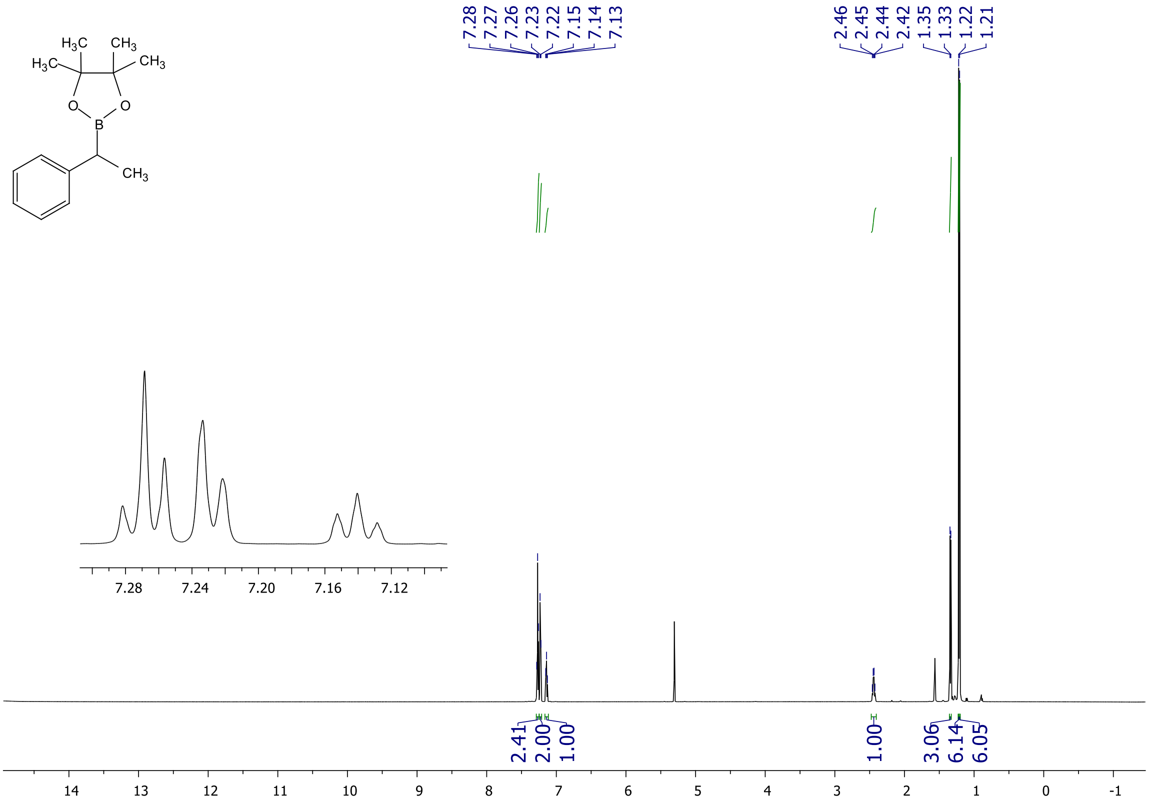

^{13}C-NMR, $CDCl_3$, 151 MHz

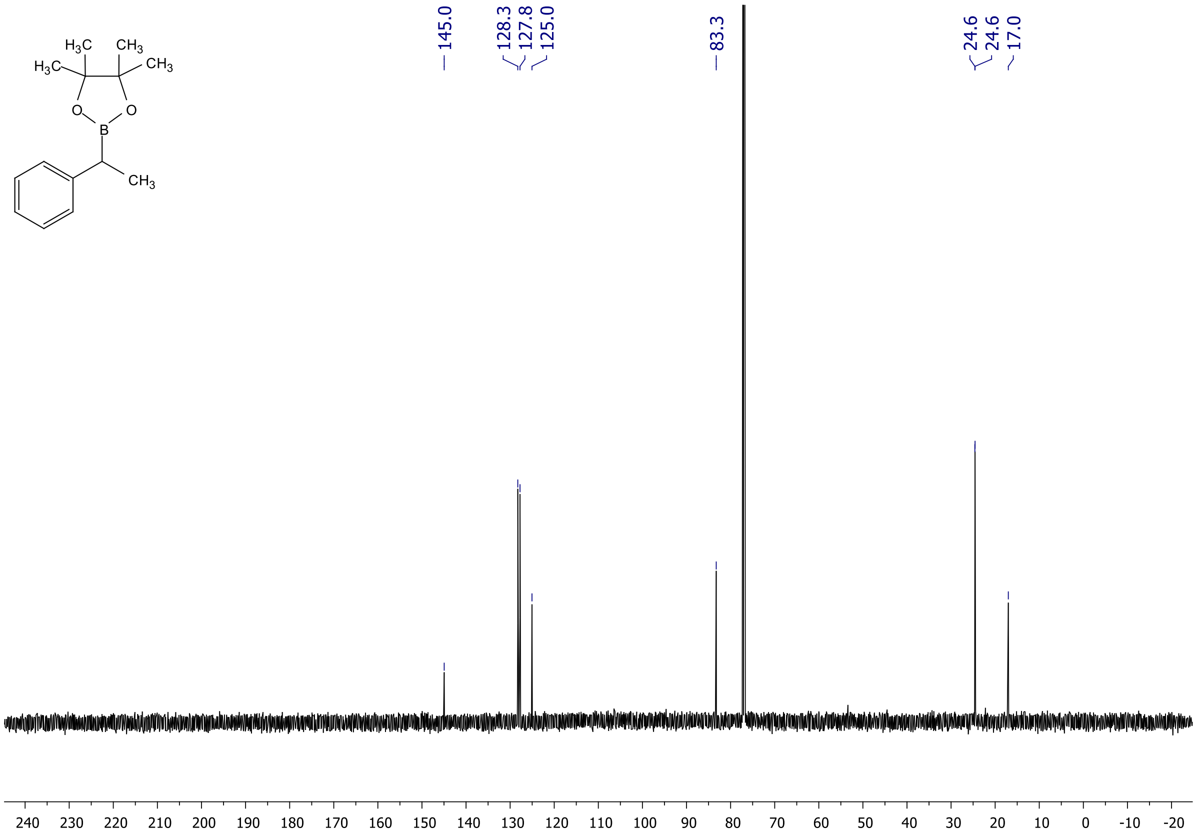

2-benzhydryl-4,4,5,5-tetramethyl-1,3,2-dioxaborolane (2j)

^{1}H-NMR, $CDCl_3$, 600 MHz

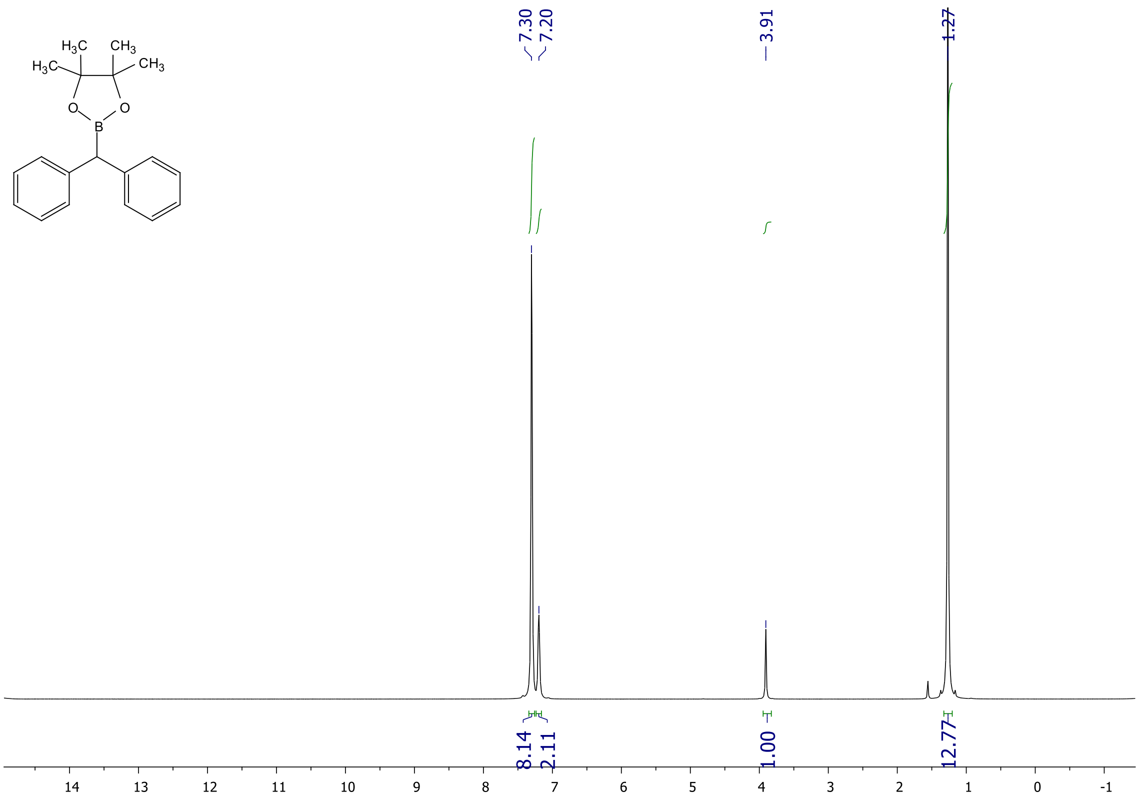

^{13}C-NMR, $CDCl_3$, 151 MHz

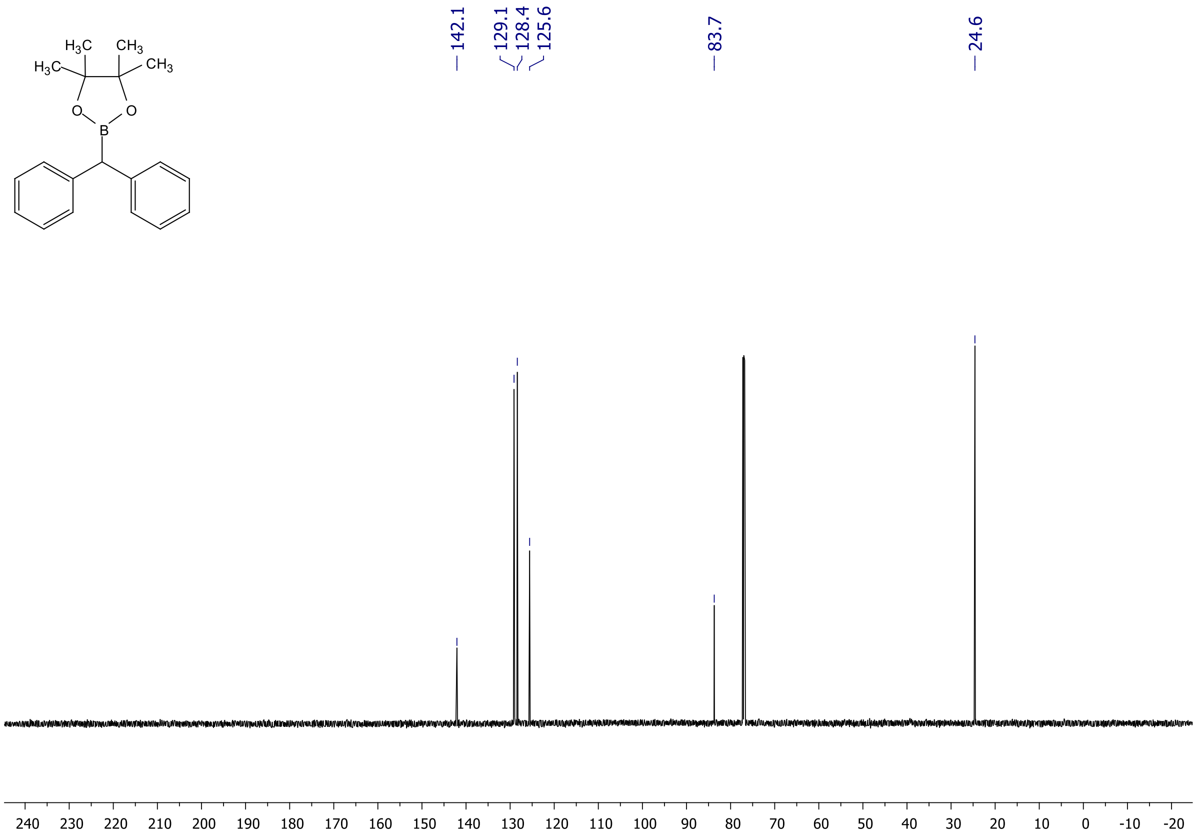

^{11}B-NMR, $CDCl_3$, 193 MHz

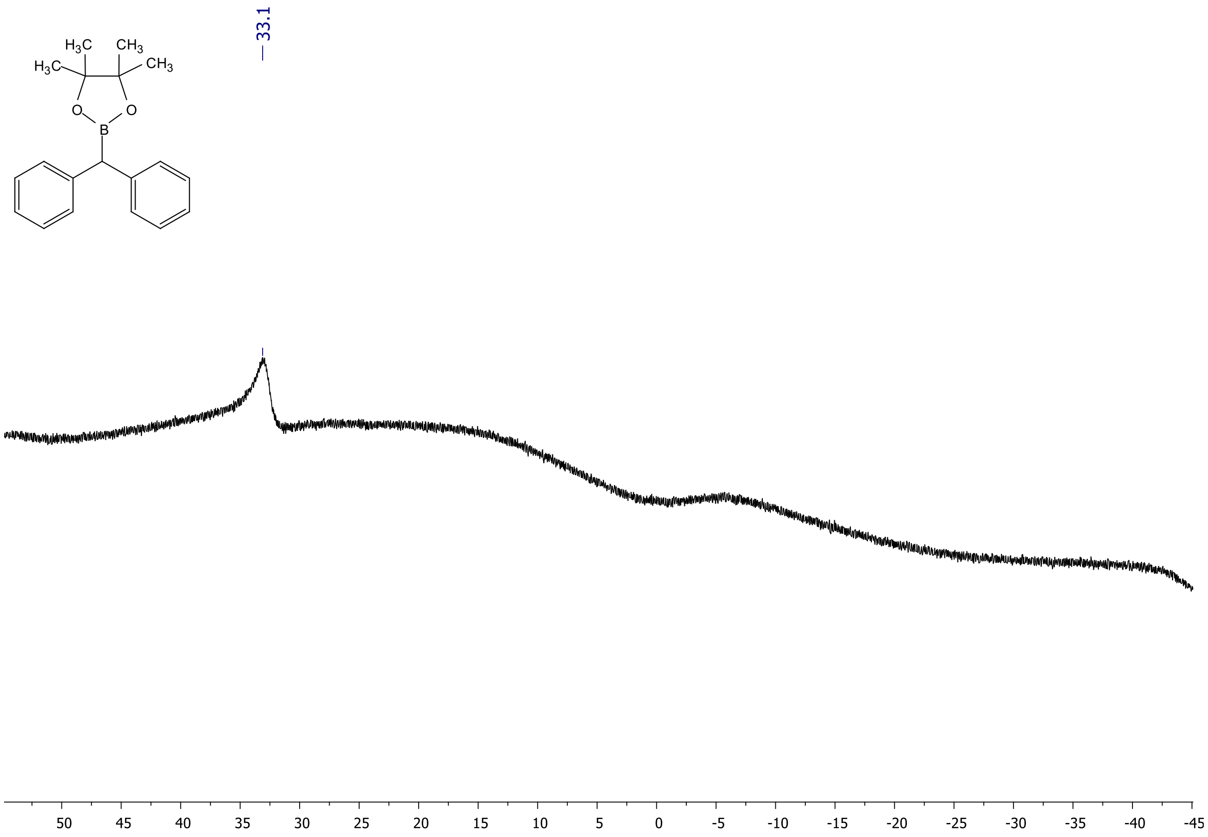

2-(2,6-difluorobenzyl)-4,4,5,5-tetramethyl-1,3,2-dioxaborolane (2k)

^{1}H-NMR, $CDCl_3$, 600 MHz

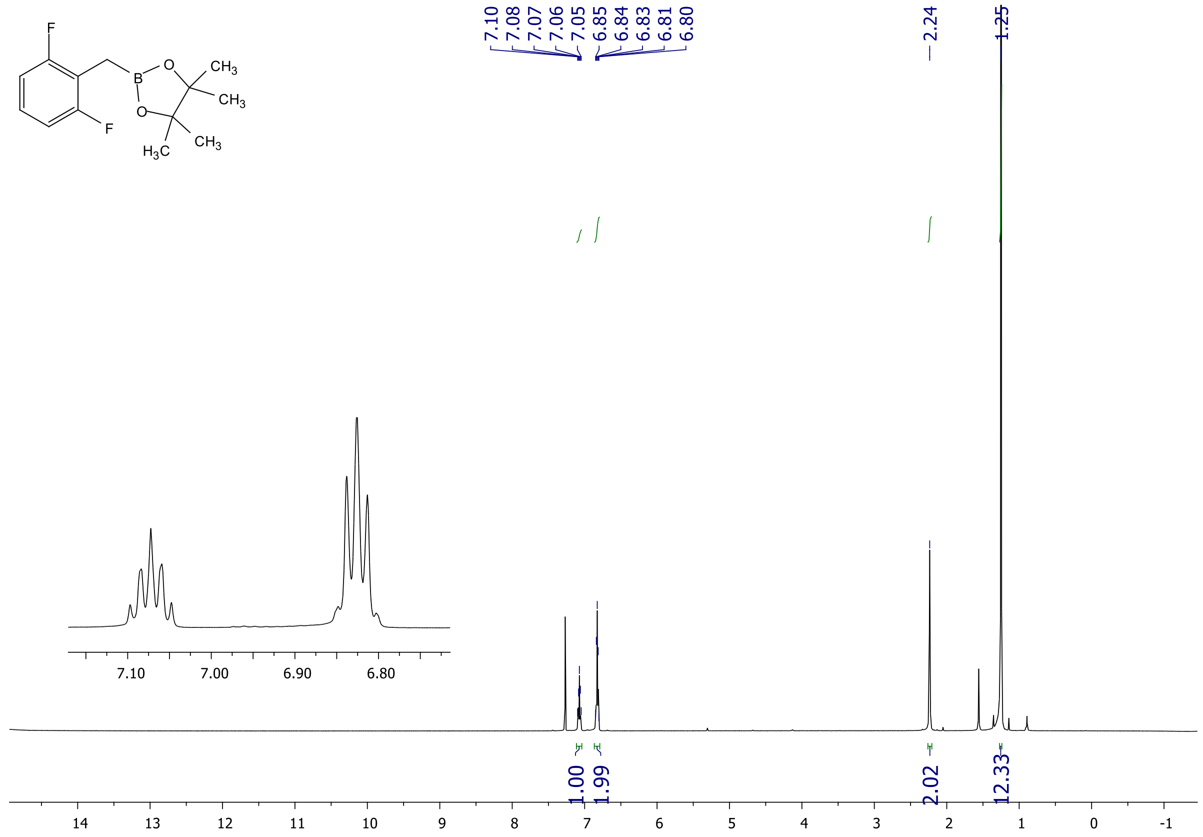

^{13}C-NMR, $CDCl_3$, 151 MHz

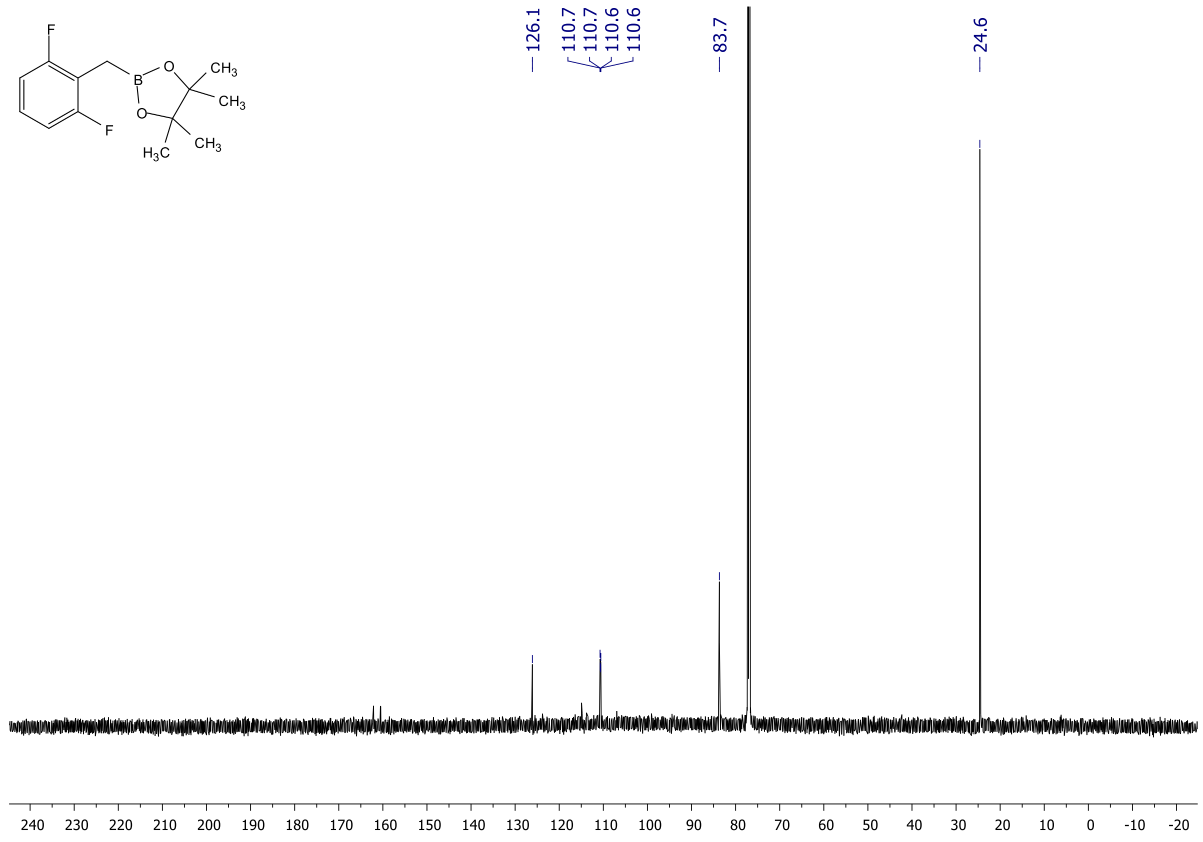

^{11}B-NMR, $CDCl_3$, 193 MHz

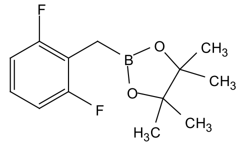

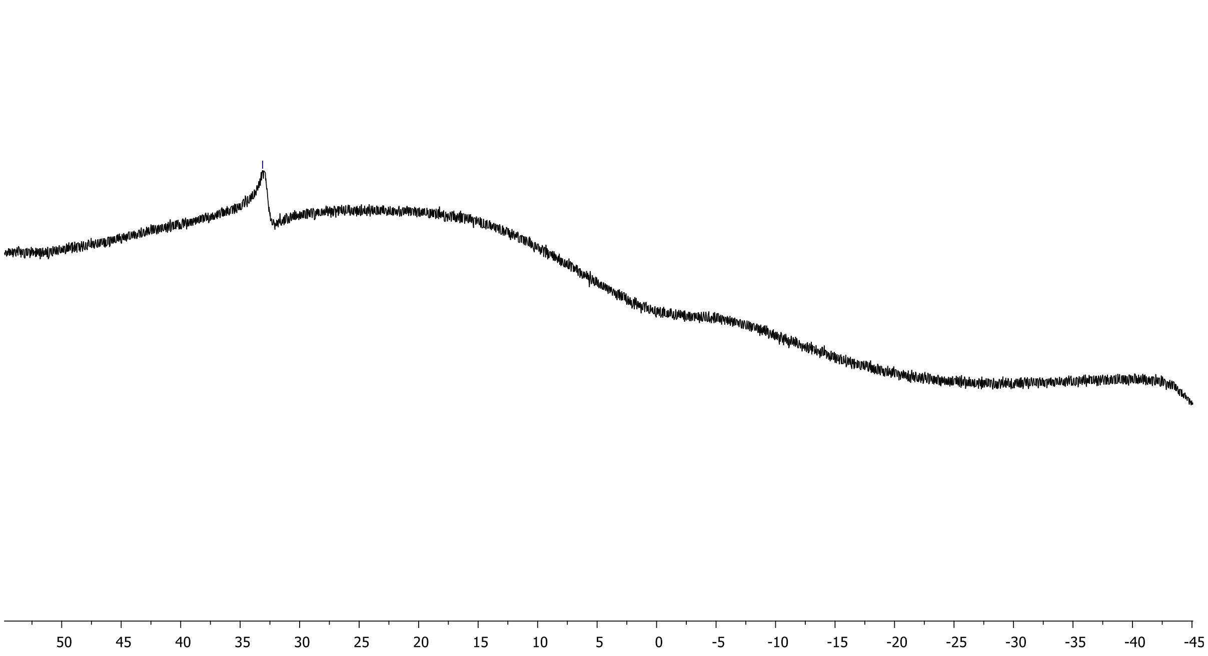

7.2 Coupling Products

7.2.1 Dual Ir/Ni catalysed arylation of benzylboronic esters

4-(4-methoxybenzyl)benzonitrile (3a)

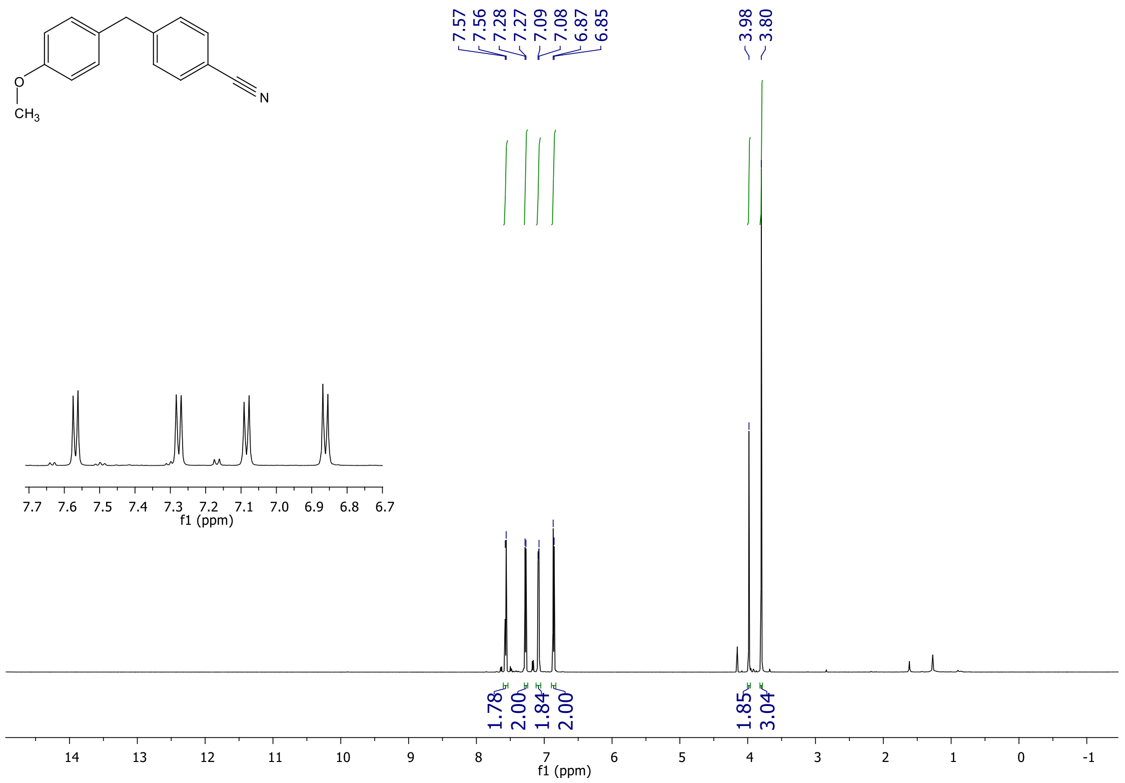

^{13}C-NMR, $CDCl_3$, 150 MHz

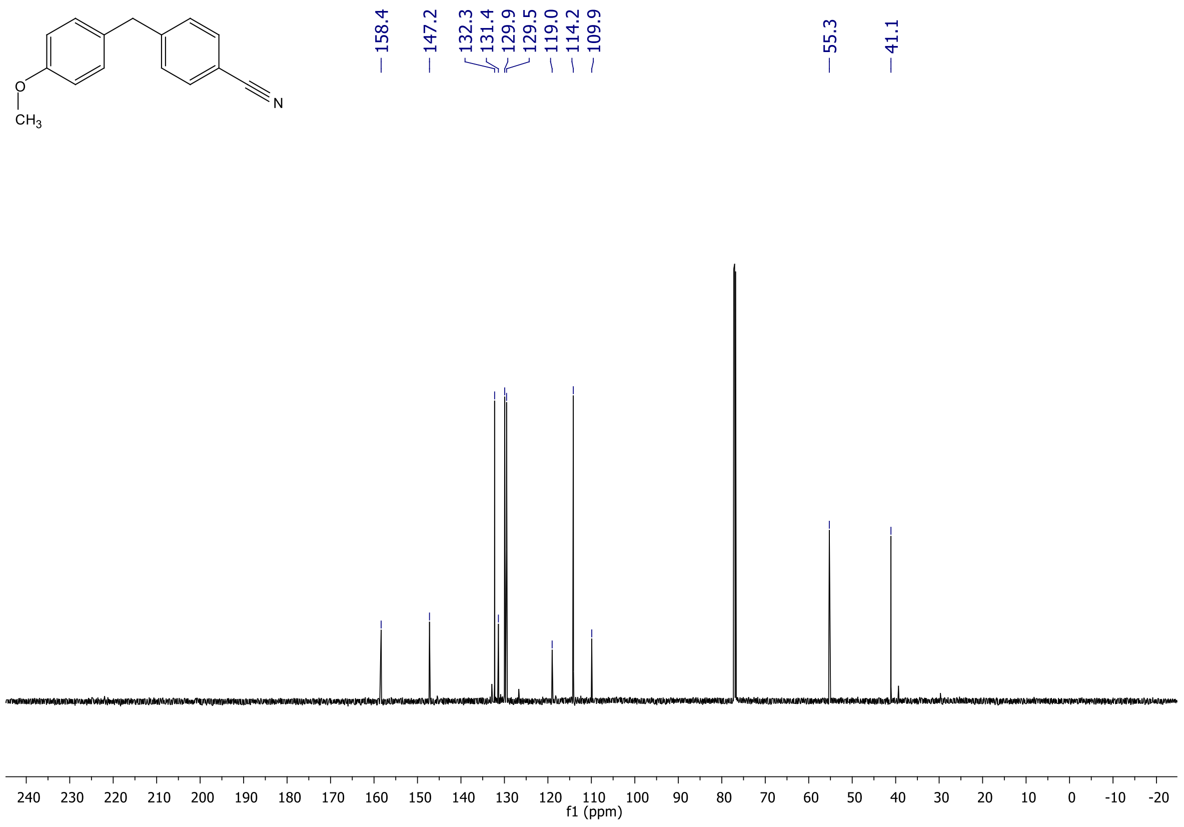

4-(3,5-dimethoxybenzyl)benzonitrile (3b)

^{1}H-NMR, $CDCl_3$, 600 MHz

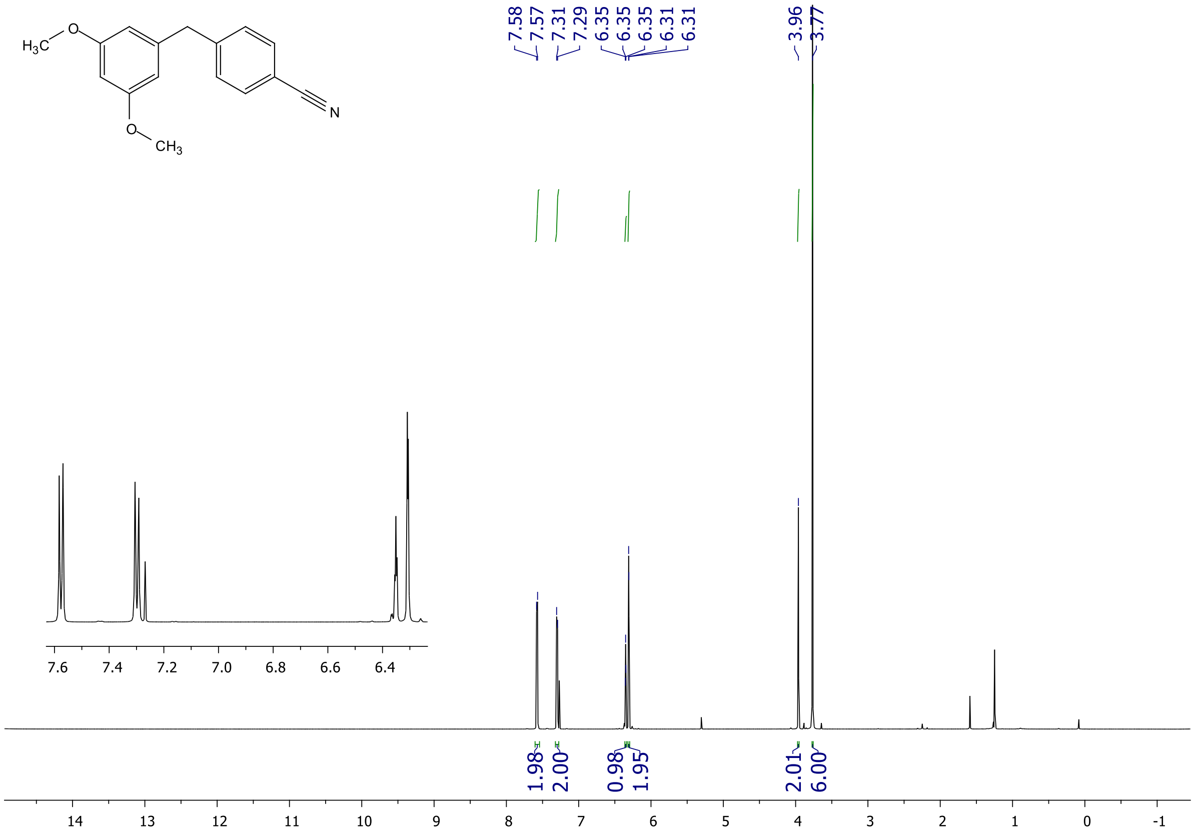

^{13}C-NMR, $CDCl_3$, 150 MHz

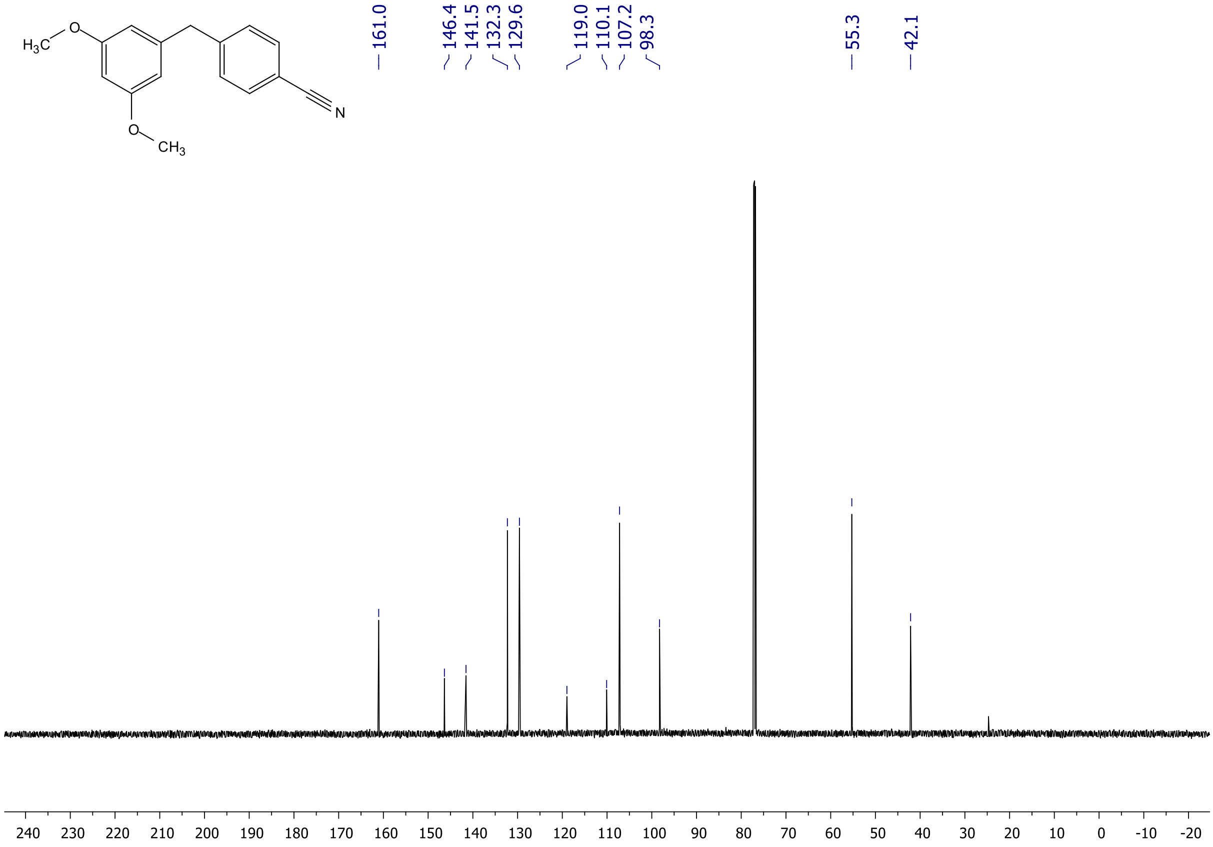

4-(4-cyanobenzyl)benzoate (3c)

^{1}H-NMR, $CDCl_3$, 600 MHz

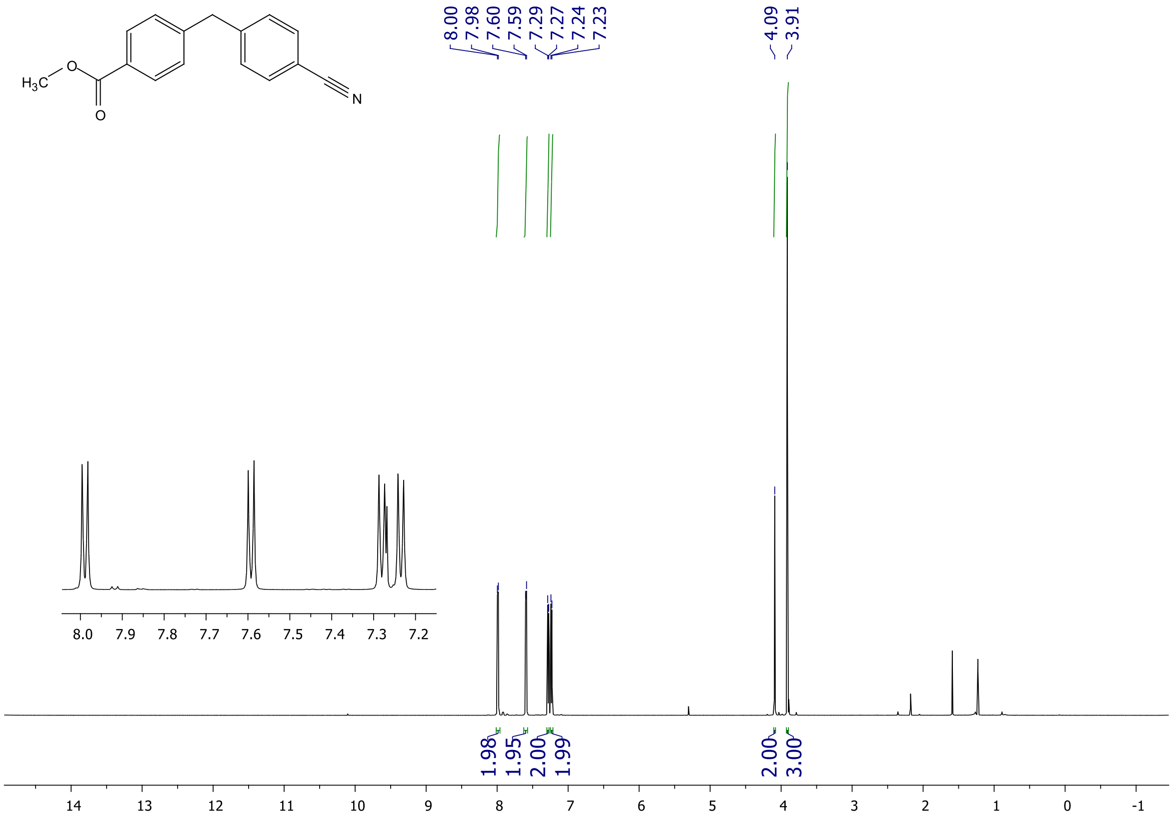

^{13}C-NMR, $CDCl_3$, 150 MHz

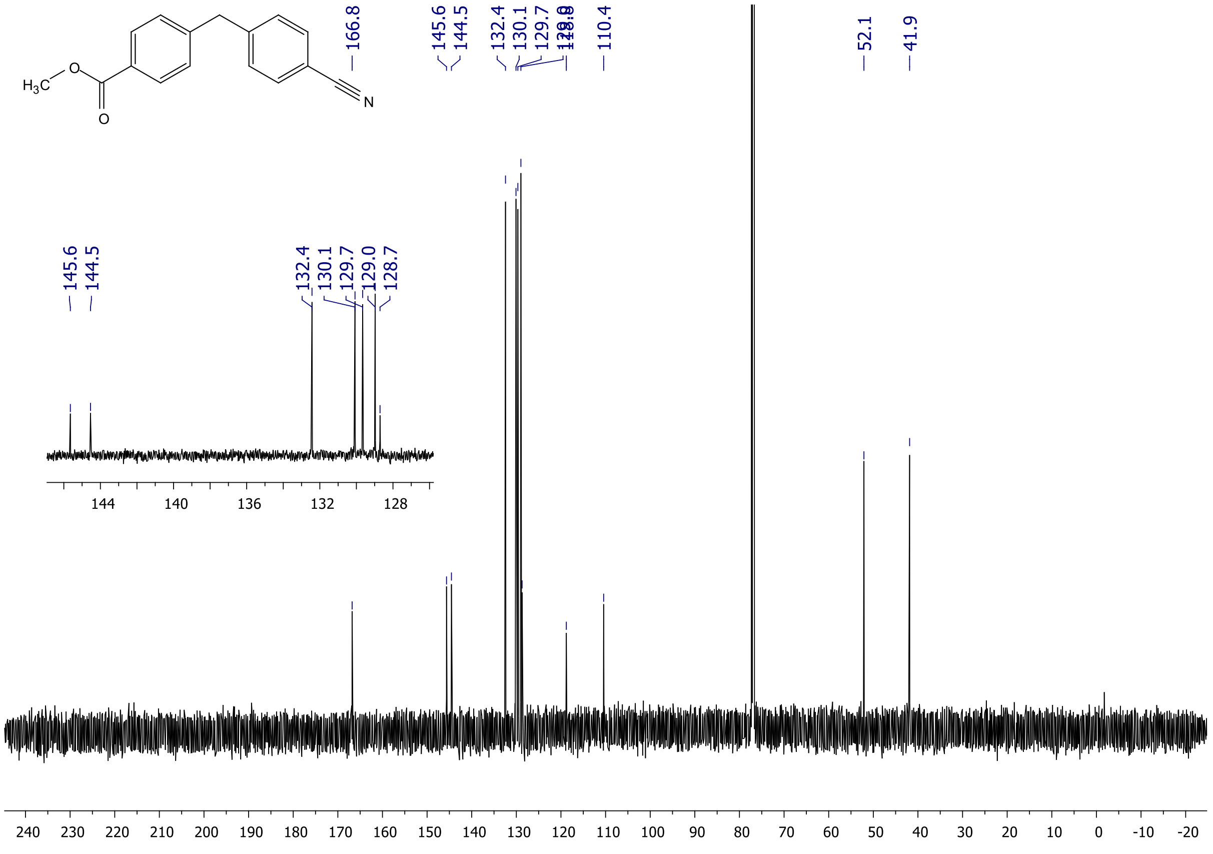

4-(3,5-bis(trifluoromethyl)benzyl)benzonitrile (3d)

^{1}H-NMR, $CDCl_3$, 600 MHz

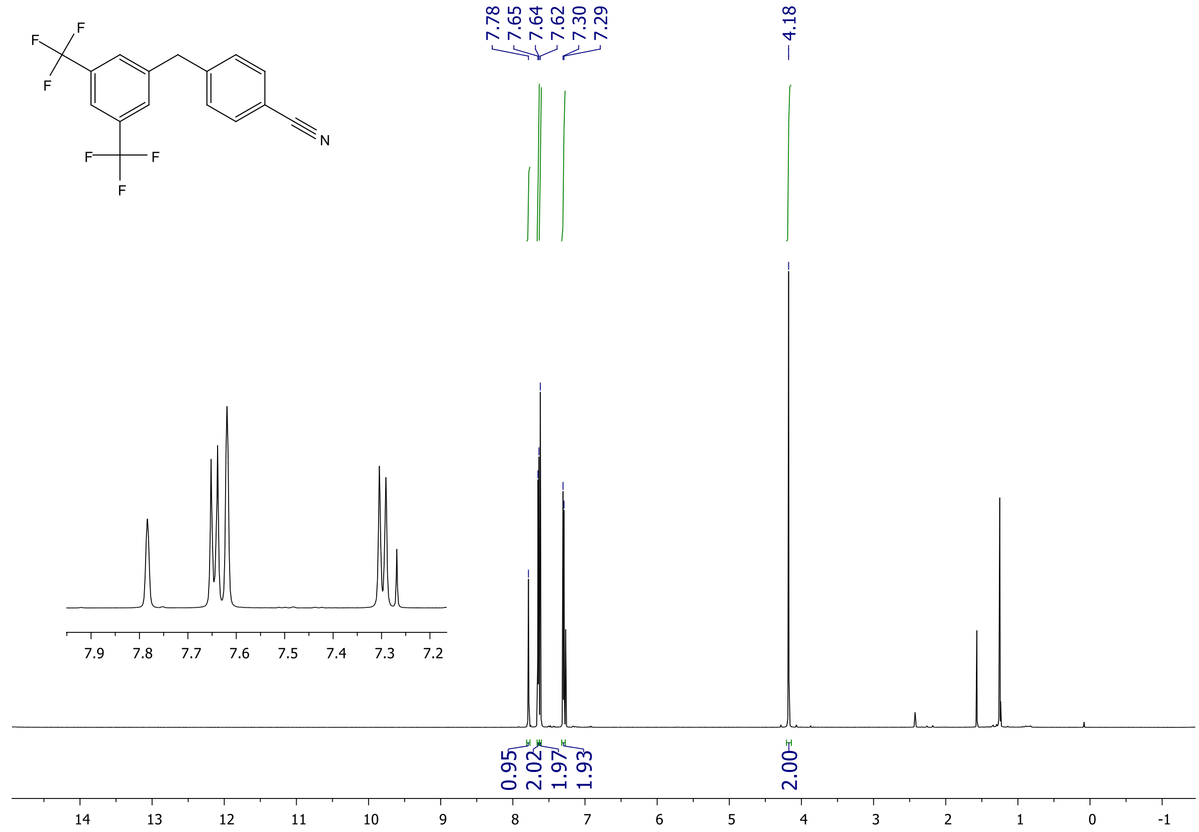

^{13}C-NMR, $CDCl_3$, 150 MHz

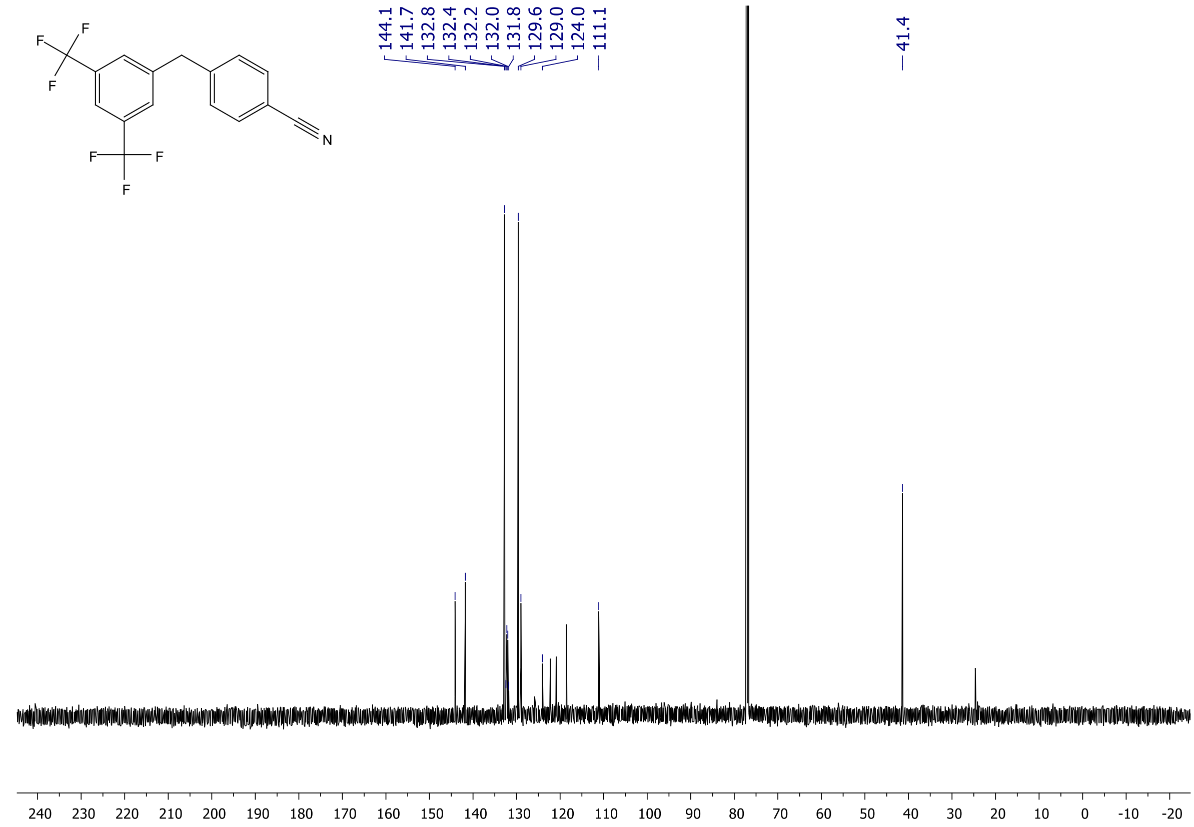

4-(4-methylbenzyl)benzonitrile (3e)

^{1}H-NMR, CDCl$_3$, 600 MHz

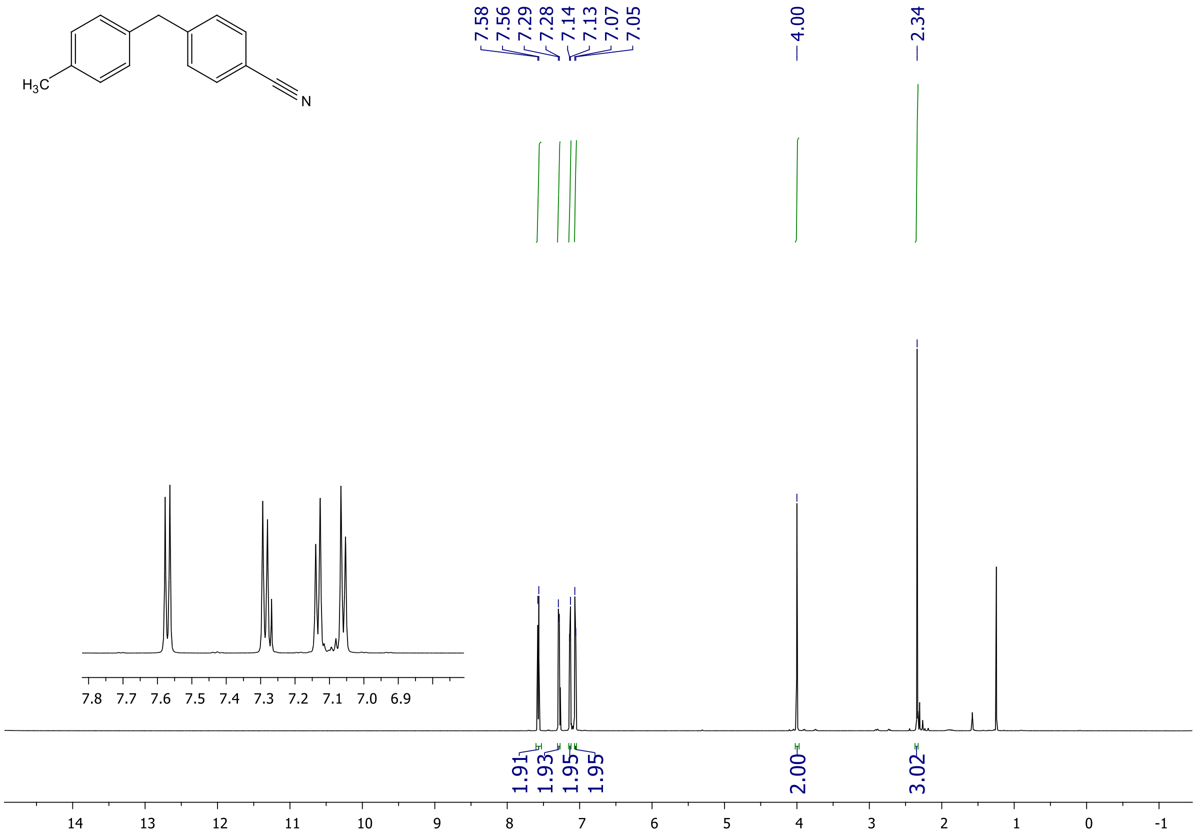

^{13}C-NMR, CDCl$_3$, 150 MHz

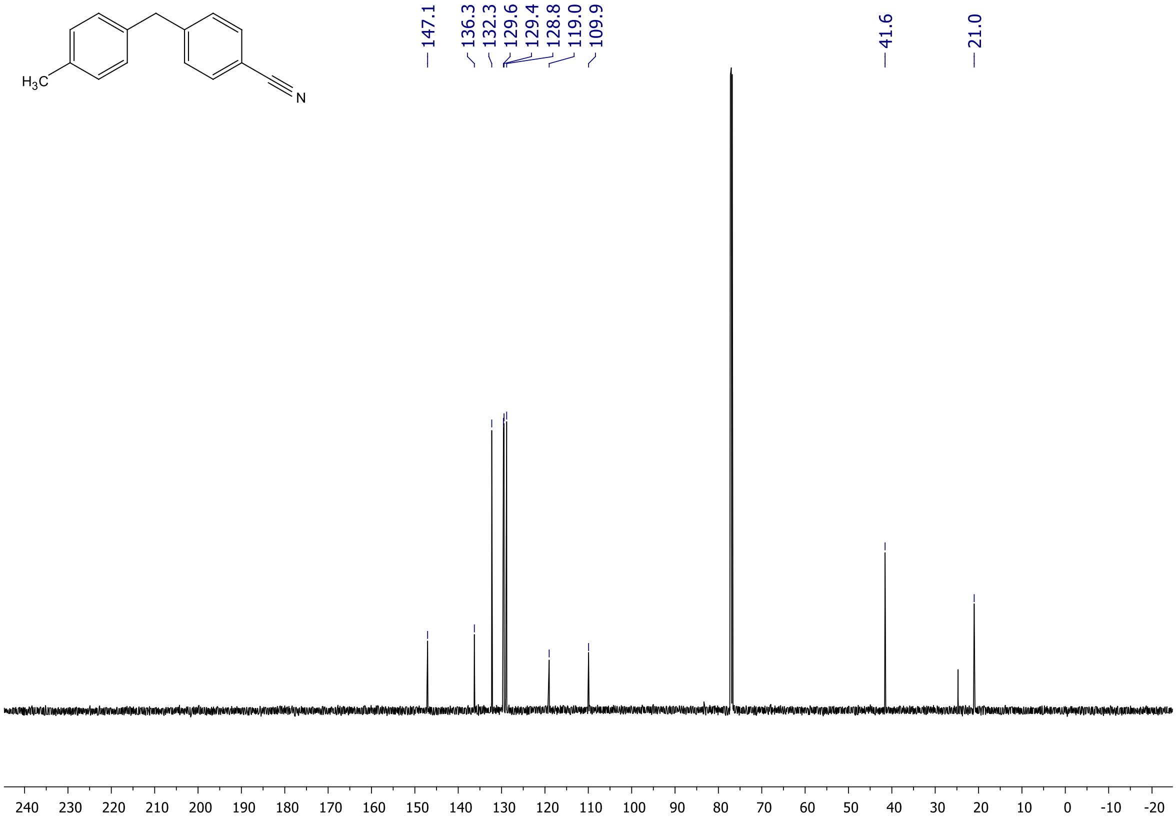

4-(4-fluorobenzyl)benzonitrile (3f)

^{1}H-NMR, $CDCl_3$, 600 MHz

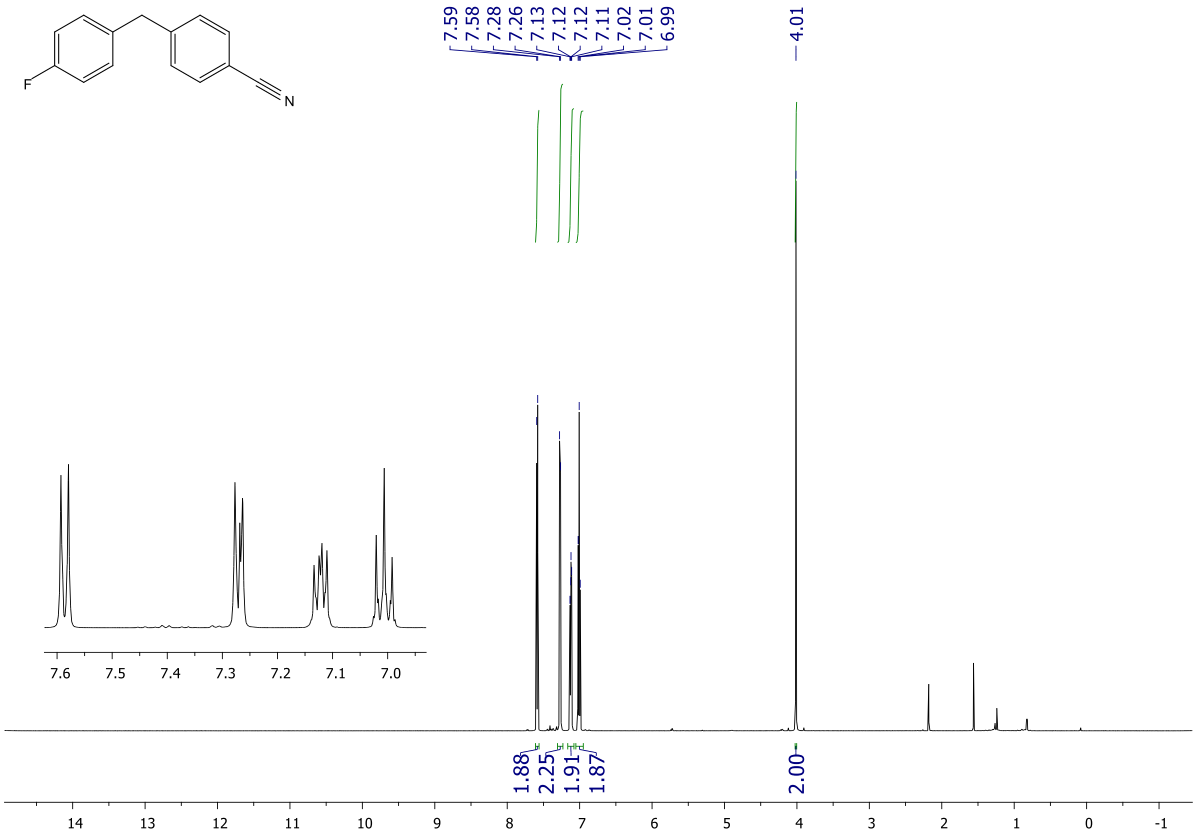

^{13}C-NMR, $CDCl_3$, 150 MHz

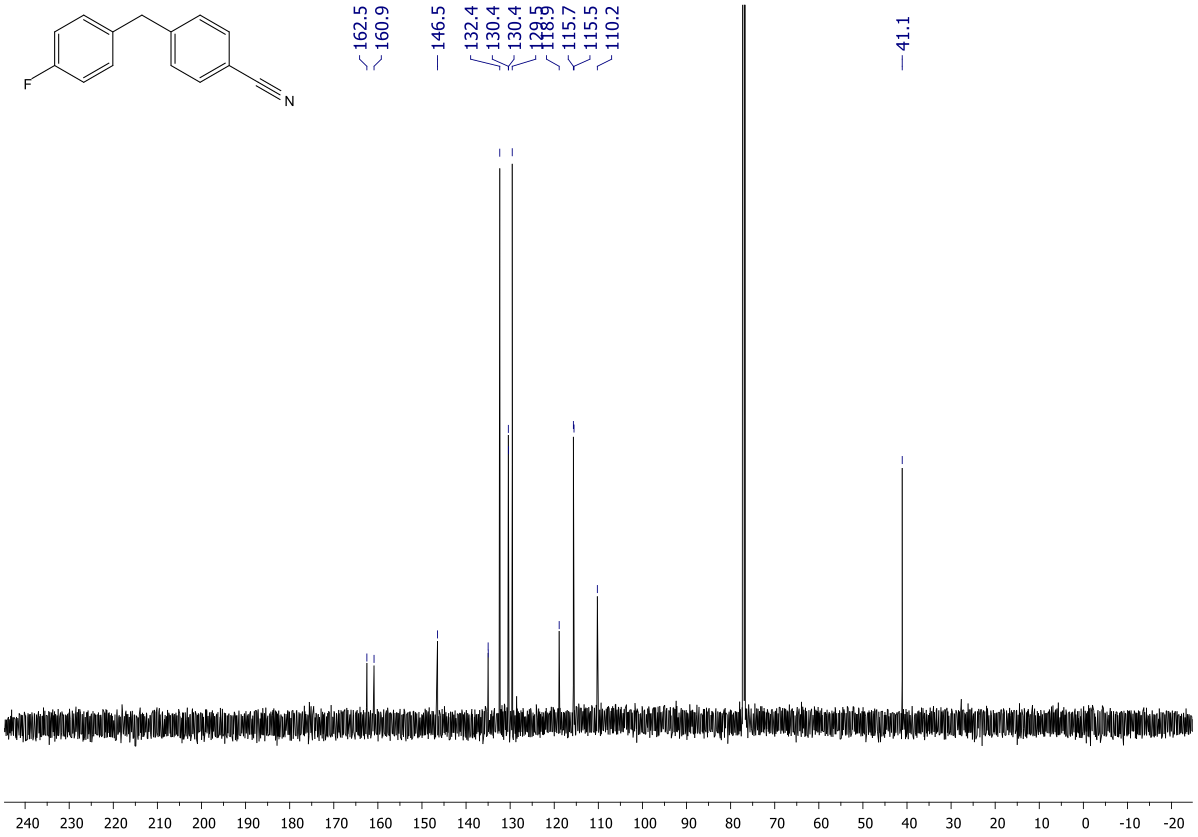

1-methoxy-4-(4-(trifluoromethyl)benzyl)benzene (3g)

^{1}H-NMR, $CDCl_3$, 600 MHz

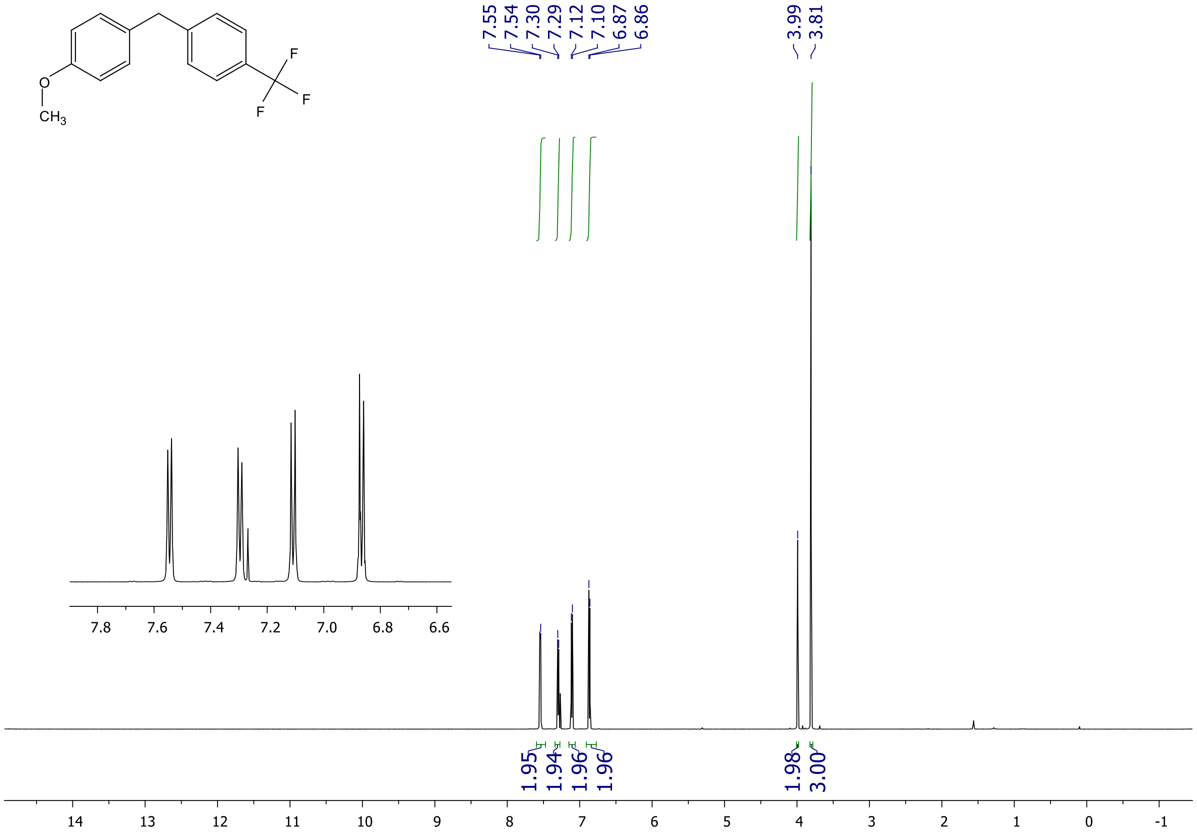

^{13}C-NMR, $CDCl_3$, 150 MHz

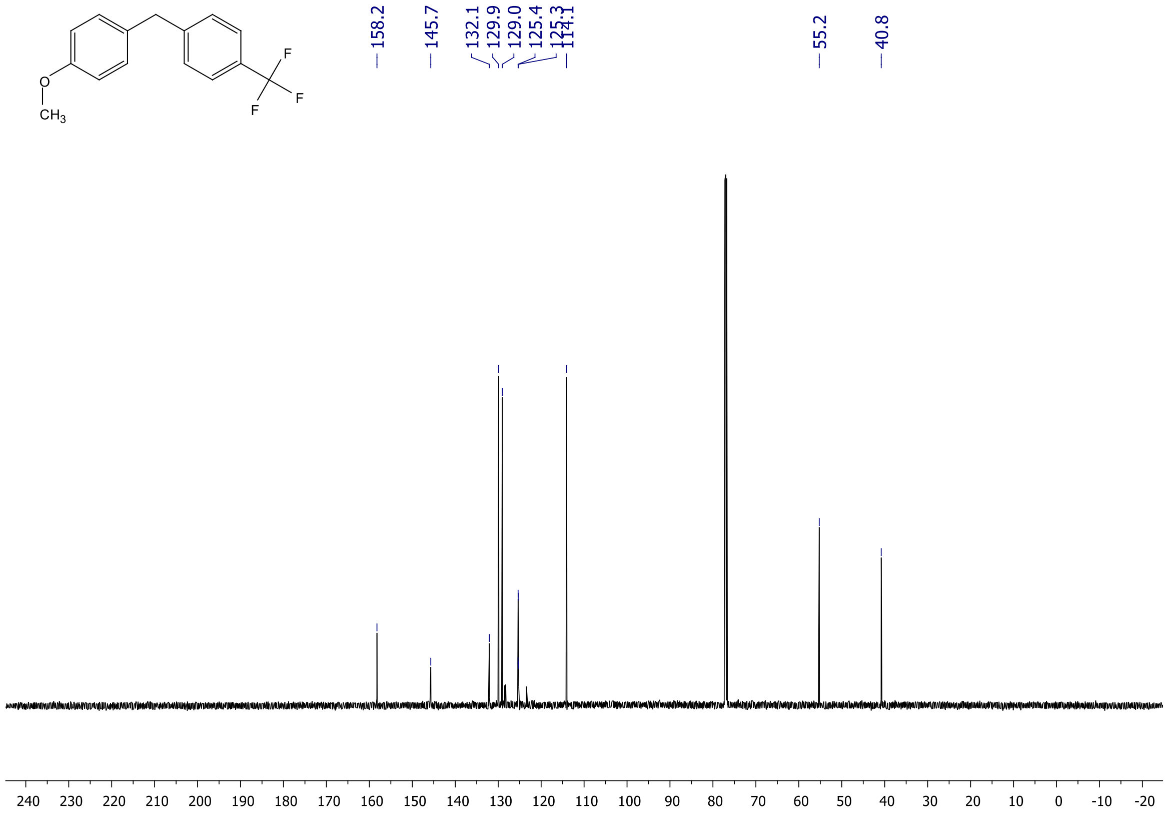

4-(4-methoxybenzyl)benzaldehyde (3h)

^{1}H-NMR, $CDCl_3$, 600 MHz

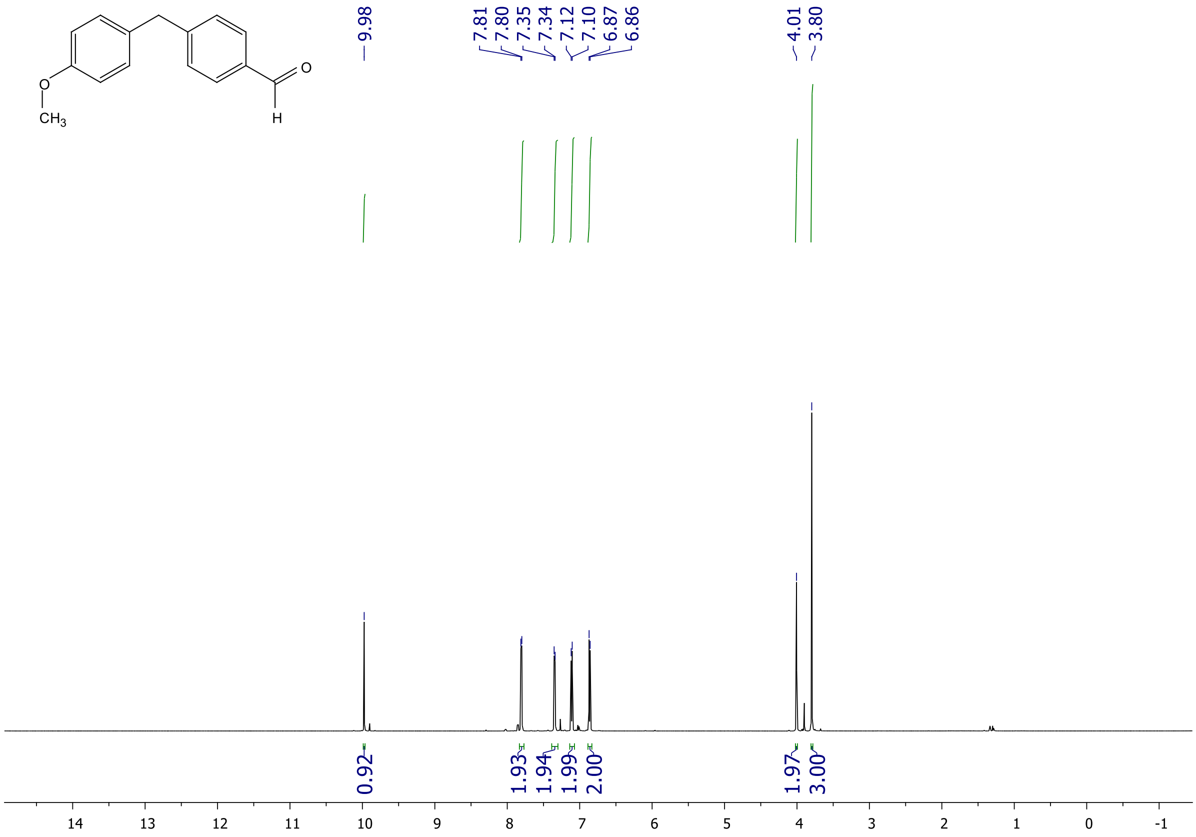

^{13}C-NMR, $CDCl_3$, 150 MHz

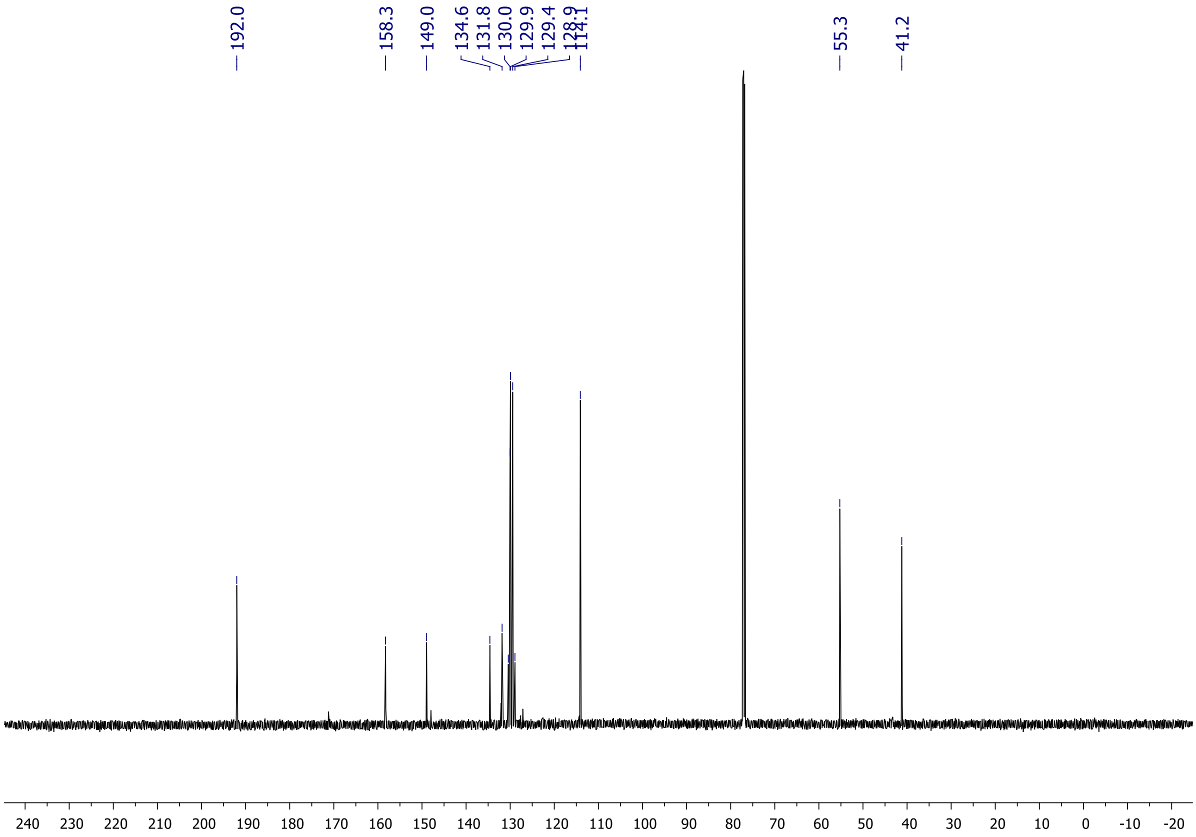

1-(4-methoxybenzyl)-2-methylbenzene (3i)

^{1}H-NMR, $CDCl_3$, 600 MHz

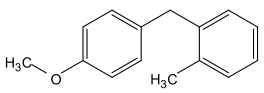

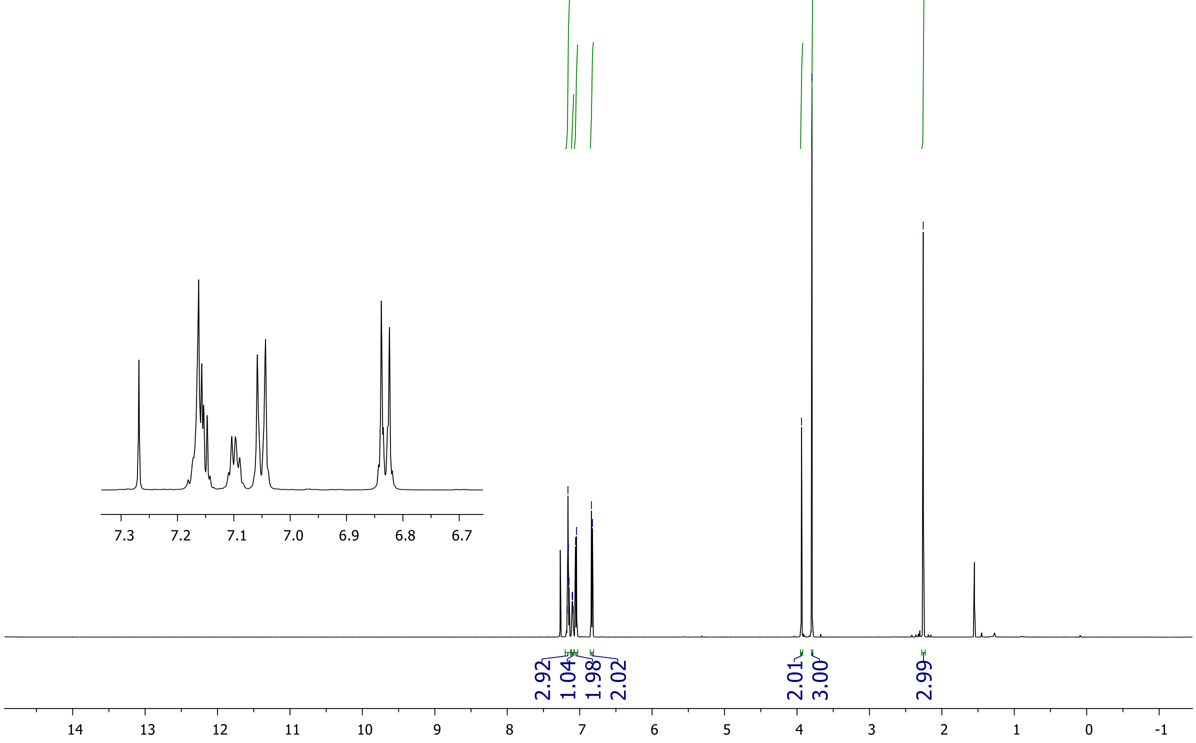

^{13}C-NMR, $CDCl_3$, 150 MHz

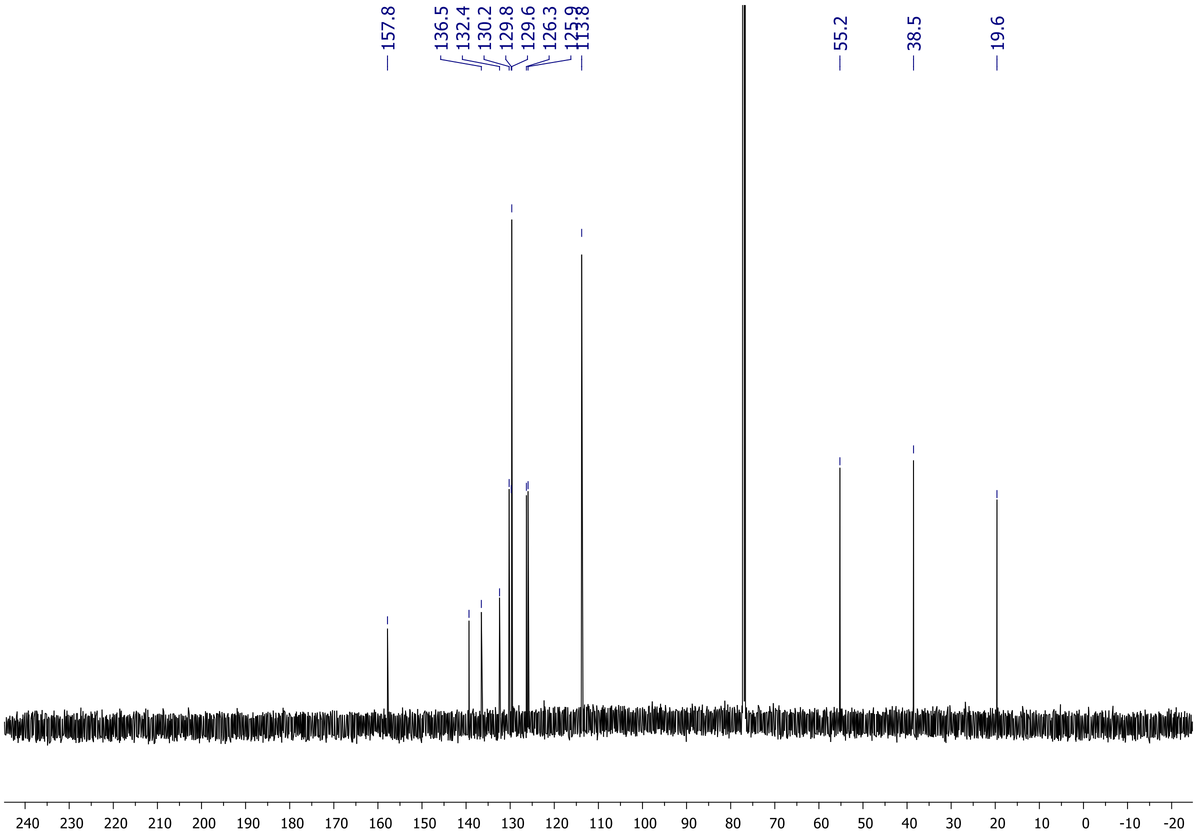

1-methoxy-4-(4-vinylbenzyl)benzene (3j)

^{1}H-NMR, $CDCl_3$, 600 MHz

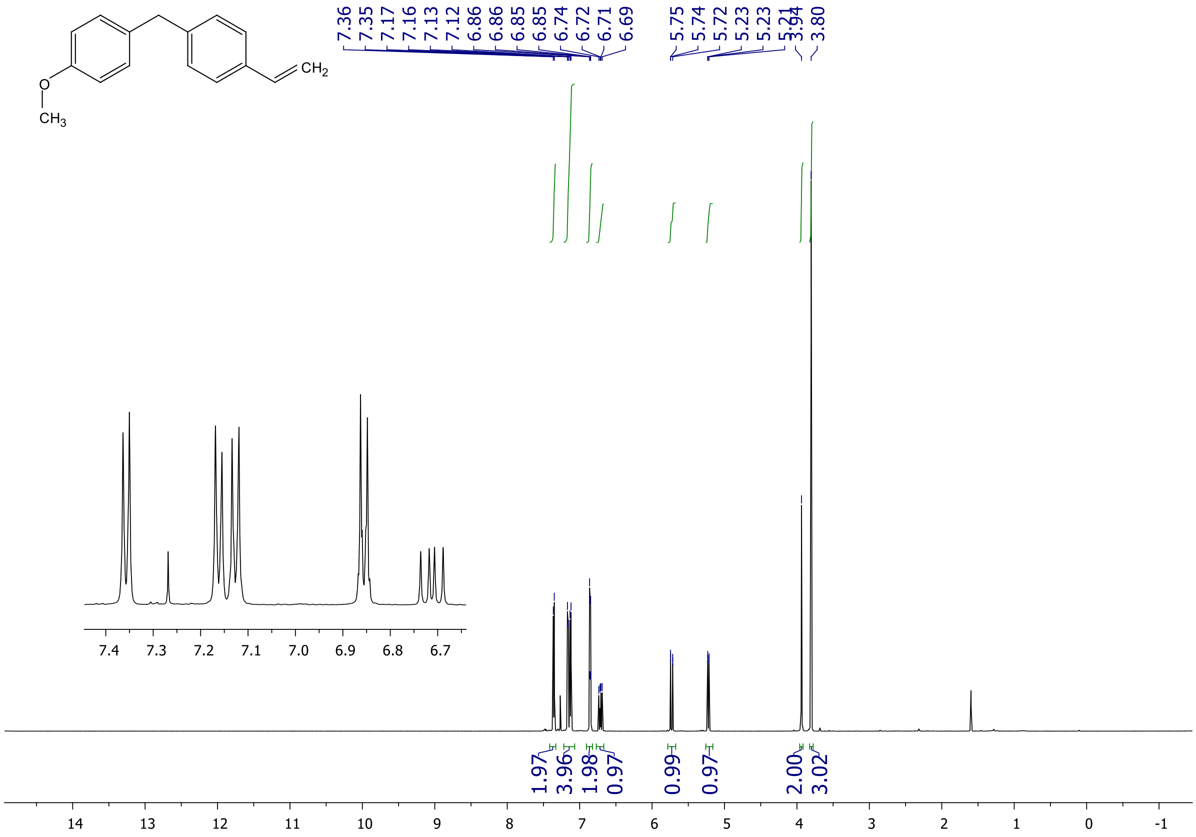

^{13}C-NMR, $CDCl_3$, 150 MHz

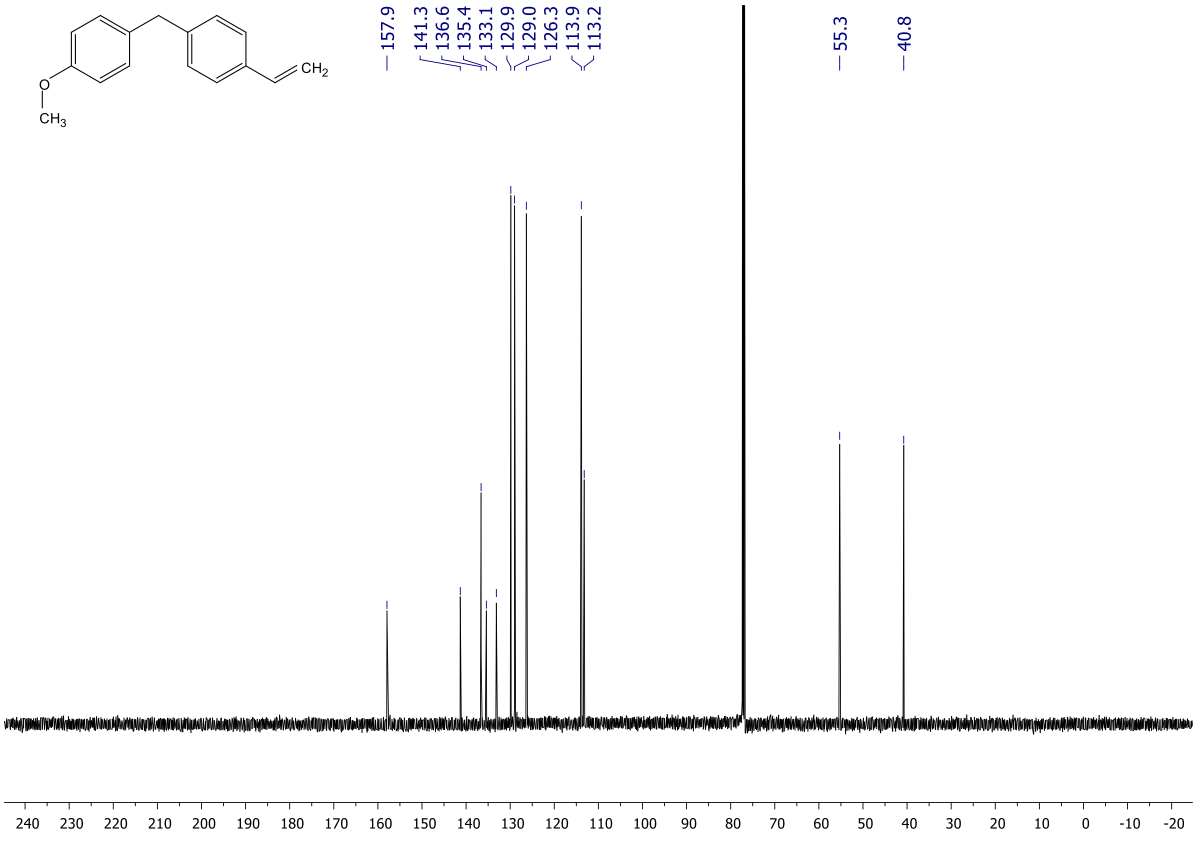

((4-(4-methoxybenzyl)phenyl)ethynyl)trimethylsilane (3k)

^{1}H-NMR, $CDCl_3$, 600 MHz

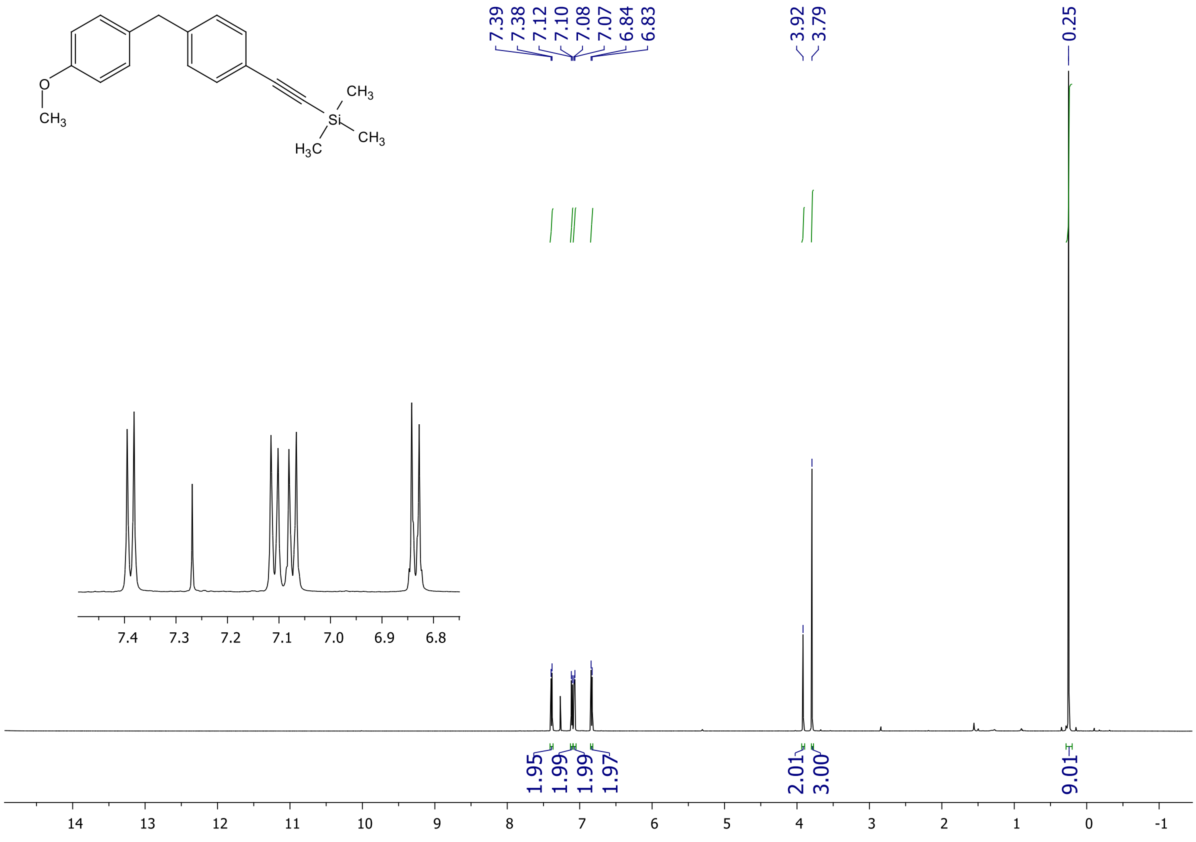

^{13}C-NMR, $CDCl_3$, 150 MHz

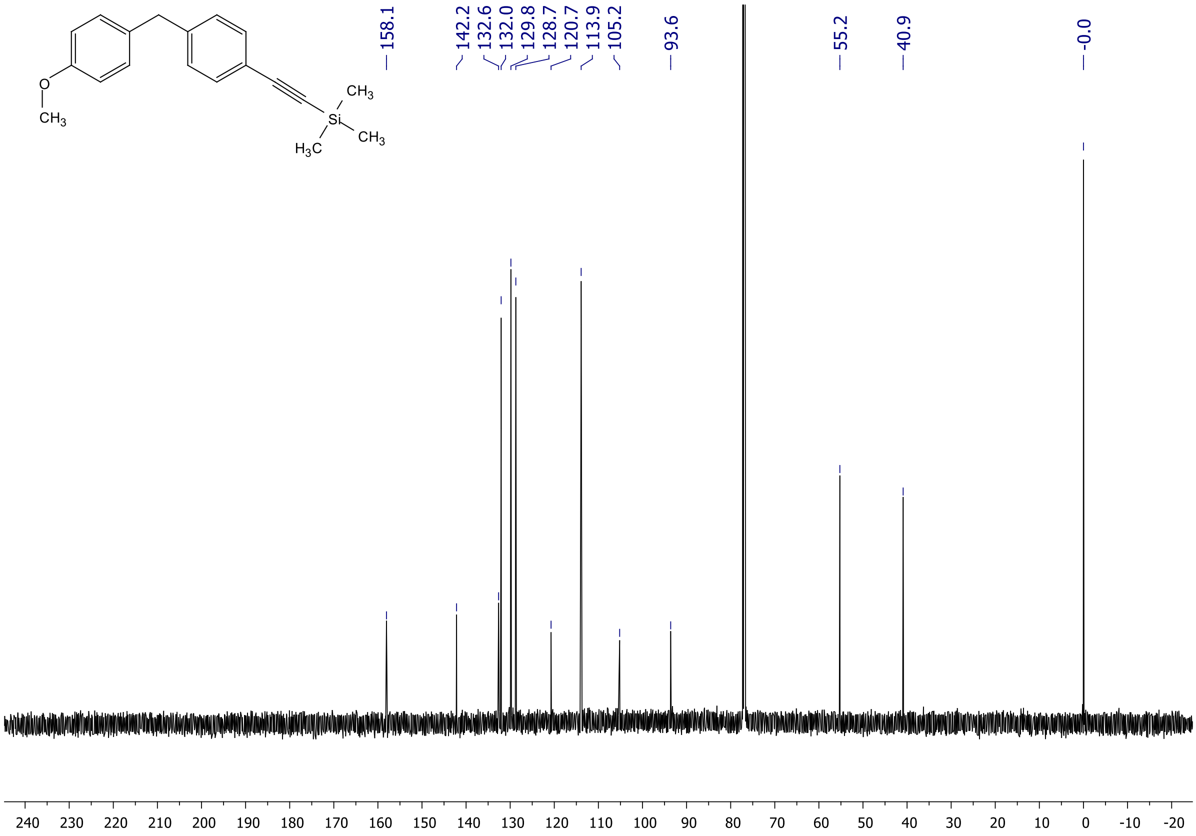

4-(4-methoxybenzyl)phenol (3l)

^{1}H-NMR, $CDCl_3$, 600 MHz

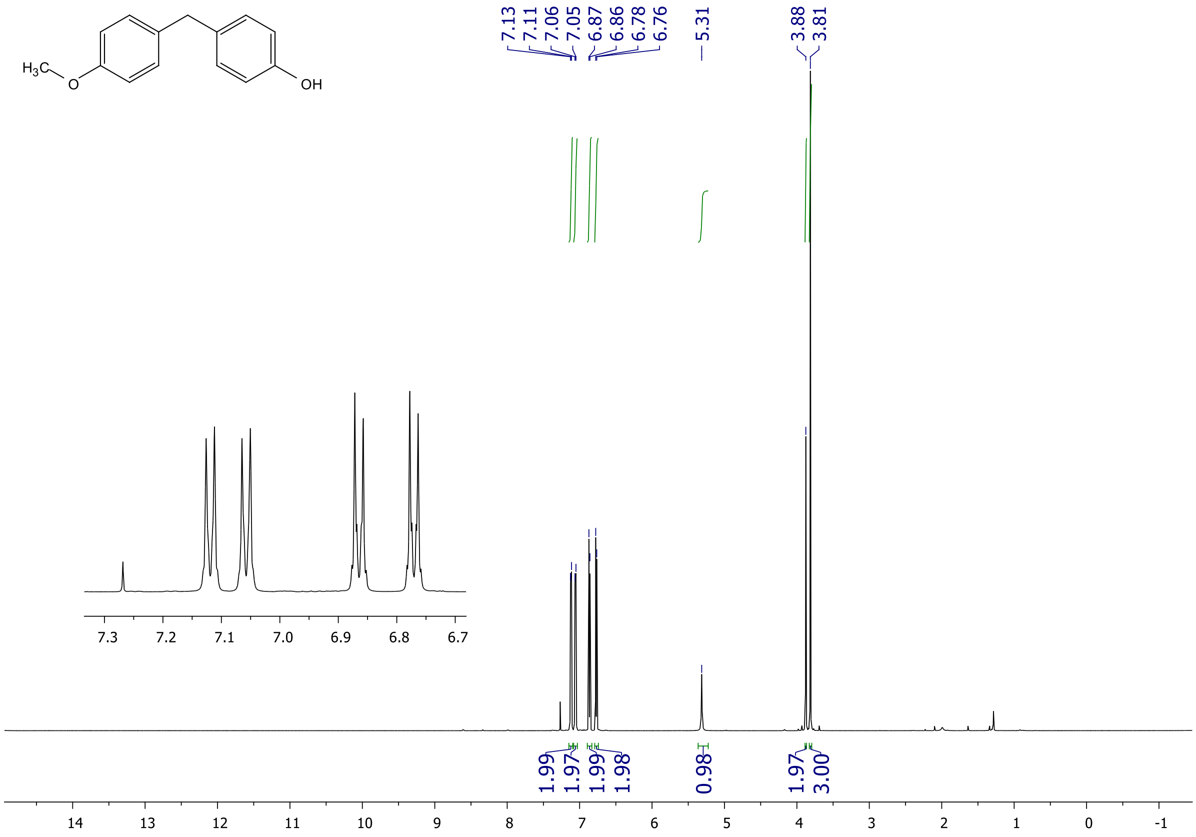

^{13}C-NMR, $CDCl_3$, 150 MHz

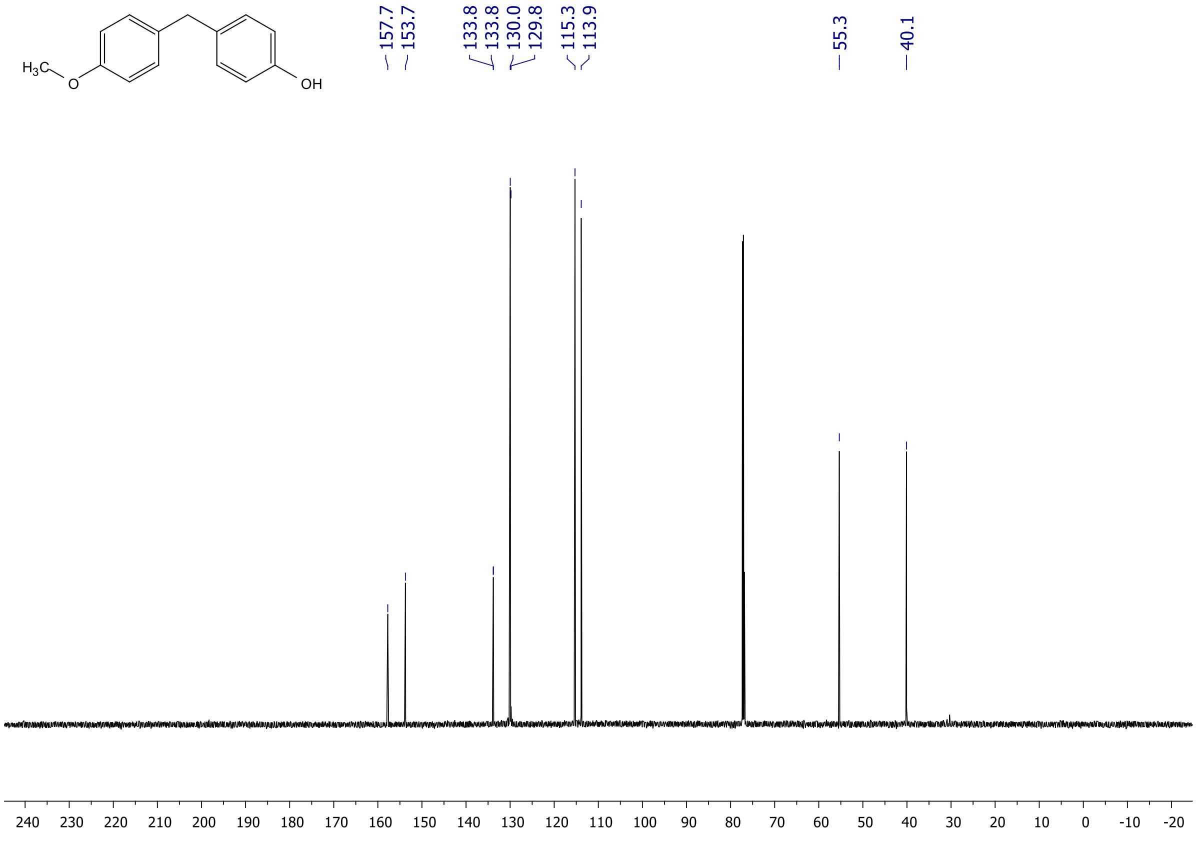

7.2.2 Beznzyl Pinacolborolanes Flow Arylation with cyanoarenes

4-(4-methoxybenzyl)pyridine (5a) :

^{1}H-NMR, $CDCl_3$, 600 MHz

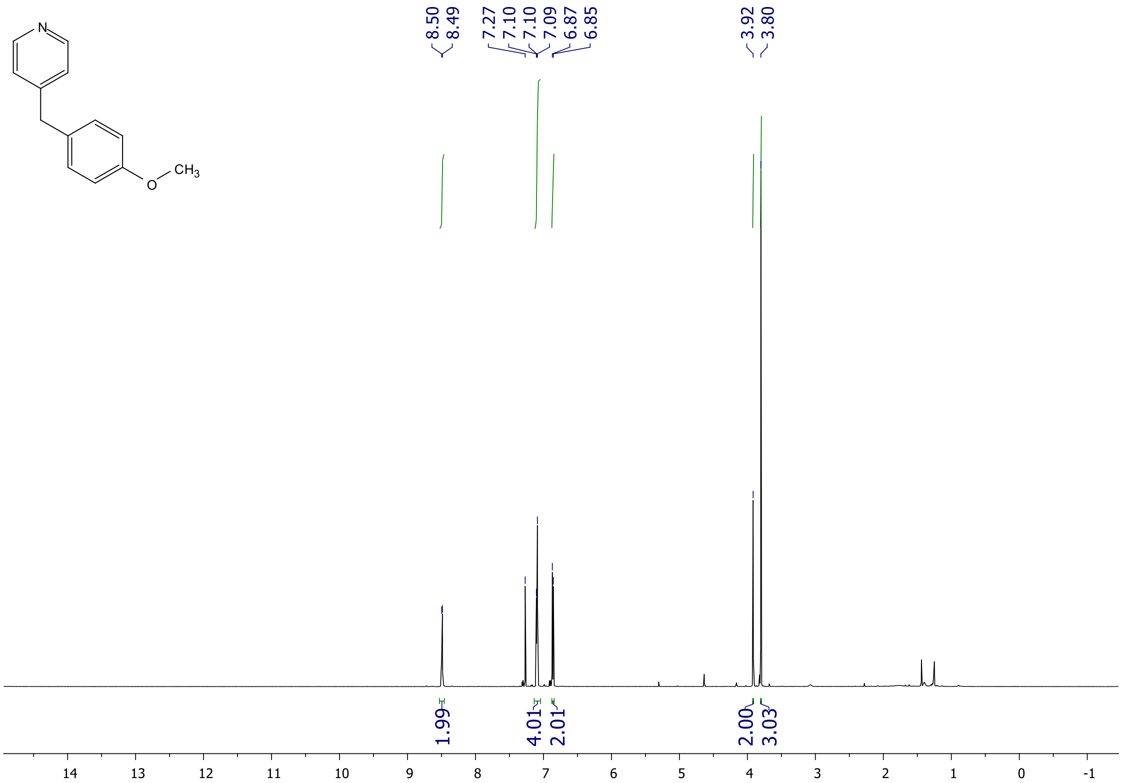

^{13}C-NMR, $CDCl_3$, 151 MHz

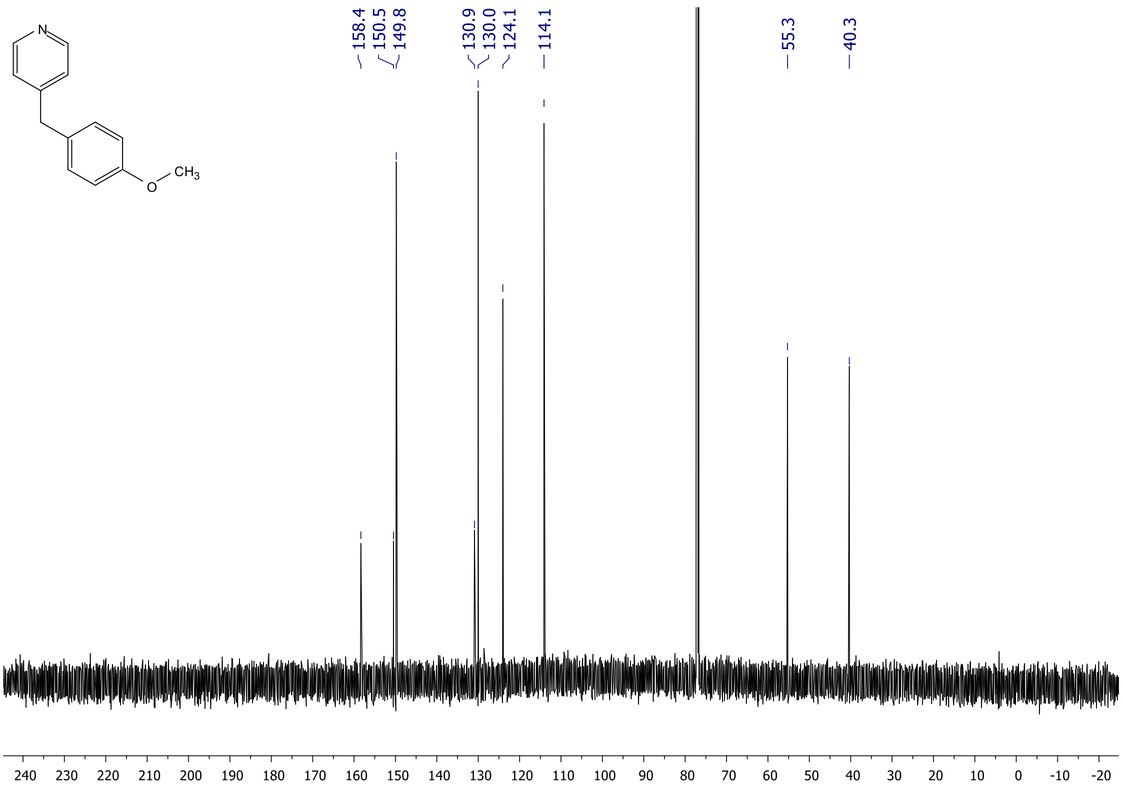

4-(4-methylbenzyl)pyridine (5b) :

^{1}H-NMR, $CDCl_3$, 600 MHz

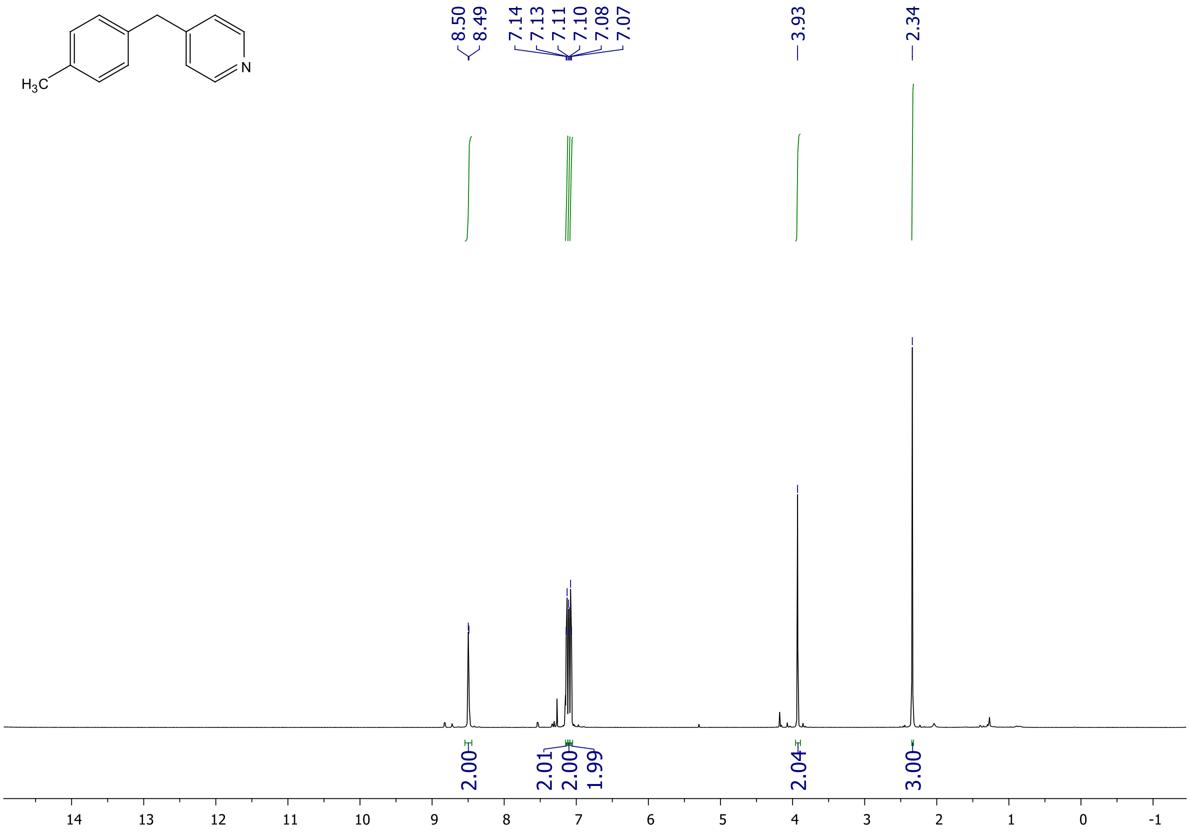

^{13}C-NMR, $CDCl_3$, 151 MHz

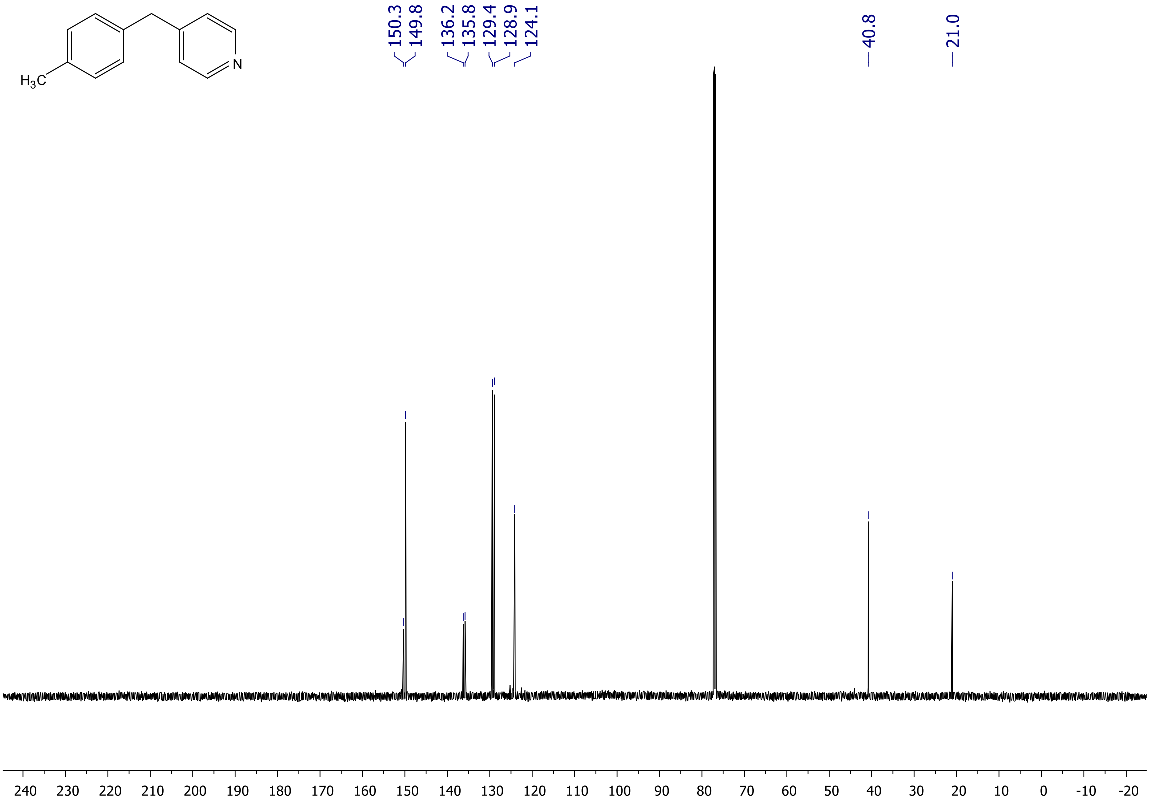

4-benzylpyridine (5c):

^{1}H-NMR, $CDCl_3$, 600 MHz

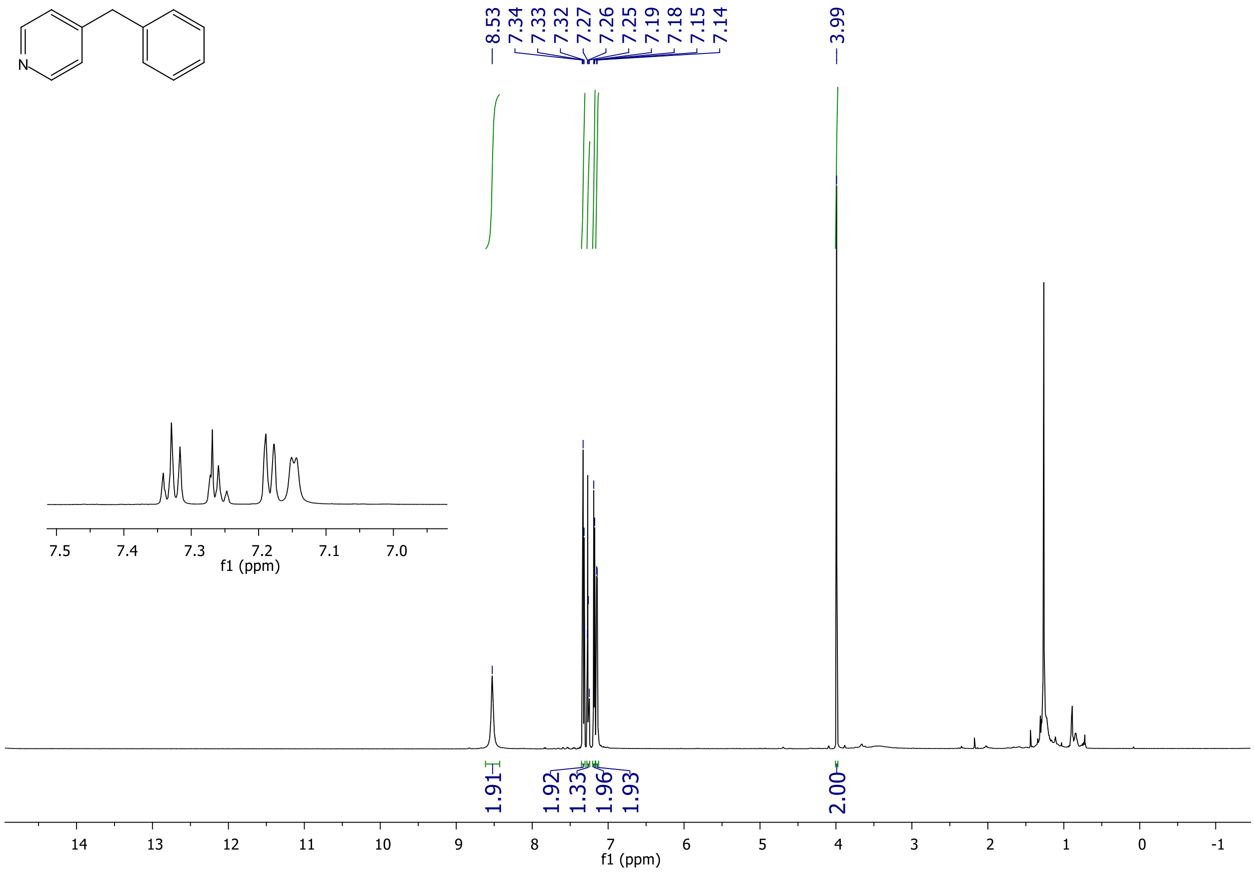

^{13}C-NMR, $CDCl_3$, 150 MHz

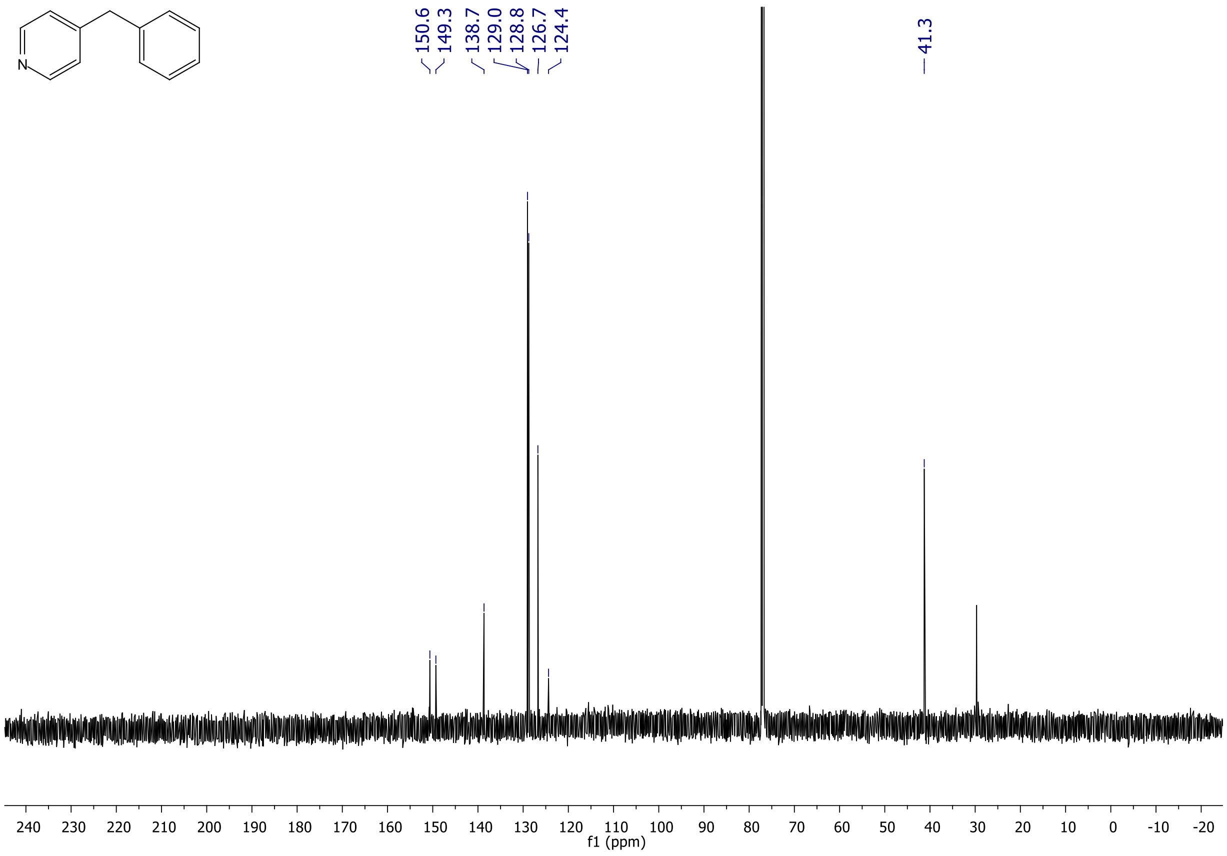

4-(1-(4-methoxyphenyl)ethyl)pyridine (5d)

^{1}H-NMR, $CDCl_3$, 600 MHz

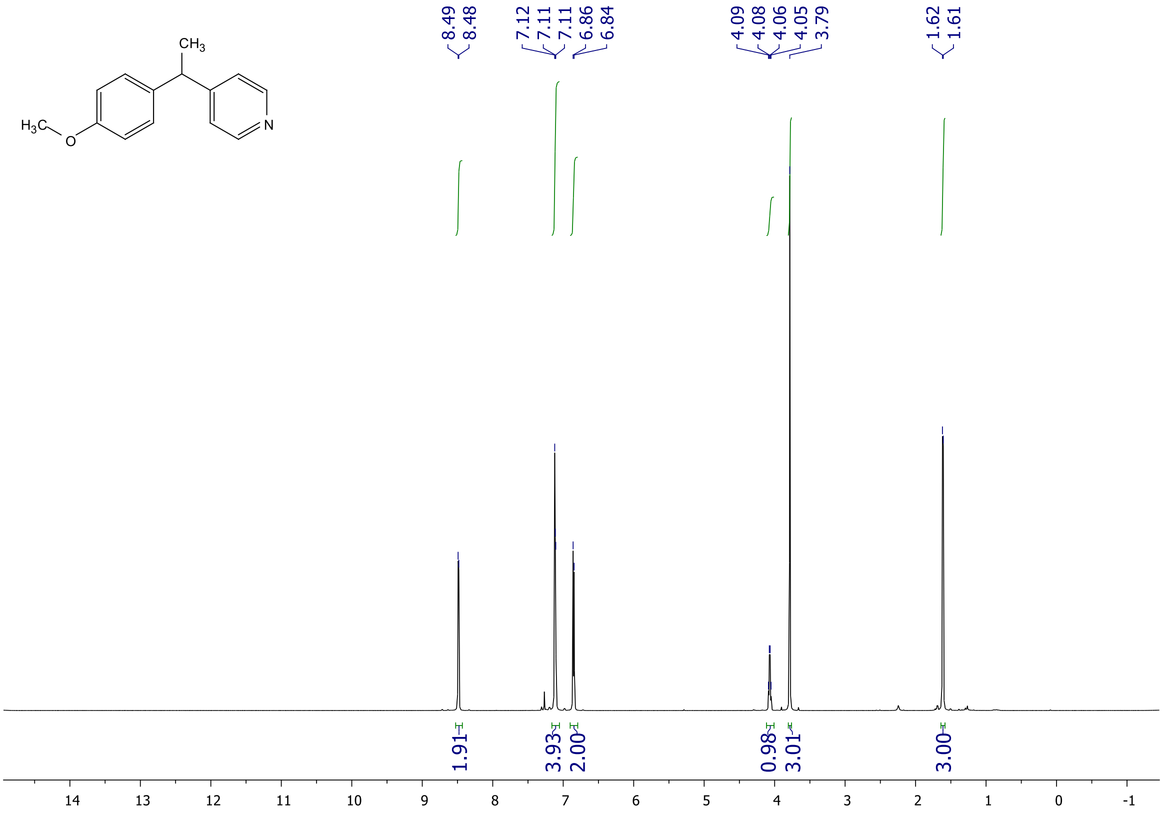

^{13}C-NMR, $CDCl_3$, 151 MHz

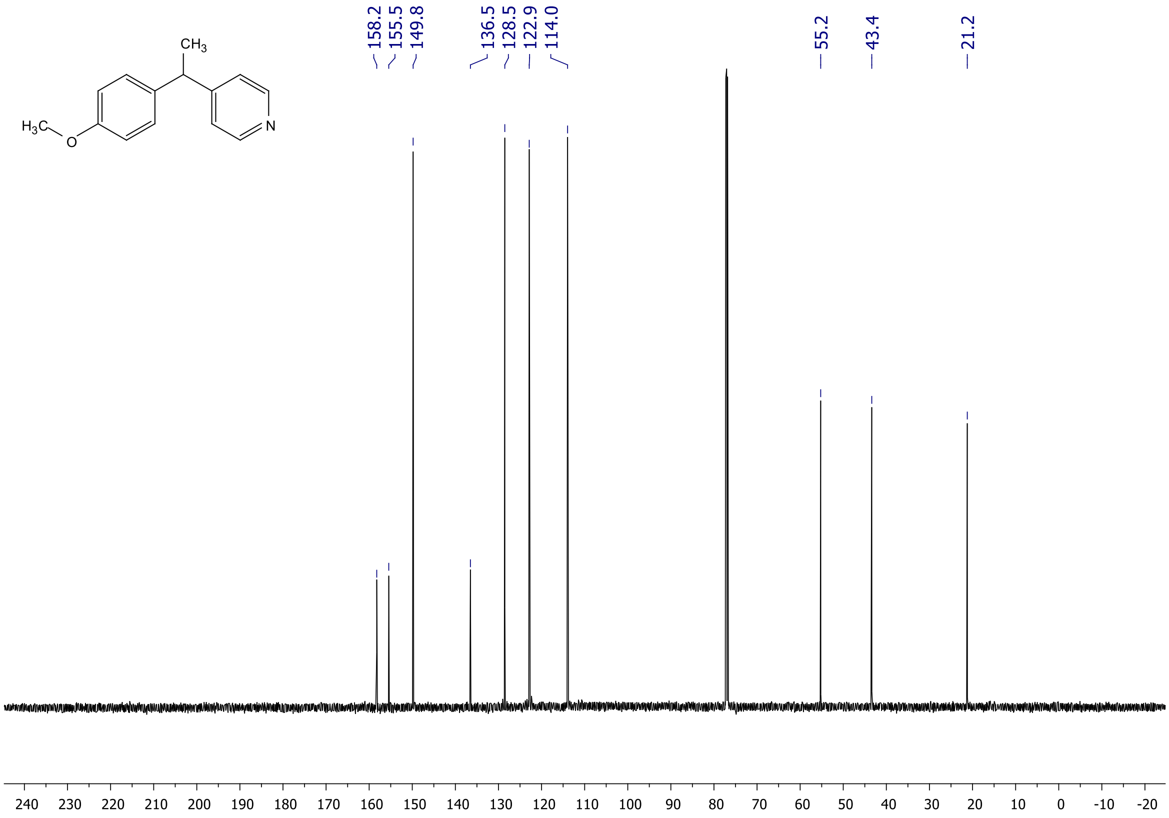

1-(4-methoxybenzyl)isoquinoline (5e)

^{1}H-NMR, $CDCl_3$, 600 MHz

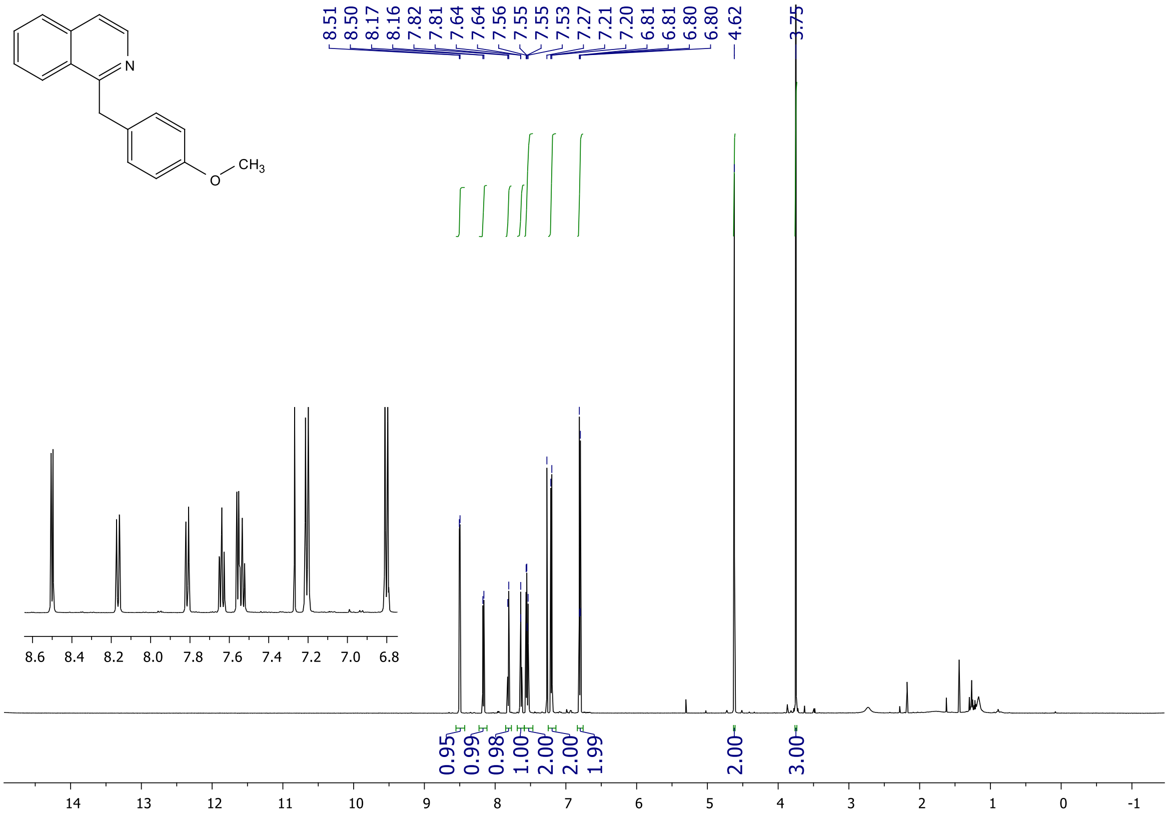

^{13}C-NMR, $CDCl_3$, 151 MHz

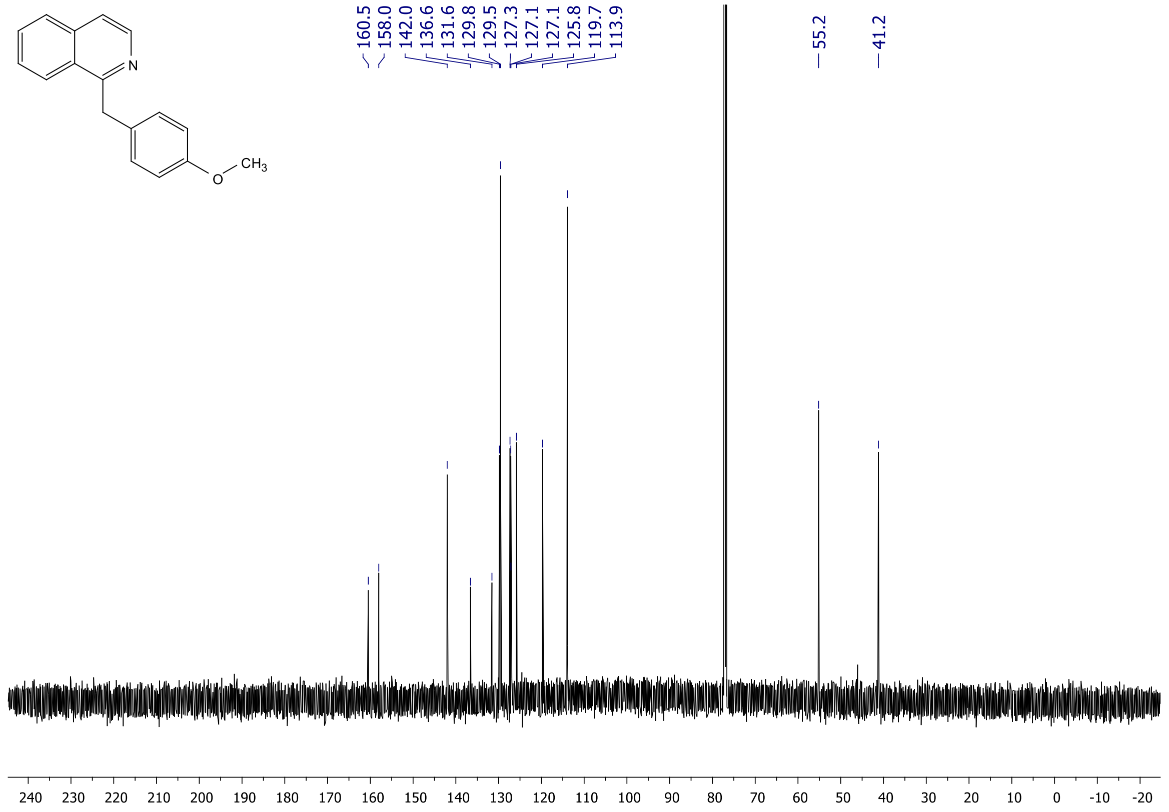

1-(1-phenylethyl)isoquinoline (5f):

^{1}H-NMR, $CDCl_3$, 600 MHz

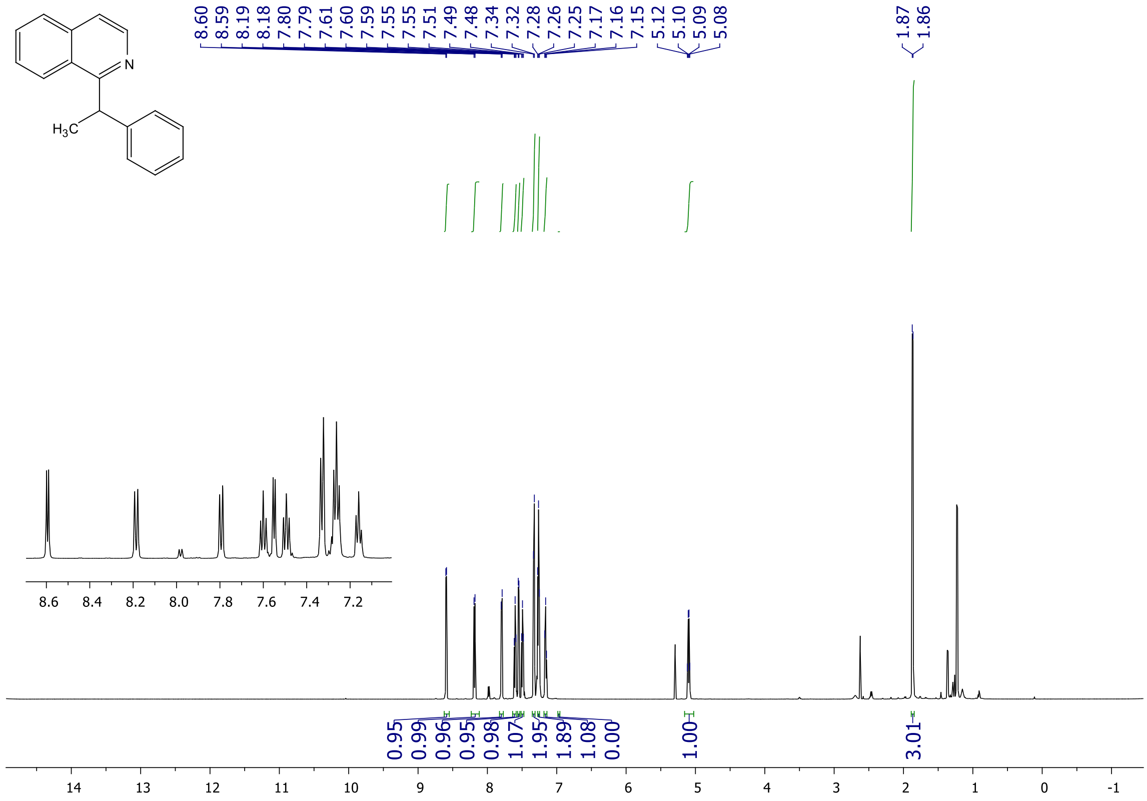

^{13}C-NMR, $CDCl_3$, 151 MHz

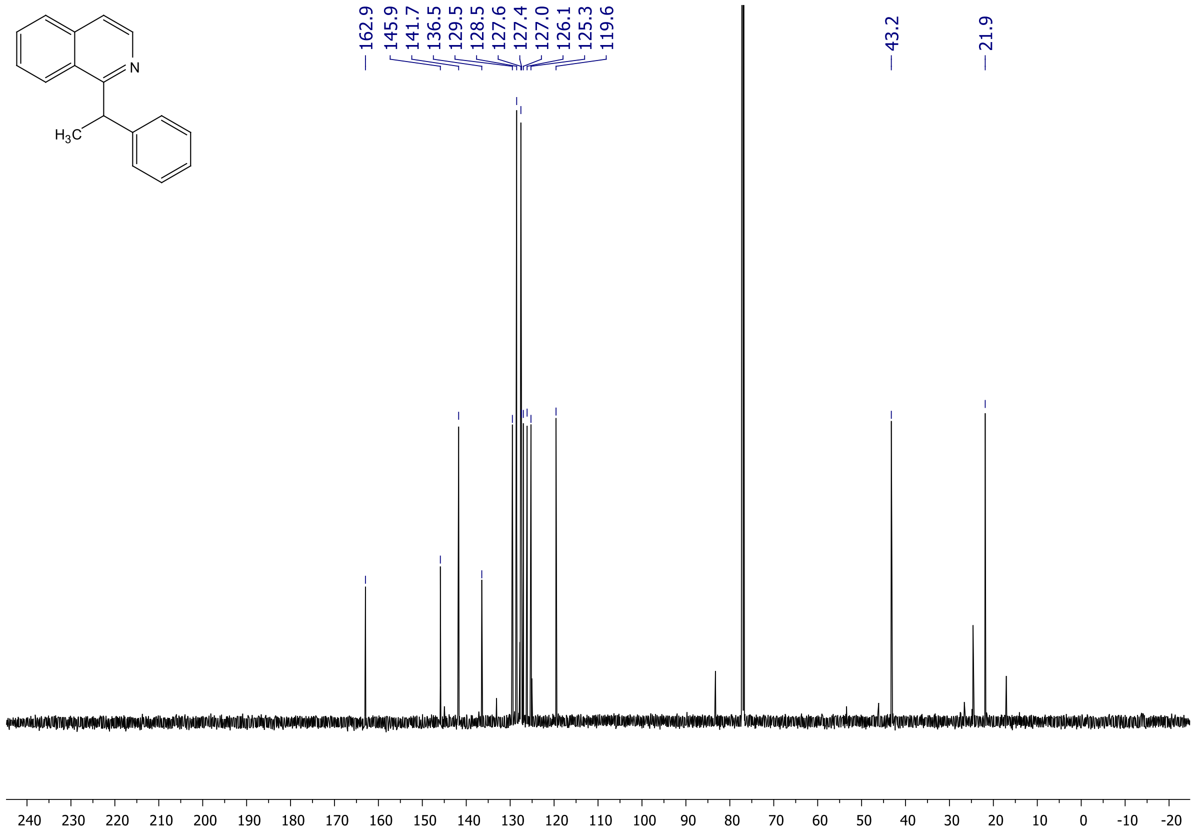

1-(4-methylbenzyl)isoquinoline (5g)

^{1}H-NMR, $CDCl_3$, 600 MHz

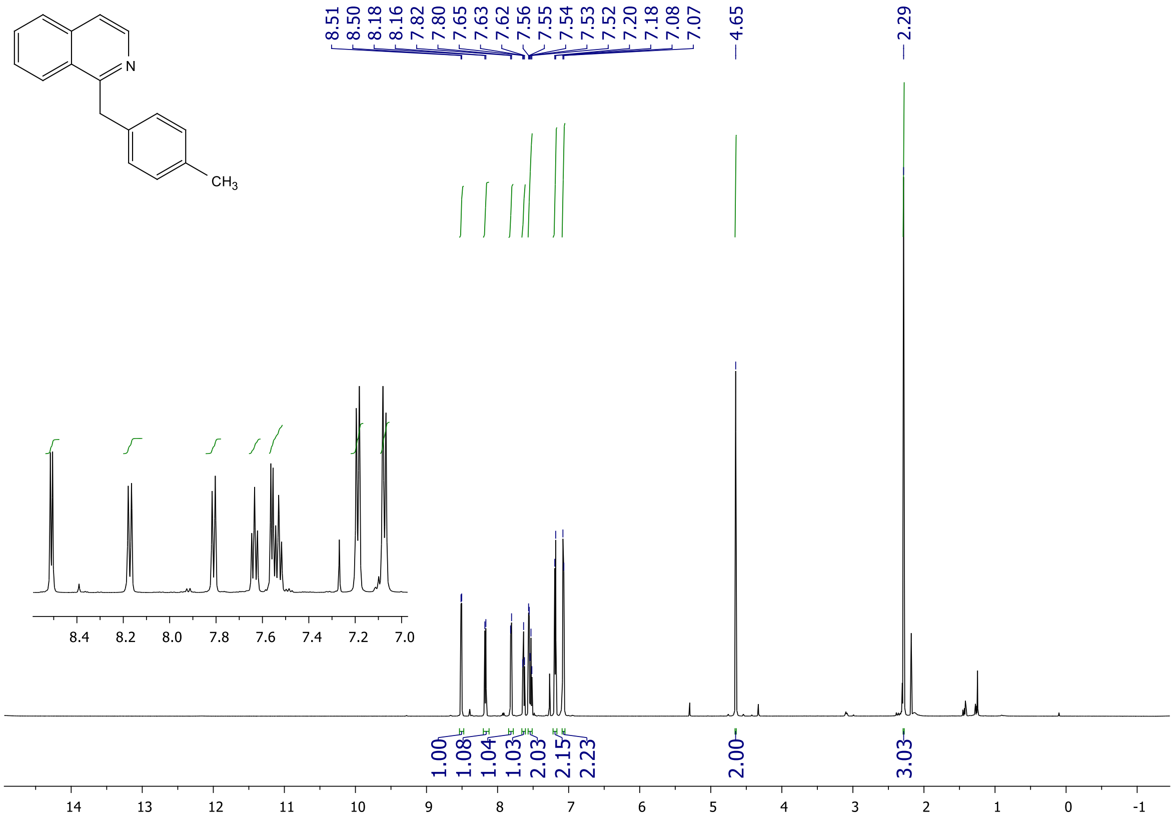

^{13}C-NMR, $CDCl_3$, 151 MHz

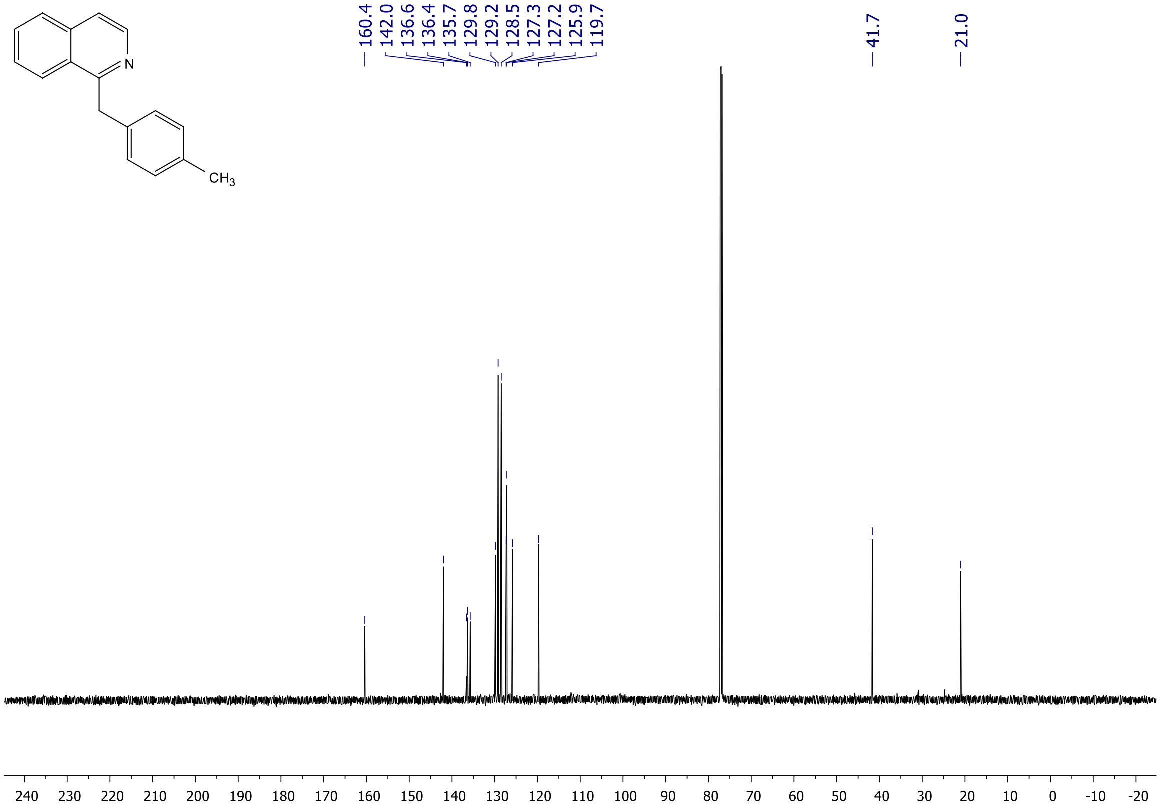

1-benzhydrylisoquinoline (5h)

^{1}H-NMR, $CDCl_3$, 600 MHz

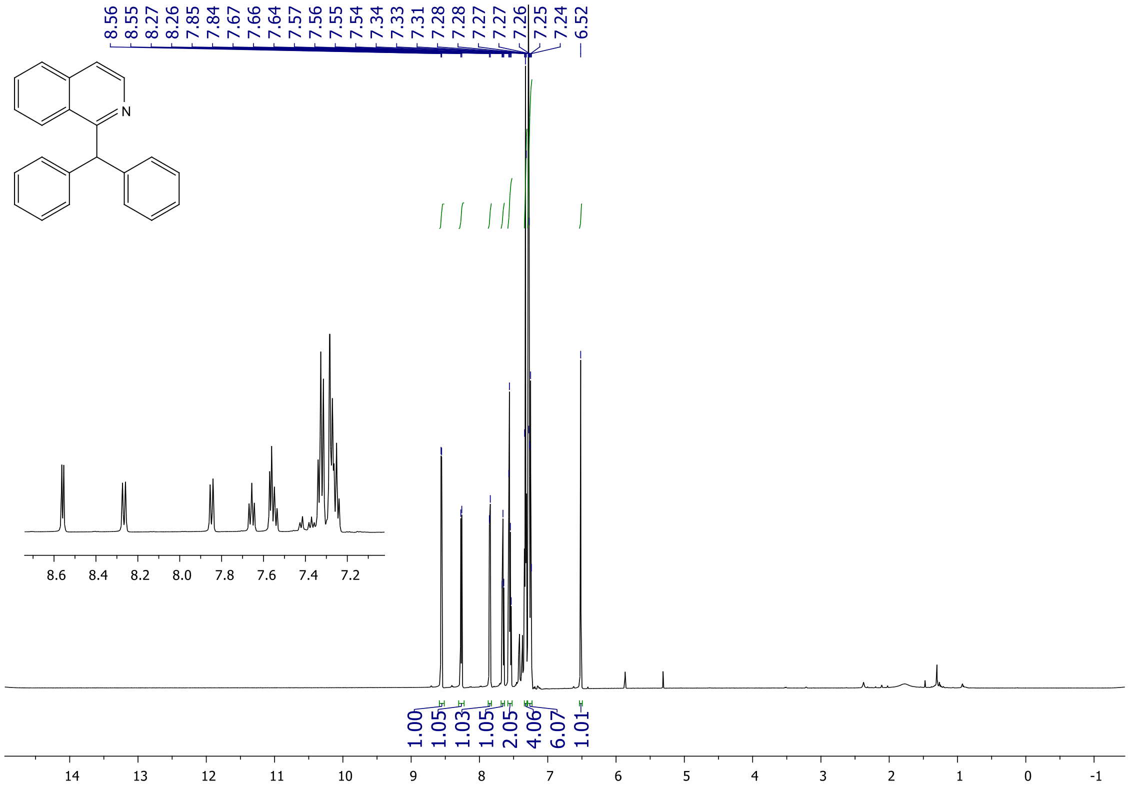

^{13}C-NMR, $CDCl_3$, 151 MHz

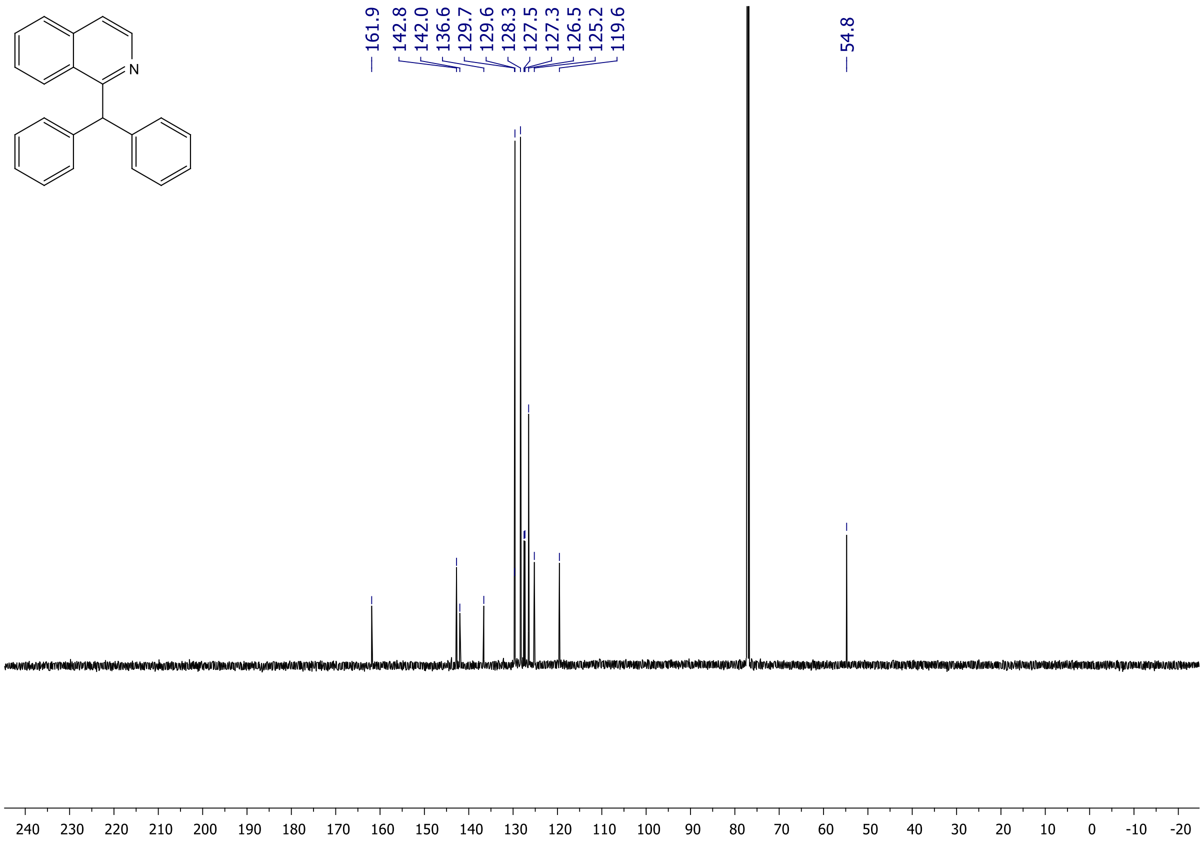

1-(4-fluorobenzyl)isoquinoline (5i)

^{1}H-NMR, $CDCl_3$, 600 MHz

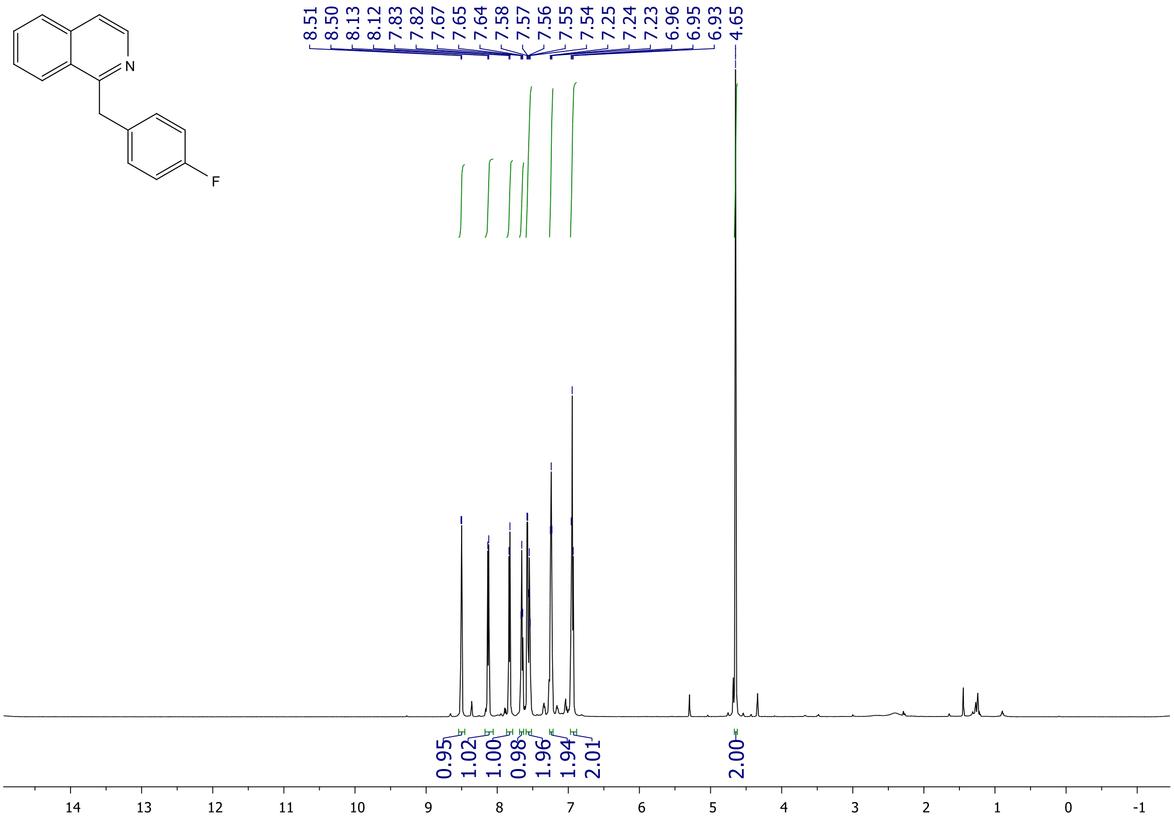

^{13}C-NMR, $CDCl_3$, 151 MHz

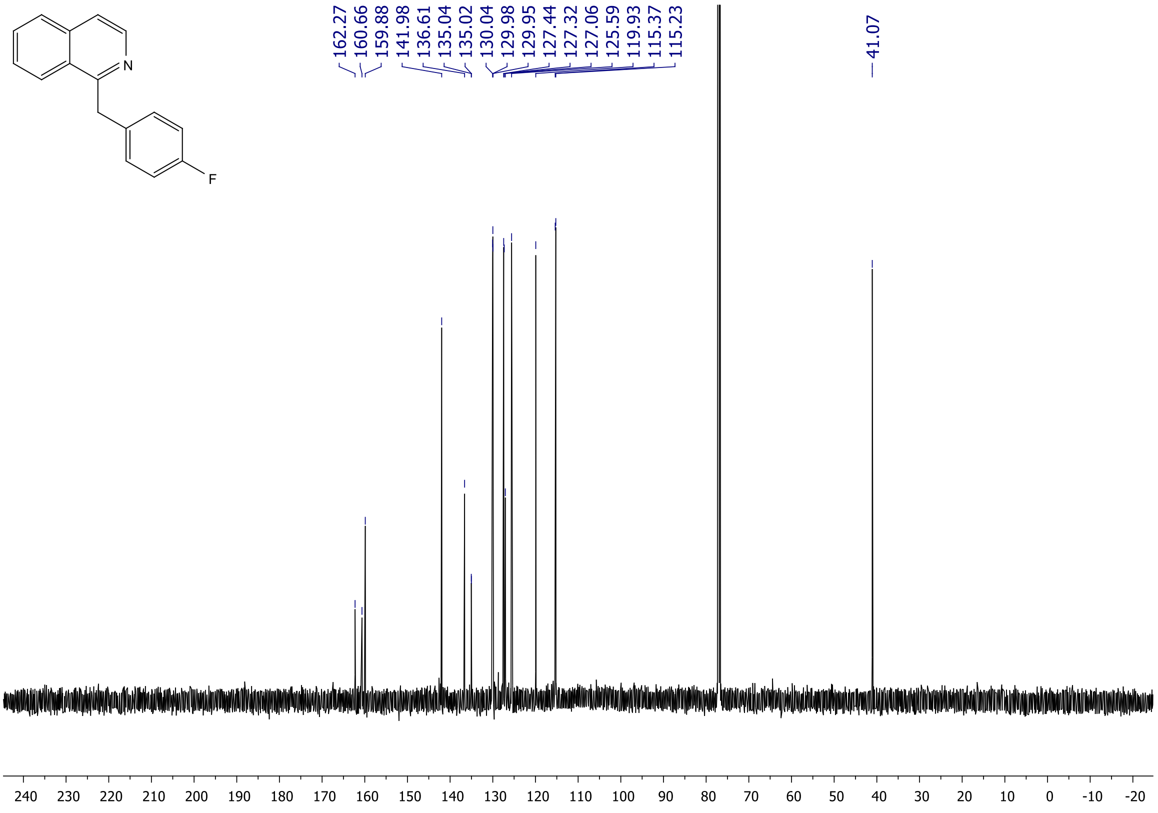

1-(3,5-bis(trifluoromethyl)benzyl)isoquinoline (5j)

^{1}H-NMR, $CDCl_3$, 600 MHz

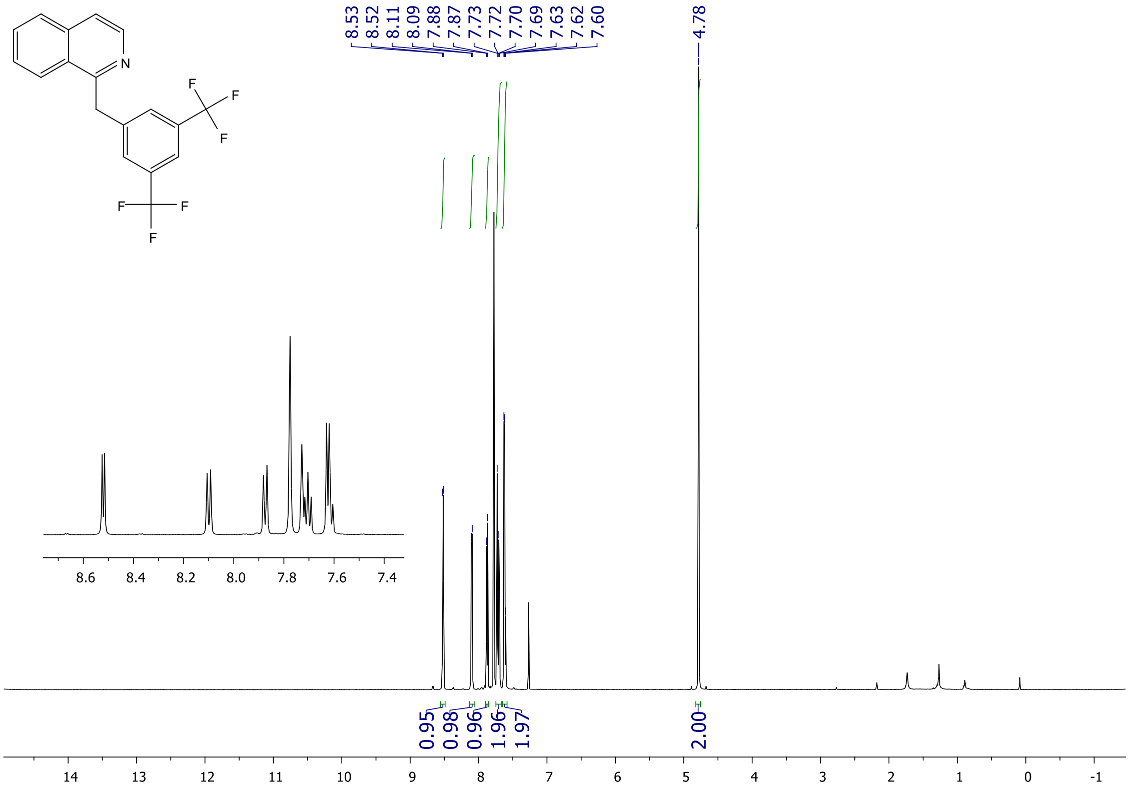

^{13}C-NMR, $CDCl_3$, 151 MHz

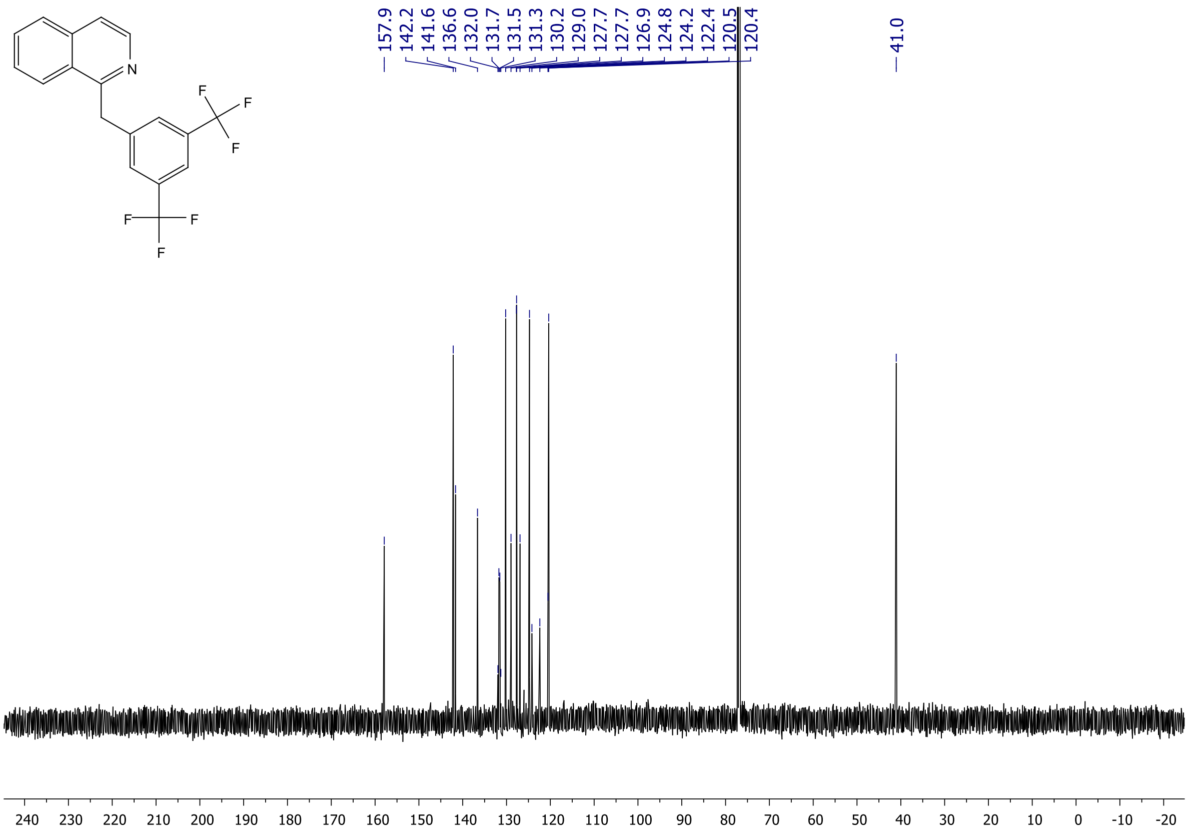

1-(2,6-difluorobenzyl)isoquinoline (5k):

^{1}H-NMR, $CDCl_3$, 600 MHz

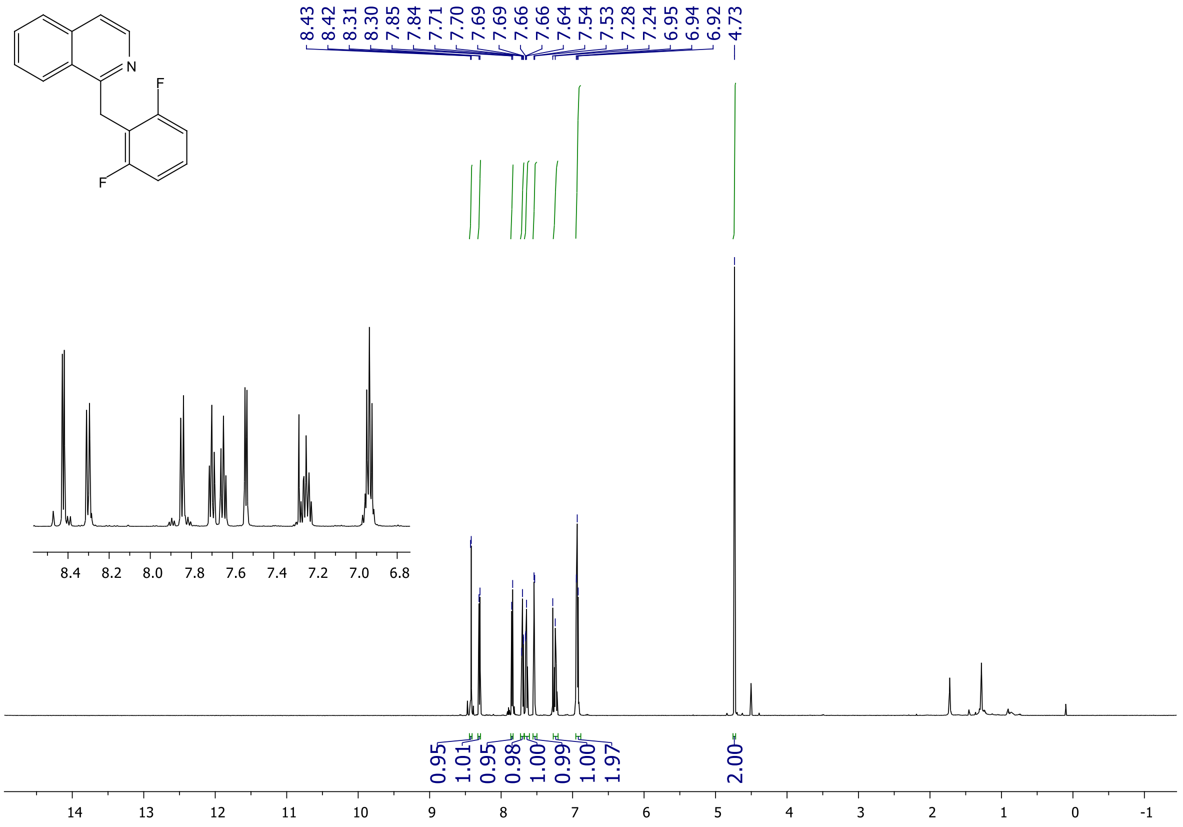

^{13}C-NMR, $CDCl_3$, 151 MHz

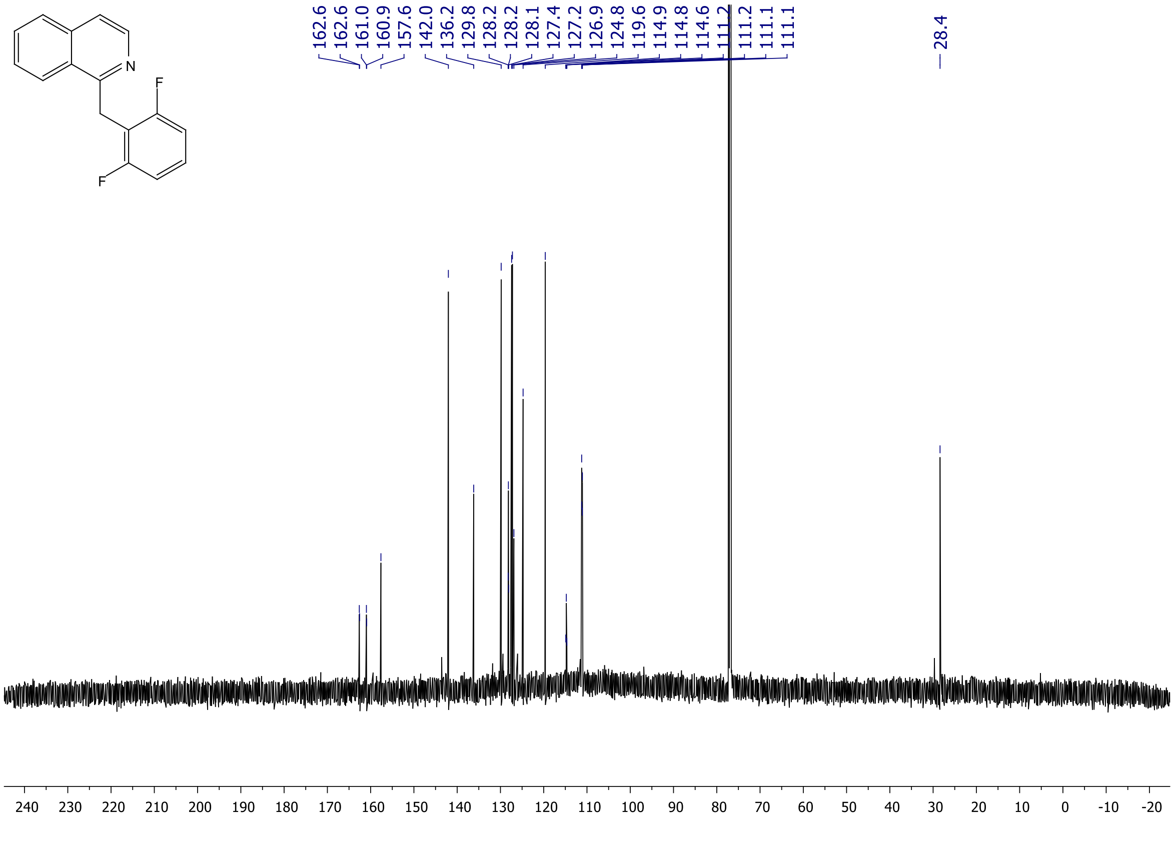

^{19}F-NMR, $CDCl_3$, 376 MHz

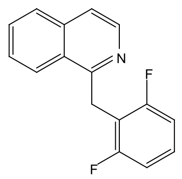

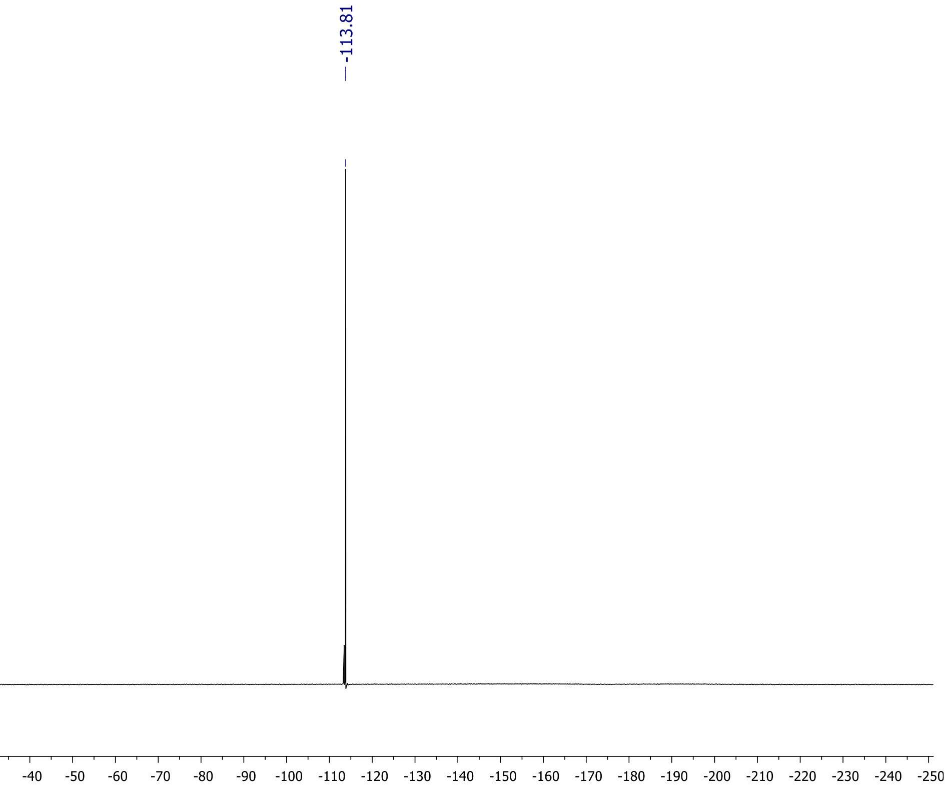

1-(3,5-dimethoxybenzyl)isoquinoline (5l)

^{1}H-NMR, $CDCl_3$, 600 MHz

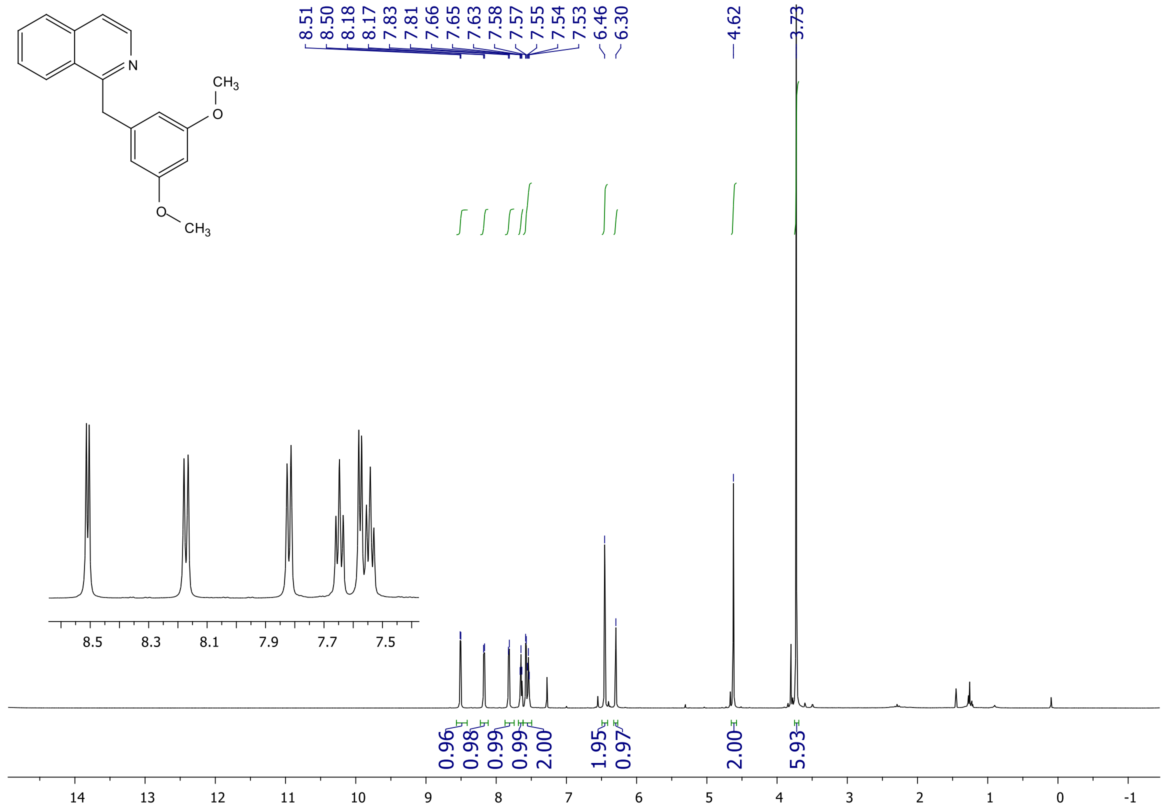

^{13}C-NMR, $CDCl_3$, 151 MHz

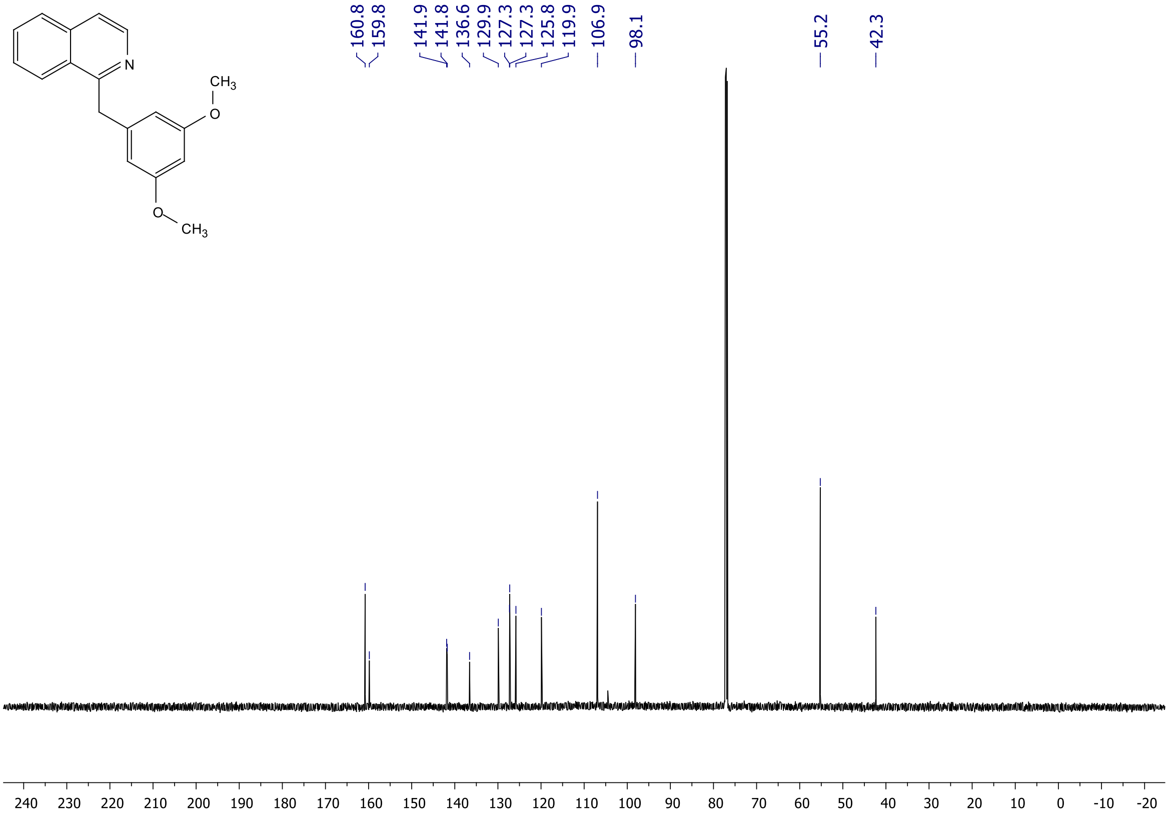

4-(4-methoxybenzyl)-1H-pyrrolo[2,3-b]pyridine (5m)

^{1}H-NMR, $CDCl_3$, 600 MHz

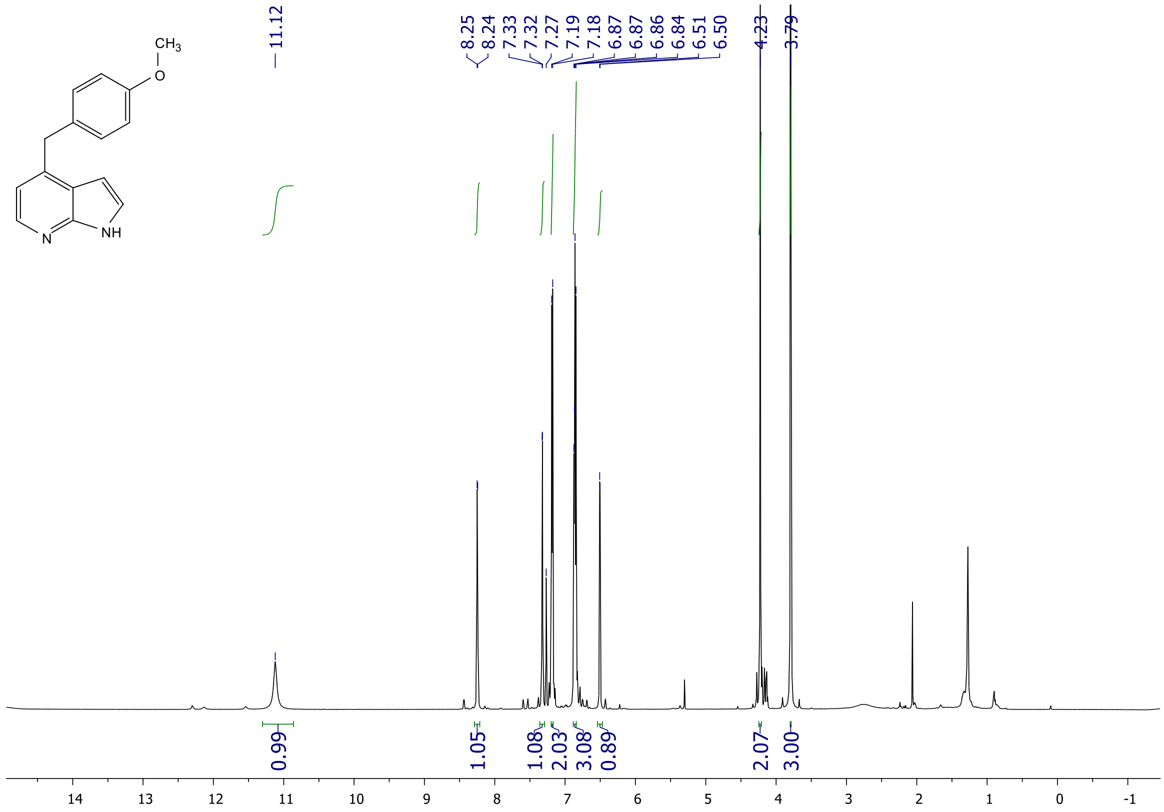

^{13}C-NMR, $CDCl_3$, 151 MHz

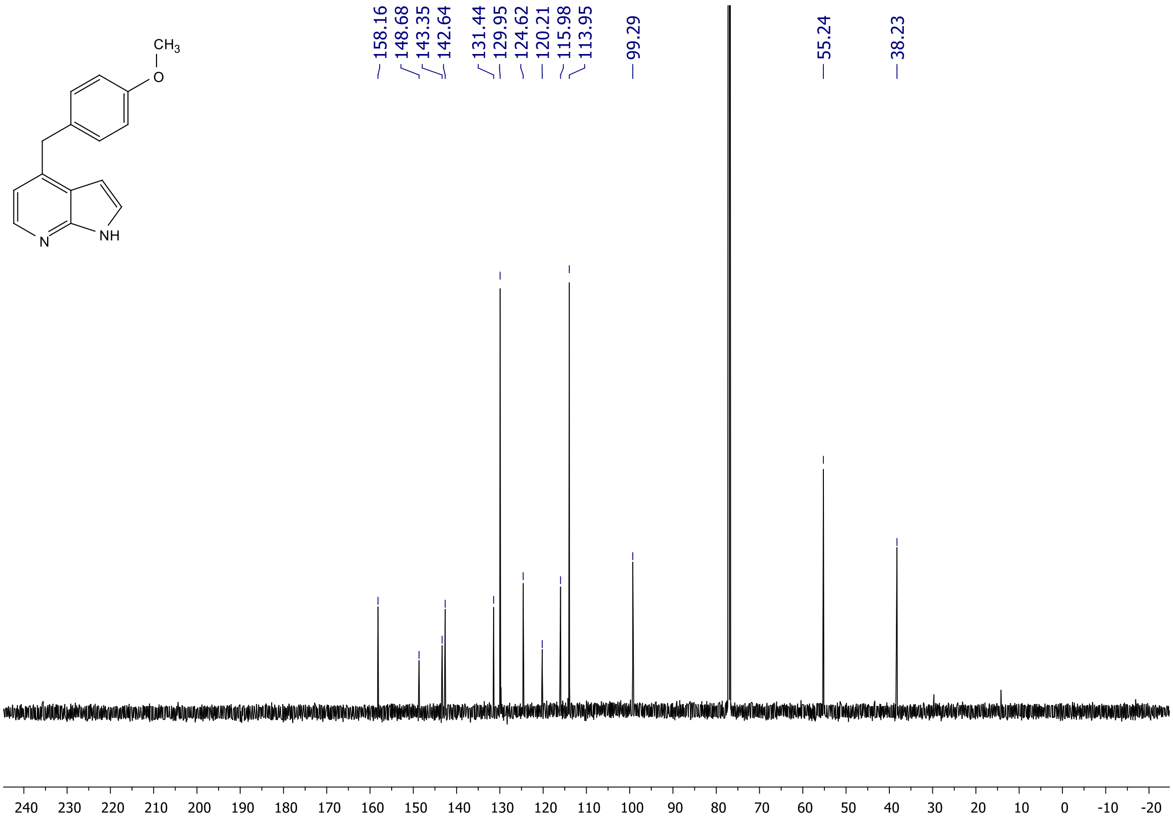

4-(4-methoxybenzyl)quinoline (5n)

^{1}H-NMR, $CDCl_3$, 600 MHz

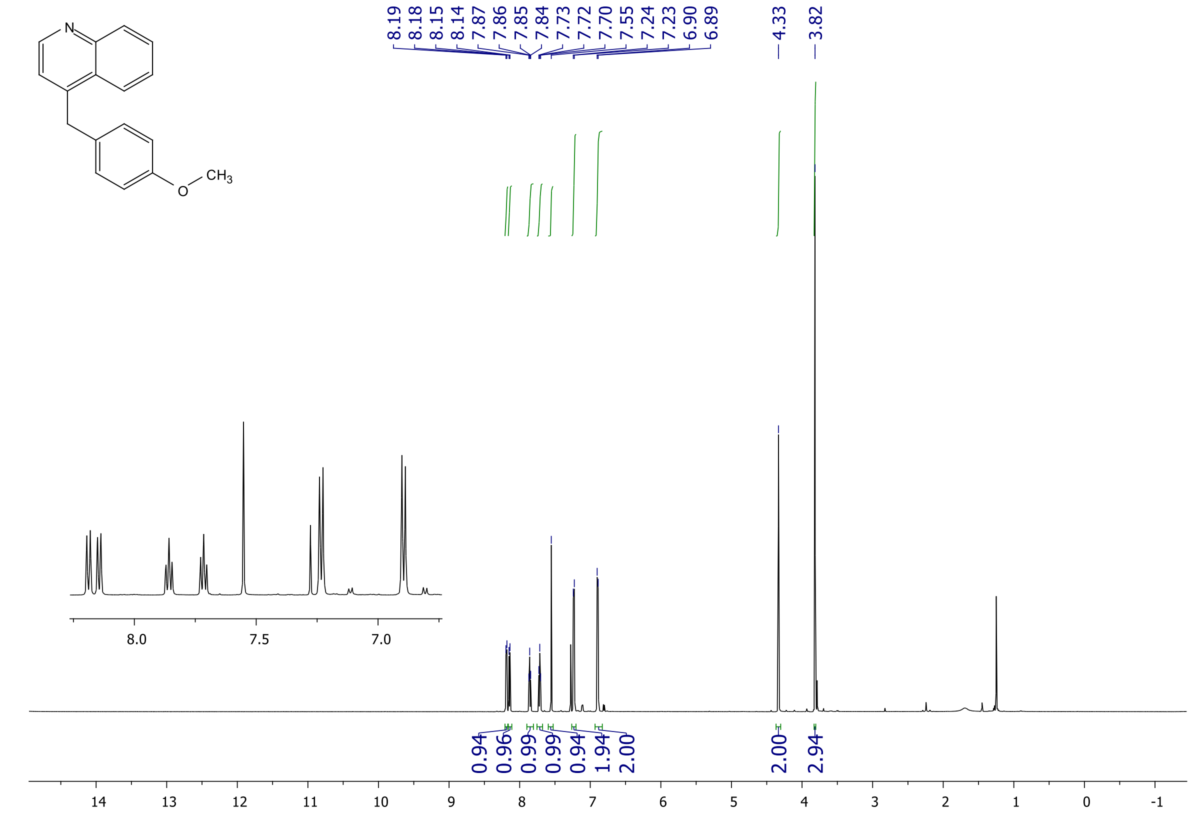

^{13}C-NMR, $CDCl_3$, 151 MHz

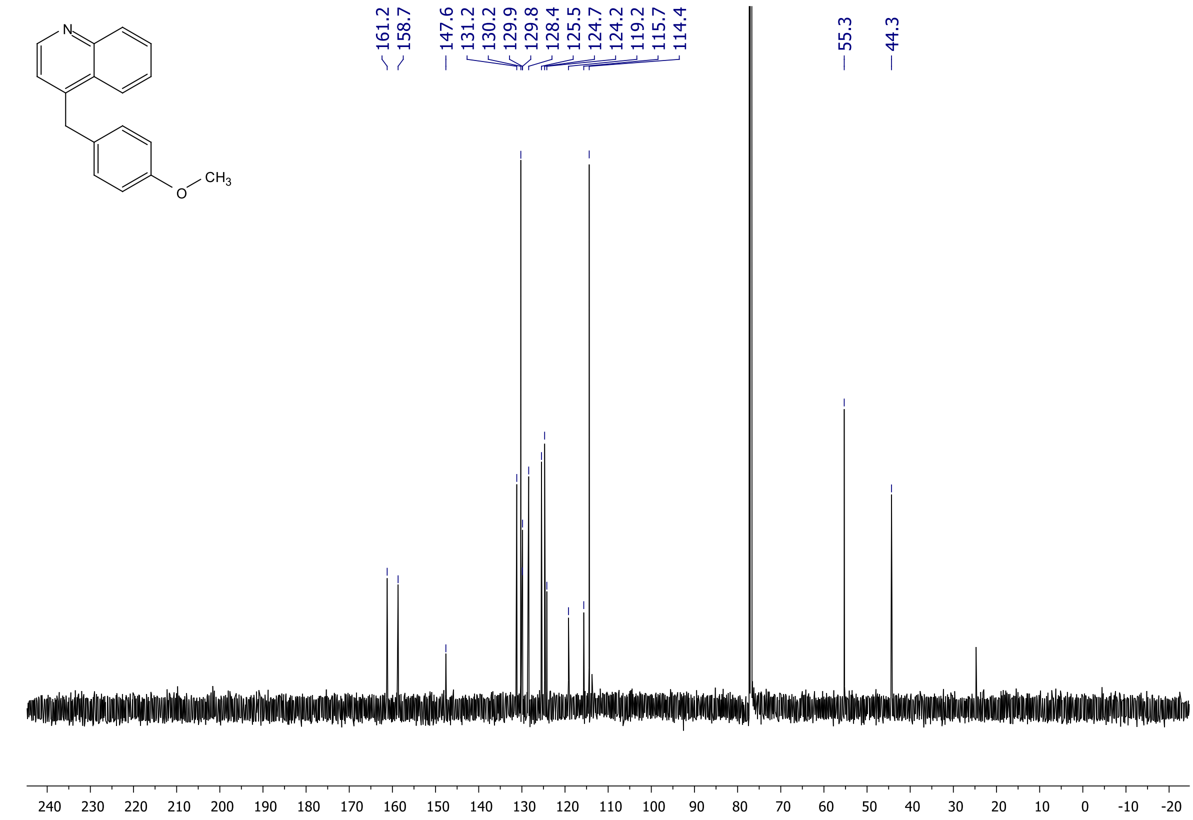

4-(4-methoxybenzyl)picolinonitrile (5o)

^{1}H-NMR, $CDCl_3$, 600 MHz

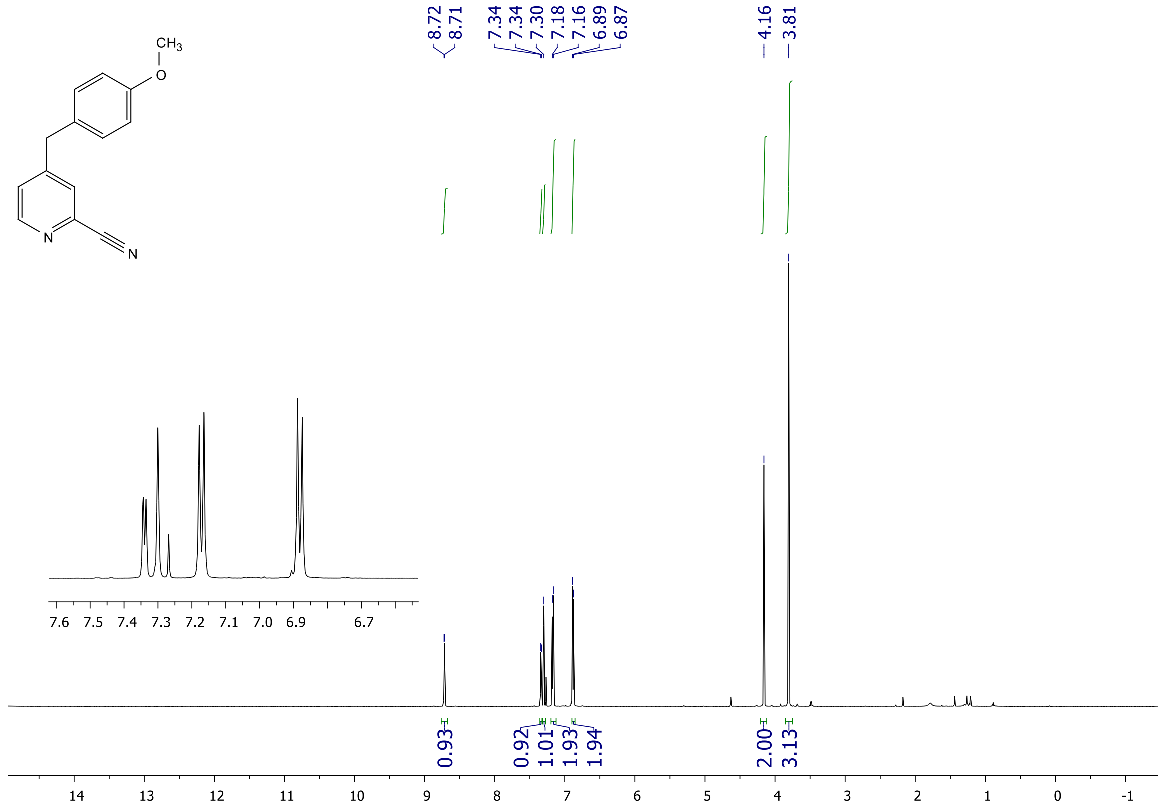

^{13}C-NMR, $CDCl_3$, 151 MHz

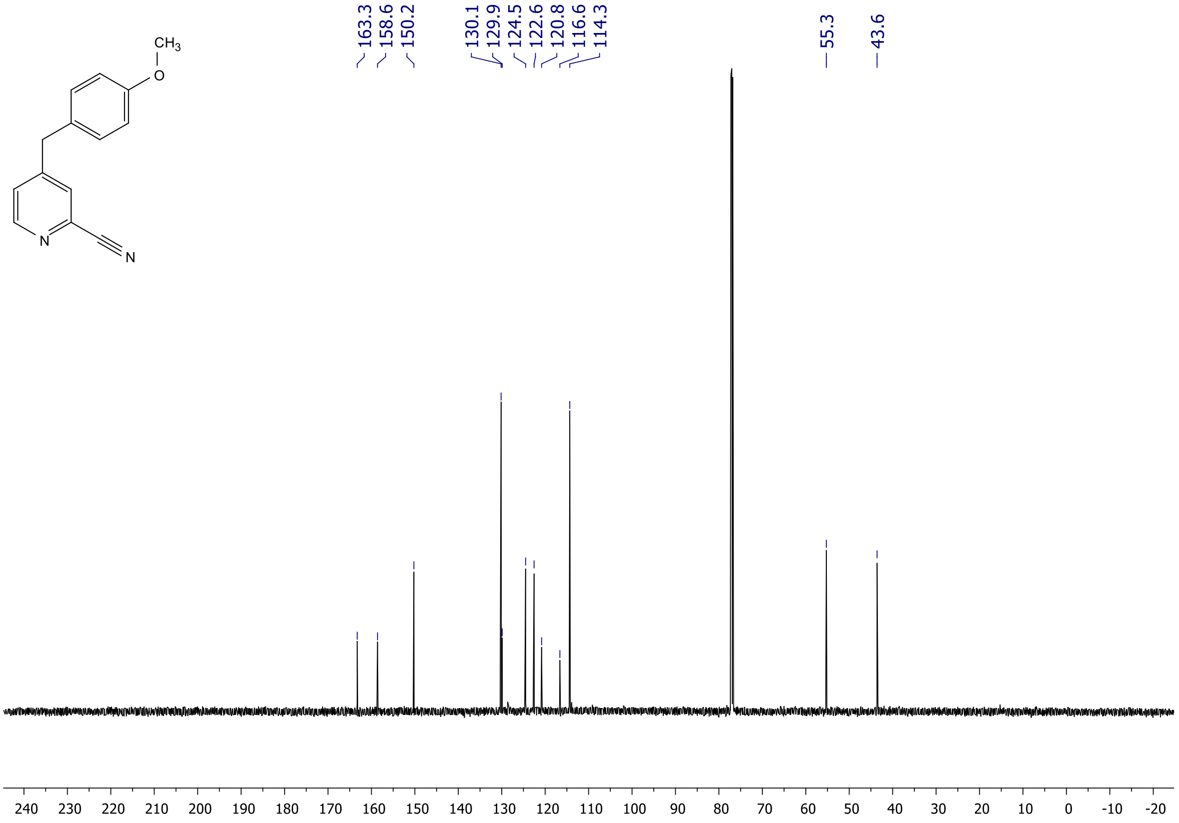

4-(4-methoxybenzyl)nicotinonitrile (5p)

^{1}H-NMR, CDCl$_3$, 600 MHz

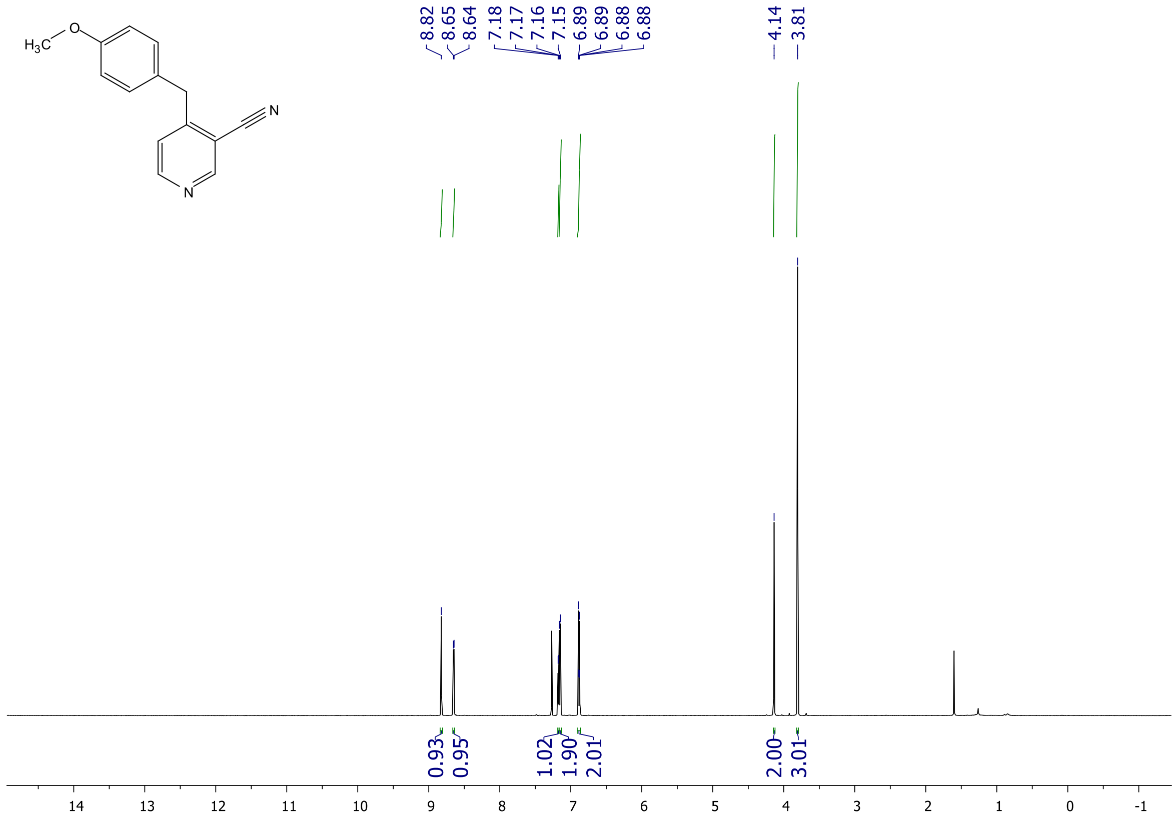

^{13}C-NMR, CDCl$_3$, 151 MHz

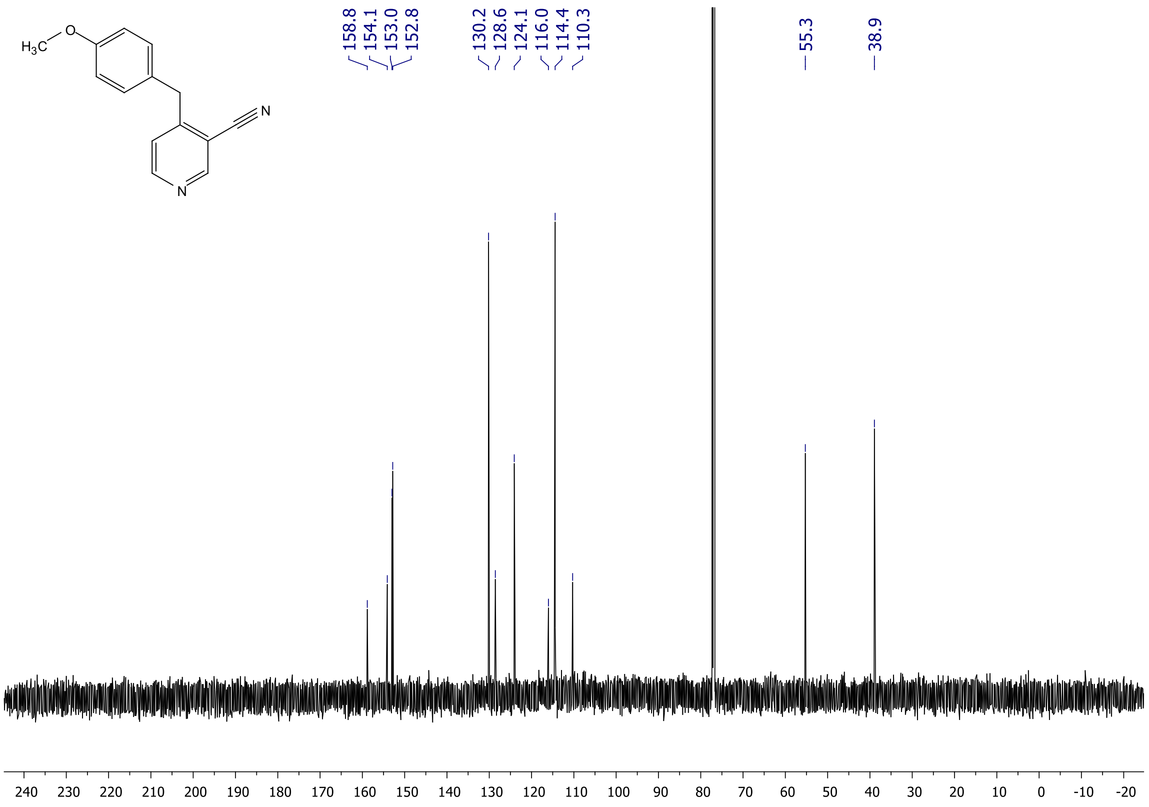

2-(4-methoxybenzyl)pyridine (5q)

^{1}H-NMR, $CDCl_3$, 600 MHz

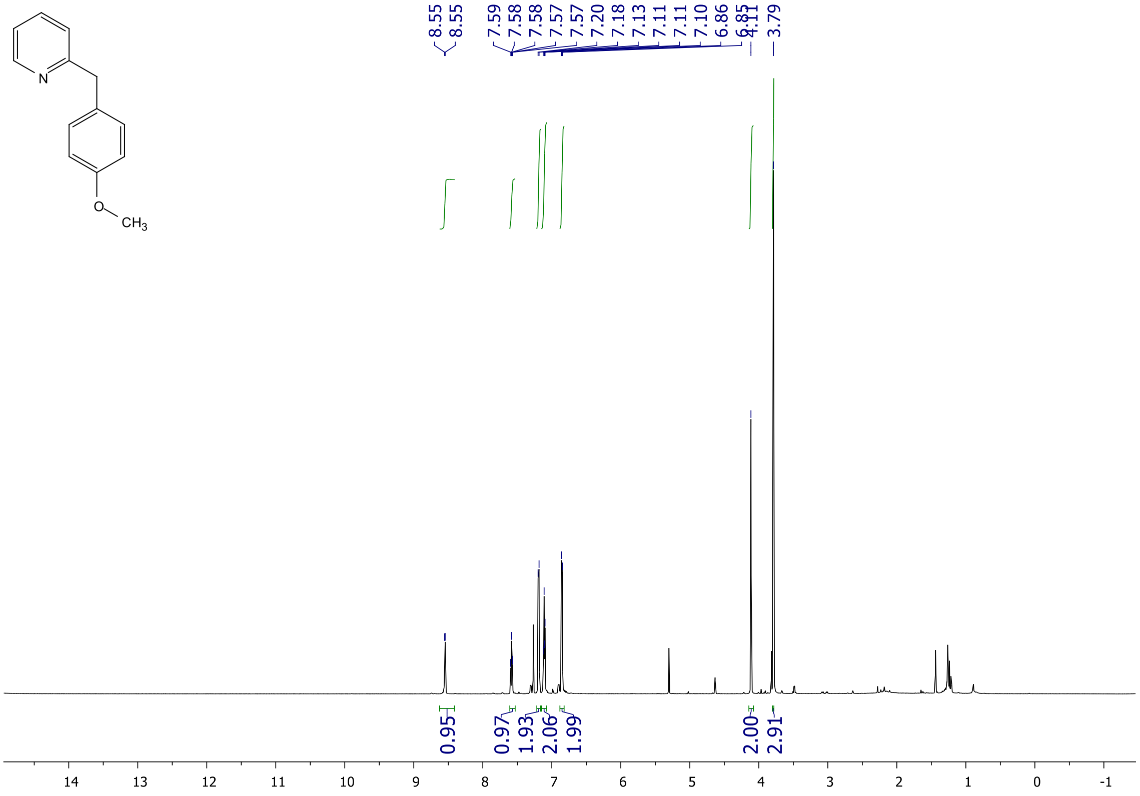

^{13}C-NMR, $CDCl_3$, 151 MHz

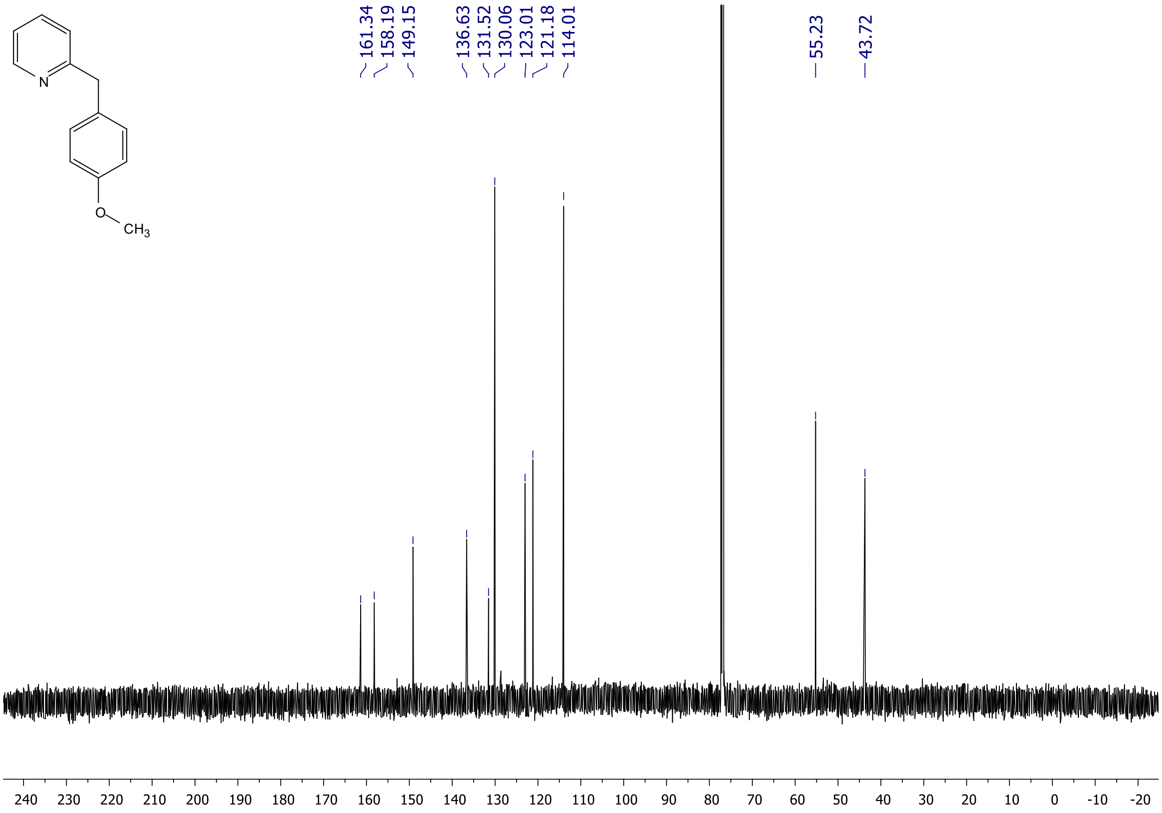

2-benzhydrylquinoline (5r)

^{1}H-NMR, $CDCl_3$, 600 MHz

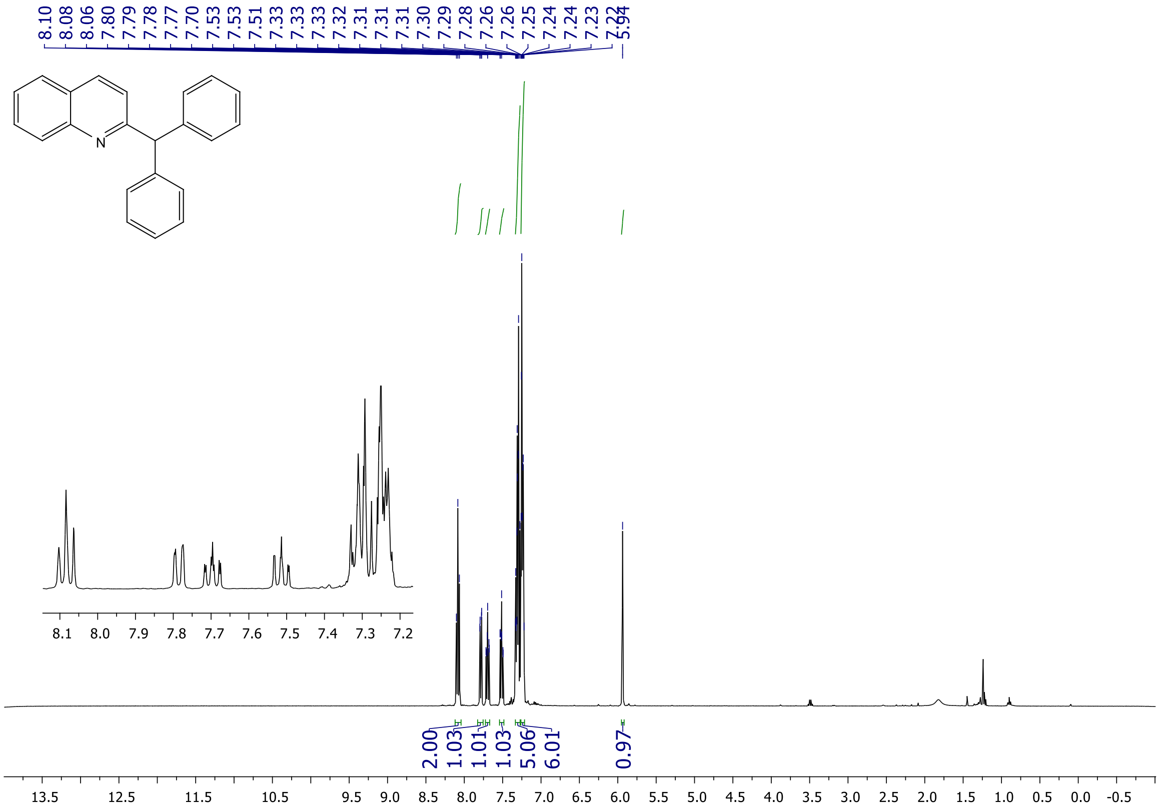

^{13}C-NMR, $CDCl_3$, 151 MHz

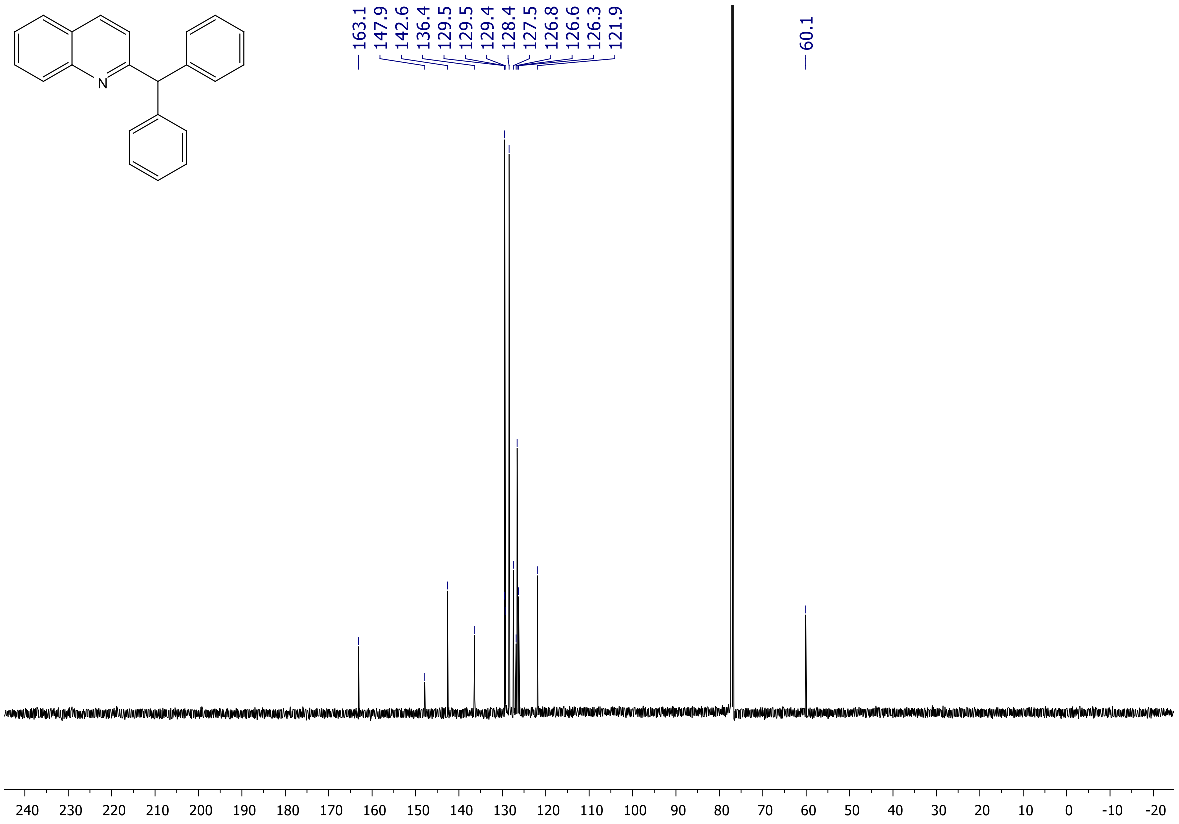

4-allylpyridine (5s)

^{1}H-NMR, $CDCl_3$, 600 MHz

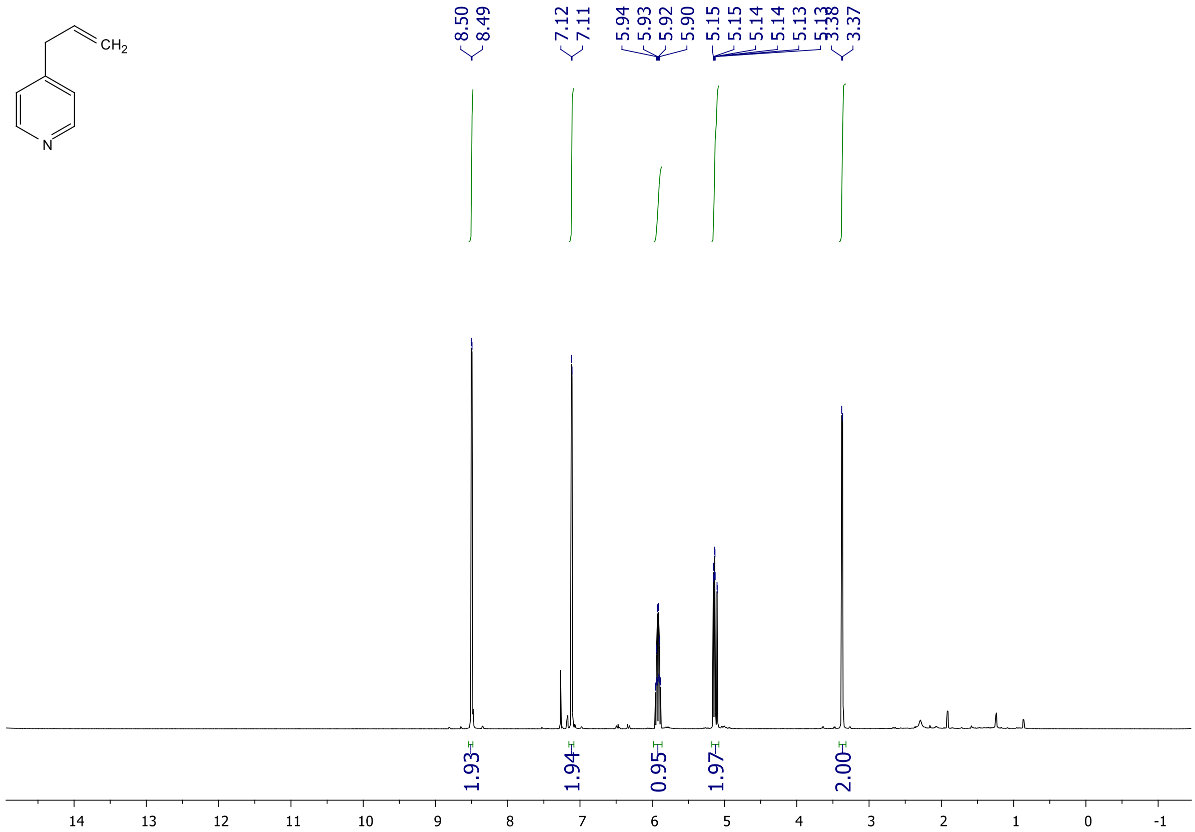

^{13}C-NMR, $CDCl_3$, 151 MHz

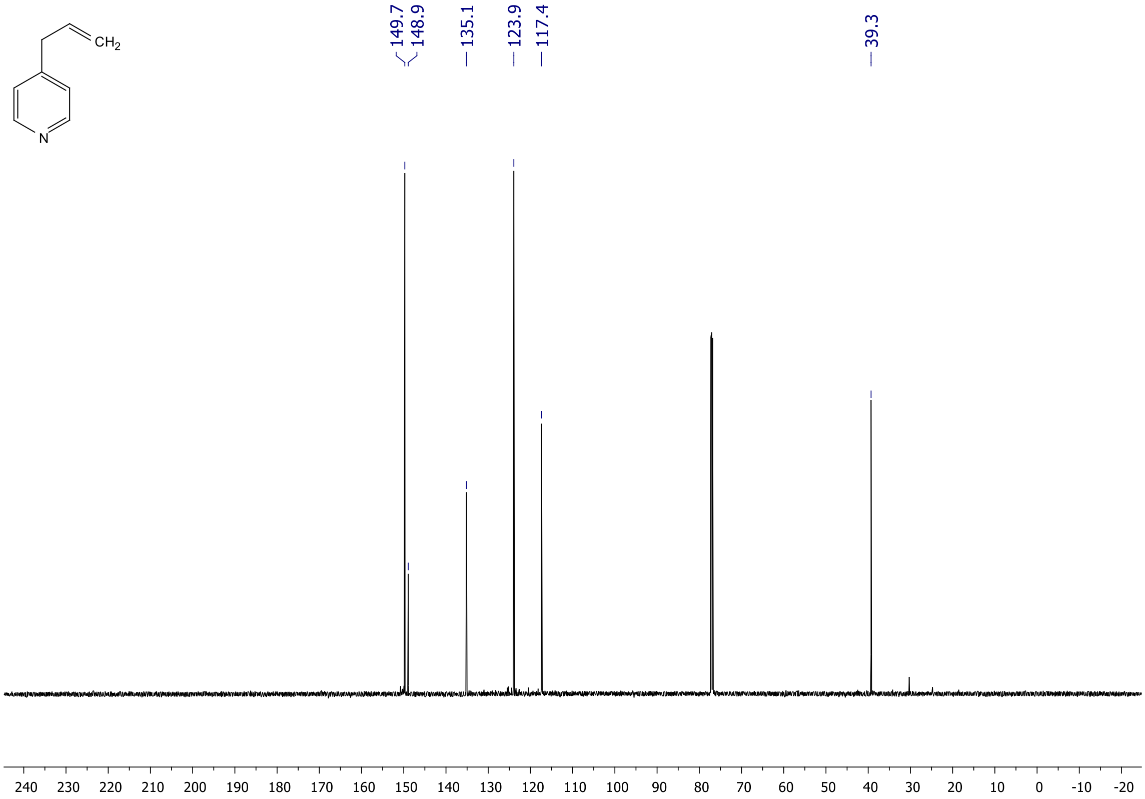

1-allylisoquinoline (5t)

^{1}H-NMR, $CDCl_3$, 600 MHz

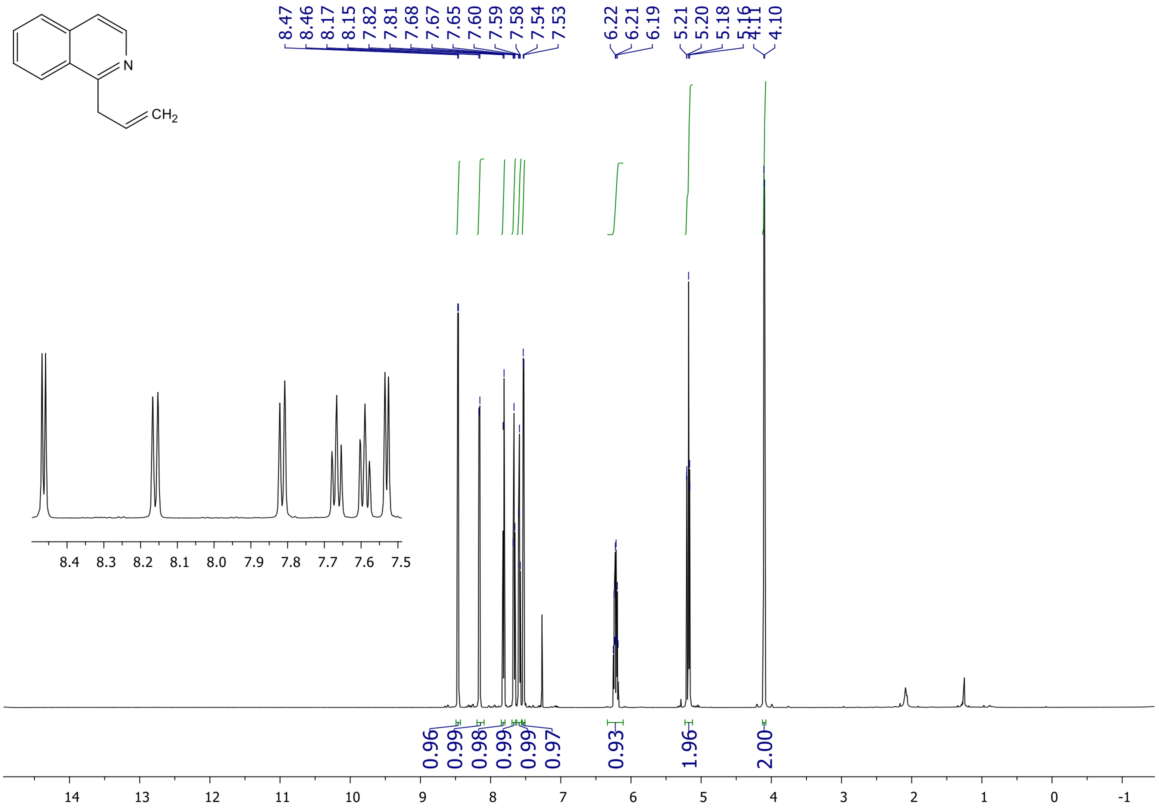

^{13}C-NMR, $CDCl_3$, 151 MHz

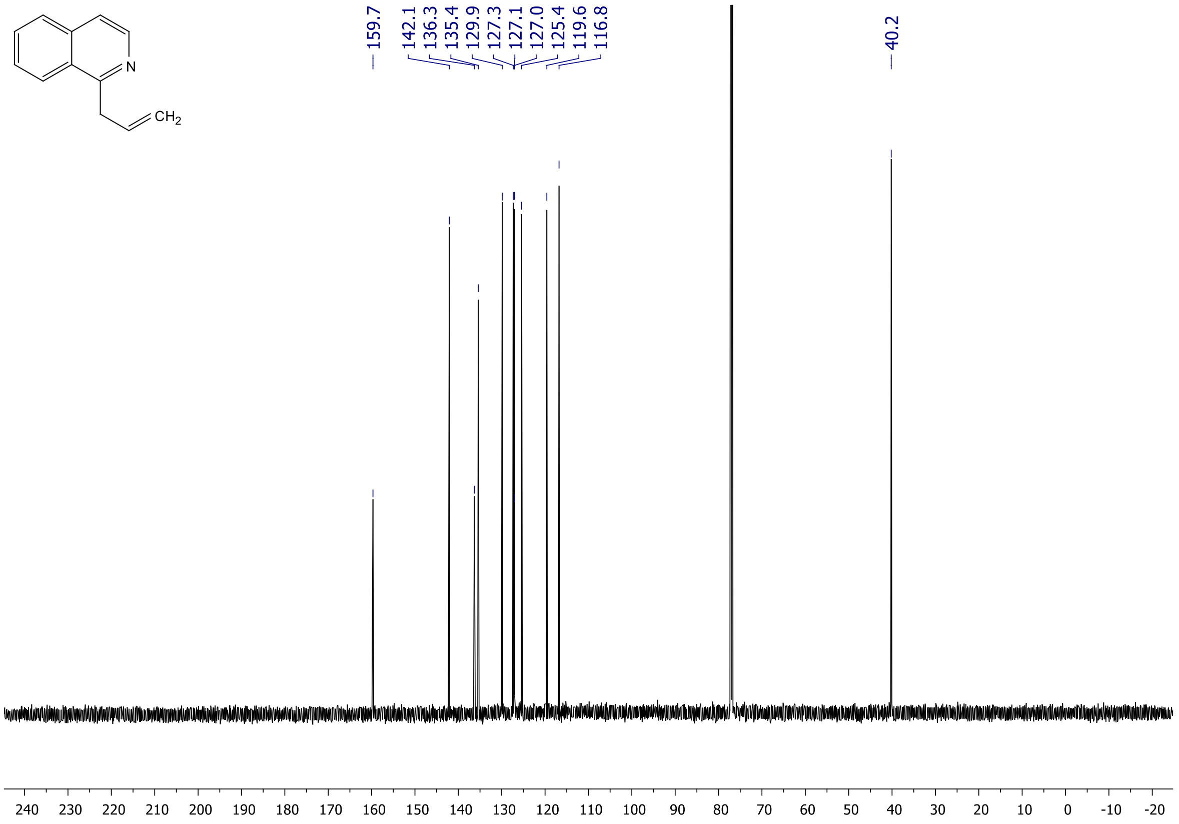

4-allyl-1H-pyrrolo[2,3-b]pyridine (5u)

^{1}H-NMR, $CDCl_3$, 600 MHz

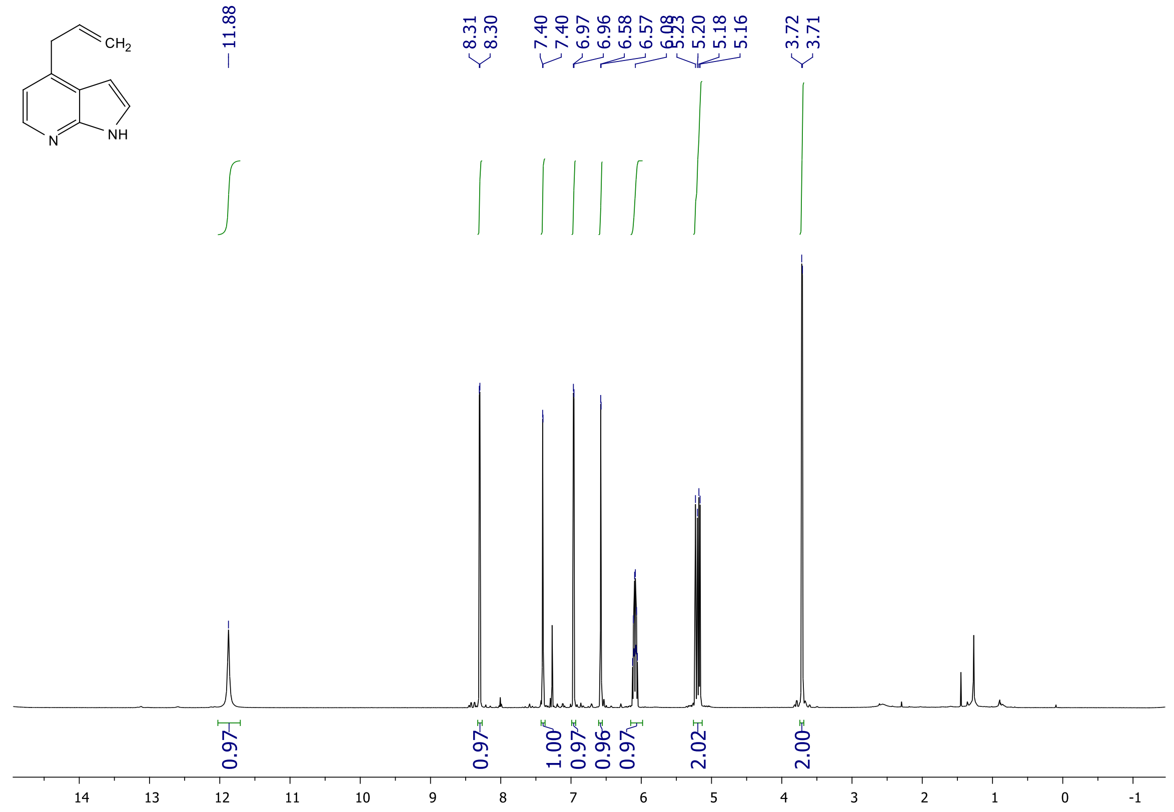

^{13}C-NMR, $CDCl_3$, 151 MHz

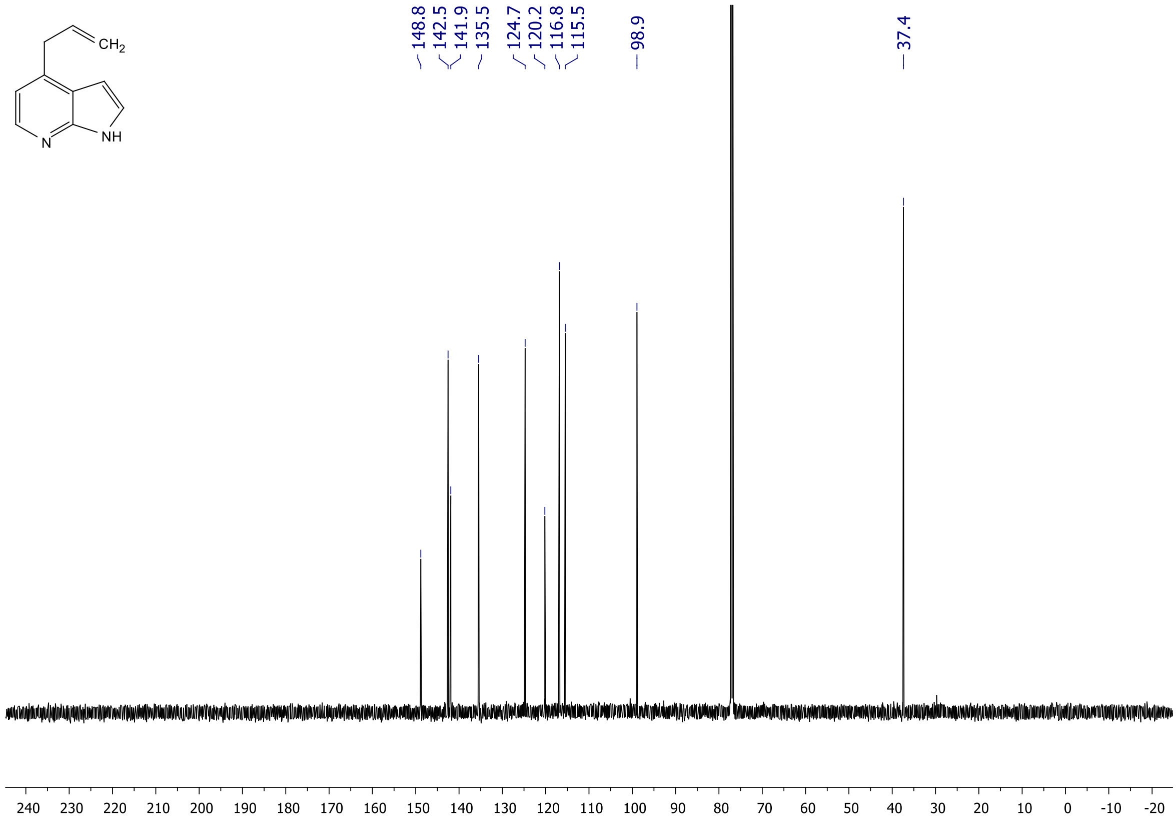

8 *Cartesian coordinates of all computed structures optimised at ωB97xd/cc-PVDZ + SMD level of theory and their uncorrected electronic energies (in Hartrees).*

2a

C	-3.074816000	0.364646000	-0.914460000
C	-3.561675000	-0.042066000	0.331318000
C	-2.862704000	-1.022726000	1.049250000
C	-1.702451000	-1.581058000	0.528525000
C	-1.200804000	-1.191960000	-0.723633000
C	-1.905372000	-0.209591000	-1.422313000
O	-4.683518000	0.442581000	0.920553000
C	0.100060000	-1.761328000	-1.254097000
B	1.273768000	-0.886600000	-0.678667000
O	1.906360000	-1.153210000	0.508226000
C	2.962548000	-0.169853000	0.668522000
C	2.487741000	0.989052000	-0.277448000
O	1.699320000	0.277884000	-1.267023000
C	3.607919000	1.736756000	-0.981110000
C	3.059455000	0.209151000	2.137305000
C	4.258678000	-0.833205000	0.207046000
C	1.541040000	1.973873000	0.406876000
C	-5.415230000	1.437783000	0.229183000
H	-3.590944000	1.124710000	-1.501142000
H	-3.248208000	-1.335889000	2.021645000
H	-1.171243000	-2.340231000	1.108373000
H	-1.537686000	0.124346000	-2.396069000
H	0.209399000	-2.811624000	-0.944709000
H	0.105880000	-1.728459000	-2.354273000
H	3.185782000	2.532447000	-1.613028000

H	4.277359000	2.206924000	-0.244704000
H	4.202861000	1.072136000	-1.621315000
H	3.401533000	-0.656899000	2.723495000
H	3.787318000	1.022901000	2.277351000
H	2.090226000	0.531531000	2.539829000
H	5.124934000	-0.173757000	0.363540000
H	4.417074000	-1.752790000	0.789751000
H	4.215233000	-1.107522000	-0.857771000
H	2.073586000	2.607881000	1.130849000
H	1.091065000	2.625867000	-0.356459000
H	0.725369000	1.452317000	0.930710000
H	-6.268502000	1.689341000	0.871338000
H	-4.811318000	2.345870000	0.062057000
H	-5.792185000	1.069016000	-0.739972000

E(RwB97XD) = -796.818826258

4a

C	-0.111931000	1.206476000	-0.000006000
C	-1.502744000	1.143794000	0.000002000
N	-2.196019000	0.000005000	0.000011000
C	-1.502756000	-1.143784000	0.000001000
C	-0.111938000	-1.206480000	-0.000006000
C	0.593037000	-0.000007000	-0.000010000
C	2.033489000	-0.000011000	-0.000015000
N	3.192986000	0.000006000	0.000020000
H	0.405969000	2.165208000	-0.000013000
H	-2.086810000	2.067994000	0.000006000
H	-2.086824000	-2.067983000	0.000006000
H	0.405949000	-2.165220000	-0.000010000

E(RwB97XD) = -340.522881536

4a (radical-anion)

C	-0.129917000	1.217665000	0.000026000
C	-1.501595000	1.145192000	-0.000014000
N	-2.246057000	-0.000021000	-0.000039000
C	-1.501570000	-1.145210000	-0.000014000
C	-0.129888000	-1.217652000	0.000026000
C	0.647431000	0.000016000	0.000056000
C	2.044342000	0.000041000	0.000055000
N	3.224303000	-0.000023000	-0.000083000
H	0.366568000	2.190883000	0.000046000
H	-2.076889000	2.079681000	-0.000023000
H	-2.076839000	-2.079715000	-0.000024000
H	0.366619000	-2.190858000	0.000047000

E(UwB97XD) = -340.609277048

6

C	-0.338019000	-1.082370000	-1.741781000
B	-1.320200000	-0.387211000	-0.649321000
N	-0.523401000	1.048460000	-0.230191000
C	0.290309000	1.075160000	0.831859000
C	1.068499000	2.183851000	1.127609000
C	0.983028000	3.290881000	0.281099000
C	0.125238000	3.260160000	-0.822181000
C	-0.612781000	2.108950000	-1.041641000
O	-2.619500000	-0.038321000	-1.159651000
C	-3.557680000	-0.179192000	-0.089961000
C	-2.905589000	-1.309222000	0.782769000
O	-1.506009000	-1.091041000	0.600489000
C	-3.676371000	1.152128000	0.662689000
C	-4.915730000	-0.540533000	-0.680221000

C	-3.235838000	-2.711602000	0.260949000
C	-3.232619000	-1.228012000	2.268859000
C	1.068841000	-1.273769000	-1.245861000
C	2.098330000	-0.383438000	-1.592711000
C	3.378270000	-0.500008000	-1.061881000
C	3.677531000	-1.526967000	-0.157581000
C	2.670892000	-2.429728000	0.200599000
C	1.389631000	-2.290799000	-0.340891000
O	4.953691000	-1.562807000	0.307539000
C	5.303932000	-2.610016000	1.191989000
C	1.774177000	4.465371000	0.545599000
N	2.412677000	5.409422000	0.755369000
H	-0.790989000	-2.057950000	-1.989221000
H	-0.331310000	-0.489540000	-2.672681000
H	0.293940000	0.175590000	1.446039000
H	1.725969000	2.179061000	1.995359000
H	0.032727000	4.110820000	-1.495291000
H	-1.305141000	2.012789000	-1.877721000
H	-2.742111000	1.422929000	1.175459000
H	-3.912361000	1.950558000	-0.057891000
H	-4.480611000	1.122377000	1.413809000
H	-5.639859000	-0.783493000	0.113309000
H	-5.314810000	0.312477000	-1.251161000
H	-4.842009000	-1.397613000	-1.363171000
H	-2.589278000	-3.442621000	0.770559000
H	-4.283458000	-2.990102000	0.452669000
H	-3.047068000	-2.787922000	-0.820391000
H	-4.318309000	-1.300212000	2.440789000
H	-2.750659000	-2.061221000	2.803869000

H	-2.869920000	-0.290182000	2.711529000
H	1.889760000	0.430241000	-2.293631000
H	4.168150000	0.200693000	-1.340881000
H	2.866682000	-3.242878000	0.900109000
H	0.610972000	-2.993559000	-0.035511000
H	6.364762000	-2.464816000	1.431549000
H	4.717222000	-2.571297000	2.125889000
H	5.174012000	-3.600976000	0.724009000

E(RwB97XD) = -1137.35247276

6-TS

C	0.621620000	-1.049499000	1.865636000
B	1.649360000	-0.463003000	0.800381000
N	0.356366000	1.210859000	0.195208000
C	-0.406630000	1.000571000	-0.879558000
C	-1.512441000	1.784775000	-1.184538000
C	-1.820708000	2.838033000	-0.319284000
C	-1.018409000	3.073039000	0.801049000
C	0.062502000	2.224157000	1.011677000
O	2.811345000	0.171923000	1.225844000
C	3.695215000	0.285869000	0.093158000
C	3.186534000	-0.861680000	-0.858245000
O	1.810012000	-1.029257000	-0.466516000
C	3.537039000	1.685976000	-0.503499000
C	5.126286000	0.109519000	0.584120000
C	3.879224000	-2.201729000	-0.604634000
C	3.243726000	-0.526375000	-2.342114000
C	-0.757544000	-1.345762000	1.333164000
C	-1.875947000	-0.608771000	1.750262000
C	-3.135638000	-0.816679000	1.199847000

C	-3.321448000	-1.788301000	0.207880000
C	-2.223630000	-2.546116000	-0.213092000
C	-0.964971000	-2.316434000	0.349230000
O	-4.584989000	-1.920505000	-0.272206000
C	-4.811264000	-2.893491000	-1.273935000
C	-2.963447000	3.675772000	-0.579136000
N	-3.883044000	4.350800000	-0.785725000
H	1.087043000	-1.980702000	2.240839000
H	0.554918000	-0.373005000	2.732338000
H	-0.118215000	0.153281000	-1.505119000
H	-2.119008000	1.576942000	-2.065103000
H	-1.232841000	3.887277000	1.492121000
H	0.715983000	2.355165000	1.877192000
H	2.530612000	1.850145000	-0.909486000
H	3.709286000	2.431778000	0.287669000
H	4.270827000	1.862836000	-1.304230000
H	5.832714000	0.107315000	-0.260379000
H	5.395770000	0.945204000	1.247830000
H	5.251582000	-0.824364000	1.147745000
H	3.353943000	-2.987472000	-1.168546000
H	4.929772000	-2.189654000	-0.931090000
H	3.846390000	-2.472263000	0.461550000
H	4.281210000	-0.337981000	-2.659247000
H	2.858625000	-1.374125000	-2.928931000
H	2.638927000	0.357249000	-2.586286000
H	-1.755800000	0.160882000	2.517743000
H	-3.995752000	-0.229557000	1.528686000
H	-2.329194000	-3.313866000	-0.979936000
H	-0.115056000	-2.900508000	-0.010323000

H	-5.881956000	-2.845530000	-1.508757000
H	-4.233146000	-2.680356000	-2.189390000
H	-4.568057000	-3.909832000	-0.920090000

E(RwB97XD) = -1137.34800174

7

C	-0.381639000	-1.020707000	-0.000720000
C	-0.907465000	0.281429000	-0.000455000
C	-0.025918000	1.381623000	-0.000174000
C	1.339145000	1.188440000	0.000056000
C	1.907750000	-0.122165000	0.000236000
C	0.993436000	-1.212260000	-0.000412000
O	-2.224948000	0.581928000	-0.000697000
C	3.300105000	-0.323558000	0.000449000
C	-3.153427000	-0.490038000	0.001078000
H	-1.038910000	-1.890345000	-0.001226000
H	-0.450467000	2.387487000	-0.000128000
H	2.005128000	2.054859000	0.000321000
H	1.386413000	-2.231927000	-0.000586000
H	3.988581000	0.524273000	0.001411000
H	3.717857000	-1.332726000	0.000630000
H	-4.148447000	-0.029013000	0.003201000
H	-3.048053000	-1.117235000	-0.899639000
H	-3.044440000	-1.117381000	0.901242000

E(UwB97XD) = -385.445688774

8

C	-0.237311000	-1.058851000	-1.713328000
B	-1.234331000	-0.362453000	-0.614083000
N	-0.620918000	1.070931000	-0.230434000

C	0.123994000	1.260005000	0.904203000
C	0.767950000	2.429160000	1.188646000
C	0.685578000	3.536864000	0.272229000
C	-0.107804000	3.322333000	-0.909229000
C	-0.718526000	2.119270000	-1.110123000
O	-2.583160000	-0.196083000	-1.157279000
C	-3.505642000	-0.437168000	-0.104801000
C	-2.747295000	-1.492387000	0.776034000
O	-1.386607000	-1.112861000	0.637360000
C	-3.776920000	0.870727000	0.653881000
C	-4.814948000	-0.940691000	-0.705250000
C	-2.908828000	-2.920254000	0.236907000
C	-3.124386000	-1.469284000	2.254417000
C	1.171167000	-1.214469000	-1.217285000
C	2.141636000	-0.219710000	-1.430645000
C	3.423856000	-0.317693000	-0.902830000
C	3.789367000	-1.429508000	-0.132776000
C	2.845112000	-2.435013000	0.093528000
C	1.559398000	-2.313772000	-0.444705000
O	5.065683000	-1.439672000	0.339797000
C	5.463444000	-2.539421000	1.133256000
C	1.340194000	4.753307000	0.514752000
N	1.887611000	5.772811000	0.718305000
H	-0.659808000	-2.045801000	-1.970207000
H	-0.241918000	-0.458882000	-2.641236000
H	0.158914000	0.400462000	1.573253000
H	1.342462000	2.514182000	2.111859000
H	-0.232220000	4.117153000	-1.645841000
H	-1.335574000	1.930286000	-1.989286000

H	-2.877859000	1.233225000	1.172215000
H	-4.083094000	1.643073000	-0.069322000
H	-4.584309000	0.759091000	1.394926000
H	-5.520507000	-1.255950000	0.080377000
H	-5.295830000	-0.136506000	-1.284716000
H	-4.645928000	-1.787413000	-1.384604000
H	-2.185883000	-3.575581000	0.747645000
H	-3.918527000	-3.324612000	0.411456000
H	-2.699739000	-2.961275000	-0.842554000
H	-4.199350000	-1.665992000	2.397742000
H	-2.565654000	-2.249299000	2.796089000
H	-2.881166000	-0.502325000	2.716431000
H	1.881184000	0.663655000	-2.020960000
H	4.163821000	0.466631000	-1.076814000
H	3.091844000	-3.316653000	0.685955000
H	0.833386000	-3.106148000	-0.246297000
H	6.508206000	-2.352821000	1.412542000
H	4.857190000	-2.624334000	2.051729000
H	5.407616000	-3.489834000	0.574630000

E(UwB97XD) = -1137.45784451

9

N	-1.177028000	1.756529000	-0.000016000
C	0.048564000	2.286661000	-0.000002000
C	1.234697000	1.538414000	0.000012000
C	1.164827000	0.144597000	0.000021000
C	-0.102133000	-0.462032000	0.000009000
C	-1.215040000	0.427135000	-0.000009000
C	2.359567000	-0.654082000	0.000049000
N	3.318788000	-1.306901000	-0.000054000

N	-2.346866000	-0.339581000	-0.000019000
C	-1.991683000	-1.664405000	0.000011000
C	-0.624083000	-1.792716000	0.000006000
H	0.104496000	3.378024000	-0.000007000
H	2.198669000	2.046233000	0.000019000
H	-3.293573000	0.021870000	0.000002000
H	-2.758785000	-2.434795000	0.000016000
H	-0.063360000	-2.723094000	0.000006000

E(RwB97XD) = -472.111609782

10

C	-2.141779000	-0.060384000	0.000000000
C	-1.359056000	-1.217801000	0.000001000
N	-0.025793000	-1.205907000	0.000000000
C	0.562831000	-0.001148000	-0.000002000
C	-0.116401000	1.216564000	0.000000000
C	-1.509129000	1.177426000	0.000000000
C	2.012381000	-0.012823000	0.000000000
N	3.171752000	-0.012722000	0.000001000
H	-3.229479000	-0.138583000	0.000000000
H	-1.831188000	-2.203641000	0.000000000
H	0.432104000	2.158550000	0.000000000
H	-2.086227000	2.103067000	0.000001000

E(RwB97XD) = -340.523551603

11

C	-0.349928000	2.141818000	-0.000007000
C	1.017889000	2.279880000	0.000002000
N	1.874480000	1.229408000	0.000008000
C	1.348988000	0.020675000	0.000011000
C	-0.046177000	-0.270840000	0.000007000

C	-0.924600000	0.847660000	-0.000004000
C	2.304056000	-1.071341000	0.000035000
C	-0.578652000	-1.588089000	0.000013000
H	-0.993049000	3.023551000	-0.000017000
H	1.481737000	3.268279000	0.000000000
C	-1.938577000	-1.771812000	0.000007000
C	-2.819618000	-0.658339000	-0.000006000
C	-2.329045000	0.623204000	-0.000012000
N	3.062396000	-1.948444000	-0.000045000
H	0.097017000	-2.445603000	0.000022000
H	-2.350780000	-2.782030000	0.000011000
H	-3.897337000	-0.830651000	-0.000012000
H	-3.001738000	1.482811000	-0.000021000

E(RwB97XD) = -494.161704484

12

C	-0.694020000	1.219831000	-0.000001000
C	0.694214000	1.219706000	-0.000002000
C	1.383563000	-0.000124000	-0.000006000
C	0.694020000	-1.219837000	-0.000002000
C	-0.694214000	-1.219711000	-0.000001000
C	-1.383563000	0.000118000	0.000001000
C	-2.823180000	0.000268000	0.000007000
N	-3.983103000	-0.000141000	0.000000000
C	2.823180000	-0.000274000	-0.000013000
N	3.983103000	0.000162000	0.000015000
H	-1.243810000	2.160853000	0.000000000
H	1.244159000	2.160635000	-0.000004000
H	1.243810000	-2.160858000	-0.000004000
H	-1.244159000	-2.160641000	0.000000000

E(RwB97XD) = -416.728560773

13

N	-1.212779000	1.775865000	0.000001000
C	0.053100000	2.286803000	0.000005000
C	1.228978000	1.562343000	0.000001000
C	1.211800000	0.121673000	-0.000007000
C	-0.112643000	-0.461544000	-0.000005000
C	-1.212378000	0.421413000	-0.000003000
C	2.375619000	-0.651495000	-0.000020000
N	3.351887000	-1.312923000	0.000018000
N	-2.350580000	-0.345860000	-0.000005000
C	-2.016186000	-1.681831000	0.000008000
C	-0.639403000	-1.789924000	-0.000002000
H	0.114240000	3.381511000	0.000010000
H	2.183732000	2.093150000	0.000004000
H	-3.290534000	0.028776000	0.000030000
H	-2.787044000	-2.447883000	0.000010000
H	-0.073408000	-2.719757000	-0.000010000

E(UwB97XD) = -472.197258683

14

C	-2.166358000	-0.056797000	-0.000001000
C	-1.374031000	-1.209543000	-0.000001000
N	-0.045586000	-1.253453000	0.000001000
C	0.604789000	-0.012114000	0.000002000
C	-0.122712000	1.228214000	0.000000000
C	-1.491663000	1.206820000	0.000000000
C	2.008934000	-0.016651000	0.000003000
N	3.188732000	-0.008795000	-0.000003000
H	-3.254951000	-0.128195000	-0.000002000

H	-1.870747000	-2.190201000	0.000000000
H	0.428010000	2.172696000	0.000000000
H	-2.058100000	2.141863000	0.000000000

E(UwB97XD) = -340.603072571

15

C	-0.295974000	2.163035000	-0.000007000
C	1.092690000	2.257513000	-0.000001000
N	1.943066000	1.230753000	0.000009000
C	1.374379000	-0.025769000	0.000016000
C	-0.050386000	-0.275411000	0.000008000
C	-0.913050000	0.867489000	-0.000003000
C	2.270410000	-1.120768000	0.000025000
C	-0.625640000	-1.562192000	0.000012000
H	-0.913207000	3.063909000	-0.000016000
H	1.557246000	3.251758000	-0.000004000
C	-2.010230000	-1.731008000	0.000005000
C	-2.853741000	-0.615961000	-0.000006000
C	-2.307598000	0.667503000	-0.000010000
N	2.996680000	-2.041958000	-0.000040000
H	0.026603000	-2.439527000	0.000020000
H	-2.432102000	-2.738679000	0.000009000
H	-3.938211000	-0.747893000	-0.000011000
H	-2.963720000	1.542285000	-0.000018000

E(UwB97XD) = -494.258003513

16

C	-0.684570000	1.232627000	-0.000001000
C	0.684193000	1.232856000	0.000009000
C	1.425612000	0.000235000	0.000018000
C	0.684570000	-1.232618000	0.000009000

C	-0.684193000	-1.232847000	-0.000001000
C	-1.425612000	-0.000227000	-0.000008000
C	-2.831912000	-0.000494000	-0.000018000
N	-4.006039000	0.000255000	0.000003000
C	2.831912000	0.000506000	0.000024000
N	4.006039000	-0.000290000	-0.000034000
H	-1.226462000	2.181145000	-0.000003000
H	1.225789000	2.181545000	0.000012000
H	1.226462000	-2.181136000	0.000012000
H	-1.225789000	-2.181536000	-0.000003000

E(UwB97XD) = -416.824288677

17

C	-2.499169000	1.217211000	0.137737000
C	-1.201298000	1.185957000	-0.372139000
C	-0.547380000	-0.028886000	-0.632867000
C	-1.243187000	-1.213826000	-0.346630000
C	-2.541455000	-1.189175000	0.163622000
C	-3.178587000	0.028127000	0.409629000
H	-2.984257000	2.178465000	0.325713000
H	-0.678809000	2.124320000	-0.575847000
H	-0.754300000	-2.174217000	-0.531444000
H	-3.059615000	-2.128873000	0.371154000
H	-4.195468000	0.050173000	0.807592000
C	0.870727000	-0.057691000	-1.121159000
H	1.045988000	-0.974148000	-1.710752000
H	1.054466000	0.797711000	-1.794220000
B	1.943200000	-0.001005000	0.098823000
F	3.286042000	-0.038678000	-0.399366000
F	1.805288000	1.199677000	0.868548000

F	1.786236000	-1.107523000	0.995767000

E(RwB97XD) = -595.716933098

18 +19

C	-2.421213000	1.212733000	0.281482000
C	-1.262805000	1.218977000	-0.479392000
C	-0.650482000	-0.000423000	-0.887205000
C	-1.262895000	-1.219371000	-0.478169000
C	-2.421307000	-1.212276000	0.282686000
C	-3.009314000	0.000445000	0.667438000
H	-2.876820000	2.158202000	0.583506000
H	-0.802639000	2.164912000	-0.774059000
H	-0.802792000	-2.165631000	-0.771880000
H	-2.876985000	-2.157408000	0.585652000
H	-3.921427000	0.000778000	1.267325000
C	0.538329000	-0.000823000	-1.646951000
H	0.977367000	-0.939130000	-1.993575000
H	0.977272000	0.937174000	-1.994558000
B	2.229236000	0.000184000	0.489906000
F	3.255554000	-0.001158000	-0.353840000
F	1.768764000	1.150474000	0.965778000
F	1.766567000	-1.148804000	0.966809000

E(UwB97XD) = -595.521391320

20

C	-2.947251000	0.500317000	-0.879906000
C	-3.532566000	-0.053191000	0.298604000
C	-2.927482000	-1.177408000	0.949160000
C	-1.789027000	-1.725096000	0.434873000
C	-1.197657000	-1.196484000	-0.749840000
C	-1.807171000	-0.065714000	-1.378027000

O	-4.619726000	0.394312000	0.862540000
C	0.067156000	-1.750471000	-1.273535000
B	1.239464000	-0.857667000	-0.662939000
O	1.897022000	-1.179651000	0.484464000
C	2.961035000	-0.193190000	0.648052000
C	2.443431000	1.005595000	-0.225433000
O	1.610138000	0.332164000	-1.215179000
C	3.530702000	1.783414000	-0.945720000
C	3.110851000	0.118890000	2.126871000
C	4.235467000	-0.832312000	0.104104000
C	1.521941000	1.955085000	0.536006000
C	-5.335547000	1.516488000	0.304320000
H	-3.400084000	1.357897000	-1.375104000
H	-3.403559000	-1.568870000	1.848600000
H	-1.320980000	-2.579385000	0.925243000
H	-1.346011000	0.347420000	-2.276051000
H	0.204088000	-2.795195000	-0.964891000
H	0.112916000	-1.658911000	-2.368902000
H	3.080651000	2.602075000	-1.526436000
H	4.227375000	2.225664000	-0.217674000
H	4.100762000	1.146703000	-1.634592000
H	3.473532000	-0.772168000	2.660144000
H	3.845297000	0.925368000	2.272652000
H	2.158519000	0.426388000	2.578305000
H	5.104044000	-0.174547000	0.251775000
H	4.422189000	-1.773922000	0.640930000
H	4.147914000	-1.061756000	-0.968548000
H	2.083275000	2.556953000	1.265054000
H	1.044038000	2.639609000	-0.180286000

H	0.728048000	1.409742000	1.069019000
H	-6.192230000	1.663098000	0.968364000
H	-4.694244000	2.407991000	0.306604000
H	-5.675634000	1.274055000	-0.711288000

E(UwB97XD) = -796.613896844

21

N	0.000017000	-0.947460000	-0.008064000
C	1.156776000	-0.267214000	-0.004808000
C	1.199075000	1.130694000	-0.000204000
C	-0.000024000	1.836819000	0.002435000
C	-1.199079000	1.130668000	0.000156000
C	-1.156734000	-0.267268000	-0.004742000
C	2.415409000	-1.090680000	0.003044000
H	2.158061000	1.651486000	-0.001017000
H	-0.000035000	2.929075000	0.003570000
H	-2.158100000	1.651397000	-0.000127000
C	-2.415413000	-1.090684000	0.002847000
H	2.380558000	-1.852392000	-0.790412000
H	3.309764000	-0.468732000	-0.139460000
H	2.515320000	-1.625824000	0.961138000
H	-2.516157000	-1.625076000	0.961276000
H	-3.309592000	-0.468750000	-0.140863000
H	-2.380004000	-1.852970000	-0.790029000

E(RwB97XD) = -326.931898786

22

N	0.000031000	-1.420089000	0.000056000
C	-1.141594000	-0.721253000	-0.000011000
C	-1.197355000	0.671768000	0.000020000
C	-0.000038000	1.384206000	0.000026000

C	1.197323000	0.671820000	0.000029000
C	1.141637000	-0.721190000	-0.000031000
H	-2.066460000	-1.306627000	-0.000141000
H	-2.161336000	1.182735000	-0.000053000
H	-0.000060000	2.476094000	-0.000239000
H	2.161275000	1.182844000	0.000087000
H	2.066524000	-1.306529000	-0.000253000

E(RwB97XD) = -248.286479283

23

N	-2.672305000	0.000012000	0.000046000
C	-1.951664000	-1.131477000	0.005463000
C	-0.566644000	-1.198747000	0.005477000
C	0.188067000	-0.000022000	-0.000128000
C	-0.566620000	1.198709000	-0.005556000
C	-1.951648000	1.131476000	-0.005451000
N	1.548376000	-0.000003000	-0.000018000
H	-2.523684000	-2.065708000	0.010734000
H	-0.084862000	-2.175373000	0.011735000
H	-0.084807000	2.175322000	-0.011705000
H	-2.523634000	2.065730000	-0.010511000
C	2.274676000	-1.255024000	-0.009643000
C	2.274586000	1.255056000	0.009710000
H	2.063524000	1.858029000	-0.890199000
H	2.026356000	1.863219000	0.896165000
H	3.349946000	1.045854000	0.034194000
H	2.063694000	-1.857912000	0.890352000
H	2.026435000	-1.863310000	-0.896002000
H	3.350012000	-1.045732000	-0.034196000

E(RwB97XD) = -382.271298349

24

C	0.042489000	-0.521172000	-1.740929000
B	-1.048019000	-0.265002000	-0.556592000
N	-0.705621000	1.362481000	0.042089000
C	0.110595000	1.568177000	1.112130000
C	0.360701000	2.858178000	1.583039000
C	-0.207634000	3.956035000	0.959858000
C	-1.002115000	3.735830000	-0.153839000
C	-1.237215000	2.437310000	-0.603752000
O	-2.427554000	-0.254266000	-0.996455000
C	-3.135428000	-1.295396000	-0.333429000
C	-2.310139000	-1.482842000	0.980766000
O	-0.984480000	-1.193449000	0.541717000
C	-4.581046000	-0.857876000	-0.126395000
C	-3.103113000	-2.541950000	-1.225334000
C	-2.334485000	-2.895071000	1.553234000
C	-2.723465000	-0.490491000	2.074673000
C	1.483178000	-0.522904000	-1.317847000
C	2.243587000	0.658335000	-1.309479000
C	3.539386000	0.691129000	-0.808253000
C	4.131952000	-0.473072000	-0.300908000
C	3.406094000	-1.667700000	-0.323386000
C	2.101805000	-1.676824000	-0.827854000
O	5.398402000	-0.343029000	0.175474000
C	6.009082000	-1.487709000	0.738663000
H	-0.221243000	-1.501690000	-2.174075000
H	-0.101604000	0.219703000	-2.544837000
C	0.788211000	0.440426000	1.841043000
H	1.017246000	2.982410000	2.444043000

H	-0.019303000	4.967590000	1.323297000
H	-1.449580000	4.568495000	-0.696297000
C	-2.096124000	2.282249000	-1.827777000
H	-4.641472000	0.113435000	0.383311000
H	-5.085421000	-0.758343000	-1.100491000
H	-5.138997000	-1.599762000	0.466955000
H	-3.727079000	-3.355976000	-0.824981000
H	-3.485643000	-2.274983000	-2.222597000
H	-2.076255000	-2.917900000	-1.346345000
H	-1.740863000	-2.934222000	2.480045000
H	-3.362896000	-3.205045000	1.797751000
H	-1.907214000	-3.622763000	0.850172000
H	-3.718598000	-0.728848000	2.480250000
H	-2.004925000	-0.542199000	2.906597000
H	-2.742702000	0.543692000	1.700926000
H	1.802524000	1.583078000	-1.692070000
H	4.114793000	1.619385000	-0.800228000
H	3.838436000	-2.596301000	0.050152000
H	1.544505000	-2.617065000	-0.819564000
H	6.999655000	-1.166608000	1.084806000
H	5.436389000	-1.874441000	1.599057000
H	6.134532000	-2.294146000	-0.004122000
H	-2.150879000	3.249056000	-2.344987000
H	-1.711239000	1.526639000	-2.519912000
H	-3.109107000	1.965369000	-1.550314000
H	1.116776000	-0.360488000	1.176244000
H	1.654755000	0.847317000	2.379800000
H	0.106009000	-0.009762000	2.574515000

E(RwB97XD) = -1123.75059220

25

C	-0.047211000	-0.536236000	-1.754738000
B	-1.132017000	-0.109450000	-0.618547000
N	-0.622479000	1.383856000	-0.062860000
C	0.239407000	1.453278000	0.961314000
C	0.769086000	2.662802000	1.386027000
C	0.392325000	3.829187000	0.725386000
C	-0.506759000	3.746083000	-0.336718000
C	-0.994173000	2.501194000	-0.703190000
O	-2.488938000	0.079301000	-1.095611000
C	-3.340106000	-0.810648000	-0.375039000
C	-2.548239000	-1.056337000	0.956445000
O	-1.190769000	-1.001308000	0.518277000
C	-4.704348000	-0.157389000	-0.187891000
C	-3.496372000	-2.091954000	-1.201992000
C	-2.797561000	-2.415175000	1.600733000
C	-2.797990000	0.043412000	1.997128000
C	1.370843000	-0.591065000	-1.258999000
C	2.268921000	0.466881000	-1.475500000
C	3.553277000	0.460219000	-0.941813000
C	3.990331000	-0.620103000	-0.165239000
C	3.116439000	-1.688600000	0.061910000
C	1.829056000	-1.660018000	-0.481569000
O	5.260146000	-0.540818000	0.313385000
C	5.730557000	-1.609882000	1.111064000
H	-0.358756000	-1.526737000	-2.129235000
H	-0.120035000	0.161509000	-2.607000000
H	0.480915000	0.501764000	1.434201000
H	1.467270000	2.681319000	2.222405000

H	0.793179000	4.795382000	1.035850000
H	-0.832275000	4.634143000	-0.877833000
H	-1.707576000	2.363691000	-1.515828000
H	-4.616165000	0.839034000	0.266290000
H	-5.201033000	-0.039795000	-1.163901000
H	-5.356154000	-0.776874000	0.448629000
H	-4.226668000	-2.787389000	-0.760028000
H	-3.846611000	-1.822516000	-2.210324000
H	-2.534556000	-2.616220000	-1.306674000
H	-2.228315000	-2.493942000	2.540471000
H	-3.864179000	-2.550424000	1.840468000
H	-2.478693000	-3.236944000	0.945430000
H	-2.047548000	-0.044072000	2.798156000
H	-2.714828000	1.050077000	1.562713000
H	-3.794924000	-0.050632000	2.454770000
H	1.950907000	1.325979000	-2.073598000
H	4.239287000	1.291434000	-1.118533000
H	3.421811000	-2.547967000	0.659515000
H	1.154589000	-2.495253000	-0.279009000
H	6.758192000	-1.349904000	1.394691000
H	5.126757000	-1.735073000	2.026263000
H	5.744439000	-2.561611000	0.552542000

E(RwB97XD) = -1045.11918163

26

C	0.826242000	-1.376190000	-1.621289000
B	1.560098000	-0.334208000	-0.602455000
N	0.467121000	0.856593000	-0.296536000
C	0.366585000	1.930750000	-1.099004000
C	-0.610378000	2.887821000	-0.953507000

C	-1.579569000	2.757236000	0.078646000
C	-1.445538000	1.620628000	0.920268000
C	-0.430223000	0.720300000	0.696332000
O	1.942734000	-0.932106000	0.665487000
C	3.180652000	-0.349366000	1.063119000
C	3.855036000	-0.018996000	-0.314317000
O	2.746450000	0.313895000	-1.144548000
C	2.912611000	0.909147000	1.900162000
C	3.943077000	-1.358306000	1.914956000
C	4.558280000	-1.239201000	-0.921580000
C	4.823966000	1.157560000	-0.279077000
N	-2.568434000	3.660541000	0.248989000
C	-3.553271000	3.473306000	1.301036000
C	-2.664480000	4.814258000	-0.629317000
C	-0.458978000	-1.938288000	-1.082760000
C	-0.454721000	-2.910992000	-0.066756000
C	-1.631514000	-3.367866000	0.511704000
C	-2.871644000	-2.861244000	0.095523000
C	-2.904477000	-1.897872000	-0.915144000
C	-1.705742000	-1.453690000	-1.486463000
O	-3.965191000	-3.364987000	0.727199000
H	0.638006000	-0.870955000	-2.584859000
H	1.544674000	-2.189735000	-1.823646000
H	1.124414000	2.005263000	-1.879387000
H	-0.619216000	3.729280000	-1.642916000
H	-2.128944000	1.434628000	1.745813000
H	-0.296527000	-0.158520000	1.326541000
H	2.426792000	1.698527000	1.308661000
H	2.241816000	0.647751000	2.733550000

H	3.840674000	1.319606000	2.327842000
H	4.959468000	-0.999521000	2.142880000
H	3.418532000	-1.513372000	2.871031000
H	4.019583000	-2.332038000	1.412374000
H	4.806870000	-1.018995000	-1.971334000
H	5.491564000	-1.490351000	-0.393933000
H	3.902387000	-2.122626000	-0.907437000
H	5.650066000	0.972040000	0.426056000
H	5.262994000	1.311215000	-1.277643000
H	4.318028000	2.088463000	0.011669000
H	-3.083980000	3.472272000	2.298276000
H	-4.275526000	4.295806000	1.263836000
H	-4.103415000	2.526577000	1.173655000
H	-2.818457000	4.512214000	-1.678434000
H	-3.518889000	5.426263000	-0.321309000
H	-1.757926000	5.438492000	-0.574880000
H	0.501650000	-3.301308000	0.288204000
H	-1.613185000	-4.123621000	1.300171000
H	-3.848253000	-1.481979000	-1.269264000
H	-1.754901000	-0.690666000	-2.268839000
C	-5.234027000	-2.888761000	0.324630000
H	-5.970414000	-3.419180000	0.941491000
H	-5.338228000	-1.803592000	0.497407000
H	-5.434212000	-3.106652000	-0.738550000

E(RwB97XD) = -1179.10935413

27

C	-0.206617000	-1.144110000	-1.699535000
B	-1.184810000	-0.732718000	-0.469081000
N	-0.495318000	0.671673000	0.176504000

C	0.458604000	0.601438000	1.113699000
C	1.214014000	1.705313000	1.503592000
C	0.987414000	2.946799000	0.903443000
C	-0.022034000	3.058254000	-0.062916000
C	-0.734219000	1.868594000	-0.369603000
O	-2.533068000	-0.409170000	-0.897851000
C	-3.430544000	-0.826301000	0.138863000
C	-2.657576000	-2.035348000	0.772749000
O	-1.287490000	-1.655141000	0.633670000
C	-3.635595000	0.327551000	1.126661000
C	-4.767135000	-1.190081000	-0.494405000
C	-2.869752000	-3.335064000	-0.010867000
C	-2.961418000	-2.272027000	2.246436000
C	1.237175000	-1.301355000	-1.309390000
C	2.191805000	-0.316019000	-1.607855000
C	3.504457000	-0.406629000	-1.157780000
C	3.913202000	-1.502514000	-0.387534000
C	2.983984000	-2.504289000	-0.086732000
C	1.668817000	-2.391307000	-0.545942000
O	5.212212000	-1.504789000	0.010656000
C	5.652658000	-2.583058000	0.813355000
C	1.773137000	4.092596000	1.273397000
N	2.400820000	5.022980000	1.564910000
H	-0.602016000	-2.097003000	-2.092362000
H	-0.297838000	-0.404350000	-2.513225000
H	0.615091000	-0.384139000	1.549324000
H	1.983813000	1.585481000	2.263744000
H	-2.711284000	0.577571000	1.666944000
H	-3.961705000	1.222095000	0.573459000

H	-4.412272000	0.088198000	1.868667000
H	-5.444215000	-1.639662000	0.248805000
H	-5.254397000	-0.284565000	-0.888686000
H	-4.640938000	-1.895726000	-1.326421000
H	-2.143693000	-4.084066000	0.340817000
H	-3.880977000	-3.745834000	0.132358000
H	-2.704451000	-3.181913000	-1.087762000
H	-4.032560000	-2.477917000	2.400086000
H	-2.394767000	-3.143754000	2.609039000
H	-2.678831000	-1.407263000	2.862028000
H	1.898797000	0.552321000	-2.204763000
H	4.234145000	0.370658000	-1.394481000
H	3.267024000	-3.374932000	0.505486000
H	0.951436000	-3.172266000	-0.283669000
H	6.709508000	-2.387305000	1.034068000
H	5.092557000	-2.642030000	1.762371000
H	5.570112000	-3.547599000	0.283722000
C	-0.583756000	4.087565000	-0.883444000
C	-1.578639000	3.492029000	-1.617046000
N	-1.663826000	2.153602000	-1.312829000
H	-2.347766000	1.462339000	-1.622423000
H	-0.289602000	5.132222000	-0.918875000
H	-2.253971000	3.925805000	-2.349618000

E(RwB97XD) = -1268.94197518

28

C	-0.072751000	-0.469326000	-1.924621000
B	-1.163490000	-0.213582000	-0.755051000
N	-0.764611000	1.383847000	-0.149746000
C	0.064940000	1.639544000	0.878403000

C	0.410863000	2.936563000	1.248812000
C	-0.119903000	4.003195000	0.528680000
C	-0.976999000	3.735692000	-0.531021000
C	-1.272629000	2.412575000	-0.835489000
O	-2.517785000	-0.129738000	-1.217907000
C	-3.369549000	-0.615896000	-0.174806000
C	-2.436378000	-1.632216000	0.573361000
O	-1.135098000	-1.067656000	0.396429000
C	-3.806830000	0.553418000	0.715697000
C	-4.603221000	-1.242784000	-0.812877000
C	-2.430761000	-3.016648000	-0.082198000
C	-2.718220000	-1.774851000	2.063648000
C	1.349997000	-0.488237000	-1.439513000
C	2.136345000	0.675830000	-1.423643000
C	3.414646000	0.688710000	-0.876903000
C	3.958545000	-0.478176000	-0.325363000
C	3.204477000	-1.656063000	-0.351001000
C	1.920315000	-1.645404000	-0.900921000
O	5.208856000	-0.369018000	0.194703000
C	5.775802000	-1.518666000	0.793333000
H	-0.331309000	-1.444953000	-2.371107000
H	-0.195312000	0.285310000	-2.720511000
H	1.087800000	3.094726000	2.087323000
H	-1.421185000	4.535502000	-1.122405000
H	-1.948579000	2.136655000	-1.644448000
H	-2.963832000	1.001046000	1.261011000
H	-4.258628000	1.335014000	0.085371000
H	-4.558919000	0.235680000	1.453870000
H	-5.230488000	-1.738770000	-0.055634000

H	-5.211520000	-0.462811000	-1.296852000
H	-4.328746000	-1.979870000	-1.579240000
H	-1.613261000	-3.611264000	0.353651000
H	-3.374636000	-3.558773000	0.082299000
H	-2.255169000	-2.942509000	-1.166088000
H	-3.743546000	-2.137448000	2.238336000
H	-2.021601000	-2.503843000	2.505745000
H	-2.588557000	-0.821975000	2.594670000
H	1.732416000	1.602575000	-1.841922000
H	4.011369000	1.603290000	-0.865769000
H	3.598874000	-2.585384000	0.060457000
H	1.340028000	-2.571358000	-0.893570000
H	6.762335000	-1.213897000	1.164683000
H	5.168661000	-1.879189000	1.641260000
H	5.905380000	-2.338464000	0.066037000
H	0.137102000	5.027926000	0.798253000
C	0.649579000	0.559119000	1.642354000
N	1.221950000	-0.164744000	2.343810000

E(RwB97XD) = -1137.34711197

29

C	-0.690664000	-1.232245000	-1.753555000
B	-1.532178000	-0.562274000	-0.542684000
N	-0.385344000	0.600360000	0.144078000
C	0.300276000	0.210029000	1.247527000
C	1.330727000	0.947779000	1.767187000
C	-0.065591000	1.739143000	-0.451228000
O	-2.704769000	0.158402000	-0.923013000
C	-3.641042000	0.074622000	0.156270000
C	-3.269930000	-1.301253000	0.813918000

O	-1.862727000	-1.403618000	0.575456000
C	-3.430446000	1.262848000	1.102434000
C	-5.049571000	0.142186000	-0.421317000
C	-3.944117000	-2.485507000	0.115050000
C	-3.529365000	-1.376853000	2.313289000
C	0.687114000	-1.685187000	-1.350748000
C	1.824687000	-0.921807000	-1.659146000
C	3.087001000	-1.265476000	-1.189407000
C	3.258248000	-2.404497000	-0.391811000
C	2.142966000	-3.189982000	-0.082965000
C	0.881334000	-2.821460000	-0.559679000
O	4.526623000	-2.659099000	0.023148000
C	4.740549000	-3.802796000	0.827890000
H	-1.290596000	-2.090769000	-2.102799000
H	-0.620042000	-0.531473000	-2.601984000
H	-0.038113000	-0.730545000	1.677258000
H	1.860939000	0.591152000	2.650625000
H	-2.455529000	1.224515000	1.608795000
H	-3.474650000	2.195580000	0.519183000
H	-4.213580000	1.306168000	1.874504000
H	-5.805672000	-0.033516000	0.359904000
H	-5.229715000	1.141586000	-0.846967000
H	-5.194409000	-0.595664000	-1.221772000
H	-3.487636000	-3.419233000	0.477951000
H	-5.024191000	-2.529279000	0.322407000
H	-3.799034000	-2.439180000	-0.974764000
H	-4.596063000	-1.218421000	2.537357000
H	-3.249188000	-2.372516000	2.690801000
H	-2.942164000	-0.629999000	2.864927000

H	1.716708000	-0.024575000	-2.275397000
H	3.961469000	-0.658342000	-1.432816000
H	2.237482000	-4.087053000	0.529490000
H	0.017985000	-3.433679000	-0.288830000
H	5.814763000	-3.822418000	1.050511000
H	4.178588000	-3.746574000	1.775954000
H	4.466743000	-4.731816000	0.299087000
C	1.702776000	2.166645000	1.156898000
C	0.976866000	2.589398000	0.007001000
C	2.763563000	2.977228000	1.644056000
C	3.076493000	4.151311000	1.008170000
H	3.891324000	4.773957000	1.381331000
C	2.350036000	4.571030000	-0.137602000
H	2.616579000	5.508148000	-0.628360000
C	1.321164000	3.812171000	-0.632788000
H	0.768657000	4.139245000	-1.514421000
H	3.316669000	2.647577000	2.524906000
C	-0.792288000	2.125212000	-1.640568000
N	-1.275251000	2.530750000	-2.612857000

E(RwB97XD) = -1290.98569405

30

C	-0.216483000	-0.958825000	-1.838730000
B	-1.219729000	-0.297731000	-0.591165000
N	-0.544638000	1.147608000	-0.239788000
C	0.321967000	1.253902000	0.778569000
C	0.951503000	2.451986000	1.070303000
C	0.657279000	3.562022000	0.275635000
C	-0.253685000	3.445327000	-0.777891000
C	-0.836318000	2.210924000	-1.003659000

O	-2.536314000	-0.119292000	-1.094688000
C	-3.440333000	-0.485276000	-0.034924000
C	-2.606466000	-1.544055000	0.768836000
O	-1.257380000	-1.074219000	0.599915000
C	-3.751536000	0.768902000	0.786957000
C	-4.720540000	-1.025873000	-0.653936000
C	-2.695672000	-2.948736000	0.169024000
C	-2.919967000	-1.598754000	2.256990000
C	1.109256000	-1.155718000	-1.321691000
C	2.128549000	-0.164435000	-1.483247000
C	3.359942000	-0.322091000	-0.915532000
C	3.640961000	-1.483231000	-0.136002000
C	2.646354000	-2.481814000	0.045085000
C	1.416321000	-2.309172000	-0.532206000
O	4.850105000	-1.535322000	0.378625000
C	5.238157000	-2.653449000	1.192222000
C	1.293631000	4.826956000	0.541106000
N	1.805788000	5.844038000	0.754052000
H	-0.750083000	-1.893608000	-2.050940000
H	-0.271971000	-0.241791000	-2.668983000
H	0.493148000	0.348448000	1.359543000
H	1.653720000	2.513105000	1.899869000
H	-0.507615000	4.295312000	-1.408724000
H	-1.562541000	2.045057000	-1.798146000
H	-2.856176000	1.162782000	1.290734000
H	-4.137099000	1.550413000	0.114763000
H	-4.516742000	0.570713000	1.552165000
H	-5.396558000	-1.412235000	0.124449000
H	-5.246406000	-0.220507000	-1.189062000

H	-4.514350000	-1.831766000	-1.370651000
H	-1.934518000	-3.587529000	0.642304000
H	-3.680877000	-3.405122000	0.347645000
H	-2.510767000	-2.939203000	-0.915358000
H	-3.977066000	-1.857299000	2.424049000
H	-2.302157000	-2.371492000	2.739417000
H	-2.711446000	-0.640477000	2.751433000
H	1.908766000	0.726663000	-2.074499000
H	4.148513000	0.421487000	-1.035737000
H	2.852801000	-3.372339000	0.636930000
H	0.639275000	-3.060755000	-0.389800000
H	6.270563000	-2.447089000	1.491304000
H	4.595815000	-2.722018000	2.081431000
H	5.195545000	-3.584479000	0.609574000

E(UwB97XD) = -1137.15750505

30-TS

C	0.620116000	1.211817000	-1.682099000
B	1.501990000	-0.059458000	-0.300918000
N	0.311849000	-1.033138000	-0.062949000
C	-0.517079000	-0.810852000	0.974331000
C	-1.627314000	-1.605658000	1.185153000
C	-1.867651000	-2.656561000	0.296637000
C	-0.991126000	-2.889456000	-0.766494000
C	0.096747000	-2.050356000	-0.916269000
O	2.588701000	-0.558901000	-0.998891000
C	3.739952000	-0.361103000	-0.128249000
C	3.294457000	0.858480000	0.757441000
O	1.848951000	0.691131000	0.806562000
C	3.905383000	-1.650442000	0.674095000

C	4.967916000	-0.107417000	-0.984024000
C	3.604050000	2.212628000	0.123958000
C	3.819002000	0.823977000	2.183526000
C	-0.717348000	1.422326000	-1.261812000
C	-1.784557000	0.629488000	-1.778985000
C	-3.062004000	0.753443000	-1.291408000
C	-3.341245000	1.671565000	-0.251828000
C	-2.307938000	2.474011000	0.273053000
C	-1.026176000	2.345682000	-0.226743000
O	-4.609355000	1.702645000	0.162842000
C	-4.962065000	2.568825000	1.238890000
C	-3.024339000	-3.495912000	0.474698000
N	-3.957273000	-4.167234000	0.618317000
H	1.345522000	2.006684000	-1.502038000
H	0.786433000	0.596164000	-2.570973000
H	-0.261405000	0.022239000	1.626337000
H	-2.288073000	-1.404389000	2.026073000
H	-1.149114000	-3.705488000	-1.468958000
H	0.820485000	-2.168640000	-1.721320000
H	3.029132000	-1.847697000	1.310342000
H	4.018327000	-2.493207000	-0.023621000
H	4.798905000	-1.612582000	1.313763000
H	5.832224000	0.130444000	-0.345640000
H	5.212231000	-1.010215000	-1.563185000
H	4.813129000	0.720416000	-1.687904000
H	3.045536000	2.995625000	0.657933000
H	4.676702000	2.441773000	0.202613000
H	3.327825000	2.253050000	-0.938429000
H	4.919531000	0.820590000	2.184250000

H	3.481504000	1.720136000	2.725181000
H	3.463794000	-0.061050000	2.727095000
H	-1.573332000	-0.087306000	-2.575521000
H	-3.881285000	0.150230000	-1.685213000
H	-2.505336000	3.190218000	1.069816000
H	-0.222730000	2.954705000	0.192257000
H	-6.034330000	2.415041000	1.403761000
H	-4.409098000	2.304604000	2.153833000
H	-4.777618000	3.622643000	0.979233000

E(UwB97XD) = -1137.15645608

31

No local minimum identified

32

C	-0.048324000	-0.374504000	-1.854006000
B	-1.128261000	0.058134000	-0.510342000
N	-0.623654000	1.508813000	-0.042752000
C	0.206101000	1.616949000	1.008811000
C	0.693722000	2.847475000	1.416089000
C	0.311123000	3.988495000	0.715554000
C	-0.554587000	3.862696000	-0.370009000
C	-1.006058000	2.603346000	-0.723110000
O	-2.467085000	0.144766000	-0.970833000
C	-3.202899000	-0.926138000	-0.347994000
C	-2.379443000	-1.182797000	0.961193000
O	-1.033263000	-0.898346000	0.533926000
C	-4.631380000	-0.457180000	-0.114164000
C	-3.201168000	-2.129810000	-1.292325000
C	-2.436066000	-2.612711000	1.476048000
C	-2.740884000	-0.210136000	2.086603000

C	1.276858000	-0.478183000	-1.327332000
C	2.167707000	0.641920000	-1.327930000
C	3.407182000	0.557745000	-0.759066000
C	3.826241000	-0.653883000	-0.140661000
C	2.961983000	-1.777804000	-0.117337000
C	1.719372000	-1.679349000	-0.691167000
O	5.036104000	-0.629272000	0.387321000
C	5.556028000	-1.799070000	1.032501000
H	-0.496896000	-1.324045000	-2.164904000
H	-0.195459000	0.434287000	-2.580818000
H	0.454761000	0.682452000	1.510641000
H	1.364313000	2.901539000	2.272682000
H	0.682005000	4.970036000	1.013994000
H	-0.882995000	4.730437000	-0.940685000
H	-1.689523000	2.434859000	-1.554645000
H	-4.661305000	0.500620000	0.421932000
H	-5.144620000	-0.323947000	-1.078703000
H	-5.193485000	-1.204049000	0.467560000
H	-3.864451000	-2.927681000	-0.926409000
H	-3.562608000	-1.809283000	-2.281012000
H	-2.193198000	-2.551604000	-1.416235000
H	-1.843726000	-2.700651000	2.399629000
H	-3.472791000	-2.900237000	1.709466000
H	-2.031016000	-3.323818000	0.743777000
H	-1.991490000	-0.294832000	2.888137000
H	-2.752215000	0.833290000	1.737491000
H	-3.726878000	-0.440098000	2.516974000
H	1.844496000	1.572622000	-1.798711000
H	4.098577000	1.401173000	-0.762193000

H	3.271526000	-2.709654000	0.353711000
H	1.041537000	-2.533173000	-0.664376000
H	6.556444000	-1.518967000	1.378193000
H	4.929066000	-2.077581000	1.891791000
H	5.627710000	-2.633603000	0.320182000

E(UwB97XD) = -1044.92747454

33

C	1.014597000	-1.280622000	-1.646566000
B	1.785365000	-0.309555000	-0.580848000
N	0.557824000	0.803862000	-0.148495000
C	0.232980000	1.798940000	-0.984937000
C	-0.941074000	2.513970000	-0.853612000
C	-1.834610000	2.157606000	0.184266000
C	-1.458877000	1.127445000	1.077366000
C	-0.250251000	0.492680000	0.876167000
O	2.216469000	-0.942785000	0.633258000
C	3.405636000	-0.271940000	1.066526000
C	4.027837000	0.215382000	-0.289346000
O	2.876675000	0.467951000	-1.101857000
C	3.027336000	0.891143000	1.991512000
C	4.266284000	-1.262937000	1.839317000
C	4.853807000	-0.875464000	-0.978060000
C	4.854366000	1.490396000	-0.182616000
N	-3.044648000	2.781418000	0.314287000
C	-4.093258000	2.223410000	1.150788000
C	-3.354790000	4.014925000	-0.383789000
C	-0.387523000	-1.662597000	-1.258079000
C	-0.640904000	-2.550836000	-0.207441000
C	-1.932197000	-2.785433000	0.274602000

C	-3.017905000	-2.113170000	-0.295964000
C	-2.790236000	-1.241467000	-1.369057000
C	-1.499164000	-1.030018000	-1.838837000
O	-4.308275000	-2.233555000	0.111203000
H	1.004913000	-0.783830000	-2.631170000
H	1.650552000	-2.177494000	-1.745335000
H	0.940233000	1.999793000	-1.789393000
H	-1.173445000	3.290861000	-1.578093000
H	-2.073687000	0.834025000	1.925138000
H	0.103288000	-0.304652000	1.527468000
H	2.464017000	1.675049000	1.464640000
H	2.398133000	0.509462000	2.810398000
H	3.919282000	1.355392000	2.438946000
H	5.244081000	-0.823053000	2.090859000
H	3.766543000	-1.536105000	2.781813000
H	4.432693000	-2.185068000	1.266663000
H	5.074483000	-0.556824000	-2.008326000
H	5.810020000	-1.058563000	-0.464527000
H	4.296366000	-1.822984000	-1.028760000
H	5.701657000	1.353477000	0.507664000
H	5.263728000	1.754394000	-1.170127000
H	4.249845000	2.337557000	0.168862000
H	-3.922774000	2.543512000	2.192377000
H	-5.053796000	2.622374000	0.808597000
H	-4.093005000	1.129664000	1.089611000
H	-3.827507000	3.761218000	-1.347833000
H	-4.075140000	4.575373000	0.223347000
H	-2.453765000	4.612018000	-0.549761000
H	0.199044000	-3.057512000	0.274423000

H	-2.072563000	-3.483017000	1.100797000
H	-3.643824000	-0.733789000	-1.823539000
H	-1.346276000	-0.335577000	-2.669714000
C	-4.576979000	-3.091369000	1.204696000
H	-5.658873000	-3.036515000	1.378045000
H	-4.303081000	-4.136243000	0.980670000
H	-4.049593000	-2.765284000	2.117515000

E(UwB97XD) = -1178.89544365

34

B	-0.518886000	-0.000030000	0.000211000
N	0.985706000	-0.000008000	0.000217000
C	1.647576000	-1.162145000	0.192327000
C	3.026152000	-1.196253000	0.201754000
C	3.721073000	0.000009000	0.000007000
C	3.026110000	1.196266000	-0.201602000
C	1.647535000	1.162141000	-0.191981000
O	-1.210186000	1.137560000	-0.180733000
C	-2.610748000	0.778983000	0.114509000
C	-2.610749000	-0.778977000	-0.114622000
O	-1.210224000	-1.137594000	0.181015000
C	-2.854523000	1.179313000	1.564144000
C	-3.512895000	1.555679000	-0.824110000
C	-2.854062000	-1.179310000	-1.564328000
C	-3.513135000	-1.555676000	0.823748000
C	5.161730000	0.000006000	-0.000134000
N	6.319685000	0.000033000	-0.000250000
H	1.038369000	-2.053028000	0.337261000
H	3.544961000	-2.139471000	0.361078000
H	3.544884000	2.139495000	-0.360978000

H	1.038308000	2.053023000	-0.336821000
H	-2.203978000	0.623884000	2.256296000
H	-2.646096000	2.252435000	1.680800000
H	-3.901132000	0.999024000	1.846851000
H	-4.555689000	1.231557000	-0.690763000
H	-3.456763000	2.628788000	-0.591257000
H	-3.232231000	1.413236000	-1.875403000
H	-2.645640000	-2.252441000	-1.680912000
H	-3.900568000	-0.998978000	-1.847395000
H	-2.203254000	-0.623903000	-2.256253000
H	-4.555901000	-1.231584000	0.690093000
H	-3.456907000	-2.628785000	0.590922000
H	-3.232783000	-1.413208000	1.875121000

E(RwB97XD) = -751.707879590

35

B	0.048540000	0.000026000	-0.000052000
N	-1.461307000	0.000020000	-0.000081000
C	-2.114508000	1.186332000	0.154395000
C	-3.499955000	1.193943000	0.158307000
C	-4.195806000	-0.000018000	0.000027000
C	-3.499934000	-1.193963000	-0.158287000
C	-2.114488000	-1.186314000	-0.154483000
O	0.747232000	-0.355917000	1.093839000
C	2.149181000	-0.437596000	0.656100000
C	2.149244000	0.437595000	-0.656072000
O	0.747305000	0.355950000	-1.093908000
C	3.028520000	0.096785000	1.770759000
C	2.433011000	-1.913699000	0.404486000
C	3.028632000	-0.096825000	-1.770665000

C	2.433094000	1.913692000	-0.404457000
C	-1.292578000	2.424773000	0.310521000
H	-4.021843000	2.141012000	0.286401000
H	-5.286718000	-0.000038000	0.000067000
H	-4.021814000	-2.141043000	-0.286332000
C	-1.292541000	-2.424754000	-0.310517000
H	2.724965000	1.103149000	2.086233000
H	2.969486000	-0.571607000	2.641852000
H	4.076373000	0.131551000	1.437700000
H	3.485561000	-2.066312000	0.127177000
H	2.236213000	-2.480462000	1.326021000
H	1.797334000	-2.323203000	-0.393879000
H	2.969705000	0.571569000	-2.641764000
H	4.076458000	-0.131644000	-1.437524000
H	2.725047000	-1.103175000	-2.086154000
H	3.485647000	2.066273000	-0.127133000
H	2.236331000	2.480451000	-1.326001000
H	1.797424000	2.323226000	0.393895000
H	-1.941173000	-3.276771000	-0.543685000
H	-0.558778000	-2.313613000	-1.123570000
H	-0.745613000	-2.643533000	0.618822000
H	-0.745431000	2.643495000	-0.618696000
H	-1.941248000	3.276810000	0.543511000
H	-0.559009000	2.313623000	1.123751000

E(RwB97XD) = -738.127748869

36

B	-0.119404000	-0.000037000	0.000679000
N	-1.617313000	0.000026000	0.000706000
C	-2.283318000	1.168817000	-0.151514000

C	-3.661961000	1.196064000	-0.156800000
C	-4.362316000	0.000006000	-0.000579000
C	-3.662108000	-1.196036000	0.156379000
C	-2.283462000	-1.168750000	0.152361000
O	0.577585000	-1.128040000	0.231135000
C	1.975827000	-0.783410000	-0.078160000
C	1.975876000	0.783410000	0.077982000
O	0.577383000	1.127913000	-0.230049000
C	2.877868000	-1.515779000	0.896265000
C	2.224335000	-1.250516000	-1.507012000
C	2.877022000	1.515680000	-0.897349000
C	2.225515000	1.250666000	1.506589000
H	-1.669485000	2.061047000	-0.266445000
H	-4.177312000	2.146905000	-0.281425000
H	-5.453143000	0.000016000	-0.001148000
H	-4.177526000	-2.146897000	0.280572000
H	-1.669702000	-2.060948000	0.268008000
H	2.597010000	-1.323533000	1.939582000
H	2.820655000	-2.598647000	0.714093000
H	3.921285000	-1.199858000	0.748516000
H	3.271912000	-1.084570000	-1.795191000
H	2.015279000	-2.327828000	-1.574416000
H	1.576333000	-0.727330000	-2.225933000
H	2.820411000	2.598536000	-0.714963000
H	3.920484000	1.199284000	-0.750968000
H	2.594809000	1.323613000	-1.940345000
H	2.016322000	2.327953000	1.574053000
H	1.578131000	0.727454000	2.226057000
H	3.273336000	1.084918000	1.793956000

E(RwB97XD) = -659.481709493

37

B	-1.004486000	0.000001000	0.000426000
N	0.470922000	0.000013000	0.000512000
C	1.164938000	-1.171009000	0.098539000
C	2.525473000	-1.211407000	0.096814000
C	3.280765000	0.000014000	0.000156000
C	2.525444000	1.211425000	-0.096394000
C	1.164913000	1.171041000	-0.097775000
O	-1.713699000	1.121373000	-0.260463000
C	-3.107037000	0.785803000	0.051873000
C	-3.106979000	-0.785819000	-0.052112000
O	-1.713822000	-1.121345000	0.261098000
C	-3.368920000	1.300428000	1.462841000
C	-4.006644000	1.484520000	-0.950786000
C	-3.367958000	-1.300457000	-1.463246000
C	-4.007221000	-1.484560000	0.949961000
N	4.613441000	0.000018000	0.000006000
C	5.353615000	1.256629000	-0.042977000
C	5.353590000	-1.256638000	0.042284000
H	0.562264000	-2.074843000	0.176527000
H	3.010385000	-2.180535000	0.180503000
H	3.010341000	2.180530000	-0.180447000
H	0.562217000	2.074864000	-0.175695000
H	-2.724491000	0.804100000	2.203684000
H	-3.161622000	2.379910000	1.494956000
H	-4.418088000	1.143207000	1.750518000
H	-5.051440000	1.175147000	-0.798454000
H	-3.949441000	2.573191000	-0.806355000

H	-3.720507000	1.257197000	-1.985664000
H	-3.160595000	-2.379931000	-1.495223000
H	-4.416953000	-1.143282000	-1.751583000
H	-2.723094000	-0.804114000	-2.203699000
H	-5.051914000	-1.175144000	0.797005000
H	-3.949976000	-2.573225000	0.805514000
H	-3.721702000	-1.257283000	1.985021000
H	5.062734000	1.911464000	0.791775000
H	6.422020000	1.038271000	0.047362000
H	5.181705000	1.786159000	-0.992636000
H	5.063318000	-1.910686000	-0.793313000
H	6.422063000	-1.038190000	-0.046987000
H	5.180967000	-1.787068000	0.991298000

E(RwB97XD) = -793.483611100

38

C	-0.134901000	-1.098314000	-1.643931000
B	-1.122299000	-0.697799000	-0.401083000
N	-0.592454000	0.682877000	0.230719000
C	0.331916000	0.727192000	1.245482000
C	1.012111000	1.860041000	1.610568000
C	0.808519000	3.110966000	0.933395000
C	-0.178976000	3.089837000	-0.118905000
C	-0.822433000	1.875959000	-0.390663000
O	-2.503313000	-0.514640000	-0.884928000
C	-3.380956000	-1.002339000	0.125015000
C	-2.534701000	-2.159055000	0.764286000
O	-1.201031000	-1.675423000	0.681225000
C	-3.689047000	0.120065000	1.125225000
C	-4.680304000	-1.455184000	-0.532101000

C	-2.630338000	-3.458227000	-0.046232000
C	-2.870727000	-2.449256000	2.223518000
C	1.308358000	-1.220665000	-1.249261000
C	2.206563000	-0.150693000	-1.399943000
C	3.521922000	-0.227320000	-0.956996000
C	3.995156000	-1.394609000	-0.344050000
C	3.124779000	-2.477534000	-0.188019000
C	1.803532000	-2.375963000	-0.635939000
O	5.295469000	-1.377958000	0.056917000
C	5.817234000	-2.552840000	0.644547000
C	1.517309000	4.271434000	1.272430000
N	2.103823000	5.251213000	1.549620000
H	-0.497636000	-2.054480000	-2.059447000
H	-0.238657000	-0.343747000	-2.444121000
H	0.496968000	-0.225668000	1.747546000
H	1.729698000	1.796963000	2.429690000
H	-2.791606000	0.422262000	1.682877000
H	-4.056484000	0.999718000	0.573376000
H	-4.465395000	-0.176952000	1.847420000
H	-5.336766000	-1.961752000	0.193407000
H	-5.225379000	-0.582280000	-0.925547000
H	-4.490234000	-2.138335000	-1.371129000
H	-1.857998000	-4.157483000	0.310795000
H	-3.610377000	-3.949273000	0.061859000
H	-2.450021000	-3.271408000	-1.115301000
H	-3.928900000	-2.733690000	2.341164000
H	-2.254659000	-3.285468000	2.590593000
H	-2.667938000	-1.578841000	2.862455000
H	1.861651000	0.775588000	-1.868284000

H	4.204754000	0.616402000	-1.078123000
H	3.457341000	-3.403896000	0.281289000
H	1.134340000	-3.227532000	-0.490263000
H	6.864777000	-2.333952000	0.887593000
H	5.284135000	-2.822125000	1.572680000
H	5.782959000	-3.408256000	-0.052321000
C	-0.741296000	4.050604000	-1.019002000
C	-1.684943000	3.391544000	-1.779501000
N	-1.721584000	2.070256000	-1.394411000
H	-2.365240000	1.333419000	-1.678000000
H	-0.479212000	5.103650000	-1.094701000
H	-2.338773000	3.756016000	-2.566846000

E(UwB97XD) = -1269.04496736

39

C	-0.084611000	-0.551366000	-1.846291000
B	-1.152863000	-0.129500000	-0.677157000
N	-0.751393000	1.344910000	-0.137526000
C	0.081096000	1.608691000	0.962974000
C	0.458936000	2.944683000	1.263894000
C	0.020230000	3.997916000	0.503994000
C	-0.840522000	3.711970000	-0.612708000
C	-1.173467000	2.409928000	-0.869516000
O	-2.512029000	-0.077798000	-1.222253000
C	-3.400370000	-0.552900000	-0.220973000
C	-2.513639000	-1.594689000	0.547103000
O	-1.214284000	-1.031839000	0.469214000
C	-3.842364000	0.611455000	0.677699000
C	-4.630717000	-1.146794000	-0.900357000
C	-2.490609000	-2.961728000	-0.150041000

C	-2.885029000	-1.785540000	2.014717000
C	1.345743000	-0.561455000	-1.392535000
C	2.156489000	0.582677000	-1.491072000
C	3.451849000	0.611171000	-0.987803000
C	3.990706000	-0.518634000	-0.358827000
C	3.210212000	-1.673493000	-0.257589000
C	1.910013000	-1.680179000	-0.771909000
O	5.263533000	-0.396906000	0.108006000
C	5.832093000	-1.514159000	0.760641000
H	-0.371922000	-1.553400000	-2.209618000
H	-0.200851000	0.142097000	-2.698806000
H	1.114727000	3.106559000	2.122011000
H	-1.231646000	4.504821000	-1.250500000
H	-1.832347000	2.140593000	-1.696181000
H	-2.999623000	1.027692000	1.247685000
H	-4.252356000	1.414807000	0.045309000
H	-4.625948000	0.306054000	1.389110000
H	-5.293881000	-1.637811000	-0.170019000
H	-5.207650000	-0.348939000	-1.394841000
H	-4.349602000	-1.880934000	-1.667781000
H	-1.686565000	-3.570837000	0.292110000
H	-3.438922000	-3.509966000	-0.032425000
H	-2.281504000	-2.854001000	-1.224983000
H	-3.923510000	-2.137965000	2.124887000
H	-2.223175000	-2.538063000	2.472186000
H	-2.768241000	-0.851965000	2.582028000
H	1.754743000	1.482767000	-1.965534000
H	4.066295000	1.510698000	-1.068460000
H	3.596551000	-2.573048000	0.222292000

H	1.311348000	-2.588662000	-0.667211000
H	6.841038000	-1.209645000	1.066953000
H	5.257420000	-1.801624000	1.658140000
H	5.910983000	-2.386480000	0.088675000
H	0.315468000	5.020674000	0.742188000
C	0.612055000	0.585549000	1.775302000
N	1.142414000	-0.152365000	2.517328000

E(UwB97XD) = -1137.44491644

40

C	-0.675115000	-1.133179000	-1.718221000
B	-1.450665000	-0.414309000	-0.467534000
N	-0.415214000	0.665003000	0.181656000
C	0.260040000	0.334383000	1.306877000
C	1.280783000	1.074300000	1.836380000
C	-0.053964000	1.825764000	-0.483503000
O	-2.684210000	0.241869000	-0.878291000
C	-3.637265000	0.073635000	0.161181000
C	-3.231936000	-1.316089000	0.765185000
O	-1.819391000	-1.331363000	0.615571000
C	-3.499247000	1.217833000	1.175943000
C	-5.036346000	0.116861000	-0.445927000
C	-3.812815000	-2.486890000	-0.037763000
C	-3.580484000	-1.492678000	2.239755000
C	0.671158000	-1.670195000	-1.323812000
C	1.852548000	-0.954474000	-1.581139000
C	3.090115000	-1.388761000	-1.119587000
C	3.193366000	-2.573640000	-0.379982000
C	2.034498000	-3.310557000	-0.117118000
C	0.799315000	-2.851915000	-0.586407000

O	4.444587000	-2.919326000	0.029037000
C	4.579624000	-4.090741000	0.808140000
H	-1.324866000	-1.951918000	-2.073354000
H	-0.564242000	-0.426549000	-2.558244000
H	-0.086155000	-0.590425000	1.767383000
H	1.776176000	0.741966000	2.749211000
H	-2.531978000	1.184594000	1.697044000
H	-3.558496000	2.176863000	0.637924000
H	-4.301545000	1.198840000	1.930353000
H	-5.805260000	-0.131074000	0.303435000
H	-5.249075000	1.129586000	-0.823587000
H	-5.131058000	-0.581150000	-1.289033000
H	-3.319840000	-3.418035000	0.283156000
H	-4.896983000	-2.604440000	0.116599000
H	-3.626210000	-2.358027000	-1.114353000
H	-4.665957000	-1.403036000	2.408053000
H	-3.268999000	-2.492653000	2.581549000
H	-3.067417000	-0.750250000	2.866743000
H	1.798792000	-0.021530000	-2.149227000
H	3.997584000	-0.816120000	-1.323309000
H	2.074689000	-4.238735000	0.454078000
H	-0.098933000	-3.427480000	-0.351066000
H	5.648171000	-4.181876000	1.041308000
H	4.011351000	-4.022650000	1.751876000
H	4.256916000	-4.990792000	0.256665000
C	1.705257000	2.285621000	1.180283000
C	1.017656000	2.668575000	-0.008514000
C	2.754424000	3.088029000	1.658483000
C	3.131730000	4.249871000	0.991418000

H	3.949317000	4.862518000	1.376729000
C	2.457073000	4.627773000	-0.175930000
H	2.746892000	5.537691000	-0.705788000
C	1.416135000	3.850726000	-0.669921000
H	0.899545000	4.155692000	-1.582274000
H	3.274713000	2.782814000	2.569983000
C	-0.720972000	2.192784000	-1.671189000
N	-1.182666000	2.585864000	-2.673445000

E(UwB97XD) = -1291.09933105

9 References

[1] M. Baumann, I. R. Baxendale, *Beilstein J. Org. Chem.* **2013**, *9*, 2265–2319.

[2] W. D. Wang, J. H. Espenson, *J. Am. Chem. Soc.* **1998**, *120*, 11335–11341.

[3] J. Da Chai, M. Head-Gordon, *Phys.Chem. Chem.Phys.* **2008**, *10*, 6615–6620.

[4] S. Schenker, C. Schneider, S. B. Tsogoeva, T. Clark, *J. Chem. Theory Comput.* **2011**, *7*, 3586–3595.

[5] M. A. Kabeshov, O. Kysilka, L. Rulisek, Y. V. Suleimanov, M. Bella, A. V. Malkov, P. Kocovsky, *Chem. Eur. J.* **2015**, *21*, 12026–12033.

[6] A. V. Marenich, C. J. Cramer, D. G. Truhlar, *J. Phys. Chem. B* **2009**, *113*, 6378–6396.

[7] J. N. M. J. Frisch, G. W. Trucks, H. B. Schlegel, G. E. Scuseria, M. A. Robb, J. R. Cheeseman, G. Scalmani, V. Barone, B. Mennucci, G. A. Petersson, H. Nakatsuji, M. Caricato, X. Li, H. P. Hratchian, A. F. Izmaylov, J. Bloino, G. Zheng, J. L. Sonnenberg, M. Had, J. V. O. J. C. K. Raghavachari, A. Rendell, J. C. Burant, S. S. Iyengar, J. Tomasi, M. Cossi, N. Rega, J. M. Millam, M. Klene, J. E. Knox, J. B. Cross, V. Bakken, C. Adamo, J. Jaramillo, R. Gomperts, R. E. Stratmann, O. Yazyev, A. J. Austin, R. Cammi, C. Pomelli, J. W., D. J. Fox, "Gaussian 09, Revision D.01," **2013**.

[8] Y. Yasu, T. Koike, M. Akita, *Adv. Synth. Catal.* **2012**, *354*, 3414–3420.

[9] A. Mcnally, C. K. Prier, D. W. C. Macmillan, *Science* **2011**, *334*, 1114–1117.

[10] N. Kitamura, Y. Nambu, T. Endo, *J. Polym. Sci. Part A Polym. Chem.* **1990**, *28*, 3137–3143.

[11] L. Chu, C. Ohta, Z. Zuo, D. W. C. MacMillan, *J. Am. Chem. Soc.* **2014**, *136*, 10886–10889.

[12] J. C. Tellis, D. N. Primer, G. A. Molander, *Science* **2014**, *345*, 433–436.

[13] J. W. Clary, T. J. Rettenmaier, R. Snelling, W. Bryks, J. Banwell, W. T. Wipke, B. Singaram, *J. Org. Chem.* **2011**, *76*, 9602–9610.

[14] M. A. Larsen, C. V. Wilson, J. F. Hartwig, *J. Am. Chem. Soc.* **2015**, *137*, 8633–8643.

[15] Z.-C. Cao, F.-X. Luo, W.-J. Shi, Z.-J. Shi, *Org. Chem. Front.* **2015**, *2*, 1505–1510.

[16] H. Li, L. Wang, Y. Zhang, J. Wang, *Angew. Chem. Int. Ed.* **2012**, *51*, 2943–2946.

[17] S. Roesner, C. a Brown, M. Mohiti, A. P. Pulis, R. Rasappan, D. J. Blair, S. Essafi, D. Leonori, V. K. Aggarwal, *Chem. Commun.* **2014**, *50*, 4053–4055.

[18] S. Duez, A. K. Steib, S. M. Manolikakes, P. Knochel, *Angew. Chem. Int. Ed.* **2011**, *50*, 7686–7690.

[19] R. B. Bedford, E. Carter, P. M. Cogswell, N. J. Gower, M. F. Haddow, J. N. Harvey, D. M. Murphy, E. C. Neeve, J. Nunn, *Angew. Chem. Int. Ed.* **2013**, *52*, 1285–1288.

[20] S. Bernhardt, Z. L. Shen, P. Knochel, *Chem. Eur. J.* **2013**, *19*, 828–833.

[21] D. Saha, R. Ghosh, A. Sarkar, *Tetrahedron* **2013**, *69*, 3951–3960.

[22] R. Kuwano, M. Yokogi, *Org. Lett.* **2005**, *7*, 945–947.

[23] X.-X. Wang, M.-J. Luo, J.-M. Lu, *Org. Biomol. Chem.* **2015**, *13*, 4925–4930.

[24] I. Erol, L. Özcan, S. Yurdakal, *J. Therm. Anal. Calorim.* **2013**, *114*, 377–385.

[25] Y. Yamashita, J. C. Tellis, G. A. Molander, *Proc. Natl. Acad. Sci.* **2015**, *112*, 12026–12029.

[26] X. Li, Y. Feng, L. Lin, G. Zou, *J. Org. Chem.* **2012**, *77*, 10991–10995.

[27] M.-J. Shiao, W.-L. Chia, *Synth. Commun.* **1991**, *21*, 401–406.

[28] T. Hoshikawa, M. Inoue, *Chem. Sci.* **2013**, 3118–3123.

[29] D. Collado, E. Perez-Inestrosa, R. Suau, *J. Org. Chem.* **2003**, *68*, 3574–84.

[30] E. Reimann, A. Höglmüller, *Arch. Pharm.* **1985**, *318*, 487–495.

[31] S. Thapa, A. Kafle, S. K. Gurung, A. Montoya, P. Riedel, R. Giri, *Angew. Chem. Int. Ed.* **2015**, 8236–8240.

[32] G. Song, Y. Su, X. Gong, K. Han, X. Li, *Org. Lett.* **2011**, *13*, 1968–1971.

[33] S. Panda, A. Coffin, Q. N. Nguyen, D. J. Tantillo, J. M. Ready, *Angew. Chem. Int. Ed.* **2015**, *55*, 2205–2209.
